# Systematic review and meta-analysis of the epidemiology of Lassa virus in humans, rodents and other mammals in sub-Saharan Africa

**DOI:** 10.1371/journal.pntd.0008589

**Published:** 2020-08-26

**Authors:** Sebastien Kenmoe, Serges Tchatchouang, Jean Thierry Ebogo-Belobo, Aude Christelle Ka'e, Gadji Mahamat, Raïssa Estelle Guiamdjo Simo, Arnol Bowo-Ngandji, Cynthia Paola Demeni Emoh, Emmanuel Che, Dimitri Tchami Ngongang, Marie Amougou-Atsama, Nathalie Diane Nzukui, Chris Andre Mbongue Mikangue, Donatien Serge Mbaga, Sorel Kenfack, Sandrine Rachel Kingue Bebey, Nathalie Amvongo Adjia, Atembeh Noura Efietngab, Hervé Raoul Tazokong, Abdou Fatawou Modiyinji, Cyprien Kengne-Nde, Serge Alain Sadeuh-Mba, Richard Njouom

**Affiliations:** 1 Department of Virology, Centre Pasteur of Cameroon, Yaoundé, Cameroon; 2 Medical Research Centre, Institut of Medical Research and Medicinal Plants Studies, Yaoundé, Cameroon; 3 Virology Department, Chantal Biya International Reference Centre, Yaoundé, Cameroon; 4 Department of Microbiology, Faculty of Science, The University of Yaounde I, Yaoundé, Cameroon; 5 Vaccinology and Biobank, Chantal Biya International Reference Centre, Yaounde, Cameroon; 6 School of Health Sciences-Catholic University of Central Africa, Department of Medical Microbiology, Yaoundé, Cameroon; 7 Department of Animals Biology and Physiology, Faculty of Science, The University of Yaoundé I, Yaoundé, Cameroon; 8 Epidemiological Surveillance, Evaluation and Research Unit, National AIDS Control Committee, Yaoundé, Cameroon; US Department of Agriculture, UNITED STATES

## Abstract

Accurate data on the Lassa virus (LASV) human case fatality rate (CFR) and the prevalence of LASV in humans, rodents and other mammals are needed for better planning of actions that will ultimately reduce the burden of LASV infection in sub-Saharan Africa. In this systematic review with meta-analysis, we searched PubMed, Scopus, Africa Journal Online, and African Index Medicus from 1969 to 2020 to obtain studies that reported enough data to calculate LASV infection CFR or prevalence. Study selection, data extraction, and risk of bias assessment were conducted independently. We extracted all measures of current, recent, and past infections with LASV. Prevalence and CFR estimates were pooled using a random-effect meta-analysis. Factors associated with CFR, prevalence, and sources of between-study heterogeneity were determined using subgroup and metaregression analyses. This review was registered with PROSPERO, CRD42020166465. We initially identified 1,399 records and finally retained 109 reports that contributed to 291 prevalence records from 25 countries. The overall CFR was 29.7% (22.3–37.5) in humans. Pooled prevalence of LASV infection was 8.7% (95% confidence interval: 6.8–10.8) in humans, 3.2% (1.9–4.6) in rodents, and 0.7% (0.0–2.3) in other mammals. Subgroup and metaregression analyses revealed a substantial statistical heterogeneity explained by higher prevalence in tissue organs, in case-control, in hospital outbreak, and surveys, in retrospective studies, in urban and hospital setting, in hospitalized patients, and in West African countries. This study suggests that LASV infections is an important cause of death in humans and that LASV are common in humans, rodents and other mammals in sub-Saharan Africa. These estimates highlight disparities between sub-regions, and population risk profiles. Western Africa, and specific key populations were identified as having higher LASV CFR and prevalence, hence, deserving more attention for cost-effective preventive interventions.

## Author summary

Lassa virus (LASV) infection constitutes a major public health threat as it has a direct impact on the mortality of febrile patients, healthcare workers, pregnant women, visitors of endemic countries with a consequential negative impact on national and individual economies. It is necessary to have accurate epidemiological data on LASV infection, in order to prioritize the policies, funding for public health interventions, and health-care planning, especially in sub-Saharan Africa (SSA). Data concerning virus occurrence in rodents and other mammal species can also assist in guiding control of Lassa fever from an ecological perspective. This could include preventive measures such as the protection of food fields by anti-rodent barriers, the protection of rodent predatory fauna, the sanitation of home and particularly the food storage place and the blocking of rodent access to home and food stored. Our systematic review and meta-analysis of LASV infection in SSA has contemporarily and comprehensively summarized the prevalence of current, recent, and past infection both in humans, rodents and other mammals. Broadly, the study showed relatively high CFR estimates in humans. Additionally, Western Africa presented the highest burden of LASV infections compared to other regions. This study emphasized various populations of SSA with relatively high burden of LASV infection. We have identified specific populations at high risk of LASV infection, who may urgently benefit from routine screening, detection and management programmes. Beyond, preventive strategies should be promoted, by educating and raising people’s awareness about LASV infection, and strengthening practitioners’ capacities towards adequate diagnosis and proper management of this infection in SSA.

## Introduction

At least 75% of emerging and re-emerging infectious diseases have an animal origin [1]. Lassa virus (LASV) is a zoonotic re-emerging pathogen and a member of the family *Arenaviridae* and the genus *Mammarenaviruses* (viruses that infect mammals). The main natural reservoir of LASV is the rodent *Mastomys natalensis* that lives in proximity with humans [2–4]. A recent study showed that up to 37.7 million people in 14 countries in sub-Saharan Africa (SSA), particularly in West Africa, were living in areas that were prone to LASV zoonotic transmission, because of the presence of rodent reservoirs in these countries [5]. The LASV is also found in other rodents and mammals species in the West Africa [6]. This presence of LASV in non-reservoirs could however represent transient or spillover infections [7]. Humans are infected either by direct contact with infected tissues, urine or excrement of rodents or indirectly through vectors such as cereals dried on the ground. Lassa virus is also transmitted from human to human by direct contact with secretions or infected blood, especially in hospitals with relatively high case fatality rates [8,9].

The LASV was isolated for the first time from two missionary nurses in the city of Lassa in north-eastern Nigeria in 1969 [10]. It is estimated that Lassa fever is responsible for 2 million infections annually in West Africa [11]. A systematic review has shown that 21 Lassa fever outbreaks recorded in Nigeria from 1969 to 2017 have been associated with approximately 6,000 suspected cases, 800 confirmed cases and nearly 700 deaths [12]. These epidemics of Lassa fever pose a constant problem in health facilities with nosocomial transmissions to health care workers and visitors of infected cases [8]. Another recent review reported 33 patients with imported cases of LASV returning from 7 West African countries and 9 other countries between 1969 and 2016 [13]. A total of 39% (12/31) of the patients with known outcomes were deceased. Lassa fever virus is listed among the WHO priority diseases in need of urgent research and development efforts and it is classified as a category “A” bioterrorism agent that can serve as biological weapons [14].

Lassa virus infection includes a wide variety of clinical presentations resulting in mild or severe forms that require medical attention and often lead to death. Asymptomatic infections also seem common. The early phase of Lassa fever is indistinguishable from other common febrile syndromes such as malaria, typhoid fever or haemorrhagic fevers caused by other viruses. Laboratory-based surveillance programs are essential for the prevention, management and control of outbreaks of LASV infections [15]. Early diagnosis and treatment are associated with better outcomes for patients with LASV [16]. Molecular assays are widely used in reference laboratories for LASV diagnosis [15,17]. However, there are still many challenges to the laboratory diagnosis of LASV including the wide diversity of LASV strains and sequences, the low sensitivity and specificity of immunoassays, and the low number of approved rapid diagnostic tests. Furthermore, there is also a possibility of cross-reaction between LASV and other Arenaviruses strains. Although promising results have been reported in the preclinical phase of the vaccine development [18], there are currently no approved vaccines against LASV infection [19]. The management of LASV fever patients is primarily mainly based on alleviating symptoms. However, encouraging results have been obtained with the administration of ribavirin in the initial phase (first week) of the disease [16].

It is recognized that the fight against zoonotic diseases can be done ideally according to the One Health approach [20]. To this end, epidemiological data from zoonotic viruses such as LASV in humans and animals are crucial in guiding common responses to this health threat. Furthermore, a recent study reported that the detection rate of the LASV in asymptomatic individuals and the identification of populations at high risk were at crucial importance [21]. We explored both the case fatality rate of the LASV in various category of human populations and the prevalence of LASV in humans, rodents and others mammals. This is important information that can inform priorities in focussing prevention efforts. Data concerning virus occurrence in rodents and other mammal species can also assist in guiding control of Lassa fever from an ecological perspective.

## Methods

### Design and inclusion criteria

The preferred reporting items for systematic reviews and meta–analyses (PRISMA) checklist was used for the design of this systematic review (S1 Table) [22]. This review was published to Prospero under the identification CRD42020166465. Inclusion criteria were met for studies including subjects of all ages and gender with any illness, apparently healthy individuals, rodents or other mammals. The LASV clinical case definitions present a wide variability on multiple factors such as the definition of fever, other symptoms considered, non-response to treatment for other febrile illnesses endemic in sub-Saharan Africa, past contact with confirmed, suspect or probable cases of LASV patients or rodents and the organisation considered for guidelines. We therefore categorized the types of participants according to the inclusion criteria of the included studies. We classified the other mammals according to the highest level listed in the included articles and in agreement with the mammal classification proposed by Wilson and Reeder in 2005 [23]. Studies reporting the prevalence of LASV (virus, antigen, RNA, IgM and/or IgG) or data to calculate this estimate were included. Lassa virus prevalence was determined using any detection assays including indirect immunofluorescence, complement fixation, culture, Reverse Transcription Polymerase Chain Reaction (RT-PCR) or Enzyme Linked Immunosorbent Assay (ELISA). We selected studies conducted in SSA, as defined by the United Nations Statistics Division (UNSD), that represent the habitat of *Mastomys natalensis*, natural hosts of LASV [5]. All study types published in peer-reviewed journals, observational or interventional, providing cross-sectional records on the prevalence of one or more LASV markers were included. We used a conceptual definition for the included study designs, considering surveys and surveillance as a cross-sectional study. Given that epidemic context is usually associated with high prevalences, we presented epidemics as community outbreak and hospital outbreak. Studies reporting imported case data, case reports, reports, reviews, systematic reviews and meta-analyses, comments and duplicate were excluded.

### Data sources and search strategy

A comprehensive search strategy designed to allow exhaustive identification of relevant studies was applied to the Pubmed, Scopus, African Journals Online, and African Index Medicus databases. Databases were consulted from 1969 to 2020 for studies published in English and French languages. The electronic search strategy was adapted to each database. S2 Table displays the details of the search strategy applied in the Pubmed database. The list of references from the included studies and other relevant articles were manually screened for other relevant studies.

### Selection and extraction of data from included studies

Duplicates identified from the complete list of studies were removed. The titles and abstracts of the eligible studies were independently examined by two study authors (SK and JTEB) for the selection of relevant studies. The differing opinions of the investigators regarding the selection of the studies were resolved by discussion, consensus and intervention of a third arbitrator if necessary. Data from the included studies was extracted using a google form by 18 study authors and verified by SK. The extracted data were the name of the first author, the year of publication, the study design, the inclusion criteria (ill, apparently healthy, rodent or other mammals), country, sampling method, study period, LASV detection assay, LASV detection marker (virus, antigen, RNA, IgM or IgG), type of sample used for LASV detection, age and gender of study participants, sample size, number of positive for LASV, and number of deaths within LASV positive. Disagreements observed during data extraction were resolved by discussion and consensus.

### Study definitions

Studies performed in several hospitals or cities were defined as multicentric and those in a single hospital or city were defined as monocentric. We grouped the countries in accordance with the breakdown of the UNSD [24]. We considered studies that performed analysis on acute and convalescent samples in order to give a diagnosis of the infection by the rise in the IgG antibody titer. Past infections were considered for articles with unspecified antibodies or IgG positive subjects. Recent infections, contact lasting up to 12 months, were considered for articles with IgM or IgM and IgG positive subjects. Current infections were considered for studies with antigen, RNA, virus positives or subjects with an increase in antibody titer between acute and convalescent samples. People with regular contacts with any animal were defined as high risk individuals. We assumed the data of the various population categories from the same article as several individual prevalences.

### Evaluation of the quality of studies and synthesis of data

The quality of the included studies was independently assessed by all study authors using a dedicated scale for prevalence studies that is based on 10 components divided into two groups: internal and external validity of the study (S3 Table) [25]. The scores of 0 or 1 were assigned to each question in the assessment tool for a total score of 10 per study. The scores of 0–3, 4–6 and 7–10 represented a high, moderate and low risk of bias, respectively. The disagreements were resolved by discussion and consensus. The R version 3.6.0 software (package "meta" and "metafor") through the RStudio interface was used to perform all meta-analyses [26,27]. Heterogeneity between studies was estimated by the Cochran Q test, and the I^2^ and the H statistics [28]. Studies were considered to lack of evidence on heterogeneity if the p value for Q test was greater than 0.05. The I2 value was indicative of the degree of heterogeneity with values ​​of 0%, 18%, 45%, and 75% considered for none, low, moderate and high heterogeneity respectively [29]. H values ​​close to 1 indicated lack of evidence on heterogeneity between studies and these values ​​were inversely correlated with degree of heterogeneity. Prevalence, 95% confidence intervals (95% CI), and prediction intervals were estimated by random effect models (DerSimonian-Laird method) [30]. The instability of the variance and the problem of the 95% CIs excluded from the range 0 to 1 were solved by the Freeman-Tukey double arcsine stabilization. Potential reasons for heterogeneity were investigated by subgroup and metaregression analyses by study design, the sampling method, the timing of data collection, the country, the UNSD region, the setting, the hospitalization, and the sample types. The dependent variable was the LASV prevalence or case fatality rate. We considered only dependent variables with at least 3 studies divided into two or more categories of the independent variables in subgroup analyses and metaregression. The independent variables were evaluated in both a univariate and a multivariate metaregression model (if p <0.2 in the univariate model). The statistical significance threshold was 0.05 in multivariate meta-regression analysis. The publication bias was assessed by visual inspection of the asymmetry of the funnel plot and the Egger test with the value of p <0.1 indicating a potential bias [31]. As cross-sectional studies are best for prevalence studies [32], a sensitivity analysis that included only low-risk of bias and cross-sectional studies was performed.

## Results

### Literature search

**Fig 1:**
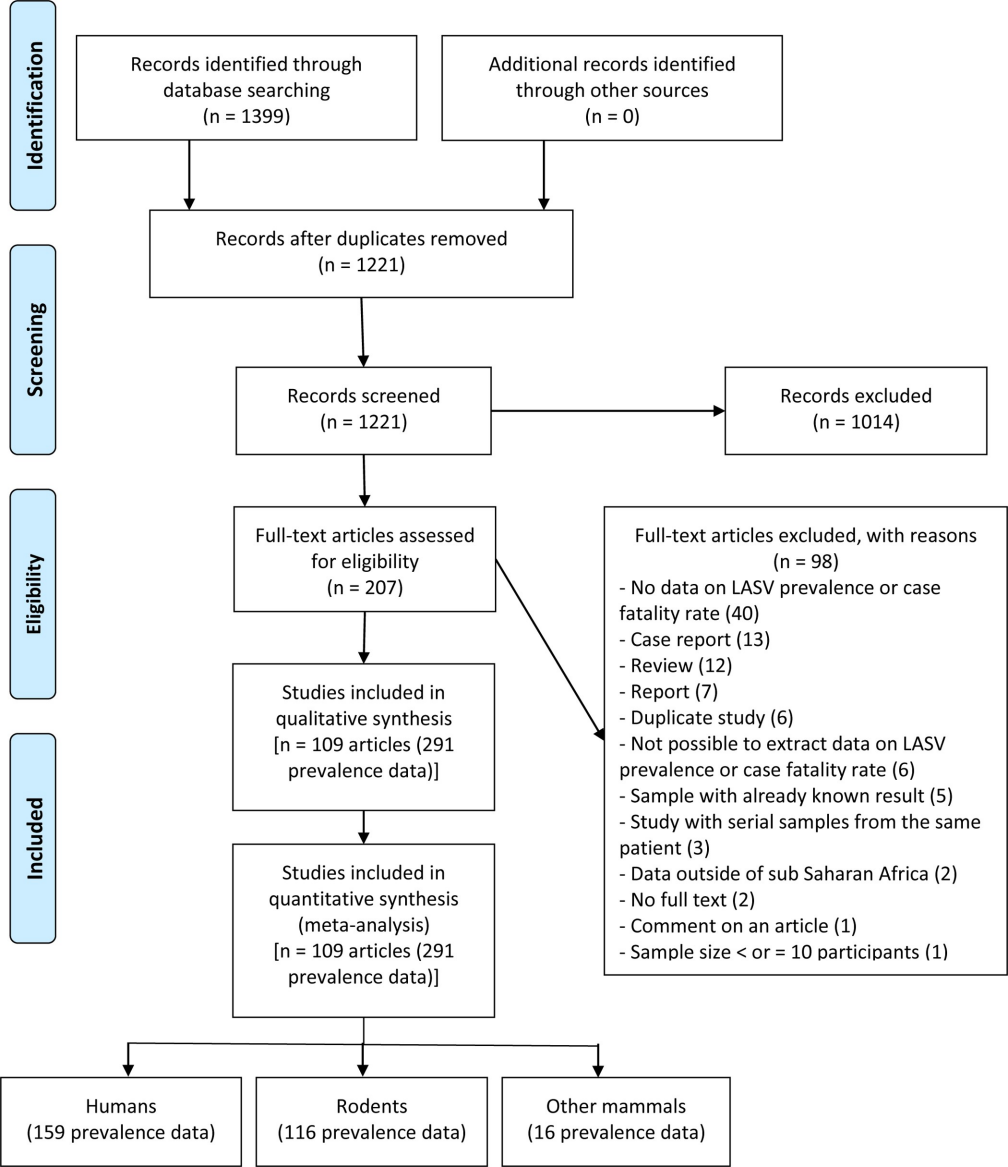
Flow-chart describing study selection process.

A total of 1,399 articles were identified by electronic search in the databases and 178 duplicates eliminated (Fig 1 and S4 Table). A group of 1,014 were eliminated after examination of the titles and abstracts, leaving a total of 207 articles to be examined completely. Of the 207 articles examined completely, 109 (291 prevalence records) met the inclusion criteria, including 159 in humans, 116 in rodents, and 16 in other mammals [2–4,6,8,9,33–135].

### Study quality assessment

The majority of articles in humans had a moderate risk of bias (112/159; 70.4%), followed by studies with a low risk of bias (46/159; 28.9%), and a high risk of bias (1/159; 0.6%). All articles in rodents and other mammals had a moderate risk of bias (S5 Table). No study was representative of the population of a country, no study described the rate of non-respondents and only 16 performed probabilistic sampling approach.

#### Characteristics of studies included in meta-analysis

Twenty-five countries in SSA were represented in this review. Most of the prevalence records were from West Africa (86.6%; 252/291) and more particularly in Nigeria (31.3%; 91/291) (S6 Table). Study participants were recruited between 1965 and 2019. Two hundred and fifty-eight (88.6%; 258/291) prevalence records were cross-sectional, 106 (36.4%) were rural, 192 (66.0%) were community based, 99 (34.0%) had tested for antibodies by indirect immunofluorescence, 225 (77.3%), and had tested for LASV in serum alone. Fifty-seven prevalence records (35.9%; 57/159) focused on apparently healthy individuals and 13 (11.2%; 13/116) on *Mastomys natalensis* rodents. Only 23 of the human included studies gave a LASV suspected or probable case definition of the recruited participants. Individual data from included studies are presented in the S7 Table.

### Meta-analysis

The included studies in this review recruited 114,848 participants, including 91,275 humans, 21,891 rodents, and 1,348 other mammals.

#### Case fatality rate of Lassa virus infections in humans

**Fig 2:**
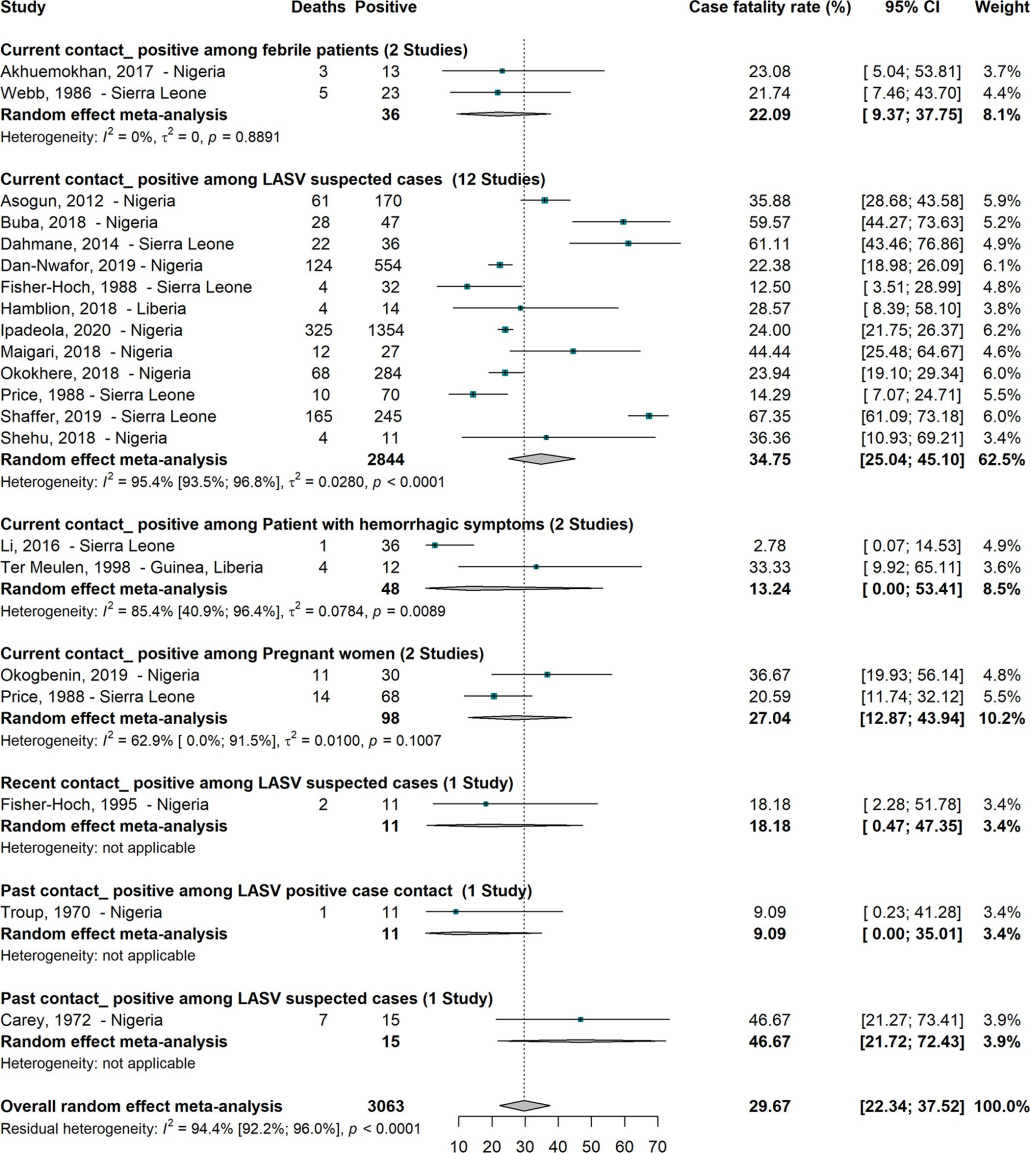
Human case fatality rate due to Lassa virus in sub-Saharan Africa.

A total of 3063 participants recruited from 20 studies conducted in West Africa gave an overall fatality rate of 29.6% (95% CI; 22.3–37.5) (Fig 2) [8,34,37,47,49,52,53,65,70,75,87,89,100,101,109,116,117,120,123,132]. All categories of participants with common, recent or old infections had relatively high case fatality rates ranging from 9.1 to 46.7%. We obtained decreasing case fatality records from participants with current LASV infections in LASV suspected cases 34.7% (95% CI; 25.0–45.1), pregnant women 27.0% (95% CI; 12.9–43.9), febrile patients 22.1% (95% CI; 9.4–37.7), and patients with haemorrhagic symptoms 13.2% (95% CI; 0.0–53.4).

### Prevalence of Lassa virus infections in humans

**Fig 3:**
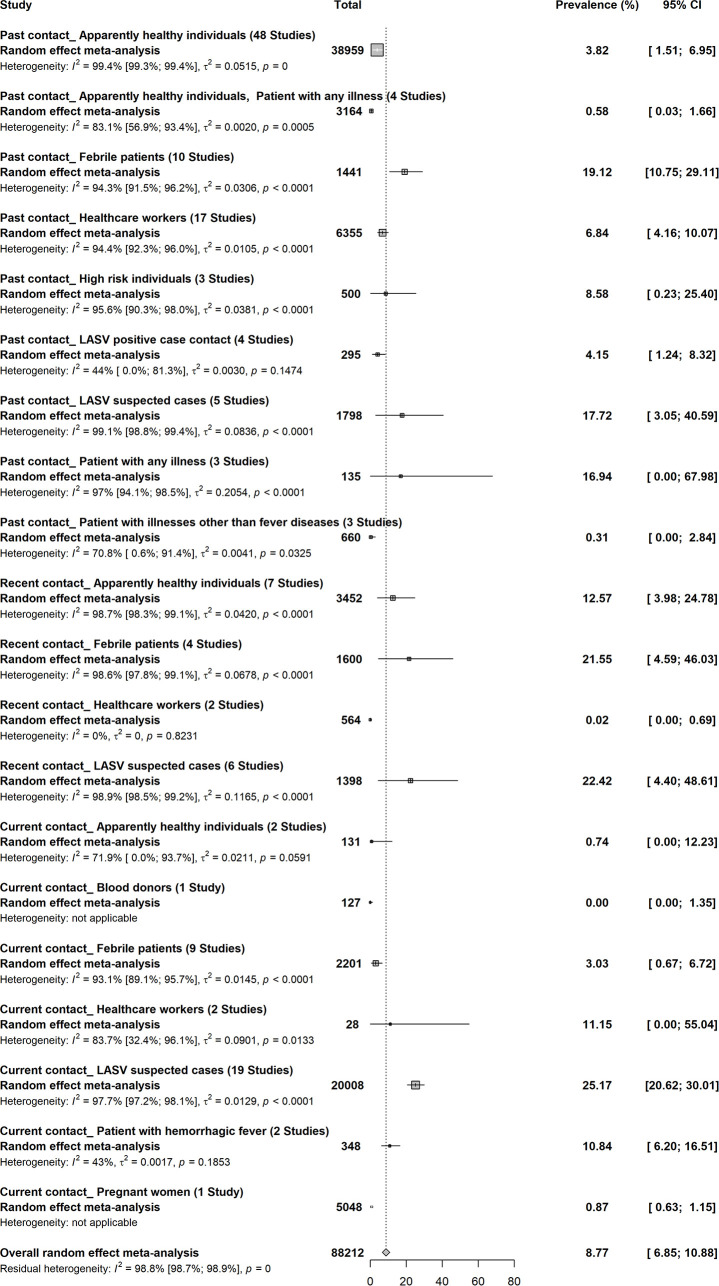
Prevalence of Lassa virus infections in humans in sub-Saharan Africa.

A total of 88,212 participants enrolled in 82 studies gave an overall prevalence of LASV of 8.7% (95% CI; 6.8–10.8) (Figs 3 and S1) [8,9,34–46,48–50,52,53,56–61,65–67,67–70,72–84,87–89,91–97,99,100,105–108,110,113,115–123,126–130,132–136]. Regardless of the type of infection; current, recent or past, LASV suspected cases and febrile patients had the highest prevalence. In studies with participants with evidence of LASV current infection, healthcare workers (11.1%) had high prevalence. In studies with participants with evidence of LASV recent infection, apparently healthy individuals (12.6%) had high prevalence. In studies with participants with evidence of previous LASV infection patients with any disease (16.9%) had high prevalence. Apart from West Africa where current and recent LASV infections have been recorded, only past infections evidence has been recorded in other regions of SSA including Central Africa (Cameroon, Central African Republic, Guinea Equatorial, Republic of Congo and Democratic Republic of Congo), Eastern Africa (Zimbabwe and Uganda) and Northern Africa (Sudan).

### Prevalence of Lassa virus infections in rodents

**Fig 4:**
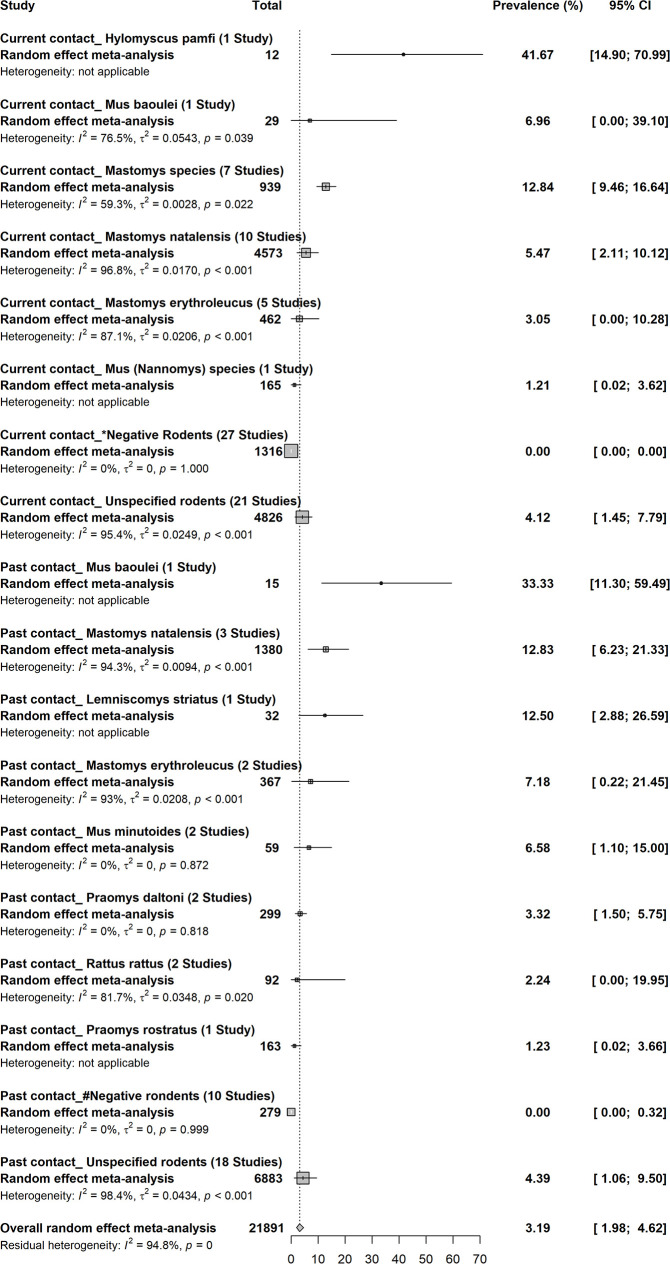
Prevalence of Lassa virus infections in rodents in sub-Saharan Africa. *Negative rodents include *Gerbilliscus species*, *Lemniscomys species*, *Praomys daltoni*, *Praomys species*, *Rattus rattus*, *Uranomys ruddi*, *Nannomys minutoides/mattheyi*, *Myomys daltoni*, *Praomys rostratus*, *Lemniscomys striatus*, *Praomys cf*. *rostratus*, *Crocodura species*, *Lophuromys sikapusi*, *Tatera cf*. *guinea*, *Gerbilliscus kempi*, *Praomys jacksoni*, *Mus setulosus*, and *Mus minutoides*. #Negative rodents include *Gerbilliscus kempi*, *Mus setulosus*, *Praomys jacksoni*, *Lemniscomys bellieri/zebra*, *Uranomys ruddi*, *Crocidura buettikoferi*, *Gerbilliscus guineae*, *Lophuromys sikapusi*, and *Mus mattheyi*.

A total of 21,891 rodents trapped in 29 studies gave an overall prevalence of LASV of 3.2% (95% CI; 2.0–4.6) (Figs 4 and S2) [2–4,6,33,43,45,49,51,54,55,62–64,69,85,86,90,94,102–104,111,112,114,124,125,129,130]. Besides the *Mastomys species*, more particularly *Mastomys natalensis*, many other rodent species had evidence of current or previous contacts with LASV. In one study, the species *Hylomyscus pamfi* showed the highest prevalence of current contact at 41.7% [6]. In terms of current and past contacts, *Mus* species have also shown relatively high prevalence of LASV [3,63,103,125]. *Lemniscomys striatus*, *Rattus rattus*, and *Praomys species* also had relatively high evidence of previous contact with LASV [63,103].

### Prevalence of Lassa virus infections in other mammals

**Fig 5:**
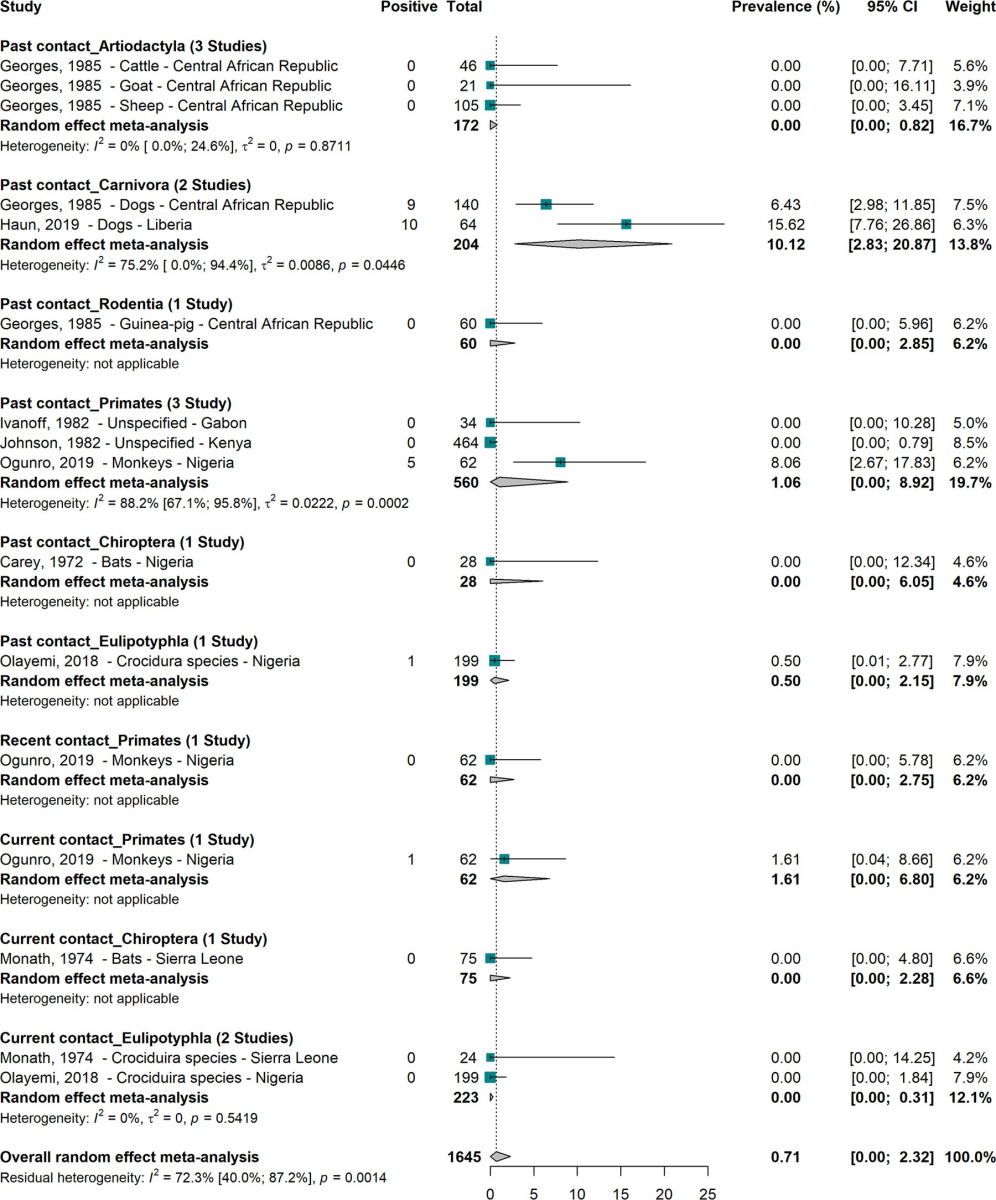
Prevalence of Lassa virus infections in other mammals in sub-Saharan Africa.

A total of 1645 other mammals recruited in 8 studies gave an overall prevalence of LASV of 0.7% (95% CI; 0.0–2.3) (Fig 5) [4,49,68,71,77,81,98,103]. Only primates, carnivora and Eulipotyphla showed evidence of current or previous contact with LASV [68,71,98,103].

### Sensitivity, heterogeneity and publication bias analysis

The overall case fatality rate in humans and the overall prevalence of LASV in humans, rodents and other mammals were similar to those estimated for cross-sectional and low risk of bias studies for the vast majority of categories (Table 1). The differences were mainly observed in the categories of febrile patients. Most study categories in rodents and other mammals had a small number of studies (<5 studies) and did not allow an objective estimate of heterogeneity. Substantial heterogeneity was recorded in the remaining included human, rodent and other mammal studies. In agreement with the funnel plot symmetry and the Egger test p value, there was no publication bias in case fatality rate in humans (p-value Egger test = 0.510), LASV prevalence in humans (p-value Egger test = 0.715), and LASV prevalence in other mammals (p-value Egger test = 0.647) analyses and evidence of publication bias in LASV prevalence in rodents (p-value Egger test = 0.045) analyses (S3–S6 Figs).

**Table 1:** Summary of meta-analysis results for prevalence of Lassa virus in humans, rodents, and other mammals in sub-Saharan Africa.

	Prevalence. % (95%CI)	95% Prediction interval	N Studies	N Participants	¶H (95%CI)	§I² (95%CI)	P heterogeneity	P Egger test
**Case fatality rate in humans**								
**Current contact**								
**Positive among LASV suspected cases**								
Overall	34.7 [25–45.1]	[4.7–73.7]	12	2844	4.7 [3.9–5.6]	95.4 [93.5–96.8]	< 0.001	0.276
Cross-sectional	35 [17–55.5]	[0–96.7]	7	1778	5.8 [4.7–7.1]	97 [95.5–98]	< 0.001	0.544
Low risk of bias	34.9 [18.2–53.6]	[0–93.6]	7	848	5 [4–6.3]	96.1 [93.8–97.5]	< 0.001	0.722
**Positive among Pregnant women**								
Overall	27 [12.9–43.9]	NA	2	98	1.6 [1–3.4]	62.9 [0–91.5]	0.101	NA
Cross-sectional	20.6 [11.7–31.1]	NA	1	68	NA	NA	1	NA
Low risk of bias	27 [12.9–43.9]	NA	2	98	1.6 [1–3.4]	62.9 [0–91.5]	0.101	NA
**Positive among Febrile patients**								
Overall	22.1 [9.4–37.7]	NA	2	36	1	0	0.889	NA
Cross-sectional	21.7 [6.9–41.2]	NA	1	23	NA	NA	1	NA
Low risk of bias	21.7 [6.9–41.2]	NA	1	23	NA	NA	1	NA
**Positive among Patient with hemorrhagic symptoms**								
Overall	13.2 [0–53.4]	NA	2	48	2.6 [1.3–5.3]	85.4 [40.9–96.4]	0.009	NA
Cross-sectional	13.2 [0–53.4]	NA	2	48	2.6 [1.3–5.3]	85.4 [40.9–96.4]	0.009	NA
Low risk of bias	13.2 [0–53.4]	NA	2	48	2.6 [1.3–5.3]	85.4 [40.9–96.4]	0.009	NA
**Recent contact**								
**Positive among LASV suspected cases**								
Overall	18.2 [0.5–47.4]	NA	1	11	NA	NA	1	NA
Cross-sectional	18.2 [0.5–47.4]	NA	1	11	NA	NA	1	NA
**Past contact**								
**Positive among LASV suspected cases**								
Overall	46.7 [21.7–72.4]	NA	1	15	NA	NA	1	NA
**Positive among LASV Positive case contact**								
Overall	9.1 [0–35]	NA	1	11	NA	NA	1	NA
**LASV prevalence in humans**								
**Current contact**								
**LASV suspected cases**								
Overall	25.2 [20.6–30]	[7.4–48.8]	20	20008	6.6 [5.9–7.3]	97.7 [97.2–98.1]	< 0.001	0.061
Cross-sectional	24.8 [19.6–30.4]	[6.2–50.2]	16	15929	6.5 [5.8–7.4]	97.7 [97–98.2]	< 0.001	0.092
Low risk of bias	34.5 [24.6–45.1]	[4.6–73.7]	10	6452	6.3 [5.4–7.4]	97.5 [96.6–98.2]	< 0.001	< 0.001
**Healthcare workers**								
Overall	11.1 [0–55]	NA	2	28	2.5 [1.2–5]	83.7 [32.4–96.1]	0.013	NA
Cross-sectional	31.3 [10.5–56.4]	NA	1	16	NA	NA	1	NA
Low risk of bias	31.3 [10.5–56.4]	NA	1	16	NA	NA	1	NA
**Patient with hemorrhagic symptoms**								
Overall	10.8 [6.2–16.5]	NA	2	348	1.3	43	0.185	NA
Cross-sectional	12.9 [9.2–17.2]	NA	1	278	NA	NA	1	NA
Low risk of bias	12.9 [9.2–17.2]	NA	1	278	NA	NA	1	NA
**Febrile patients**								
Overall	3 [0.7–6.7]	[0–21.7]	9	2201	3.8 [3–4.8]	93.1 [89.1–95.7]	< 0.001	0.1
Cross-sectional	2.6 [0.3–6.6]	[0–23.7]	7	1927	4.1 [3.2–5.3]	94.2 [90.3–96.5]	< 0.001	0.11
Low risk of bias	6.5 [0.1–20.3]	[0–100]	3	751	5.1 [3.5–7.4]	96.1 [91.7–98.2]	< 0.001	0.421
**Pregnant women**								
Overall	0.9 [0.6–1.1]	NA	1	5048	NA	NA	1	NA
Low risk of bias	0.9 [0.6–1.1]	NA	1	5048	NA	NA	1	NA
**Apparently healthy individuals**								
Overall	0.7 [0–12.2]	NA	2	131	1.9 [1–4]	71.9 [0–93.7]	0.059	NA
Cross-sectional	0 [0–1.5]	NA	1	114	NA	NA	1	NA
**Blood donors**								
Overall	0 [0–1.3]	NA	1	127	NA	NA	1	NA
Cross-sectional	0 [0–1.3]	NA	1	127	NA	NA	1	NA
Low risk of bias	0 [0–1.3]	NA	1	127	NA	NA	1	NA
**Recent contact**								
**LASV suspected cases**								
Overall	22.4 [4.4–48.6]	[0–100]	6	1398	9.7 [8.3–11.4]	98.9 [98.5–99.2]	< 0.001	0.621
Cross-sectional	19.3 [1.9–48.1]	[0–100]	5	1326	10.6 [9–12.6]	99.1 [98.8–99.4]	< 0.001	0.782
Low risk of bias	22.8 [0.4–62.7]	[0–100]	4	668	8.9 [7.2–11.1]	98.8 [98.1–99.2]	< 0.001	0.002
**Febrile patients**								
Overall	21.6 [4.6–46]	[0–100]	4	1600	8.5 [6.8–10.7]	98.6 [97.8–99.1]	< 0.001	0.446
Cross-sectional	10.1 [0–33.5]	NA	2	482	6.5 [4.2–10.2]	97.7 [94.3–99]	< 0.001	NA
Low risk of bias	30.4 [13.2–51]	NA	2	1332	6.1 [3.8–9.7]	97.3 [93.2–98.9]	< 0.001	NA
**Apparently healthy individuals**								
Overall	12.6 [4–24.8]	[0–64.7]	7	3452	8.9 [7.6–10.4]	98.7 [98.3–99.1]	< 0.001	0.752
Cross-sectional	12.8 [3.9–25.8]	[0–69.5]	6	3435	9.7 [8.3–11.4]	98.9 [98.5–99.2]	< 0.001	0.684
Low risk of bias	11.5 [0.4–34.3]	[0–100]	3	1946	12.6 [10.2–15.5]	99.4 [99–99.6]	< 0.001	0.295
**Healthcare workers**								
Overall	0 [0–0.7]	NA	2	564	1	0	0.823	NA
Cross-sectional	1.1 [0.4–2.2]	NA	1	552	NA	NA	1	NA
Low risk of bias	1.1 [0.4–2.2]	NA	1	552	NA	NA	1	NA
**Past contact**								
**Febrile patients**								
Overall	19.1 [10.8–29.1]	[0–60]	10	1441	4.2 [3.4–5.2]	94.3 [91.5–96.2]	< 0.001	0.486
Cross-sectional	19.9 [9.6–32.6]	[0–69.5]	7	1291	5.1 [4.1–6.3]	96.1 [93.9–97.5]	< 0.001	0.57
Low risk of bias	3.3 [0–13.4]	NA	2	326	1.9 [1–4.1]	73.2 [0–94]	0.053	NA
**LASV suspected cases**								
Overall	17.7 [3.1–40.6]	[0–98.6]	5	1798	10.6 [9–12.6]	99.1 [98.8–99.4]	< 0.001	0.554
Cross-sectional	17.7 [3.1–40.6]	[0–98.6]	5	1798	10.6 [9–12.6]	99.1 [98.8–99.4]	< 0.001	0.554
Low risk of bias	7 [0–25.8]	NA	2	762	7.3 [4.8–11.1]	98.1 [95.6–99.2]	< 0.001	NA
**Patient with any illness**								
Overall	16.9 [0–68]	[0–100]	3	135	5.8 [4.1–8.2]	97 [94.1–98.5]	< 0.001	< 0.001
Cross-sectional	16.9 [0–68]	[0–100]	3	135	5.8 [4.1–8.2]	97 [94.1–98.5]	< 0.001	< 0.001
**High risk individuals**								
Overall	8.6 [0.2–25.4]	[0–100]	3	500	4.8 [3.2–7.1]	95.6 [90.3–98]	< 0.001	0.78
Cross-sectional	8.6 [0.2–25.4]	[0–100]	3	500	4.8 [3.2–7.1]	95.6 [90.3–98]	< 0.001	0.78
Low risk of bias	26.1 [19.6–33.2]	NA	1	161	NA	NA	1	NA
**Healthcare workers**								
Overall	6.8 [4.2–10.1]	[0–23]	15	6355	4.2 [3.6–5]	94.4 [92.3–96]	< 0.001	0.922
Cross-sectional	8.3 [5.3–11.9]	[0.3–24.7]	11	5940	4.5 [3.7–5.4]	95 [92.7–96.5]	< 0.001	0.728
Low risk of bias	12.3 [9.7–15.2]	NA	1	552	NA	NA	1	NA
**LASV Positive case contact**								
Overall	4.2 [1.2–8.3]	[0–24.1]	4	295	1.3 [1–2.3]	44 [0–81.3]	0.147	0.998
**Apparently healthy individuals**								
Overall	3.8 [1.5–7]	[0–38.8]	48	38959	12.7 [12.2–13.3]	99.4 [99.3–99.4]	< 0.001	0.424
Cross-sectional	3.4 [1.2–6.5]	[0–37.4]	46	37989	12.7 [12.2–13.3]	99.4 [99.3–99.4]	< 0.001	0.377
Low risk of bias	7.5 [0.8–20]	[0–68.1]	6	5446	13.6 [12–15.5]	99.5 [99.3–99.6]	< 0.001	0.327
**Apparently healthy individuals, Patient with any illness**								
Overall	0.6 [0–1.7]	[0–9]	4	3164	2.4 [1.5–3.9]	83.1 [56.9–93.4]	< 0.001	0.466
Cross-sectional	0.3 [0–1.1]	[0–33.1]	3	2864	2.1 [1.2–3.9]	78.4 [30.4–93.3]	0.01	0.851
**Patient with illnesses other than fever diseases**								
Overall	0.3 [0–2.8]	[0–80.8]	3	660	1.9 [1–3.4]	70.8 [0.6–91.4]	0.032	0.717
Cross-sectional	0.3 [0–2.8]	[0–80.8]	3	660	1.9 [1–3.4]	70.8 [0.6–91.4]	0.032	0.717
Low risk of bias	0 [0–0.3]	NA	2	202	1	0	0.537	NA
**LASV prevalence in rodents**								
**Current contact**								
**Hylomyscus pamfi**								
Overall	41.7 [14.9–71]	NA	1	12	NA	NA	1	NA
Cross-sectional	41.7 [14.9–71]	NA	1	12	NA	NA	1	NA
**Mastomys species**								
Overall	12.8 [9.5–16.6]	[4.3–24.9]	7	939	1.6 [1–2.4]	59.3 [6.4–82.3]	0.022	0.124
Cross-sectional	12.8 [9.5–16.6]	[4.3–24.9]	7	939	1.6 [1–2.4]	59.3 [6.4–82.3]	0.022	0.124
**Mus baoulei**								
Overall	7 [0–39.1]	NA	2	29	2.1 [1–4.3]	76.5 [0–94.7]	0.039	NA
Cross-sectional	7 [0–39.1]	NA	2	29	2.1 [1–4.3]	76.5 [0–94.7]	0.039	NA
**Mastomys natalensis**								
Overall	5.5 [2.1–10.1]	[0–28.2]	10	4573	5.6 [4.7–6.6]	96.8 [95.4–97.7]	< 0.001	0.942
Cross-sectional	5.5 [2.1–10.1]	[0–28.2]	10	4573	5.6 [4.7–6.6]	96.8 [95.4–97.7]	< 0.001	0.942
**Mastomys erythroleucus**								
Overall	3 [0–10.3]	[0–42.4]	5	462	2.8 [1.9–4.1]	87.1 [72.3–94]	< 0.001	0.059
Cross-sectional	3 [0–10.3]	[0–42.4]	5	462	2.8 [1.9–4.1]	87.1 [72.3–94]	< 0.001	0.059
**Mus (Nannomys) species**								
Overall	1.2 [0–3.6]	NA	1	165	NA	NA	1	NA
Cross-sectional	1.2 [0–3.6]	NA	1	165	NA	NA	1	NA
*Negative Rodents								
Overall	0 [0–0]	[0–0]	27	1316	1 [1–1]	0 [0–0]	1	< 0.001
Cross-sectional	0 [0–0]	[0–0]	27	1316	1 [1–1]	0 [0–0]	1	< 0.001
**Unspecified rodents**								
Overall	4.1 [1.5–7.8]	[0–28.1]	21	4826	4.7 [4.1–5.3]	95.4 [94.1–96.5]	< 0.001	0.969
Cross-sectional	4.2 [1.4–8]	[0–28.7]	20	4760	4.8 [4.2–5.5]	95.7 [94.4–96.7]	< 0.001	0.976
**Past contact**								
**Mus baoulei**								
Overall	33.3 [11.3–59.5]	NA	1	15	NA	NA	1	NA
Cross-sectional	33.3 [11.3–59.5]	NA	1	15	NA	NA	1	NA
**Mastomys natalensis**								
Overall	12.8 [6.2–21.3]	[0–100]	3	1380	4.2 [2.7–6.4]	94.3 [86.8–97.6]	< 0.001	0.979
Cross-sectional	12.8 [6.2–21.3]	[0–100]	3	1380	4.2 [2.7–6.4]	94.3 [86.8–97.6]	< 0.001	0.979
**Lemniscomys striatus**								
Overall	12.5 [2.9–26.6]	NA	1	32	NA	NA	1	NA
Cross-sectional	12.5 [2.9–26.6]	NA	1	32	NA	NA	1	NA
**Mastomys erythroleucus**								
Overall	7.2 [0.2–21.5]	NA	2	367	3.8 [2.1–6.9]	93 [76.7–97.9]	< 0.001	NA
Cross-sectional	7.2 [0.2–21.5]	NA	2	367	3.8 [2.1–6.9]	93 [76.7–97.9]	< 0.001	NA
**Mus minutoides**								
Overall	6.6 [1.1–15]	NA	2	59	1	0	0.872	NA
Cross-sectional	6.6 [1.1–15]	NA	2	59	1	0	0.872	NA
**Praomys daltoni**								
Overall	3.3 [1.5–5.7]	NA	2	299	1	0	0.818	NA
Cross-sectional	3.3 [1.5–5.7]	NA	2	299	1	0	0.818	NA
**Rattus rattus**								
Overall	2.2 [0–20]	NA	2	92	2.3 [1.1–4.8]	81.7 [22.3–95.7]	0.02	NA
Cross-sectional	2.2 [0–20]	NA	2	92	2.3 [1.1–4.8]	81.7 [22.3–95.7]	0.02	NA
**Praomys rostratus**								
Overall	1.2 [0–3.7]	NA	1	163	NA	NA	1	NA
Cross-sectional	1.2 [0–3.7]	NA	1	163	NA	NA	1	NA
**#Negative rondents**								
Overall	0 [0–0.3]	[0–0.5]	10	279	1 [1–1]	0 [0–0]	0.999	< 0.001
Cross-sectional	0 [0–0.3]	[0–0.5]	10	279	1 [1–1]	0 [0–0]	0.999	< 0.001
**Unspecified rodents**								
Overall	4.4 [1.1–9.5]	[0–39]	18	6883	7.9 [7.2–8.8]	98.4 [98.1–98.7]	< 0.001	0.266
Cross-sectional	4.4 [1.1–9.5]	[0–39]	18	6883	7.9 [7.2–8.8]	98.4 [98.1–98.7]	< 0.001	0.266
**Other Mammals**								
**Current contact**								
**Primates**								
Overall	1.6 [0–6.8]	NA	1	62	NA	NA	1	NA
Cross-sectional	1.6 [0–6.8]	NA	1	62	NA	NA	1	NA
**Chiroptera**								
Overall	0 [0–2.3]	NA	1	75	NA	NA	1	NA
Cross-sectional	0 [0–2.3]	NA	1	75	NA	NA	1	NA
**Eulipotyphla**								
Overall	0 [0–0.3]	NA	2	223	1	0	0.542	NA
Cross-sectional	0 [0–0.3]	NA	2	223	1	0	0.542	NA
**Recent contact**								
**Primates**								
Overall	0 [0–2.8]	NA	1	62	NA	NA	1	NA
Cross-sectional	0 [0–2.8]	NA	1	62	NA	NA	1	NA
**Past contact**								
**Artiodactyla**								
Overall	0 [0–0.8]	[0–26.3]	3	172	1 [1–1.2]	0 [0–24.6]	0.871	0.001
Cross-sectional	0 [0–0.8]	[0–26.3]	3	172	1 [1–1.2]	0 [0–24.6]	0.871	0.001
**Carnivora**								
Overall	10.1 [2.8–20.9]	NA	2	204	2 [1–4.2]	75.2 [0–94.4]	0.045	NA
Cross-sectional	10.1 [2.8–20.9]	NA	2	204	2 [1–4.2]	75.2 [0–94.4]	0.045	NA
**Rodentia**								
Overall	0 [0–2.8]	NA	1	60	NA	NA	1	NA
Cross-sectional	0 [0–2.8]	NA	1	60	NA	NA	1	NA
**Primates**								
Overall	1.1 [0–8.9]	[0–100]	3	560	2.9 [1.7–4.9]	88.2 [67.1–95.8]	< 0.001	0.478
Cross-sectional	1.1 [0–8.9]	[0–100]	3	560	2.9 [1.7–4.9]	88.2 [67.1–95.8]	< 0.001	0.478
**Chiroptera**								
Overall	0 [0–6.1]	NA	1	28	NA	NA	1	NA
Cross-sectional	0 [0–6.1]	NA	1	28	NA	NA	1	NA
**Eulipotyphla**								
Overall	0.5 [0–2.1]	NA	1	199	NA	NA	1	NA
Cross-sectional	0.5 [0–2.1]	NA	1	199	NA	NA	1	NA

CI: confidence interval; N: Number; 95% CI: 95% Confidence Interval; NA: not applicable.

¶H estimates the extent of heterogeneity, a value of H = 1 indicates lack of evidence on heterogeneity of effects and a value of H >1indicates a potential heterogeneity of effects.

§: I^2^ describes the proportion of total variation in study estimates that is due to heterogeneity, a value > 50% indicates presence of heterogeneity. All these estimates were obtained using random effect meta-analysis

### Subgroup analysis and metaregression

**Fig 6:**
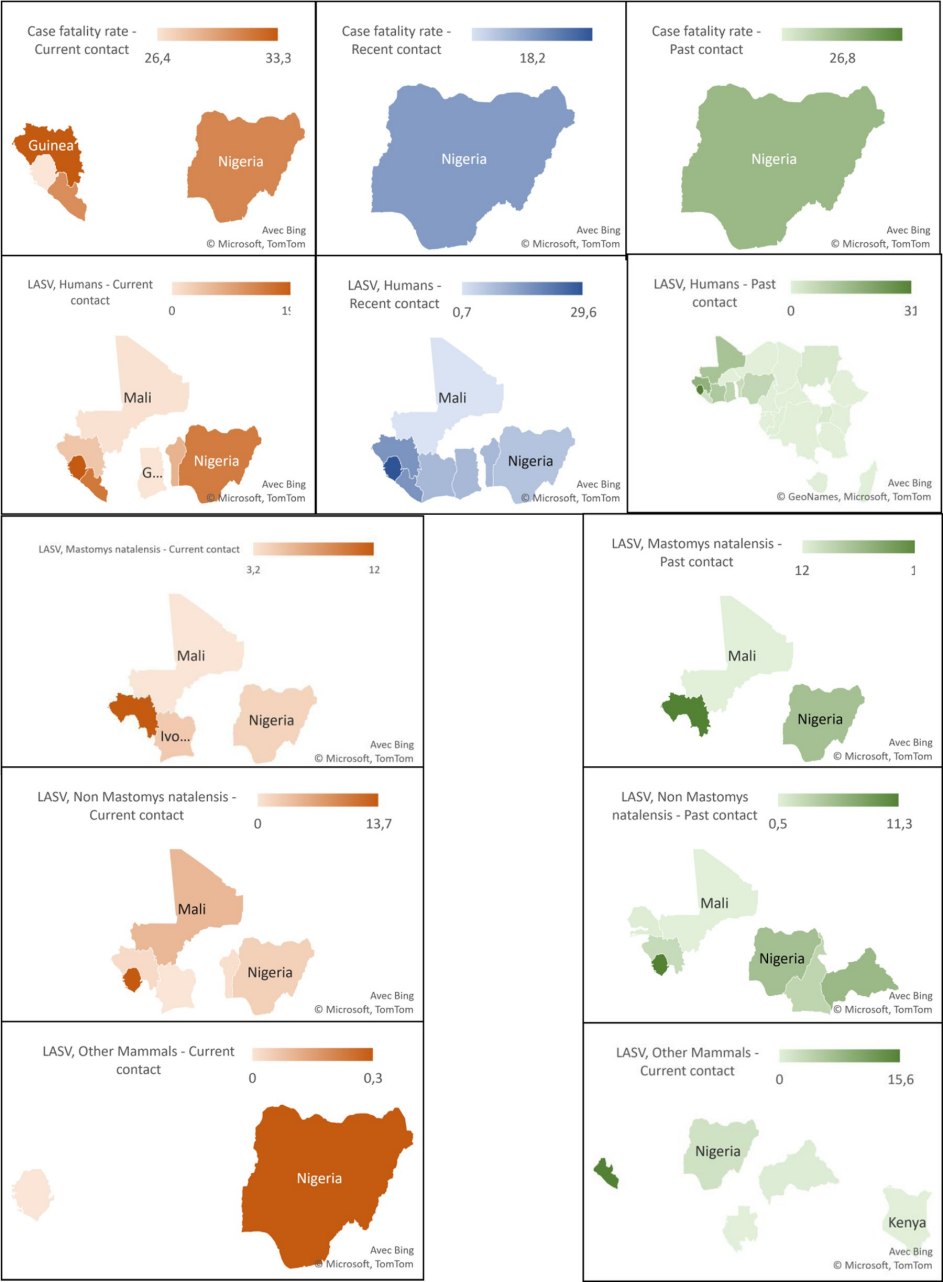
Lassa virus case fatality rate in humans and Lassa virus prevalence in humans, rodents, and other mammals in sub-Saharan Africa, 1970–2020. LASV: Lassa virus prevalence; Past contacts denote articles with unspecified antibodies or IgG positive subjects. Recent contacts denote articles with IgM or IgM and IgG positive subjects. Current contacts denote studies with antigen, RNA, virus positives or subjects with an increase in antibody titer between acute and convalescent samples. Current, recent and past contacts are indicated by shades of red, blue and green respectively. Lines 1, 2, 3, 4 and 5 indicate the LASV case fatality rate in humans, the prevalence of LASV in humans, *Mastomys natalensis*, non-*Mastomys natalensis* rodents and other mammals respectively.

All LASV deaths have been recorded only in West Africa including Nigeria, Guinea, and Sierra Leone (Fig 6) [34,37,47,53,65,70,120,123]. Only the case fatality rate category in LASV suspected cases fulfilled the conditions for subgroup analyses (at least 3 studies divided into two or more categories). The case fatality rate was significantly higher in studies on community outbreak (p = 0.036), with a retrospective design (p = 0.018) [47,53,89,101,116,117], conducted in rural areas (p <0.001) [53], and in the community (p = 0.039) (S8 Table) [47]. S9 Table displayed the results of the meta-regression analysis. The final model of the case fatality rate in current LASV infection suspected cases explained 32.5% of the heterogeneity. In this model only retrospective studies presented significantly high case fatality rates (p = 0.002).

The LASV prevalence in current infections in humans was statistically higher in hospitalized febrile patients (p = 0.001), in febrile patients in Liberia and Sierra Leone (p <0.001), in febrile patients in urban areas (p = 0.049) and in LASV suspected cases in rural areas (p = 0.048), and in case-control studies for LASV suspected cases (p <0.001). In febrile patients, the final meta-regression model explained 0% heterogeneity for the prevalence of the current LASV infection. Case control and hospital/community-based studies showed the highest prevalence.

The LASV seroprevalence in recent infections in humans was statistically higher in Sierra Leone in apparently healthy individuals (p <0.001) and in LASV suspected cases (p <0.001), in urban areas in apparently healthy individuals (p = 0.015), and in case-control studies in febrile patients (p = 0.036). In apparently healthy individuals, the final meta-regression model explained 60.3% heterogeneity for the seroprevalence of recent LASV infection and Sierra Leone had the highest prevalence.

The seroprevalence of LASV in past infections in humans was statistically higher in West African countries including Guinea, Sierra Leone, Nigeria, Mali, Ivory Coast, in context urban, especially in apparently healthy individuals/ patients with any disease and LASV suspected cases. In apparently healthy individuals, the final meta-regression model explained 75.9% heterogeneity for the seroprevalence of past LASV infection. West Africa and hospital/community-based studies showed the highest prevalences. In febrile patients, the final meta-regression model explained 54.1% heterogeneity for the seroprevalence of past LASV infection. Non-probabilistic and West Africa studies showed the highest prevalences. In LASV suspected cases, the final meta-regression model explained 99.0% heterogeneity for the seroprevalence of past LASV infection and studies conducted in urban setting showed the highest prevalences. In patients with any disease, the final meta-regression model explained 100% heterogeneity for the seroprevalence of past LASV infection and studies conducted in hospitals showed the highest prevalences. In patients with diseases other than fever, the final meta-regression model explained 100% heterogeneity for the seroprevalence of past LASV infection and studies conducted in West Africa showed the highest prevalences.

The LASV prevalence in current infections in Mastomys species was statistically higher in West African countries (p <0.001) including Guinea and Sierra Leone and in spleen samples (p = 0.004). In the Mastomys species, the final meta-regression model explained 100% heterogeneity for the prevalence of the current LASV infection and the studies conducted in Sierra Leone presented the highest prevalences.

The LASV seroprevalence in past infections in Mastomys natalensis was statistically higher in Nigeria (p = 0.001). In Mastomys natalensis, the final meta-regression model explained 82.0% heterogeneity for the prevalence of the current LASV infection and the studies conducted in Guinea showed the highest prevalences.

## Discussion

Our study presents the most comprehensive records to date on the LASV human case fatality rate and LASV prevalence in humans, rodents, and other mammals. We estimated an overall LASV case fatality rate of around 30% and a LASV prevalence around 10%, 3%, and 1% in humans, rodents, and other mammals respectively. The prevalence of LASV infections was higher in West African region, in LASV suspect cases, febrile patients, patients with haemorrhagic symptoms, healthcare workers, apparently healthy patients, and patient with any diseases. In addition to *Mastomys natalensis*, the main reservoir of LASV [137], this study clarifies the LASV current contact estimated mainly by RT-PCR and culture assays in *Hylomyscus pamfi*, *Mus baoulei*, *Mastomys erythroleucus*, and *Mus (Nannomys) species*. In the present work, the primates have shown evidence of LASV antigen and antibodies. Carnivora and Eulipotyphla have shown evidence of past contact with LASV through antibody detection.

This relatively high overall case fatality rate of around 30% due to LASV infections is surprising in this era of availability of antivirals such as ribavirin which have been shown to be effective in the early phase of LASV infections in individuals with of elevated aspartate aminotransferase [138]. The case fatality rate of 27.1% observed in pregnant women in this study is comparable to that of a recent meta-analysis which also included this same population [139]. This meta-analysis had however included studies with pregnant women with suspected or probable LASV infections [140,141]. Patients who died with past LASV infections presented in this work may be those IgM and/or IgG positive in the early stage of infection rather than sequelae of mortality from an old infection. Indeed, it has been shown that IgM is produced from the 4th day and rarely persists beyond one month in LASV infections [142–144]. Immunoglobulin G appear around the 8th day after the onset of symptoms and persist longer. Although reported in only two studies, compared to other subgroups, it was surprising to record a relatively low case fatality rate of 13,2% among participants with haemorrhagic symptoms, since bleeding is known as a predictor of bad outcomes in viral haemorrhagic symptoms [145]. This could be explained by several factors including the management and the treatment delay implemented in these studies with patients with haemorrhagic symptoms.

The various stakeholders in human and animal health should make concerted efforts to fight LASV. This fight should not be limited to healthcare professionals, but should also include funding agencies, politicians, citizens and all relevant stakeholders. This study shows that studies using direct methods of detecting LASV have been conducted only in West Africa. Thus, additional research using direct detection assays for current LASV infections are needed outside West Africa where past and recent LASV infections have been reported. On human side, Lassa fever should be taken into account in the differential diagnosis of febrile illnesses across West Africa in addition to the common febrile illnesses such as malaria, typhoid fever and dengue. This study highlights the need to educate people in SSA about the signs and symptoms of Lassa fever and to keep dwellings safe from rodents by creating adequate food storage spaces. All LASV infections in healthcare workers could have been reduced or avoided through actions such as 1) educating healthcare workers about Lassa fever guidelines and the risk of infection during their activity, 2) strengthening the use of personal protective equipment; and 3) training in the adequate use of personal protective equipment. Findings from this study point out the need to establish LASV laboratory-based surveillance programs across West Africa. The construction of well-equipped laboratories and research centre would help to quickly diagnose and treat Lassa fever. To this end, the development of a simple rapid diagnostic test would be of great added value for the diagnosis of patients. Additional research on viral factors (viral load, genotype), biomarkers, clinical and socio-demographic factors associated with mortality from the LASV is necessary. We identified a single study with LASV prevalence record among pregnant women. This suggests an urgent need to provide more data on the proportion of pregnant women with LASV infections. This study clearly demonstrates that LASV is a highly lethal pathogen, so the paradox of infections recorded in apparently asymptomatic individuals remains to be elucidated in particular their role in the transmission chain of LASV infection.

The results of this study suggest that apart from *Mastomys natalensis*, other rodent species may be involved in the LASV transmission. Most IgG-positive results detected in small mammals outside the LASV-endemic zone beyond West Africa during this study are probably signatures of the several arenaviruses other than Lassa that continue to be described across sub Saharan Africa of late. Nevertheless, these results are equally crucial for portrayal in a manuscript like this; as these arenaviruses are related by varying degrees to LASV and their epidemiological significance needs to be further understood. An effort should be directed towards the sequencing and phylogenetic characterization of the LASV strains identified. The promotion of research on the development of vaccines in rodents or other mammals sensitive to LASV which can subsequently be adapted for human vaccination is necessary. There should be a recognition of LASV surveillance in rodents and other mammals to better control the risk of human infection.

Most of the included studies used the Josiah strain as an antigen for the detection of antibodies in serological methods. Due to the great variability of LASV, however, it may have antibodies that do not recognize the Josiah antigen. This suggests the possibility of having false negative results in some included studies. There is a high probability of cross-reactions between Arenavirus species [146,147]. Although plaque reduction neutralisation test which is the most specific assay in serological detection has been used to assess result in 19 included studies, the potential cross reactions associated with other serological methods suggests the possibility of false positive results in some studies included. Another important weakness to highlight in this study is the absence or variability in the case definitions of LASV infections in the included studies.

These results regarding the vector role of LASV for non-*Mastomys natalensis* rodents should be interpreted with caution, because these infections could rather reflect a spillover infection or a misclassification of rodents due to the same number of chromosomes in certain species [7]. It should also be noted that all of the studies on current LASV detected in non-Mastomys rodents have used either RT-PCR, culture or Antigen detection by ELISA. Regarding the detection of RNA by RT-PCR, none of these studies sequenced the viruses detected and could therefore be subject to false positive results [146,147]. The only study that isolated LASV by culture raised the possibility of an incorrect classification of rodent species [124]. Antibodies suggesting past contacts with LASV in *Mastomys natalensis* and non-*Mastomys natalensis* rodents have been reported in the present study. These antibodies could, however, be acquired transplacentally or by lactation in young rodents [148]. The ages of trapped rodents were unfortunately not reported in most included studies.

### Conclusions

Despite its limitations, this systematic review provides a comprehensive overview of the case fatality rate due to LASV in humans and the prevalence of LASV in humans, rodents and other mammals. We carried out a categorization of meta-analyses according to the different categories of population and target of the LASV sought. Meta-analyses suggest a relatively high case fatality rate and a prevalence of LASV infection influenced by geographic locations. The results of the study suggest that LASV is widespread in West Africa. It also appears that apart from *Mastomys natalensis*, other rodents could serve as reservoirs for LASV.

## Supporting information

S1 Table Preferred reporting items for systematic reviews and meta-analyses checklist. (PDF)

S2 Table Search strategy in Medline (Pubmed). (PDF)

S3 Table Items for risk of bias assessment. (PDF)

S4 Table Main reasons of exclusion of eligible studies. (PDF)

S5 Table Risk of bias assessment. (PDF)

S6 Table Characteristics of included studies. (PDF)

S7 Table Individual characteristics of included studies. (PDF)

S8 Table Subgroup of human case fatality rate and prevalence of Lassa virus in humans, rodents, and other animals in sub-Saharan Africa. (PDF)

S9 Table Univariable and multivariable meta‐regression analysis on the human case fatality rate and prevalence of Lassa virus in humans, rodents, and other animals. (PDF)

S1 Fig Prevalence of Lassa virus infections in humans in sub-Saharan Africa. (PDF)

S2 Fig Prevalence of Lassa virus infections in rodents in sub-Saharan Africa. (PDF)

S3 Fig Funnel plot for publication for Lassa virus case fatality rate in humans. (PDF)

S4 Fig Funnel plot for publication for Lassa virus prevalence in humans. (PDF)

S5 Fig Funnel plot for publication for Lassa virus prevalence in rodents. (PDF)

S6 Fig Funnel plot for publication for Lassa virus prevalence in other mammals. (PDF)
