# Supplementary material for: Systematic review and meta-analysis of the epidemiology of Lassa virus in humans, rodents and other mammals in sub-Saharan Africa
Source: PLoS Negl Trop Dis. 2020 Aug 26;14(8):e0008589. doi: 10.1371/journal.pntd.0008589 (PMC7478710; doi:10.1371/journal.pntd.0008589)
Supplement: S2 Table — (PDF) [file pntd.0008589.s002.pdf]

S2 Table: Search strategy in Medline (Pubmed)

Search	Virus
#1	Lassa OR Lassa Virus OR LASV OR Lassa fever virus OR Lassa Fever OR Lassa hemorrhagic fever OR Lassa hemorrhagic fever virus OR Lassa infection OR Lassa virus disease
#2	sub Saharan Africa OR Central Africa OR West Africa OR Western Africa OR East Africa OR Eastern African OR South African OR Southern African OR Angola OR Benin OR Botswana OR Burkina Faso OR Burundi OR Cameroon OR Canary Islands OR Cape Verde OR Central African Republic OR Chad OR Comoros OR Congo OR Democratic Republic of Congo OR Djibouti OR Equatorial Guinea OR Eritrea OR Ethiopia OR Gabon OR Gambia OR Ghana OR Guinea OR Guinea Bissau OR Ivory Coast OR Cote d'Ivoire OR Kenya OR Lesotho OR Liberia OR Madagascar OR Malawi OR Mali OR Mauritania OR Mauritius OR Mayotte OR Mozambique OR Namibia OR Niger OR Nigeria OR Principe OR Reunion OR Rwanda OR Sao Tome OR Senegal OR Seychelles OR Sierra Leone OR Somalia OR South Africa OR South Sudan OR St Helena OR Sudan OR Swaziland OR Tanzania OR Togo OR Uganda OR Western Sahara OR Zaire OR Zambia OR Zimbabwe
#3	(#1 AND #2)
