# Supplementary material for: Systematic review and meta-analysis of the epidemiology of Lassa virus in humans, rodents and other mammals in sub-Saharan Africa
Source: PLoS Negl Trop Dis. 2020 Aug 26;14(8):e0008589. doi: 10.1371/journal.pntd.0008589 (PMC7478710; doi:10.1371/journal.pntd.0008589)
Supplement: S3 Table — (PDF) [file pntd.0008589.s003.pdf]

S3 Table: Items for risk of bias assessment

**Hoy et al. tool for cross sectional studies**	**Yes (1)/No (0)**
**External validity**	
1. Was the study's target population a close representation of the national population in relation to Lassa virus prevalence or case fatality rate?	**1**
2. Was the sampling frame a true or close representation of the study population?	**1**
3. Was some form of random selection used to select the sample, OR was a census undertaken?	**1**
4. Was the likelihood of nonresponse bias minimal (> 70%)?	**1**
**Internal validity**	**1**
5. Were data collected directly from the subjects (as opposed to a proxy)?	**1**
6. Was an acceptable case definition used in the study?	**1**
7. Was the study viral detection assay shown to have validity and reliability?	**1**
8. Was the same mode type of sample collected for all subjects?	**1**
9. Was the length of the length of the study period > 1 year?	**1**
10. Were the numerator(s) and denominator(s) for the Lassa virus prevalence or case fatality rate appropriate?	**1**
Total score	**10**
**Interpretation of the risk of bias tool**<br><br>• 7-10: Low risk of bias<br>• 4-6: Moderate risk of bias<br>• 0-3: High risk of bias	
