# Supplementary material for: Systematic review and meta-analysis of the epidemiology of Lassa virus in humans, rodents and other mammals in sub-Saharan Africa
Source: PLoS Negl Trop Dis. 2020 Aug 26;14(8):e0008589. doi: 10.1371/journal.pntd.0008589 (PMC7478710; doi:10.1371/journal.pntd.0008589)
Supplement: S4 Table — (PDF) [file pntd.0008589.s004.pdf]

S4 Table: Main reasons of exclusion of eligible studies

N°	Author. Year	Title	Reason of exclusion
1	Abdulraheem. 2012	Public health importance of lassa fever epidemiology. clinical features and current management review of literature	Review
2	Adogo. 2020	Yellow fever in Nigeria: a review of the current situation	Review
3	Adomeh. 2012	The Sensitivity and Specificity of Lassa Virus IgM by ELISA as Screening Tool at Early Phase of Lassa Fever Infection.	Sample with already known result
4	Aiko Fukuma. 2012	Rapid detection of Lassa virus by reverse transcription-loop-mediated isothermal amplification.	No data on LASV prevalence or case fatality rate
5	Ajayi. 2013	Containing a Lassa fever epidemic in a resource-limited setting: outbreak description and lessons learned from Abakaliki. Nigeria (January-March 2012).	No data on LASV prevalence or case fatality rate
6	Akhiwu. 2018	Lassa fever outbreak in adolescents in North Central Nigeria: report of cases.	Case report
7	Akpede. 2019	Acute Abdomen in Pediatric Patients With Lassa Fever: Prevalence and Response to Nonoperative Management.	No data on LASV prevalence or case fatality rate
8	Akpede. 2019	Caseload and Case Fatality of Lassa Fever in Nigeria. 2001-2018: A Specialist Center's Experience and Its Implications.	Review
9	Akpede. 2018	Lassa fever outbreaks in Nigeria.	Case report
10	Azeez-Akande. 2016	A REVIEW OF LASSA FEVER. AN EMERGING OLD WORLD HAEMORRHAGIC VIRAL DISEASE IN SUB-SAHARAN AFRICA	Review
11	Bausch. 2000	Diagnosis and clinical virology of Lassa fever as evaluated by enzyme-linked immunosorbent assay. indirect fluorescent-antibody test. and virus isolation.	Study with serial samples from the same patient
12	Bergmann. 1982	New African viral fevers Ebola. Lassa and Marburg.	Review
13	Bloch. 1978	A serological survey of Lassa fever in Liberia.	Duplicate study
14	Boiro. 1987	Experimental studies of haemorrhagic fever in Guinea (Clinical. epidemiological and serological investigations).	Duplicate study
15	Boisen. 2016	Field Validation of the ReEBOV Antigen Rapid Test for Point-of-Care Diagnosis of Ebola Virus Infection.	No data on LASV prevalence or case fatality rate

16	Boisen. 2015	Multiple circulating infections can mimic the early stages of viral hemorrhagic fevers and possible human exposure to filoviruses in sierra leone prior to the 2014 outbreak.	No data on LASV prevalence or case fatality rate
17	Bonney. 2018	Molecular detection of dengue virus in patients suspected of Ebola virus disease in Ghana.	No data on LASV prevalence or case fatality rate
18	Bonney . 2016	Molecular confirmation of Lassa fever imported into Ghana.	Sample size < or = 10 participants
19	Borremans . 2011	Presence of Mopeia virus. an African arenavirus. related to biotope and individual rodent host characteristics: implications for virus transmission.	No data on LASV prevalence or case fatality rate
20	Bowen . 2000	Genetic diversity among Lassa virus strains.	No data on LASV prevalence or case fatality rate
21	Chare. 2003	Phylogenetic analysis reveals a low rate of homologous recombination in negative-sense RNA viruses.	No data on LASV prevalence or case fatality rate
22	Cummins. 1989	Electrocardiographic abnormalities in patients with Lassa fever.	No data on LASV prevalence or case fatality rate
23	Cummins. 1989	A plasma inhibitor of platelet aggregation in patients with Lassa fever.	No data on LASV prevalence or case fatality rate
24	Dedkov. 2019	Development and Evaluation of a One-Step Quantitative RT-PCR Assay for Detection of Lassa Virus.	Sample with already known result
25	Dongo. 2013	Lassa fever presenting as acute abdomen: a case series.	Case report
26	Drosten. 2002	Rapid detection and quantification of RNA of Ebola and Marburg viruses. Lassa virus. Crimean-Congo hemorrhagic fever virus. Rift Valley fever virus. dengue virus. and yellow fever virus by real-time reverse transcription-PCR.	Report
27	Duvignaud. 2019	Delayed-onset paraparesis in Lassa fever: A case report.	Case report
28	Dyer. 2019	Lassa outbreak: WHO warns of unusually rapid spread in Nigeria.	Review
29	Dzotsi. 2012	The first cases of Lassa fever in Ghana.	Case report
30	Ehichioya. 2019	Phylogeography of Lassa Virus in Nigeria.	No data on LASV prevalence or case fatality rate
31	Ehichioya . 2011	Current molecular epidemiology of Lassa virus in Nigeria.	No data on LASV prevalence or case fatality rate
32	Ehichioya . 2010	Lassa fever. Nigeria. 2005-2008.	Report

33	Eze. 2014	Acute abdominal pain in patients with lassa fever: Radiological assessment and diagnostic challenges.	Review
34	Eze. 2010	High Lassa Fever activity in Northern part of Edo State. Nigeria: reanalysis of confirmatory test results	Duplicate study
35	Fatiregun. 2019	Lassa fever awareness and knowledge among community residents in Ondo State. Nigeria	No data on LASV prevalence or case fatality rate
36	Fichet-Calvet. 2016	Spatial and temporal evolution of Lassa virus in the natural host population in Upper Guinea.	No data on LASV prevalence or case fatality rate
37	Fichet-Calvet. 2013	Lassa Fever: A rodent-human interaction.	Report
38	Fichet-Calvet. 2008	Reproductive characteristics of Mastomys natalensis and Lassa virus prevalence in Guinea. West Africa.	No data on LASV prevalence or case fatality rate
39	Fichet-calvet. 2005	Spatial distribution of commensal rodents in regions with high and low Lassa fever prevalence in Guinea.	No data on LASV prevalence or case fatality rate
40	Frame. 1984	The use of Lassa fever convalescent plasma in Nigeria.	No data on LASV prevalence or case fatality rate
41	Frame. 1975	Surveillance of Lassa fever in missionaries stationed in West Africa.	Study with serial samples from the same patient
42	Fraser. 1974	Lassa fever in the Eastern Province of Sierra Leone. 1970-1972. I. Epidemiologic studies.	Not possible to extract data on LASV prevalence or case fatality rate
43	FYUMAGWA. 2011	Response to Rift Valley Fever in Tanzania: Challenges and Opportunities	Review
44	Grundy. 1980	Isolated case of Lassa fever in Zaria. Northern Nigeria.	Case report
45	Günther. 2001	Antibodies to Lassa virus Z protein and nucleoprotein co-occur in human sera from Lassa fever endemic regions.	No data on LASV prevalence or case fatality rate
46	Ibekwe. 2012	The sensitivity and specificity of Lassa virus IgM by ELISA as screening tool at early phase of Lassa fever infection.	Sample with already known result
47	Ibekwe. 2010	Early-onset sensorineural hearing loss in Lassa fever.	No data on LASV prevalence or case fatality rate
48	Ilori. 2019	Increase in Lassa Fever Cases in Nigeria. January-March 2018.	No data on LASV prevalence or case fatality rate
49	Ilori. 2019	Epidemiologic and Clinical Features of Lassa Fever Outbreak in Nigeria. January 1-May 6. 2018.	Duplicate study
50	Iroezindu. 2015	Lessons learnt from the management of a case of Lassa fever and follow-up of nosocomial primary contacts in	Case report

		Nigeria during Ebola virus disease outbreak in West Africa.	
51	Ishak. 1982	Viral hemorrhagic fevers with hepatic involvement: pathologic aspects with clinical correlations.	Review
52	Jones. 2000	Lassa fever imported to England.	Case report
53	Kafetzopoulou. 2019	Metagenomic sequencing at the epicenter of the Nigeria 2018 Lassa fever outbreak.	No data on LASV prevalence or case fatality rate
54	Keïta . 2019	Investigation of a cross-border case of Lassa fever in West Africa.	Case report
55	Knobloch. 1980	Clinical observations in 42 patients with lassa fever.	No full text
56	Koehler. 2018	A highly multiplexed broad pathogen detection assay for infectious disease diagnostics.	No data on LASV prevalence or case fatality rate
57	Manning. 2015	Lassa virus isolates from Mali and the Ivory Coast represent an emerging fifth lineage.	No data on LASV prevalence or case fatality rate
58	Martin Gabriel. 2018	Development and evaluation of antibody-capture immunoassays for detection of Lassa virus nucleoprotein-specific immunoglobulin M and G.	Sample with already known result
59	Mateo. 2019	Fatal Case of Lassa Fever. Bangolo District. Côte d'Ivoire. 2015.	Case report
60	Maxmen. 2018	Deadly Lassa-fever outbreak tests Nigeria's revamped health agency.	Report
61	Mertens. 1973	Clinical presentation of Lassa fever cases during the hospital epidemic at Zorzor. Liberia. March-April 1972.	Case report
62	Monath. 1974	Lassa fever in the Eastern Province of Sierra Leone. 1970-1972. II. Clinical observations and virological studies on selected hospital cases.	No data on LASV prevalence or case fatality rate
63	Monson. 1987	Pediatric Lassa fever: a review of 33 Liberian cases.	Not possible to extract data on LASV prevalence or case fatality rate
64	Monson. 1984	Endemic Lassa fever in Liberia. I. Clinical and epidemiological aspects at Curran Lutheran Hospital. Zorzor. Liberia.	No data on LASV prevalence or case fatality rate
65	Niklasson. 1984	Detection of Lassa virus antigens and Lassa virus-specific immunoglobulins G and M by enzyme-linked immunosorbent assay.	No data on LASV prevalence or case fatality rate
66	No author listed. 2014	After the “blood diamond” conflict: lassa fever in Sierra Leone.	Comment on an article

67	No author listed. 2000	Large Ebola haemorrhagic fever outbreak in Uganda.	No full text
68	No authorlisted. 1974	Rodent control and lassa fever.	Review
69	No authors listed. 2000	Lassa fever. imported case. Netherlands.	Case report
70	Okuno. 1976	Failure to prove arenavirus infection among the small mammals from an endemic area of Korean hemorrhagic nephrosonephritis.	Data outside of sub Saharan Africa
71	Olowookere. 2014	Diagnostic proficiency and reporting of Lassa fever by physicians in Osun State of Nigeria.	No data on LASV prevalence or case fatality rate
72	Olschläger. 2010	Improved detection of Lassa virus by reverse transcription-PCR targeting the 5' region of S RNA.	No data on LASV prevalence or case fatality rate
73	Olugasa. 2015	Mapping of Lassa fever cases in post-conflict Liberia. 2008-2012: a descriptive and categorical analysis of age. gender and seasonal pattern.	Report
74	Onyedibe. 2018	A cross sectional study of dengue virus infection in febrile patients presumptively diagnosed of malaria in Maiduguri and Jos plateau. Nigeria	No data on LASV prevalence or case fatality rate
75	Richards. 2015	Viral haemorrhagic fevers in South Africa	Review
76	Roberts. 2018	Nigeria hit by unprecedented Lassa fever outbreak.	Report
77	Roth. 2015	Factors associated with mortality in febrile patients in a government referral hospital in the Kenema district of Sierra Leone.	No data on LASV prevalence or case fatality rate
78	Safronetz. 2017	Annual Incidence of Lassa Virus Infection in Southern Mali.	Sample with already known result
79	Salu. 2016	Biosafety level-2 laboratory diagnosis of Zaire Ebola virus disease imported from Liberia to Nigeria.	Case report
80	Satterly. 2017	Comparison of MagPix assays and enzyme-linked immunosorbent assay for detection of hemorrhagic fever viruses.	No data on LASV prevalence or case fatality rate
81	Sebba. 2018	A point-of-care diagnostic for differentiating Ebola from endemic febrile diseases.	No data on LASV prevalence or case fatality rate
82	Shaffer. 2019	Data set on Lassa fever in post-conflict Sierra Leone.	Duplicate study
83	Shaffer. 2014	Lassa Fever in Post-Conflict Sierra Leone.	Duplicate study
84	Siddle. 2018	Genomic Analysis of Lassa Virus during an Increase in Cases in Nigeria in 2018.	Not possible to extract data on LASV prevalence or case fatality rate

85	Singh. 2013	Arenavirus and West Nile virus in solid organ transplantation.	Review
86	Stremlau. 2015	Discovery of novel rhabdoviruses in the blood of healthy individuals from West Africa.	No data on LASV prevalence or case fatality rate
87	Tambo. 2018	Re-emerging Lassa fever outbreaks in Nigeria: Re-enforcing	Not possible to extract data on LASV prevalence or case fatality rate
88	Ter Meulen. 2001	Short communication: Lassa fever in Sierra Leone: UN peacekeepers are at risk.	No data on LASV prevalence or case fatality rate
89	Trappier. 1993	Evaluation of the polymerase chain reaction for diagnosis of Lassa virus infection.	Study with serial samples from the same patient
90	Usifoh. 2019	Lassa Fever-associated Stigmatization among Staff and Students of the University of Benin. Nigeria.	No data on LASV prevalence or case fatality rate
91	Usifoh. 2018	Impact of Lassa Fever on the Practice and Consumption of Stored Food by University of Benin Community. in Benin City. Nigeria	No data on LASV prevalence or case fatality rate
92	Vieth. 2007	RT-PCR assay for detection of Lassa virus and related Old World arenaviruses targeting the L gene.	No data on LASV prevalence or case fatality rate
93	White. 1972	Lassa fever A study of 23 hospital cases.	Not possible to extract data on LASV prevalence or case fatality rate
94	Whitmer. 2016	New Lineage of Lassa Virus. Togo. 2016.	No data on LASV prevalence or case fatality rate
95	Wolff. 2019	Lassa virus circulating in Liberia: a retrospective genomic characterisation.	No data on LASV prevalence or case fatality rate
96	Wolff. 2016	Genome sequence of Lassa virus isolated from the first domestically acquired case in Germany.	Data outside of sub Saharan Africa
97	World Health Organization. 2005	Update on Lassa fever in West Africa.	Report

Reference

1. *Rodent control and lassa fever.* Lancet, 1974. **2**(7881): p. 632.
2. *Large Ebola haemorrhagic fever outbreak in Uganda.* New Zealand Public Health Report, 2000. **7**(11-12): p. 58.
3. *Lassa fever, imported case, Netherlands.* Releve epidemiologique hebdomadaire, 2000. **75**(33): p. 265-265.

4. *Lassa fever imported to England.* Communicable disease report. CDR weekly, 2000. **10**(11): p. 99.
5. *After the "blood diamond" conflict: lassa fever in Sierra Leone.* Clinical infectious diseases : an official publication of the Infectious Diseases Society of America, 2014. **59**(12): p. iii-iv.
6. Abdulraheem, I.S., *Public health importance of lassa fever epidemiology, clinical features and current management review of literature.* African Journal of Clinical and Experimental Microbiology, 2002. **3**(1): p. 33-37.
7. Adogo, L.Y. and M.O. Ogoh, *Review Article: Yellow fever in Nigeria: A review of the current situation.* African Journal of Clinical and Experimental Microbiology, 2020. **21**(1): p. 1-13.
8. Adomeh, D.I., et al., *The Sensitivity and Specificity of Lassa Virus IgM by ELISA as Screening Tool at Early Phase of Lassa Fever Infection.* Nigerian Medical Journal, 2012.
9. Ajayi, N.A., et al., *Containing a Lassa fever epidemic in a resource-limited setting: outbreak description and lessons learned from Abakaliki, Nigeria (January-March 2012).* International journal of infectious diseases : IJID : official publication of the International Society for Infectious Diseases, 2013. **17**(11): p. e1011-e1016.
10. Akhiwu, H.O., et al., *Lassa fever outbreak in adolescents in North Central Nigeria: report of cases.* Journal of virus eradication, 2018. **4**(4): p. 225-227.
11. Akpede, G.O., et al., *Acute Abdomen in Pediatric Patients With Lassa Fever: Prevalence and Response to Nonoperative Management.* Journal of the Pediatric Infectious Diseases Society, 2019. **8**(6): p. 519-524.
12. Akpede, G.O., et al., *Caseload and Case Fatality of Lassa Fever in Nigeria, 2001-2018: A Specialist Center's Experience and Its Implications.* Frontiers in public health, 2019. **7**: p. 170-170.
13. Akpede, G.O., et al., *Lassa fever outbreaks in Nigeria.* Expert review of anti-infective therapy, 2018. **16**(9): p. 663-666.
14. Bausch, D.G., et al., *Diagnosis and clinical virology of Lassa fever as evaluated by enzyme-linked immunosorbent assay, indirect fluorescent-antibody test, and virus isolation.* Journal of clinical microbiology, 2000. **38**(7): p. 2670-2677.
15. Bergmann, J.F., *New African viral fevers Ebola, Lassa and Marburg.* Revue de Medecine, 1982. **23**(35): p. 1859-1864.
16. Bloch, A., *A serological survey of Lassa fever in Liberia.* Bulletin of the World Health Organization, 1978. **56**(5): p. 811-813.
17. Boiro, I., et al., *Experimental studies of haemorrhagic fever in Guinea (Clinical, epidemiological and serological investigations).* Bulletin de la Societe de Pathologie Exotique et de ses Filiales, 1987. **80**(4): p. 607-612.
18. Boisen, M.L., et al., *Field validation of recombinant antigen immunoassays for diagnosis of Lassa fever.* Scientific reports, 2018. **8**(1): p. 5939-5939.
19. Boisen, M.L., et al., *Multiple circulating infections can mimic the early stages of viral hemorrhagic fevers and possible human exposure to filoviruses in sierra leone prior to the 2014 outbreak.* Viral Immunology, 2015. **28**(1): p. 19-31.
20. Bonney, J.H.K., et al., *Molecular detection of dengue virus in patients suspected of Ebola virus disease in Ghana.* PloS one, 2018. **13**(12): p. e0208907-e0208907.
21. Bonney, J.H.K., et al., *Molecular confirmation of Lassa fever imported into Ghana.* African journal of laboratory medicine, 2016. **5**(1): p. 288-288.
22. Borremans, B., et al., *Presence of Mopeia virus, an African arenavirus, related to biotope and individual rodent host characteristics: implications for virus transmission.* Vector borne and zoonotic diseases (Larchmont, N.Y.), 2011. **11**(8): p. 1125-1131.
23. Bowen, M.D., et al., *Genetic diversity among Lassa virus strains.* Journal of virology, 2000. **74**(15): p. 6992-7004.
24. Chare, E.R., E.A. Gould, and E.C. Holmes, *Phylogenetic analysis reveals a low rate of homologous recombination in negative-sense RNA viruses.* Journal of General Virology, 2003. **84**(10): p. 2691-2703.

25. Cummins, D., et al., *Electrocardiographic abnormalities in patients with Lassa fever.* The Journal of tropical medicine and hygiene, 1989. **92**(5): p. 350-355.
26. Cummins, D., et al., *A plasma inhibitor of platelet aggregation in patients with Lassa fever.* British journal of haematology, 1989. **72**(4): p. 543-548.
27. Dedkov, V.G., et al., *Development and Evaluation of a One-Step Quantitative RT-PCR Assay for Detection of Lassa Virus.* Journal of virological methods, 2019. **271**: p. 113674-113674.
28. Dongo, A.E., et al., *Lassa fever presenting as acute abdomen: a case series.* Virology journal, 2013. **10**: p. 123-123.
29. Drosten, C., et al., *Rapid detection and quantification of RNA of Ebola and Marburg viruses, Lassa virus, Crimean-Congo hemorrhagic fever virus, Rift Valley fever virus, dengue virus, and yellow fever virus by real-time reverse transcription-PCR.* Journal of clinical microbiology, 2002. **40**(7): p. 2323-2330.
30. Duvignaud, A., et al., *Delayed-onset paraparesis in Lassa fever: A case report.* International journal of infectious diseases : IJID : official publication of the International Society for Infectious Diseases, 2019: p. S1201-9712(19)30495-3.
31. Dyer, O., *Lassa outbreak: WHO warns of unusually rapid spread in Nigeria.* BMJ (Clinical research ed.), 2019. **364**: p. l781-l781.
32. Dzotsi, E.K., et al., *The first cases of Lassa fever in Ghana.* Ghana medical journal, 2012. **46**(3): p. 166-170.
33. Ehichioya, D.U., et al., *Phylogeography of Lassa Virus in Nigeria.* Journal of virology, 2019. **93**(21): p. e00929-19.
34. Ehichioya, D.U., et al., *Current molecular epidemiology of Lassa virus in Nigeria.* Journal of clinical microbiology, 2011. **49**(3): p. 1157-1161.
35. Ehichioya, D.U., et al., *Lassa fever, Nigeria, 2005-2008.* Emerging infectious diseases, 2010. **16**(6): p. 1040-1041.
36. Eze, K.C., T.A. Salami, and J.U. Kpolugbo, *Acute abdominal pain in patients with lassa fever: Radiological assessment and diagnostic challenges.* Nigerian medical journal : journal of the Nigeria Medical Association, 2014. **55**(3): p. 195-200.
37. Eze, K.C., et al., *High Lassa Fever activity in Northern part of Edo State, Nigeria: reanalysis of confirmatory test results.* African Journal of Health Sciences, 2010. **17**(3&4): p. 52-56.
38. Fatiregun, A.A., et al., *Lassa fever awareness and knowledge among community residents in Ondo State, Nigeria.* Journal of Community Medicine and Primary Health Care, 2019. **31**(2): p. 26-35.
39. Fichet-Calvet, E., *Lassa Fever: A rodent-human interaction*, in *The Role of Animals in Emerging Viral Diseases*. 2013. p. 89-123.
40. Fichet-Calvet, E., et al., *Spatial distribution of commensal rodents in regions with high and low Lassa fever prevalence in Guinea.* Belgian Journal of Zoology, 2005. **135**(SUPPL.1): p. 63-67.
41. Fichet-Calvet, E., et al., *Reproductive characteristics of Mastomys natalensis and Lassa virus prevalence in Guinea, West Africa.* Vector borne and zoonotic diseases (Larchmont, N.Y.), 2008. **8**(1): p. 41-48.
42. Fichet-Calvet, E., et al., *Spatial and temporal evolution of Lassa virus in the natural host population in Upper Guinea.* Scientific reports, 2016. **6**: p. 21977-21977.
43. Frame, J.D., *Surveillance of Lassa fever in missionaries stationed in West Africa.* Bulletin of the World Health Organization, 1975. **52**(4-6): p. 593-598.
44. Frame, J.D., et al., *The use of Lassa fever convalescent plasma in Nigeria.* Transactions of the Royal Society of Tropical Medicine and Hygiene, 1984. **78**(3): p. 319-324.
45. Fraser, D.W., et al., *Lassa fever in the Eastern Province of Sierra Leone, 1970-1972. I. Epidemiologic studies.* The American journal of tropical medicine and hygiene, 1974. **23**(6): p. 1131-1139.
46. Fukuma, A., et al., *Rapid detection of Lassa virus by reverse transcription-loop-mediated isothermal amplification.* Microbiology and Immunology, 2011. **55**(1): p. 44-50.

47. Fyumagwa, R.D., et al., *Response to Rift Valley Fever in Tanzania: Challenges and Opportunities.* Tanzania Journal of Health Research, 2011. **13**(5 Suppl 1): p. 332-339.
48. Gabriel, M., et al., *Development and evaluation of antibody-capture immunoassays for detection of Lassa virus nucleoprotein-specific immunoglobulin M and G.* PLoS neglected tropical diseases, 2018. **12**(3): p. e0006361.
49. Grundy, D.J., E.T. Bowen, and G. Lloyd, *Isolated case of Lassa fever in Zaria, Northern Nigeria.* Lancet (London, England), 1980. **2**(8195 pt 1): p. 649-650.
50. Günther, S., et al., *Antibodies to Lassa virus Z protein and nucleoprotein co-occur in human sera from Lassa fever endemic regions.* Medical microbiology and immunology, 2001. **189**(4): p. 225-229.
51. Ibekwe, T.S., et al., *The sensitivity and specificity of Lassa virus IgM by ELISA as screening tool at early phase of Lassa fever infection.* Nigerian medical journal : journal of the Nigeria Medical Association, 2012. **53**(4): p. 196-199.
52. Ibekwe, T.S., et al., *Early-onset sensorineural hearing loss in Lassa fever.* European Archives of Oto-Rhino-Laryngology, 2011. **268**(2): p. 197-201.
53. Ilori, E.A., et al., *Increase in Lassa Fever Cases in Nigeria, January-March 2018.* Emerging infectious diseases, 2019. **25**(5): p. 1026-1027.
54. Ilori, E.A., et al., *Epidemiologic and Clinical Features of Lassa Fever Outbreak in Nigeria, January 1-May 6, 2018.* Emerging infectious diseases, 2019. **25**(6): p. 1066-1074.
55. Iroezindu, M.O., et al., *Lessons learnt from the management of a case of Lassa fever and follow-up of nosocomial primary contacts in Nigeria during Ebola virus disease outbreak in West Africa.* Tropical Medicine and International Health, 2015. **20**(11): p. 1424-1430.
56. Ishak, K.G., et al., *Viral hemorrhagic fevers with hepatic involvement: pathologic aspects with clinical correlations.* Progress in liver diseases, 1982. **7**: p. 495-515.
57. Kafetzopoulou, L.E., et al., *Metagenomic sequencing at the epicenter of the Nigeria 2018 Lassa fever outbreak.* Science (New York, N.Y.), 2019. **363**(6422): p. 74-77.
58. Keïta, M., et al., *Investigation of a cross-border case of Lassa fever in West Africa.* BMC infectious diseases, 2019. **19**(1): p. 606-606.
59. Knobloch, J., et al., *Clinical observations in 42 patients with lassa fever.* Tropenmedizin und Parasitologie, 1980. **31**(4): p. 389-398.
60. Koehler, J.W., C.E. Douglas, and T.D. Minogue, *A highly multiplexed broad pathogen detection assay for infectious disease diagnostics.* PLoS Neglected Tropical Diseases, 2018. **12**(11).
61. Manning, J.T., N. Forrester, and S. Paessler, *Lassa virus isolates from Mali and the Ivory Coast represent an emerging fifth lineage.* Frontiers in microbiology, 2015. **6**: p. 1037-1037.
62. Mateo, M., et al., *Fatal Case of Lassa Fever, Bangolo District, Côte d'Ivoire, 2015.* Emerging infectious diseases, 2019. **25**(9): p. 1753-1756.
63. Maxmen, A., *Deadly Lassa-fever outbreak tests Nigeria's revamped health agency.* Nature, 2018. **555**(7697): p. 421-422.
64. Mertens, P.E., et al., *Clinical presentation of Lassa fever cases during the hospital epidemic at Zorzor, Liberia, March-April 1972.* The American journal of tropical medicine and hygiene, 1973. **22**(6): p. 780-784.
65. Monath, T.P., et al., *Lassa fever in the Eastern Province of Sierra Leone, 1970-1972. II. Clinical observations and virological studies on selected hospital cases.* The American journal of tropical medicine and hygiene, 1974. **23**(6): p. 1140-1149.
66. Monson, M.H., et al., *Pediatric Lassa fever: a review of 33 Liberian cases.* The American journal of tropical medicine and hygiene, 1987. **36**(2): p. 408-415.
67. Monson, M.H., et al., *Endemic Lassa fever in Liberia. I. Clinical and epidemiological aspects at Curran Lutheran Hospital, Zorzor, Liberia.* Transactions of the Royal Society of Tropical Medicine and Hygiene, 1984. **78**(4): p. 549-553.

68. Niklasson, B.S., P.B. Jahrling, and C.J. Peters, *Detection of Lassa virus antigens and Lassa virus-specific immunoglobulins G and M by enzyme-linked immunosorbent assay.* Journal of clinical microbiology, 1984. **20**(2): p. 239-244.
69. Okuno, T., et al., *Failure to Prove Arenavirus Infection Among the Small Mammals from an Endemic Area of Korean Hemorrhagic Nephrosonephritis.* Japanese Journal of Medical Science and Biology, 1976. **29**(4): p. 187-197.
70. Olowookere, S.A., et al., *Diagnostic proficiency and reporting of Lassa fever by physicians in Osun State of Nigeria.* BMC infectious diseases, 2014. **14**: p. 344-344.
71. Olschläger, S., et al., *Improved detection of Lassa virus by reverse transcription-PCR targeting the 5' region of S RNA.* Journal of clinical microbiology, 2010. **48**(6): p. 2009-2013.
72. Olugasa, B.O., et al., *Development of a time-trend model for analyzing and predicting case-pattern of Lassa fever epidemics in Liberia, 2013-2017.* Annals of African medicine, 2015. **14**(2): p. 89-96.
73. Onyedibe, K., et al., *A cross sectional study of dengue virus infection in febrile patients presumptively diagnosed of malaria in Maiduguri and Jos plateau, Nigeria.* Malawi Medical Journal, 2018. **30**(4): p. 276-282.
74. Organization, W.H., *Update on Lassa fever in West Africa = Le point sur la fièvre de Lassa en Afrique de l'Ouest.* Weekly Epidemiological Record = Relevé épidémiologique hebdomadaire, 2005. **80**(10): p. 86-88.
75. Richards, G.A., J. Weyer, and L.H. Blumberg, *Viral haemorrhagic fevers in South Africa.* SAMJ: South African Medical Journal, 2015. **105**(9): p. 748-751.
76. Roberts, L., *Nigeria hit by unprecedented Lassa fever outbreak.* Science (New York, N.Y.), 2018. **359**(6381): p. 1201-1202.
77. Roth, P.J., et al., *Factors associated with mortality in febrile patients in a government referral hospital in the Kenema district of Sierra Leone.* The American Journal of Tropical Medicine and Hygiene, 2015. **92**(1): p. 172-177.
78. Safronetz, D., et al., *Annual Incidence of Lassa Virus Infection in Southern Mali.* The American journal of tropical medicine and hygiene, 2017. **96**(4): p. 944-946.
79. Salu, O.B., et al., *Biosafety level-2 laboratory diagnosis of Zaire Ebola virus disease imported from Liberia to Nigeria.* African Journal of Laboratory Medicine, 2016. **5**(1).
80. Satterly, N.G., et al., *Comparison of MagPix assays and enzyme-linked immunosorbent assay for detection of hemorrhagic fever viruses.* Journal of Clinical Microbiology, 2017. **55**(1): p. 68-78.
81. Sebba, D., et al., *A point-of-care diagnostic for differentiating Ebola from endemic febrile diseases.* Science translational medicine, 2018. **10**(471): p. eaat0944.
82. Shaffer, J.G., et al., *Lassa Fever in Post-Conflict Sierra Leone.* PLoS Neglected Tropical Diseases, 2014. **8**(3).
83. Shaffer, J.G., et al., *Data set on Lassa fever in post-conflict Sierra Leone.* Data in brief, 2019. **23**: p. 103673-103673.
84. Siddle, K.J., et al., *Genomic Analysis of Lassa Virus during an Increase in Cases in Nigeria in 2018.* The New England journal of medicine, 2018. **379**(18): p. 1745-1753.
85. Singh, N., M.E. Levi, and A.I.D.C.o. Practice, *Arenavirus and West Nile virus in solid organ transplantation.* American Journal of Transplantation: Official Journal of the American Society of Transplantation and the American Society of Transplant Surgeons, 2013. **13 Suppl 4**: p. 361-371.
86. Stremlau, M.H., et al., *Discovery of Novel Rhabdoviruses in the Blood of Healthy Individuals from West Africa.* PLoS Neglected Tropical Diseases, 2015. **9**(3).
87. Stremlau, M.H., et al., *Discovery of novel rhabdoviruses in the blood of healthy individuals from West Africa.* PLoS neglected tropical diseases, 2015. **9**(3): p. e0003631.
88. Tambo, E., O.T. Adetunde, and O.A. Olalubi, *Re-emerging Lassa fever outbreaks in Nigeria: Re-enforcing "One Health" community surveillance and emergency response practice.* Infectious diseases of poverty, 2018. **7**(1): p. 37-37.

89. ter Meulen, J., et al., *Short communication: Lassa fever in Sierra Leone: UN peacekeepers are at risk.* Tropical medicine & international health : TM & IH, 2001. **6**(1): p. 83-84.
90. Trappier, S.G., et al., *Evaluation of the polymerase chain reaction for diagnosis of Lassa virus infection.* The American journal of tropical medicine and hygiene, 1993. **49**(2): p. 214-221.
91. Usifoh, S.F., et al., *Impact of lassa fever on the practice and consumption of stored food by university of Benin community, in Benin city, Nigeria.* Journal of Community Medicine and Primary Health Care, 2018. **30**(1): p. 66-76.
92. Usifoh, S.F., et al., *Lassa Fever-associated Stigmatization among Staff and Students of the University of Benin, Nigeria.* Journal of epidemiology and global health, 2019. **9**(2): p. 107-115.
93. Vieth, S., et al., *RT-PCR assay for detection of Lassa virus and related Old World arenaviruses targeting the L gene.* Transactions of the Royal Society of Tropical Medicine and Hygiene, 2007. **101**(12): p. 1253-1264.
94. White, H.A., *Lassa fever A study of 23 hospital cases.* Transactions of the Royal Society of Tropical Medicine and Hygiene, 1972. **66**(3): p. 390-398.
95. Whitmer, S.L.M., et al., *New Lineage of Lassa Virus, Togo, 2016.* Emerging infectious diseases, 2018. **24**(3): p. 599-602.
96. Wiley, M.R., et al., *Lassa virus circulating in Liberia: a retrospective genomic characterisation.* The Lancet Infectious Diseases, 2019. **19**(12): p. 1371-1378.
97. Wolff, S., et al., *Genome sequence of Lassa virus isolated from the first domestically acquired case in Germany.* Genome Announcements, 2016. **4**(5).
