# Supplementary material for: Systematic review and meta-analysis of the epidemiology of Lassa virus in humans, rodents and other mammals in sub-Saharan Africa
Source: PLoS Negl Trop Dis. 2020 Aug 26;14(8):e0008589. doi: 10.1371/journal.pntd.0008589 (PMC7478710; doi:10.1371/journal.pntd.0008589)
Supplement: S5 Table — (PDF) [file pntd.0008589.s005.pdf]

S5 Table: Risk of bias assessment

Author	Was the study's target population a close representation of the national population in relation to LASV prevalence?	Was the sampling frame a true or close representation of the target population?	Was some form of random selection used to select the sample, OR was a census undertaken?	Were data collected directly from the subjects (as opposed to a proxy)?	Was an acceptable clinical case definition used in the study?	Was the response rate ≥ 70 % or not significant different in relevant demographic characteristics between responders and non-responders?	Was the LASV detection assay shown to have reliability and validity?	Was the same mode of data collection used for all subjects?	Was the length of the study period less than 1 year?	Were the numerator(s) and denominator(s) for the LASV prevalence or case fatality rate appropriate?	species	Risk of bias
Agbonlahor, 2017	No	Yes	No	Not applicable (for animals)	No	No	Yes	Yes	Yes	Yes	Rodents	Moderate risk of bias
Agbonlahor, 2017	No	Yes	No	Not applicable (for animals)	No	No	Yes	Yes	Yes	Yes	Rodents	Moderate risk of bias
Agbonlahor, 2017	No	Yes	No	Not applicable (for animals)	No	No	Yes	Yes	Yes	Yes	Rodents	Moderate risk of bias
Agbonlahor, 2017	No	Yes	No	Not applicable (for animals)	No	No	Yes	Yes	Yes	Yes	Rodents	Moderate risk of bias
Akhuemokhan, 2017	No	Yes	No	Yes	Yes	No	Yes	Yes	No	Yes	Humans	Moderate risk of bias
Akoua-Koffi, 2006	No	Yes	No	Yes	Yes	No	Yes	Yes	Yes	Yes	Humans	Low risk of bias
Arnold, 1977	No	Yes	No	Yes	Yes	No	Yes	No	No	Yes	Humans	Moderate risk of bias
Arnold, 1977	No	Yes	No	Yes	Yes	No	Yes	No	No	Yes	Humans	Moderate risk of bias
Asogun, 2012	No	Yes	No	Yes	Yes	No	Yes	Yes	Yes	Yes	Humans	Low risk of bias
Babalola, 2019	No	Yes	No	Yes	No	No	Yes	Yes	Yes	Yes	Humans	Moderate risk of bias
Bajani, 1997	No	Yes	No	Yes	Yes	No	Yes	Yes	Yes	Yes	Humans	Low risk of bias
Bajani, 1997	No	Yes	No	Yes	Yes	No	Yes	Yes	Yes	Yes	Humans	Low risk of bias
Baumann, 2019	No	Yes	No	Yes	No	No	Yes	Yes	Yes	Yes	Humans	Moderate risk of bias
Bausch, 2001	No	Yes	No	Yes	Yes	No	Yes	Yes	Yes	Yes	Humans	Low risk of bias
Blackburn, 1982	No	Yes	No	Yes	Yes	No	Yes	Yes	Yes	Yes	Humans	Low risk of bias
Boiro, 1987	No	Yes	No	Yes	No	No	Yes	No	Yes	Yes	Humans	Moderate risk of bias
Boiro, 1987	No	Yes	No	Not applicable (for animals)	No	No	Yes	Yes	Yes	Yes	Rodents	Moderate risk of bias
Bonney, 2013	No	Yes	No	Yes	No	No	Yes	Yes	Yes	Yes	Humans	Moderate risk of bias
Bowen, 1975	No	Yes	No	Not applicable (for animals)	Yes	No	Yes	Yes	No	Yes	Rodents	Moderate risk of bias
Bowen, 1975	No	Yes	No	Yes	Yes	No	Yes	No	No	Yes	Humans	Moderate risk of bias
Branco, 2011	No	Yes	No	Yes	Yes	No	Yes	Yes	No	Yes	Humans	Moderate risk of bias
Branco, 2011	No	Yes	No	Yes	Yes	No	Yes	Yes	No	Yes	Humans	Moderate risk of bias
Branco, 2011	No	Yes	No	Yes	Yes	No	Yes	Yes	No	Yes	Humans	Moderate risk of bias
Branco, 2011	No	Yes	No	Yes	Yes	No	Yes	Yes	No	Yes	Humans	Moderate risk of bias
Branco, 2011	No	Yes	No	Yes	Yes	No	Yes	Yes	No	Yes	Humans	Moderate risk of bias
Buba, 2018	No	Yes	No	Yes	Yes	No	Yes	Yes	Yes	No	Humans	Moderate risk of bias
Bukbuk, 2014	No	Yes	Yes	Yes	No	No	Yes	Yes	Yes	Yes	Humans	Low risk of bias
Carey, 1972	No	Yes	No	Not applicable (for animals)	No	No	Yes	Yes	Yes	Yes	Rodents	Moderate risk of bias
Carey, 1972	No	Yes	No	Yes	No	No	Yes	Yes	Yes	Yes	Humans	Moderate risk of bias
Carey, 1972	No	Yes	No	Not applicable (for animals)	No	No	Yes	Yes	Yes	Yes	Other mammals	Moderate risk of bias
Carey, 1972	No	Yes	No	Yes	No	No	Yes	Yes	Yes	Yes	Humans	Moderate risk of bias
Carey, 1972	No	Yes	No	Yes	No	No	Yes	Yes	Yes	Yes	Humans	Moderate risk of bias
Clements, 2019	No	Yes	No	Yes	No	No	Yes	Yes	Yes	Yes	Humans	Moderate risk of bias
Coulibaly-N'Golo, 2011	No	Yes	No	Not applicable (for animals)	No	No	Yes	Yes	Yes	Yes	Rodents	Moderate risk of bias
Coulibaly-N'Golo, 2011	No	Yes	No	Not applicable (for animals)	No	No	Yes	Yes	Yes	Yes	Rodents	Moderate risk of bias
Coulibaly-N'Golo, 2011	No	Yes	No	Not applicable (for animals)	No	No	Yes	Yes	Yes	Yes	Rodents	Moderate risk of bias
Coulibaly-N'Golo, 2011	No	Yes	No	Not applicable (for animals)	No	No	Yes	Yes	Yes	Yes	Rodents	Moderate risk of bias
Coulibaly-N'Golo, 2011	No	Yes	No	Not applicable (for animals)	No	No	Yes	Yes	Yes	Yes	Rodents	Moderate risk of bias
Coulibaly-N'Golo, 2011	No	Yes	No	Not applicable (for animals)	No	No	Yes	Yes	Yes	Yes	Rodents	Moderate risk of bias
Coulibaly-N'Golo, 2011	No	Yes	No	Not applicable (for animals)	No	No	Yes	Yes	Yes	Yes	Rodents	Moderate risk of bias
Cummins, 1990	No	Yes	No	Yes	Yes	No	Yes	Yes	Yes	Yes	Humans	Low risk of bias
Dahmane, 2014	No	Yes	No	Yes	Yes	No	Yes	Yes	Yes	Yes	Humans	Low risk of bias
Dahmane, 2014	No	Yes	No	Yes	Yes	No	Yes	Yes	Yes	Yes	Humans	Low risk of bias
Dahmane, 2014	No	Yes	No	Yes	Yes	No	Yes	Yes	Yes	Yes	Humans	Low risk of bias
Dan-Nwafor, 2019	No	Yes	No	Yes	No	No	Yes	No	Yes	Yes	Humans	Moderate risk of bias
Dan-Nwafor, 2019	No	Yes	No	Yes	Yes	No	Yes	Yes	Yes	Yes	Humans	Low risk of bias
Demartini, 1975	No	Yes	No	Not applicable (for animals)	No	No	Yes	Yes	Yes	Yes	Rodents	Moderate risk of bias
Demby, 1994	No	Yes	No	Yes	Yes	No	Yes	Yes	Yes	Yes	Humans	Low risk of bias
Demby, 1994	No	Yes	No	Yes	Yes	No	Yes	Yes	Yes	Yes	Humans	Low risk of bias
Demby, 1994	No	Yes	No	Yes	Yes	No	Yes	Yes	Yes	Yes	Humans	Low risk of bias
Demby, 2001	No	Yes	Yes	Not applicable (for animals)	No	No	Yes	Yes	Yes	Yes	Rodents	Moderate risk of bias
Demby, 2001	No	Yes	Yes	Not applicable (for animals)	No	No	Yes	Yes	Yes	Yes	Rodents	Moderate risk of bias
Demby, 2001	No	Yes	Yes	Not applicable (for animals)	No	No	Yes	Yes	Yes	Yes	Rodents	Moderate risk of bias

Demby, 2001	No	Yes	Yes	Not applicable (for animals)	No	No	Yes	Yes	Yes	Yes	Rodents	Moderate risk of bias
Demby, 2001	No	Yes	Yes	Not applicable (for animals)	No	No	Yes	Yes	Yes	Yes	Rodents	Moderate risk of bias
Demby, 2001	No	Yes	Yes	Not applicable (for animals)	No	No	Yes	Yes	Yes	Yes	Rodents	Moderate risk of bias
Ehichioya , 2012	No	Yes	No	Yes	Yes	No	Yes	Yes	Yes	Yes	Humans	Low risk of bias
Ehichioya , 2012	No	Yes	No	Yes	Yes	No	Yes	Yes	Yes	Yes	Humans	Low risk of bias
Ehichioya , 2012	No	Yes	No	Yes	Yes	No	Yes	Yes	Yes	Yes	Humans	Low risk of bias
Ehichioya , 2012	No	Yes	No	Yes	Yes	No	Yes	Yes	Yes	Yes	Humans	Low risk of bias
Emmerich, 2006	No	Yes	No	Yes	Yes	No	Yes	Yes	Unclear	Yes	Humans	Moderate risk of bias
Emmerich, 2006	No	Yes	No	Yes	Yes	No	Yes	Yes	Unclear	Yes	Humans	Moderate risk of bias
Emmerich, 2008	No	Yes	No	Yes	Yes	No	Yes	Yes	Unclear	Yes	Humans	Moderate risk of bias
Emmerich, 2008	No	Yes	No	Yes	Yes	No	Yes	Yes	Unclear	Yes	Humans	Moderate risk of bias
Fabiyi, 1975	No	Yes	No	Yes	No	No	Yes	No	Yes	Yes	Humans	Moderate risk of bias
Fabiyi, 1979	No	Yes	No	Yes	No	No	Yes	Yes	Unclear	Yes	Humans	Moderate risk of bias
Fabiyi, 1979	No	Yes	No	Yes	No	No	Yes	Yes	Unclear	Yes	Humans	Moderate risk of bias
Fair, 2007	No	Yes	No	Not applicable (for animals)	No	No	Yes	Yes	No	Yes	Rodents	Moderate risk of bias
Fichet-Calvet, 2007	No	Yes	No	Not applicable (for animals)	No	No	Yes	Yes	Yes	Yes	Rodents	Moderate risk of bias
Fichet-Calvet, 2007	No	Yes	No	Not applicable (for animals)	No	No	Yes	Yes	Yes	Yes	Rodents	Moderate risk of bias
Fichet-Calvet, 2007	No	Yes	No	Not applicable (for animals)	No	No	Yes	Yes	Yes	Yes	Rodents	Moderate risk of bias
Fichet-Calvet, 2007	No	Yes	No	Not applicable (for animals)	No	No	Yes	Yes	Yes	Yes	Rodents	Moderate risk of bias
Fichet-Calvet, 2007	No	Yes	No	Not applicable (for animals)	No	No	Yes	Yes	Yes	Yes	Rodents	Moderate risk of bias
Fichet-Calvet, 2007	No	Yes	No	Not applicable (for animals)	No	No	Yes	Yes	Yes	Yes	Rodents	Moderate risk of bias
Fichet-Calvet, 2014	No	Yes	No	Not applicable (for animals)	Yes	No	Yes	Yes	Yes	Yes	Rodents	Moderate risk of bias
Fichet-Calvet, 2014	No	Yes	No	Not applicable (for animals)	Yes	No	Yes	Yes	Yes	Yes	Rodents	Moderate risk of bias
Fichet-Calvet, 2014	No	Yes	No	Not applicable (for animals)	Yes	No	Yes	Yes	Yes	Yes	Rodents	Moderate risk of bias
Fichet-Calvet, 2014	No	Yes	No	Not applicable (for animals)	Yes	No	Yes	Yes	Yes	Yes	Rodents	Moderate risk of bias
Fichet-Calvet, 2014	No	Yes	No	Not applicable (for animals)	Yes	No	Yes	Yes	Yes	Yes	Rodents	Moderate risk of bias
Fichet-Calvet, 2014	No	Yes	No	Not applicable (for animals)	Yes	No	Yes	Yes	Yes	Yes	Rodents	Moderate risk of bias
Fichet-Calvet, 2014	No	Yes	No	Not applicable (for animals)	Yes	No	Yes	Yes	Yes	Yes	Rodents	Moderate risk of bias
Fichet-Calvet, 2014	No	Yes	No	Not applicable (for animals)	Yes	No	Yes	Yes	Yes	Yes	Rodents	Moderate risk of bias
Fichet-Calvet, 2014	No	Yes	No	Not applicable (for animals)	Yes	No	Yes	Yes	Yes	Yes	Rodents	Moderate risk of bias
Fichet-Calvet, 2014	No	Yes	No	Not applicable (for animals)	Yes	No	Yes	Yes	Yes	Yes	Rodents	Moderate risk of bias
Fichet-Calvet, 2014	No	Yes	No	Not applicable (for animals)	Yes	No	Yes	Yes	Yes	Yes	Rodents	Moderate risk of bias
Fichet-Calvet, 2014	No	Yes	No	Not applicable (for animals)	Yes	No	Yes	Yes	Yes	Yes	Rodents	Moderate risk of bias
Fichet-Calvet, 2014	No	Yes	No	Not applicable (for animals)	Yes	No	Yes	Yes	Yes	Yes	Rodents	Moderate risk of bias
Fichet-Calvet, 2014	No	Yes	No	Not applicable (for animals)	Yes	No	Yes	Yes	Yes	Yes	Rodents	Moderate risk of bias
Fisher-Hoch, 1988	No	Yes	No	Yes	Yes	No	Yes	Yes	Yes	Yes	Humans	Low risk of bias
Fisher-Hoch, 1995	No	Yes	No	Yes	Yes	No	Yes	Yes	No	Yes	Humans	Moderate risk of bias
Fisher-Hoch, 1995	No	Yes	No	Yes	Yes	No	Yes	Yes	No	Yes	Humans	Moderate risk of bias
Fisher-Hoch, 1995	No	Yes	No	Yes	Yes	No	Yes	Yes	No	Yes	Humans	Moderate risk of bias
Frame, 1979	No	Yes	No	Yes	No	No	Yes	Yes	Yes	Yes	Humans	Moderate risk of bias
Frame, 1984	No	Yes	No	Yes	Yes	No	Yes	No	Yes	Yes	Humans	Moderate risk of bias
Frame, 1984	No	Yes	No	Yes	Yes	No	Yes	No	Yes	Yes	Humans	Moderate risk of bias
Frame, 1984	No	Yes	No	Yes	No	No	Yes	Yes	Yes	Yes	Humans	Moderate risk of bias
Georges, 1985	No	Yes	No	Not applicable (for animals)	No	No	Yes	Yes	Yes	Yes	Other mammals	Moderate risk of bias
Georges, 1985	No	Yes	No	Not applicable (for animals)	No	No	Yes	Yes	Yes	Yes	Other mammals	Moderate risk of bias
Georges, 1985	No	Yes	No	Not applicable (for animals)	No	No	Yes	Yes	Yes	Yes	Other mammals	Moderate risk of bias
Georges, 1985	No	Yes	No	Not applicable (for animals)	No	No	Yes	Yes	Yes	Yes	Other mammals	Moderate risk of bias
Georges, 1985	No	Yes	No	Not applicable (for animals)	No	No	Yes	Yes	Yes	Yes	Other mammals	Moderate risk of bias
Georges, 1985	No	Yes	No	Yes	No	No	Yes	Yes	Yes	Yes	Humans	Moderate risk of bias
Gonzalez, 1983	No	Yes	No	Not applicable (for animals)	No	No	Yes	Yes	Unclear	Yes	Rodents	Moderate risk of bias
Gonzalez, 1983	No	Yes	No	Not applicable (for animals)	No	No	Yes	Yes	Unclear	Yes	Humans	Moderate risk of bias
Gonzalez, 1983	No	Yes	No	Not applicable (for animals)	No	No	Yes	Yes	Unclear	Yes	Rodents	Moderate risk of bias
Gonzalez, 1989	No	Yes	Yes	Not applicable (for animals)	No	No	Yes	Yes	Yes	No	Rodents	Moderate risk of bias
Gonzalez, 1989	No	Yes	Yes	Yes	No	No	Yes	Yes	Yes	No	Humans	Moderate risk of bias
Gonzalez, 1989	No	Yes	Yes	Yes	No	No	Yes	Yes	Yes	No	Humans	Moderate risk of bias
Gonzalez, 1989	No	Yes	Yes	Yes	No	No	Yes	Yes	Yes	No	Humans	Moderate risk of bias
Gonzalez, 1989	No	Yes	Yes	Yes	No	No	Yes	Yes	Yes	No	Humans	Moderate risk of bias
Gonzalez, 1989	No	Yes	Yes	Yes	No	No	Yes	Yes	Yes	No	Humans	Moderate risk of bias
Gonzalez, 1989	No	Yes	Yes	Yes	No	No	Yes	Yes	Yes	No	Humans	Moderate risk of bias
Hamblion, 2018	No	Yes	No	Yes	Yes	No	Yes	No	Yes	Yes	Humans	Moderate risk of bias
Haun, 2019	No	Yes	No	Not applicable (for animals)	Yes	No	Yes	Yes	No	Yes	Other mammals	Moderate risk of bias

Helmick, 1986	No	Yes	No	Yes	No	No	Yes	Yes	Yes	Yes	Humans	Moderate risk of bias
Helmick, 1986	No	Yes	No	Yes	No	No	Yes	Yes	Yes	Yes	Humans	Moderate risk of bias
Henderson, 1972	No	Yes	No	Yes	No	No	Yes	Yes	Yes	Yes	Humans	Moderate risk of bias
Henderson, 1972	No	Yes	No	Yes	No	No	Yes	Yes	Yes	Yes	Humans	Moderate risk of bias
Henderson, 1972	No	Yes	No	Yes	No	No	Yes	Yes	Yes	Yes	Humans	Moderate risk of bias
Henderson, 1972	No	Yes	No	Yes	No	No	Yes	Yes	Yes	Yes	Humans	Moderate risk of bias
Henderson, 1972	No	Yes	No	Yes	No	No	Yes	Yes	Yes	Yes	Humans	Moderate risk of bias
Henderson, 1972	No	Yes	No	Yes	No	No	Yes	Yes	Yes	Yes	Humans	Moderate risk of bias
Henderson, 1972	No	Yes	No	Yes	No	No	Yes	Yes	Yes	Yes	Humans	Moderate risk of bias
Henderson, 1972	No	Yes	No	Yes	No	No	Yes	Yes	Yes	Yes	Humans	Moderate risk of bias
Henderson, 1972	No	Yes	No	Yes	No	No	Yes	Yes	Yes	Yes	Humans	Moderate risk of bias
Henderson, 1972	No	Yes	No	Yes	No	No	Yes	Yes	Yes	Yes	Humans	Moderate risk of bias
Henderson, 1972	No	Yes	No	Yes	No	No	Yes	Yes	Yes	Yes	Humans	Moderate risk of bias
Henderson, 1972	No	Yes	No	Yes	No	No	Yes	Yes	Yes	Yes	Humans	Moderate risk of bias
Henderson, 1972	No	Yes	No	Yes	No	No	Yes	Yes	Yes	Yes	Humans	Moderate risk of bias
Henderson, 1972	No	Yes	No	Yes	No	No	Yes	Yes	Yes	Yes	Humans	Moderate risk of bias
Henderson, 1972	No	Yes	No	Yes	No	No	Yes	Yes	Yes	Yes	Humans	Moderate risk of bias
Henderson, 1972	No	Yes	No	Yes	No	No	Yes	Yes	Yes	Yes	Humans	Moderate risk of bias
Ibekwe, 2012	No	Yes	No	Yes	Yes	No	Yes	Yes	Yes	Yes	Humans	Low risk of bias
Ibekwe, 2012	No	Yes	No	Yes	Yes	No	Yes	Yes	Yes	Yes	Humans	Low risk of bias
Ipadeola, 2020	No	Yes	No	Yes	No	No	Yes	Yes	Yes	Yes	Humans	Moderate risk of bias
Isere, 2018	No	Yes	No	Yes	Yes	No	Yes	Yes	No	Yes	Humans	Moderate risk of bias
Ivanoff, 1982	No	Yes	No	Not applicable (for animals)	No	No	Yes	Yes	Yes	Yes	Other mammals	Moderate risk of bias
Ivanoff, 1982	No	Yes	No	Yes	No	No	Yes	Yes	Yes	Yes	Humans	Moderate risk of bias
Jahrling, 1985	No	Yes	No	Yes	Yes	No	Yes	No	Yes	Yes	Humans	Moderate risk of bias
Johnson, 1982	No	Yes	No	Not applicable (for animals)	No	No	Yes	Yes	Unclear	Yes	Other mammals	Moderate risk of bias
Johnson, 1982	No	Yes	No	Yes	No	No	Yes	Yes	Unclear	Yes	Humans	Moderate risk of bias
Johnson, 1983	No	Yes	No	Yes	No	No	Yes	Yes	Unclear	Yes	Humans	Moderate risk of bias
Johnson, 1983	No	Yes	No	Yes	No	No	Yes	No	Yes	Yes	Humans	Moderate risk of bias
Johnson, 1993	No	Yes	No	Yes	No	No	Yes	Yes	Unclear	Yes	Humans	Moderate risk of bias
Keane, 1977	No	Yes	No	Yes	No	No	Yes	Yes	Yes	Yes	Humans	Moderate risk of bias
Keenlyside, 1983	No	Yes	Yes	Not applicable (for animals)	No	No	Yes	Yes	Unclear	Yes	Rodents	Moderate risk of bias
Keenlyside, 1983	No	Yes	Yes	Yes	No	No	Yes	Yes	Unclear	Yes	Humans	Moderate risk of bias
Kernéis, 2009	No	Yes	Yes	Yes	Yes	No	Yes	Yes	Yes	Yes	Humans	Low risk of bias
Klempa, 2013	No	Yes	No	Yes	Yes	No	Yes	No	Yes	Yes	Humans	Moderate risk of bias
Klempa, 2013	No	Yes	No	Yes	Yes	No	Yes	No	Yes	Yes	Humans	Moderate risk of bias
Klempa, 2013	No	Yes	No	Yes	Yes	No	Yes	No	Yes	Yes	Humans	Moderate risk of bias
Knobloch, 1982	No	Yes	No	Yes	No	No	Yes	Yes	Yes	Yes	Humans	Moderate risk of bias
Knobloch, 1982	No	Yes	No	Yes	Not applicable	No	Yes	Yes	Yes	Yes	Humans	Moderate risk of bias
Knobloch, 1982	No	Yes	No	Yes	No	No	Yes	Yes	Yes	Yes	Humans	Moderate risk of bias
Knobloch, 1982	No	Yes	No	Yes	Not applicable	No	Yes	Yes	Yes	Yes	Humans	Moderate risk of bias
Kouadio , 2015	No	Yes	Yes	Not applicable (for animals)	No	No	Yes	Yes	Yes	Yes	Rodents	Moderate risk of bias
Lalis , 2012	No	Yes	Yes	Not applicable (for animals)	No	No	Yes	Yes	Yes	Yes	Rodents	Moderate risk of bias
Lecompte, 2006	No	Yes	No	Not applicable (for animals)	Yes	No	Yes	Yes	Yes	Yes	Rodents	Moderate risk of bias
Lecompte, 2006	No	Yes	No	Not applicable (for animals)	Yes	No	Yes	Yes	Yes	Yes	Rodents	Moderate risk of bias
Lecompte, 2006	No	Yes	No	Not applicable (for animals)	Yes	No	Yes	Yes	Yes	Yes	Rodents	Moderate risk of bias
Lecompte, 2006	No	Yes	No	Not applicable (for animals)	Yes	No	Yes	Yes	Yes	Yes	Rodents	Moderate risk of bias
Lecompte, 2006	No	Yes	No	Not applicable (for animals)	Yes	No	Yes	Yes	Yes	Yes	Rodents	Moderate risk of bias
Lecompte, 2006	No	Yes	No	Not applicable (for animals)	Yes	No	Yes	Yes	Yes	Yes	Rodents	Moderate risk of bias
Lecompte, 2006	No	Yes	No	Not applicable (for animals)	Yes	No	Yes	Yes	Yes	Yes	Rodents	Moderate risk of bias
Lecompte, 2006	No	Yes	No	Not applicable (for animals)	Yes	No	Yes	Yes	Yes	Yes	Rodents	Moderate risk of bias
Lecompte, 2006	No	Yes	No	Not applicable (for animals)	Yes	No	Yes	Yes	Yes	Yes	Rodents	Moderate risk of bias
Lecompte, 2006	No	Yes	No	Not applicable (for animals)	Yes	No	Yes	Yes	Yes	Yes	Rodents	Moderate risk of bias
Leski (GPC gene), 2015	No	Yes	No	Not applicable (for animals)	Yes	No	Yes	Yes	Yes	Yes	Rodents	Moderate risk of bias
Leski (NP gene), 2015	No	Yes	No	Not applicable (for animals)	Yes	No	Yes	Yes	Yes	Yes	Rodents	Moderate risk of bias
Leski (NP gene), 2015	No	Yes	No	Not applicable (for animals)	Yes	No	Yes	Yes	Yes	Yes	Rodents	Moderate risk of bias
Leski, 2015	No	Yes	No	Not applicable (for animals)	Yes	No	Yes	Yes	Yes	Yes	Rodents	Moderate risk of bias
Li, 2016	No	Yes	No	Yes	Yes	No	Yes	Yes	Yes	Yes	Humans	Low risk of bias
Lukashevich, 1993	No	Yes	No	Yes	No	No	Yes	Yes	Yes	Yes	Humans	Moderate risk of bias
Lukashevich, 1993	No	Yes	No	Yes	No	No	Yes	Yes	Yes	Yes	Humans	Moderate risk of bias
Lukashevich, 1993	No	Yes	No	Yes	No	No	Yes	Yes	Yes	Yes	Humans	Moderate risk of bias

Maigari, 2018	No	Yes	No	Yes	No	No	Yes	Yes	Yes	Yes	Humans	Moderate risk of bias
Mariën, 2017	No	Yes	No	Not applicable (for animals)	No	No	Yes	Yes	Yes	Yes	Rodents	Moderate risk of bias
Mariën, 2017	No	Yes	No	Not applicable (for animals)	No	No	Yes	Yes	Yes	Yes	Rodents	Moderate risk of bias
Mathiot, 1989	No	Yes	No	Yes	No	No	Yes	Yes	Unclear	Yes	Humans	Moderate risk of bias
McCarthy, 1996	No	Yes	No	Yes	Yes	No	Yes	No	Yes	Yes	Humans	Moderate risk of bias
McCormick, 1987	No	Yes	No	Yes	Yes	No	Yes	Yes	Yes	Yes	Humans	Low risk of bias
McCormick, 1987	No	Yes	Yes	Yes	No	No	Yes	Yes	Unclear	Yes	Humans	Moderate risk of bias
McCormick, 1987	No	Yes	Yes	Not applicable (for animals)	No	No	Yes	Yes	Unclear	Yes	Rodents	Moderate risk of bias
Meunier, 1987	No	Yes	No	Yes	No	No	Yes	Yes	Yes	Yes	Humans	Moderate risk of bias
Monath, 1973	No	Yes	No	Yes	No	No	Yes	Yes	Yes	Yes	Humans	Moderate risk of bias
Monath, 1973	No	Yes	No	Yes	No	No	Yes	Yes	Yes	Yes	Humans	Moderate risk of bias
Monath, 1973	No	Yes	No	Yes	No	No	Yes	Yes	Yes	Yes	Humans	Moderate risk of bias
Monath, 1974	No	Yes	No	Not applicable (for animals)	No	No	Yes	Yes	Yes	Yes	Rodents	Moderate risk of bias
Monath, 1974	No	Yes	No	Not applicable (for animals)	No	No	Yes	Yes	Yes	Yes	Other mammals	Moderate risk of bias
Monath, 1974	No	Yes	No	Not applicable (for animals)	No	No	Yes	Yes	Yes	Yes	Other mammals	Moderate risk of bias
Monath, 1974	No	Yes	No	Not applicable (for animals)	No	No	Yes	Yes	Yes	Yes	Rodents	Moderate risk of bias
Nakounné, 2000	No	Yes	No	Yes	Yes	No	Yes	Yes	Yes	Yes	Humans	Low risk of bias
Nakounné, 2000	No	Yes	No	Yes	Yes	No	Yes	Yes	Yes	Yes	Humans	Low risk of bias
Nakounné, 2000	No	Yes	No	Yes	Yes	No	Yes	Yes	Yes	Yes	Humans	Low risk of bias
Nakounné, 2000	No	Yes	No	Yes	Yes	No	Yes	Yes	Yes	Yes	Humans	Low risk of bias
Nimo-Paintsil, 2019	No	Yes	No	Yes	Yes	No	Yes	Yes	Yes	Yes	Humans	Low risk of bias
Ogunro, 2019	No	Yes	No	Not applicable (for animals)	No	No	Yes	Yes	Yes	Yes	Other mammals	Moderate risk of bias
Ogunro, 2019	No	Yes	No	Not applicable (for animals)	No	No	Yes	Yes	Yes	Yes	Other mammals	Moderate risk of bias
Ogunro, 2019	No	Yes	No	Not applicable (for animals)	No	No	Yes	Yes	Yes	Yes	Other mammals	Moderate risk of bias
O'Hearn, 2016	No	Yes	No	Yes	No	No	Yes	Yes	Yes	Yes	Humans	Moderate risk of bias
Okogbenin, 2019	No	Yes	No	Yes	Yes	No	Yes	Yes	Yes	Yes	Humans	Low risk of bias
Okokhere, 2018	No	Yes	No	Yes	Yes	No	Yes	Yes	Yes	Yes	Humans	Low risk of bias
Okoror, 2005	No	Yes	No	Not applicable (for animals)	No	No	Yes	Yes	Yes	Yes	Rodents	Moderate risk of bias
Okoror, 2005	No	Yes	No	Not applicable (for animals)	No	No	Yes	Yes	Yes	Yes	Rodents	Moderate risk of bias
Olayemi, 2016	No	Yes	No	Not applicable (for animals)	No	No	Yes	Yes	Yes	Yes	Rodents	Moderate risk of bias
Olayemi, 2016	No	Yes	No	Not applicable (for animals)	No	No	Yes	Yes	Yes	Yes	Rodents	Moderate risk of bias
Olayemi, 2016	No	Yes	No	Not applicable (for animals)	No	No	Yes	Yes	Yes	Yes	Rodents	Moderate risk of bias
Olayemi, 2016	No	Yes	No	Not applicable (for animals)	No	No	Yes	Yes	Yes	Yes	Rodents	Moderate risk of bias
Olayemi, 2016	No	Yes	No	Not applicable (for animals)	No	No	Yes	Yes	Yes	Yes	Rodents	Moderate risk of bias
Olayemi, 2018	No	Yes	Yes	Not applicable (for animals)	No	No	Yes	Yes	Yes	Yes	Rodents	Moderate risk of bias
Olayemi, 2018	No	Yes	Yes	Not applicable (for animals)	No	No	Yes	Yes	Yes	Yes	Rodents	Moderate risk of bias
Olayemi, 2018	No	Yes	Yes	Not applicable (for animals)	No	No	Yes	Yes	Yes	Yes	Rodents	Moderate risk of bias
Olayemi, 2018	No	Yes	Yes	Not applicable (for animals)	No	No	Yes	Yes	Yes	Yes	Rodents	Moderate risk of bias
Olayemi, 2018	No	Yes	Yes	Not applicable (for animals)	No	No	Yes	Yes	Yes	Yes	Rodents	Moderate risk of bias
Olayemi, 2018	No	Yes	Yes	Not applicable (for animals)	No	No	Yes	Yes	Yes	Yes	Rodents	Moderate risk of bias
Olayemi, 2018	No	Yes	Yes	Not applicable (for animals)	No	No	Yes	Yes	Yes	Yes	Rodents	Moderate risk of bias
Olayemi, 2018	No	Yes	Yes	Not applicable (for animals)	No	No	Yes	Yes	Yes	Yes	Rodents	Moderate risk of bias
Olayemi, 2018	No	Yes	Yes	Not applicable (for animals)	No	No	Yes	Yes	Yes	Yes	Rodents	Moderate risk of bias
Olayemi, 2018	No	Yes	Yes	Not applicable (for animals)	No	No	Yes	Yes	Yes	Yes	Rodents	Moderate risk of bias
Olayemi, 2018	No	Yes	Yes	Not applicable (for animals)	No	No	Yes	Yes	Yes	Yes	Other mammals	Moderate risk of bias
Olayemi, 2018	No	Yes	Yes	Not applicable (for animals)	No	No	Yes	Yes	Yes	Yes	Rodents	Moderate risk of bias
Olayemi, 2018	No	Yes	Yes	Not applicable (for animals)	No	No	Yes	Yes	Yes	Yes	Rodents	Moderate risk of bias
Olayemi, 2018	No	Yes	Yes	Not applicable (for animals)	No	No	Yes	Yes	Yes	Yes	Other mammals	Moderate risk of bias
Olayemi, 2018	No	Yes	Yes	Not applicable (for animals)	No	No	Yes	Yes	Yes	Yes	Rodents	Moderate risk of bias
Olayemi, 2018	No	Yes	Yes	Not applicable (for animals)	No	No	Yes	Yes	Yes	Yes	Rodents	Moderate risk of bias
Olayemi, 2018	No	Yes	Yes	Not applicable (for animals)	No	No	Yes	Yes	Yes	Yes	Rodents	Moderate risk of bias
Olayemi, 2018	No	Yes	Yes	Not applicable (for animals)	No	No	Yes	Yes	Yes	Yes	Rodents	Moderate risk of bias
Olayemi, 2018	No	Yes	Yes	Not applicable (for animals)	No	No	Yes	Yes	Yes	Yes	Rodents	Moderate risk of bias
Olayemi, 2018	No	Yes	Yes	Not applicable (for animals)	No	No	Yes	Yes	Yes	Yes	Rodents	Moderate risk of bias
Oloniniyi, 2018	No	Yes	No	Yes	Yes	No	Yes	Yes	Yes	Yes	Humans	Low risk of bias
Omilabu, 2005	No	Yes	No	Yes	No	No	Yes	Yes	No	Yes	Humans	Moderate risk of bias
Omilabu, 2005	No	Yes	No	Yes	No	No	Yes	Yes	No	Yes	Humans	Moderate risk of bias
Omilabu, 2005	No	Yes	No	Yes	No	No	Yes	Yes	No	Yes	Humans	Moderate risk of bias
Omilabu, 2005	No	Yes	No	Yes	No	No	Yes	Yes	No	Yes	Humans	Moderate risk of bias
Omilabu, 2005	No	Yes	No	Yes	No	No	Yes	Yes	No	Yes	Humans	Moderate risk of bias
Omilabu, 2005	No	Yes	No	Yes	No	No	Yes	Yes	No	Yes	Humans	Moderate risk of bias

Omilabu, 2005	No	Yes	No	Yes	No	No	Yes	Yes	No	Yes	Humans	Moderate risk of bias
Omilabu, 2005	No	Yes	No	Yes	No	No	Yes	Yes	No	Yes	Humans	Moderate risk of bias
Omilabu, 2005	No	Yes	No	Yes	No	No	Yes	Yes	No	Yes	Humans	Moderate risk of bias
Paix, 1988	No	Yes	No	Yes	No	No	Yes	Yes	Unclear	Yes	Humans	Moderate risk of bias
Panning, 2010	No	Yes	No	Yes	Yes	No	Yes	Yes	Unclear	Yes	Humans	Moderate risk of bias
Panning, 2010	No	Yes	No	Yes	Yes	No	Yes	Yes	Unclear	Yes	Humans	Moderate risk of bias
Panning, 2010	No	Yes	No	Yes	Yes	No	Yes	Yes	Unclear	Yes	Humans	Moderate risk of bias
Price, 1988	No	Yes	No	Yes	Yes	No	Yes	Yes	Yes	Yes	Humans	Low risk of bias
Price, 1988	No	Yes	No	Yes	Yes	No	Yes	Yes	Yes	Yes	Humans	Low risk of bias
Rodhain, 1989	No	Yes	No	Yes	No	No	Yes	Yes	Yes	Yes	Humans	Moderate risk of bias
Safronetz, 2010	No	Yes	No	Not applicable (for animals)	No	No	Yes	Yes	Yes	Yes	Rodents	Moderate risk of bias
Safronetz, 2010	No	Yes	No	Not applicable (for animals)	No	No	Yes	Yes	Yes	Yes	Rodents	Moderate risk of bias
Safronetz, 2010	No	Yes	No	Not applicable (for animals)	No	No	Yes	Yes	Yes	Yes	Rodents	Moderate risk of bias
Safronetz, 2010	No	Yes	No	Not applicable (for animals)	No	No	Yes	Yes	Yes	Yes	Rodents	Moderate risk of bias
Safronetz, 2010	No	Yes	No	Not applicable (for animals)	No	No	Yes	Yes	Yes	Yes	Rodents	Moderate risk of bias
Safronetz, 2010	No	Yes	No	Not applicable (for animals)	No	No	Yes	Yes	Yes	Yes	Rodents	Moderate risk of bias
Safronetz, 2013	No	Yes	No	Not applicable (for animals)	No	No	Yes	Yes	Yes	Yes	Rodents	Moderate risk of bias
Safronetz, 2013	No	Yes	No	Not applicable (for animals)	No	No	Yes	Yes	Yes	Yes	Rodents	Moderate risk of bias
Safronetz, 2013	No	Yes	No	Not applicable (for animals)	No	No	Yes	Yes	Yes	Yes	Rodents	Moderate risk of bias
Safronetz, 2013	No	Yes	No	Not applicable (for animals)	No	No	Yes	Yes	Yes	Yes	Rodents	Moderate risk of bias
Salu, 2019	No	Yes	No	Yes	No	No	Yes	Yes	Yes	Yes	Humans	Moderate risk of bias
Saluzzo, 1988	No	Yes	No	Not applicable (for animals)	No	No	Yes	Yes	Unclear	Yes	Rodents	Moderate risk of bias
Saluzzo, 1988	No	Yes	No	Not applicable (for animals)	No	No	Yes	Yes	Unclear	Yes	Rodents	Moderate risk of bias
Saluzzo, 1988	No	Yes	No	Not applicable (for animals)	No	No	Yes	Yes	Unclear	Yes	Rodents	Moderate risk of bias
Saluzzo, 1988	No	Yes	No	Not applicable (for animals)	No	No	Yes	Yes	Unclear	Yes	Rodents	Moderate risk of bias
Saluzzo, 1988	No	Yes	No	Not applicable (for animals)	No	No	Yes	Yes	Unclear	Yes	Rodents	Moderate risk of bias
Schoepp, 2014	No	Yes	No	Yes	Yes	No	Yes	No	Yes	Yes	Humans	Moderate risk of bias
Schoepp, 2014	No	Yes	No	Yes	Yes	No	Yes	No	Yes	Yes	Humans	Moderate risk of bias
Shaffer, 2019	No	Yes	No	Yes	Yes	No	Yes	Yes	Yes	Yes	Humans	Low risk of bias
Shehu, 2018	No	Yes	No	Yes	Yes	No	Yes	Yes	Yes	Yes	Humans	Low risk of bias
Smith, 1979	No	Yes	No	Yes	No	No	Yes	Yes	Unclear	Yes	Humans	Moderate risk of bias
Smith, 1979	No	Yes	No	Yes	No	No	Yes	Yes	Unclear	Yes	Humans	Moderate risk of bias
Sogoba, 2016	No	Yes	No	Yes	Yes	No	Yes	Yes	Yes	Yes	Humans	Low risk of bias
Sogoba, 2016	No	Yes	No	Yes	Yes	No	Yes	Yes	Yes	Yes	Humans	Low risk of bias
Ter Meulen, 1994	No	Yes	Yes	Yes	Yes	No	Yes	Yes	Yes	Yes	Humans	Low risk of bias
Ter Meulen, 1998	No	Yes	No	Yes	Yes	No	Yes	Yes	Yes	Yes	Humans	Low risk of bias
Ter Meulen, 1998	No	Yes	No	Yes	Yes	No	Yes	Yes	Yes	Yes	Humans	Low risk of bias
Ter Meulen, 1998	No	Yes	No	Yes	Yes	No	Yes	Yes	Yes	Yes	Humans	Low risk of bias
Ter Meulen, 1998	No	Yes	No	Yes	Yes	No	Yes	Yes	Yes	Yes	Humans	Low risk of bias
Tomori, 1988	No	Yes	No	Yes	No	No	Yes	Yes	Unclear	Yes	Humans	Moderate risk of bias
Troup, 1970	No	Yes	No	Yes	No	No	Yes	Yes	No	Yes	Humans	Moderate risk of bias
Troup, 1970	No	Yes	No	Yes	No	No	Yes	Yes	No	Yes	Humans	Moderate risk of bias
Van der Waals, 1986	No	Yes	No	Yes	No	No	Yes	No	Unclear	No	Humans	High risk of bias
Webb, 1986	No	Yes	No	Yes	Yes	No	Yes	Yes	Yes	Yes	Humans	Low risk of bias
Webb, 1986	No	Yes	No	Yes	Yes	No	Yes	Yes	Yes	Yes	Humans	Low risk of bias
Wulff, 1975	No	Yes	No	Not applicable (for animals)	No	No	Yes	Yes	Yes	Yes	Rodents	Moderate risk of bias
Wulff, 1975	No	Yes	No	Not applicable (for animals)	No	No	Yes	Yes	Yes	Yes	Rodents	Moderate risk of bias
Wulff, 1975	No	Yes	No	Not applicable (for animals)	No	No	Yes	Yes	Yes	Yes	Rodents	Moderate risk of bias
Wulff, 1975	No	Yes	No	Not applicable (for animals)	No	No	Yes	Yes	Yes	Yes	Rodents	Moderate risk of bias
Yadouleton, 2019	No	Yes	No	Not applicable (for animals)	Yes	No	Yes	Yes	Yes	Yes	Rodents	Moderate risk of bias
Yadouleton, 2019	No	Yes	No	Not applicable (for animals)	Yes	No	Yes	Yes	Yes	Yes	Rodents	Moderate risk of bias
Yadouleton, 2019	No	Yes	No	Not applicable (for animals)	Yes	No	Yes	Yes	Yes	Yes	Rodents	Moderate risk of bias
Yadouleton, 2019	No	Yes	No	Not applicable (for animals)	Yes	No	Yes	Yes	Yes	Yes	Rodents	Moderate risk of bias
Yadouleton, 2019	No	Yes	No	Not applicable (for animals)	Yes	No	Yes	Yes	Yes	Yes	Rodents	Moderate risk of bias
Yalley-Ogunro, 1984	No	Yes	No	Yes	No	No	Yes	Yes	Yes	Yes	Humans	Moderate risk of bias

Reference

1. Fisher-Hoch SP, Tomori O, Nasidi A, Perez-Oronoz GI, Fakile Y, Hutwagner L, et al. Review of cases of nosocomial Lassa fever in Nigeria: the high price of poor medical practice. BMJ. 1995;311: 857–859.

2. Kouadio L, Nowak K, Akoua-Koffi C, Weiss S, Allali BK, Witkowski PT, et al. Lassa Virus in Multimammate Rats, Côte d'Ivoire, 2013. Emerg Infect Dis. 2015;21: 1481–1483. doi:10.3201/eid2108.150312

3. Lecompte E, Fichet-Calvet E, Daffis S, Koulémou K, Sylla O, Kourouma F, et al. Mastomys natalensis and Lassa Fever, West Africa. Emerg Infect Dis. 2006;12: 1971–1974. doi:10.3201/eid1212.060812

4. Monath TP, Newhouse VF, Kemp GE, Setzer HW, Cacciapuoti A. Lassa virus isolation from Mastomys natalensis rodents during an epidemic in Sierra Leone. Science. 1974;185: 263–265. doi:10.1126/science.185.4147.263

5. Olayemi A, Cadar D, Magassouba N, Obadare A, Kourouma F, Oyeyiola A, et al. New Hosts of The Lassa Virus. Sci Rep. 2016;6: 25280. doi:10.1038/srep25280

6. Monath TP, Mertens PE, Patton R, Moser CR, Baum JJ, Pinneo L, et al. A hospital epidemic of Lassa fever in Zorzor, Liberia, March-April 1972. Am J Trop Med Hyg. 1973;22: 773–779. doi:10.4269/ajtmh.1973.22.773

7. Agbonlahor DE, Erah A, Agba IM, Oviasogie FE, Ehiaghe AF, Wankasi M, et al. Prevalence of Lassa virus among rodents trapped in three South-South States of Nigeria. J Vector Borne Dis. 2017; 5.

8. Akhuemokhan OC, Ewah-Odiase RO, Akpede N, Ehimuan J, Adomeh DI, Odia I, et al. Prevalence of Lassa Virus Disease (LVD) in Nigerian children with fever or fever and convulsions in an endemic area. PLoS Negl Trop Dis. 2017;11. doi:10.1371/journal.pntd.0005711

9. Akoua-Koffi C, Ter Meulen J, Legros D, Akran V, Aïdara M, Nahounou N, et al. [Detection of anti-Lassa antibodies in the Western Forest area of the Ivory Coast]. Med Trop (Mars). 2006;66: 465–468.

10. Arnold RB, Gary GW. A neutralization test survey for Lassa fever activity in Lassa, Nigeria. Transactions of the Royal Society of Tropical Medicine and Hygiene. 1977;71: 152–154. doi:10.1016/0035-9203(77)90085-2

11. Asogun DA, Adomeh DI, Ehimuan J, Odia I, Hass M, Gabriel M, et al. Molecular Diagnostics for Lassa Fever at Irrua Specialist Teaching Hospital, Nigeria: Lessons Learnt from Two Years of Laboratory Operation. PLoS Negl Trop Dis. 2012;6. doi:10.1371/journal.pntd.0001839

12. Babalola SO, Babatunde JA, Remilekun OM, Amaobichukwu AR, Abiodun AM, Jide I, et al. Lassa virus RNA detection from suspected cases in Nigeria, 2011-2017. Pan Afr Med J. 2019;34. doi:10.11604/pamj.2019.34.76.16425

13. Bajani MD, Tomori O, Rollin PE, Harry TO, Bukbuk ND, Wilson L, et al. A survey for antibodies to Lassa virus among health workers in Nigeria. Transactions of the Royal Society of Tropical Medicine and Hygiene. 1997;91: 379–381. doi:10.1016/S0035-9203(97)90247-9

14. Baumann J, Knüpfer M, Ouedraogo J, Traoré BY, Heitzer A, Kané B, et al. Lassa and Crimean-Congo Hemorrhagic Fever Viruses, Mali. Emerging Infect Dis. 2019;25: 999–1002. doi:10.3201/eid2505.181047

15. Bausch DG, Demby AH, Coulibaly M, Kanu J, Goba A, Bah A, et al. Lassa fever in Guinea: I. Epidemiology of human disease and clinical observations. Vector Borne Zoonotic Dis. 2001;1: 269–281. doi:10.1089/15303660160025903

16. Blackburn NK, Searle L, Taylor P. Viral haemorrhagic fever antibodies in Zimbabwe schoolchildren. Transactions of the Royal Society of Tropical Medicine and Hygiene. 1982;76: 803–805. doi:10.1016/0035-9203(82)90113-4

17. Boiro I, Lomonossov NN, Sotsinski VA, Constantinov OK, Tkachenko EA, Inapogui AP, et al. [Clinico-epidemiologic and laboratory research on hemorrhagic fevers in Guinea]. Bull Soc Pathol Exot Filiales. 1987;80: 607–612.

18. Bonney JHK, Osei-Kwasi M, Adiku TK, Barnor JS, Amesiya R, Kubio C, et al. Hospital-Based Surveillance for Viral Hemorrhagic Fevers and Hepatitides in Ghana. Kasper M, editor. PLoS Negl Trop Dis. 2013;7: e2435. doi:10.1371/journal.pntd.0002435

19. Bowen GS, Wulff H, Casals J, Noonan A, Downs WG. Lassa fever in Onitsha, East Central State, Nigeria, in 1974. 1975; 6.

20. Branco LM, Grove JN, Boisen ML, Shaffer JG, Goba A, Fullah M, et al. Emerging trends in Lassa fever: redefining the role of immunoglobulin M and inflammation in diagnosing acute infection. Virol J. 2011;8: 478. doi:10.1186/1743-422X-8-478

21. Buba MI, Dalhat MM, Nguku PM, Waziri N, Mohammad JO, Bomoi IM, et al. Mortality Among Confirmed Lassa Fever Cases During the 2015–2016 Outbreak in Nigeria. Am J Public Health. 2018;108: 262–264. doi:10.2105/AJPH.2017.304186

22. Bukbuk DN, Fukushi S, Tani H, Yoshikawa T, Taniguchi S, Iha K, et al. Development and validation of serological assays for viral hemorrhagic fevers and determination of the prevalence of Rift Valley fever in Borno State, Nigeria. Transactions of The Royal Society of Tropical Medicine and Hygiene. 2014;108: 768–773. doi:10.1093/trstmh/tru163

23. Carey DE, Kemp GE, White HA, Pinneo L, Addy RF, Fom ALMD, et al. Lassa fever Epidemiological aspects of the 1970 epidemic, Jos, Nigeria. Trans R Soc Trop Med Hyg. 1972;66: 402–408. doi:10.1016/0035-9203(72)90271-4

24. Clements TL, Rossi CA, Irish AK, Kibuuka H, Eller LA, Robb ML, et al. Chikungunya and O’nyong-nyong Viruses in Uganda: Implications for Diagnostics. Open Forum Infectious Diseases. 2019;6. doi:10.1093/ofid/ofz001

25. Coulibaly-N’Golo D, Allali B, Rieger T, Akoua-Koffi C. Novel Arenavirus Sequences in Hylomyscus sp. and Mus (Nannomys) setulosus from Coˆ te d’Ivoire: Implications for Evolution of Arenaviruses in Africa. PLoS ONE. 2011;6: 9.

26. Dan-Nwafor CC, Ipadeola O, Smout E, Ilori E, Adeyemo A, Umeokonkwo C, et al. A cluster of nosocomial Lassa fever cases in a tertiary health facility in Nigeria: Description and lessons learned, 2018. International Journal of Infectious Diseases. 2019;83: 88–94. doi:10.1016/j.ijid.2019.03.030

27. Dahmane A, van Griensven J, Van Herp M, Van den Bergh R, Nzomukunda Y, Prior J, et al. Constraints in the diagnosis and treatment of Lassa Fever and the effect on mortality in

hospitalized children and women with obstetric conditions in a rural district hospital in Sierra Leone. Trans R Soc Trop Med Hyg. 2014;108: 126–132. doi:10.1093/trstmh/tru009

28. Demartini JC, Green DE, Monath TP. Lassa virus infection in Mastomys natalensis in Sierra Leone. 1975; 12.

29. Demby AH, Inapogui A, Kargbo K, Koninga J, Kourouma K, Kanu J, et al. Lassa fever in Guinea: II. Distribution and prevalence of Lassa virus infection in small mammals. Vector Borne Zoonotic Dis. 2001;1: 283–297. doi:10.1089/15303660160025912

30. Demby AH, Chamberlain J, Brown DW, Clegg CS. Early diagnosis of Lassa fever by reverse transcription-PCR. Journal of Clinical Microbiology. 1994;32: 2898–2903. doi:10.1128/JCM.32.12.2898-2903.1994

31. Ehichioya DU, Asogun DA, Ehimuan J, Okokhere PO, Pahlmann M, Ölschläger S, et al. Hospital-based surveillance for Lassa fever in Edo State, Nigeria, 2005-2008: Lassa fever in Edo State, Nigeria. Tropical Medicine & International Health. 2012;17: 1001–1004. doi:10.1111/j.1365-3156.2012.03010.x

32. Emmerich P, Günther S, Schmitz H. Strain-specific antibody response to Lassa virus in the local population of west Africa. Journal of Clinical Virology. 2008;42: 40–44. doi:10.1016/j.jcv.2007.11.019

33. Emmerich P, Thome-Bolduan C, Drosten C, Gunther S, Ban E, Sawinsky I, et al. Reverse ELISA for IgG and IgM antibodies to detect Lassa virus infections in Africa. Journal of Clinical Virology. 2006;37: 277–281. doi:10.1016/j.jcv.2006.08.015

34. Fabiyi A, Tomori O, Pinneo P. Lassa fever antibodies in hospital personnel in the Plateau State of Nigeria. Niger Med J. 1979;9: 23–25.

35. Fabiyi A. Use of the complement fixation (CF) test in Lassa fever surveillance. 1975; 4.

36. Fair J, Jentes E, Inapogui A, Kourouma K, Goba A, Bah A, et al. Lassa Virus-Infected Rodents in Refugee Camps in Guinea: A Looming Threat to Public Health in a Politically Unstable Region. Vector-Borne and Zoonotic Diseases. 2007;7: 167–171. doi:10.1089/vbz.2006.0581

37. Fichet-Calvet E, Becker-Ziaja B, Koivogui L, Günther S. Lassa Serology in Natural Populations of Rodents and Horizontal Transmission. Vector-Borne and Zoonotic Diseases. 2014;14: 665–674. doi:10.1089/vbz.2013.1484

38. Fichet-Calvet E, Lecompte E, Koivogui L, Soropogui B, Doré A, Kourouma F, et al. Fluctuation of Abundance and Lassa Virus Prevalence in *Mastomys natalensis* in Guinea, West Africa. Vector-Borne and Zoonotic Diseases. 2007;7: 119–128. doi:10.1089/vbz.2006.0520

39. Fisher-Hoch S, McCormick JB, Sasso D, Craven RB. Hematologic dysfunction in Lassa fever. J Med Virol. 1988;26: 127–135. doi:10.1002/jmv.1890260204

40. Frame JD, Jahrling PB, Yalley-Ogunro JE, Monson MH. Endemic Lassa fever in Liberia. II. Serological and virological findings in hospital patients. Trans R Soc Trop Med Hyg. 1984;78: 656–660. doi:10.1016/0035-9203(84)90232-3

41. Frame JD, Casals J, Dennis EA. Lassa virus antibodies in hospital personnel in western Liberia. Transactions of the Royal Society of Tropical Medicine and Hygiene. 1979;73: 219–224. doi:10.1016/0035-9203(79)90218-9

42. Georges AJ, Gonzalez JP, Abdul-Wahid S, Saluzzo JF, Meunier DMY, McCormick JB. Antibodies to Lassa and lassa-like viruses in man and mammals in the Central African Republic. Transactions of the Royal Society of Tropical Medicine and Hygiene. 1985;79: 78–79. doi:10.1016/0035-9203(85)90242-1

43. Gonzalez JP, Josse R, Johnson ED, Merlin M, Georges AJ, Abandja J, et al. Antibody prevalence against haemorrhagic fever viruses in randomized representative central African populations. Research in Virology. 1989;140: 319–331. doi:10.1016/S0923-2516(89)80112-8

44. Hamblion EL, Raftery P, Wendland A, Dweh E, Williams GS, George RNC, et al. The challenges of detecting and responding to a Lassa fever outbreak in an Ebola-affected setting. International Journal of Infectious Diseases. 2018;66: 65–73. doi:10.1016/j.ijid.2017.11.007

45. Haun BK, Kamara V, Dweh AS, Garalde-Machida K, Forkay SSE, Takaaze M, et al. Serological evidence of Ebola virus exposure in dogs from affected communities in Liberia: A preliminary report. Rimoin AW, editor. PLoS Negl Trop Dis. 2019;13: e0007614. doi:10.1371/journal.pntd.0007614

46. Helmick C, Scribner C, Webb P, Krebs J, Mccormick J. NO EVIDENCE FOR INCREASED RISK OF LASSA FEVER INFECTION IN HOSPITAL STAFF. The Lancet. 1986;328: 1202–1205. doi:10.1016/S0140-6736(86)92206-3

47. Henderson BE, Gary GW, Kissling RE, Frame JD, Carey DE. Lassa fever virological and serological studies. Transactions of the Royal Society of Tropical Medicine and Hygiene. 1972;66: 409–416. doi:10.1016/0035-9203(72)90272-6

48. Ibekwe T, Nwegbu M, Okokhere P, Adomeh D, Asogun D. The sensitivity and specificity of Lassa virus IgM by ELISA as screening tool at early phase of Lassa fever infection. Niger Med J. 2012;53: 196. doi:10.4103/0300-1652.107552

49. Ipadeola O, Furuse Y, Ilori EA, Dan-Nwafor CC, Akabike KO, Ahumibe A, et al. Epidemiology and case-control study of Lassa fever outbreak in Nigeria from 2018 to 2019. Journal of Infection. 2020; S0163445320300013. doi:10.1016/j.jinf.2019.12.020

50. Isere EE, Fatiregun A, Ilesanmi O, Ijarotimi I, Egube B, Adejugbagbe A, et al. Lessons Learnt from Epidemiological Investigation of Lassa Fever Outbreak in a Southwest State of Nigeria December 2015 to April 2016. PLoS Curr. 2018 [cited 25 Feb 2020]. doi:10.1371/currents.outbreaks.bc4396a6650d0ed1985d731583bf5ded

51. Ivanoff’ B, Duquesnoy’ P, Languillat’ G, Saluzzo’ JF, Georges’ A, Gonzalez’ JP, et al. Haemorrhagic fever in Gabon. I. Incidence of Lassa, Ebola and Marburg viruses in Haut-Ogoou6. 1982; 2.

52. Jahrling PB, Frame JD, Smith SB, Monson MH. Endemic Lassa fever in Liberia. III. Characterization of Lassa virus isolates. Transactions of the Royal Society of Tropical Medicine and Hygiene. 1985;79: 374–379. doi:10.1016/0035-9203(85)90386-4

53. Johnson BK, Ocheng D, Gichogo A, Okiro M, Libondo D, Tukei PM, et al. Antibodies against haemorrhagic fever viruses in Kenya populations. Transactions of the Royal Society of Tropical Medicine and Hygiene. 1983;77: 731–733. doi:10.1016/0035-9203(83)90216-X

54. Johnson BK, Ocheng D, Gitau LG, Gichogo A, Tukei PM, Ngindu A, et al. Viral Haemorrhagic Fever Surveillance in Kenya, 1980-198. 1983; 1.

55. Johnson' BK, Gitau LG, Gichogop A, Tukei' M, Else' JG, Suleman MA, et al. Marburg, Ebota and Rift Valley fever virus antibodies in East African primates. Transactions of the Royal Society of Tropical Medicine and Hygiene. 1982; 4.

56. Keane E, Gilles HM. Lassa fever in Panguma Hospital, Sierra Leone, 1973-6. BMJ. 1977;1: 1399–1402. doi:10.1136/bmj.1.6073.1399

57. Kernéis S, Koivogui L, Magassouba N, Koulemou K, Lewis R, Aplogan A, et al. Prevalence and Risk Factors of Lassa Seropositivity in Inhabitants of the Forest Region of Guinea: A Cross-Sectional Study. Aksoy S, editor. PLoS Negl Trop Dis. 2009;3: e548. doi:10.1371/journal.pntd.0000548

58. Klempa B, Koulemou K, Auste B, Emmerich P, Thomé-Bolduan C, Günther S, et al. Seroepidemiological study reveals regional co-occurrence of Lassa- and Hantavirus antibodies in Upper Guinea, West Africa. Trop Med Int Health. 2012; n/a-n/a. doi:10.1111/tmi.12045

59. Lalis A, Leblois R, Lecompte E, Denys C, ter Meulen J, Wirth T. The Impact of Human Conflict on the Genetics of Mastomys natalensis and Lassa Virus in West Africa. Mores CN, editor. PLoS ONE. 2012;7: e37068. doi:10.1371/journal.pone.0037068

60. Leski TA, Stockelman MG, Moses LM, Park M, Stenger DA, Ansumana R, et al. Sequence Variability and Geographic Distribution of Lassa Virus, Sierra Leone - Volume 21, Number 4—April 2015 - Emerging Infectious Diseases journal - CDC. 2015 [cited 26 Oct 2019]. doi:10.3201/eid2104.141469

61. Li W-G, Chen W-W, Li L, Ji D, Ji Y-J, Li C, et al. The etiology of Ebola virus disease-like illnesses in Ebola virusnegative patients from Sierra Leone. Oncotarget. 2016;7. doi:10.18632/oncotarget.8558

62. Lukashevich IS, Clegg JC, Sidibe K. Lassa virus activity in Guinea: distribution of human antiviral antibody defined using enzyme-linked immunosorbent assay with recombinant antigen. J Med Virol. 1993;40: 210–217. doi:10.1002/jmv.1890400308

63. Maigari IM, Jibrin YB, Umar MS, Lawal SM, Gandi AY. Descriptive features of Lassa fever in Bauchi, Northeastern Nigeria - a retrospective review. Research Journal of Health Sciences. 2018;6: 149. doi:10.4314/rejhs.v6i3.7

64. Mariën J, Borremans B, Gryseels S, Soropogui B, De Bruyn L, Bongo GN, et al. No measurable adverse effects of Lassa, Morogoro and Gairo arenaviruses on their rodent reservoir host in natural conditions. Parasites Vectors. 2017;10: 210. doi:10.1186/s13071-017-2146-0

65. Mathiot CC, Fontenille D, Georges AJ, Coulanges P. Antibodies to haemorrhagic fever viruses in Madagascar populations. Transactions of the Royal Society of Tropical Medicine and Hygiene. 1989;83: 407–409. doi:10.1016/0035-9203(89)90519-1

66. McCarthy MC, Haberberger RL, Salib AW, Soliman BA, El-Tigani A, Watts DM. Evaluation of arthropod-borne viruses and other infectious disease pathogens as the causes of febrile illnesses in the Khartoum Province of Sudan. 1996; 6.

67. McCormick JB, King IJ, Webb PA, Johnson KM, O'Sullivan R, Smith ES, et al. A Case-Control Study of the Clinical Diagnosis and Course of Lassa Fever. J Infect Dis. 1987;155: 445–455. doi:10.1093/infdis/155.3.445

68. McCormick JB, Webb PA, Krebs JW, Johnson KM, Smith ES. A Prospective Study of the Epidemiology and Ecology of Lassa Fever. J Infect Dis. 1987;155: 437–444. doi:10.1093/infdis/155.3.437

69. Meunier DM, Johnson ED, Gonzalez JP, Georges-Courbot MC, Madelon MC, Georges AJ. [Current serologic data on viral hemorrhagic fevers in the Central African Republic]. Bull Soc Pathol Exot Filiales. 1987;80: 51–61.

70. Nakounné E, Selekon B, Morvan J. Veille microbiologique : les fièvres hémorragiques virales en République centrafricaine ; 2000; 8.

71. Nimo-Paintsil SC, Fichet-Calvet E, Borremans B, Letizia AG, Mohareb E, Bonney JHK, et al. Rodent-borne infections in rural Ghanaian farming communities. Schieffelin J, editor. PLoS ONE. 2019;14: e0215224. doi:10.1371/journal.pone.0215224

72. Ogunro BN, Olugasa BO, Kayode A, Ishola OO, Kolawole ON, Odigie EA, et al. Detection of Antibody and Antigen for Lassa Virus Nucleoprotein in Monkeys from Southern Nigeria. JEGH. 2019 [cited 25 Feb 2020]. doi:10.2991/jegh.k.190421.001

73. O'Hearn AE, Voorhees MA, Fetterer DP, Wauquier N, Coomber MR, Bangura J, et al. Serosurveillance of viral pathogens circulating in West Africa. Virol J. 2016;13: 163. doi:10.1186/s12985-016-0621-4

74. Okogbenin S, Okoeguale J, Akpede G, Colubri A, Barnes KG, Mehta S, et al. Retrospective Cohort Study of Lassa Fever in Pregnancy, Southern Nigeria. Emerg Infect Dis. 2019;25: 1494–1500. doi:10.3201/eid2508.181299

75. Okokhere P, Colubri A, Azubike C, Iruolagbe C, Osazuwa O, Tabrizi S, et al. Clinical and laboratory predictors of Lassa fever outcome in a dedicated treatment facility in Nigeria: a retrospective, observational cohort study. The Lancet Infectious Diseases. 2018;18: 684–695. doi:10.1016/S1473-3099(18)30121-X

76. Okoror LE, Esumeh FI, Agbonlahor DE, Umolu PI. Lassa virus: Seroepidemiological survey of rodents caught in Ekpoma and environs. Trop Doct. 2005;35: 16–17. doi:10.1258/0049475053001912

77. Olayemi A, Oyeyiola A, Obadare A, Igbokwe J, Adesina AS, Onwe F, et al. Widespread arenavirus occurrence and seroprevalence in small mammals, Nigeria. Parasites Vectors. 2018;11: 416. doi:10.1186/s13071-018-2991-5

78. Olayemi A, Obadare A, Oyeyiola A, Igbokwe J, Fasogbon A, Igbahenah F, et al. Arenavirus Diversity and Phylogeography of *Mastomys natalensis* Rodents, Nigeria. Emerg Infect Dis. 2016;22: 687–690. doi:10.3201/eid2204.150155

79. Oloniniyi OK, Unigwe US, Okada S, Kimura M, Koyano S, Miyazaki Y, et al. Genetic characterization of Lassa virus strains isolated from 2012 to 2016 in southeastern Nigeria. PLoS Negl Trop Dis. 2018;12. doi:10.1371/journal.pntd.0006971

80. Omilabu SA, Badaru SO, Okokhere P, Asogun D, Drosten C, Emmerich P, et al. Lassa Fever, Nigeria, 2003 and 2004. Emerg Infect Dis. 2005;11: 1642–1644. doi:10.3201/eid1110.041343

81. Paix MA, Poveda JD, Malvy D, Bailly C, Merlin M, Fleury HJ. [Serological study of the virus responsible for hemorrhagic fever in an urban population of Cameroon]. Bull Soc Pathol Exot Filiales. 1988;81: 679–682.

82. Panning M, Emmerich P, Ölschläger S, Bojenko S, Koivogui L, Marx A, et al. Laboratory Diagnosis of Lassa Fever, Liberia. Emerg Infect Dis. 2010;16: 1041–1043. doi:10.3201/eid1606.100040

83. Price ME, Fisher-Hoch SP, Craven RB, McCormick JB. A prospective study of maternal and fetal outcome in acute Lassa fever infection during pregnancy. 1988;297: 4.

84. Rodhain F, Gonzalez JP, Mercier E, Helynck B, Larouze B, Hannoun C. Arbovirus infections and viral haemorrhagic fevers in Uganda: a serological survey in Karamoja district, 1984. Transactions of the Royal Society of Tropical Medicine and Hygiene. 1989;83: 851–854. doi:10.1016/0035-9203(89)90352-0

85. Safronetz D, Sogoba N, Lopez JE, Maiga O, Dahlstrom E, Zivcec M, et al. Geographic Distribution and Genetic Characterization of Lassa Virus in Sub-Saharan Mali. PLoS Negl Trop Dis. 2013;7. doi:10.1371/journal.pntd.0002582

86. Safronetz D, Lopez JE, Sogoba N, Traore' SF, Raffel SJ, Fischer ER, et al. Detection of Lassa virus, Mali. Emerging Infect Dis. 2010;16: 1123–1126. doi:10.3201/eid1607.100146

87. Salu OB, James AB, Bankolé HS, Agbla JM, Da Silva M, Gbaguidi F, et al. Molecular confirmation and phylogeny of Lassa fever virus in Benin Republic 2014–2016. African Journal of Laboratory Medicine. 2019;8. doi:10.4102/ajlm.v8i1.803

88. Saluzzo JF, Adam F, McCormick JB, Digoutte JP. Lassa Fever Virus in Senegal. Journal of Infectious Diseases. 1988;157: 605–605. doi:10.1093/infdis/157.3.605

89. Schoepp RJ, Rossi CA, Khan SH, Goba A, Fair JN. Undiagnosed Acute Viral Febrile Illnesses, Sierra Leone. Emerg Infect Dis. 2014;20: 1176–1182. doi:10.3201/eid2007.131265

90. Shaffer JG, Schieffelin JS, Gbakie M, Alhasan F, Roberts NB, Goba A, et al. A medical records and data capture and management system for Lassa fever in Sierra Leone: Approach, implementation, and challenges. Verdonck K, editor. PLoS ONE. 2019;14: e0214284. doi:10.1371/journal.pone.0214284

91. Shehu NY, Gomerep SS, Isa SE, Iraoyah KO, Mafuka J, Bitrus N, et al. Lassa Fever 2016 Outbreak in Plateau State, Nigeria—The Changing Epidemiology and Clinical Presentation. Front Public Health. 2018;6: 232. doi:10.3389/fpubh.2018.00232

92. Smith EA, Fabiyi A, Kuteyi OE, Tomori O. Epidemiological aspect of the 1976 Pankshin Lassa fever outbreak. Niger Med J. 1979;9: 20–22.

93. Sogoba N, Rosenke K, Adjemian J, Diawara SI, Maiga O, Keita M, et al. Lassa Virus Seroprevalence in Sibirilia Commune, Bougouni District, Southern Mali. Emerg Infect Dis. 2016;22: 657–663. doi:10.3201/eid2204.151814

94. ter Meulen J, Koulemou K, Wittekindt T, Windisch K, Strigl S, Conde S, et al. Detection of Lassa Virus Antinucleoprotein Immunoglobulin G (IgG) and IgM Antibodies by a Simple Recombinant Immunoblot Assay for Field Use. Journal of Clinical Microbiology. 1998;36: 3143–3148. doi:10.1128/JCM.36.11.3143-3148.1998

95. Tomori O, Fabiyi A, Sorungbe A, Smith A, McCormick JB. Viral hemorrhagic fever antibodies in Nigerian populations. Am J Trop Med Hyg. 1988;38: 407–410. doi:10.4269/ajtmh.1988.38.407

96. Van der Waals FW, Pomeroy KL, Goudsmit J, Asher DM, Gajdusek DC. Hemorrhagic fever virus infections in an isolated rainforest area of central Liberia. Limitations of the indirect immunofluorescence slide test for antibody screening in Africa. Trop Geogr Med. 1986;38: 209–214.

97. Webb PA, McCormick JB, King IJ, Bosman I, Johnson KM, Elliott LH, et al. Lassa fever in children in Sierra Leone, West Africa. Trans R Soc Trop Med Hyg. 1986;80: 577–582. doi:10.1016/0035-9203(86)90147-1

98. Wulff H, Fabiyi A, Monath TP. Recent isolations of Lassa virus from Nigerian rodents. Bull World Health Organ. 1975;52: 609–613.

99. Yadouleton A, Agolinou A, Kourouma F, Saizonou R, Pahlmann M, Bedié SK, et al. Lassa Virus in Pygmy Mice, Benin, 2016–2017. Emerg Infect Dis. 2019;25: 1977–1979. doi:10.3201/eid2510.180523

100. Yalley-Ogunro JE, Frame JD, Hanson AP. Endemic Lassa fever in Liberia. VI. Village serological surveys for evidence of Lassa virus activity in Lofa County, Liberia. Transactions of the Royal Society of Tropical Medicine and Hygiene. 1984;78: 764–770. doi:10.1016/0035-9203(84)90013-0

101. Cummins D, McCormick JB, Bennett D, Samba JA, Farrar B, Machin SJ, et al. Acute sensorineural deafness in Lassa fever. JAMA. 1990;264: 2093–2096.

102. Frame JD, Yalley-Ogunro JE, Hanson AP. Endemic Lassa fever in Liberia. V. Distribution of Lassa virus activity in Liberia: hospital staff surveys. Transactions of the Royal Society of Tropical Medicine and Hygiene. 1984;78: 761–763. doi:10.1016/0035-9203(84)90012-9

103. Gonzalez JP, McCormick JB, Saluzzo JF, Herve JP, Georges AJ, Johnson KM. An arenavirus isolated from wild-caught rodents (Pramys species) in the Central African Republic. Intervirology. 1983;19: 105–112. doi:10.1159/000149344

104. Keenlyside RA, McCormick JB, Webb PA, Smith E, Elliott L, Johnson KM. Case-control study of Mastomys natalensis and humans in Lassa virus-infected households in Sierra Leone. Am J Trop Med Hyg. 1983;32: 829–837. doi:10.4269/ajtmh.1983.32.829

105. Meulen JT, Lukashevich I, Sidibe K, Inapogui A, Marx M, Dorlemann A, et al. Hunting of Peridomestic Rodents and Consumption of Their Meat as Possible Risk Factors for Rodent-to-Human Transmission of Lassa Virus in the Republic of Guinea. The American Journal of Tropical Medicine and Hygiene. 1996;55: 661–666. doi:10.4269/ajtmh.1996.55.661

106. Troup JM, White HA, Fom AL, Carey DE. An outbreak of Lassa fever on the Jos plateau, Nigeria, in January-February 1970. A preliminary report. Am J Trop Med Hyg. 1970;19: 695–696. doi:10.4269/ajtmh.1970.19.695

107. Bloch A. A serological survey of Lassa fever in Liberia. 1978; 3.

108. Dan-Nwafor CC, Furuse Y, Ilori EA, Ipadeola O, Akabike KO, Ahumibe A, et al. Measures to control protracted large Lassa fever outbreak in Nigeria, 1 January to 28 April 2019. Eurosurveillance. 2019;24. doi:10.2807/1560-7917.ES.2019.24.20.1900272

109. Johnson ED, Gonzalez JP, Georges A. Haemorrhagic fever virus activity in equatorial Africa: distribution and prevalence of filovirus reactive antibody in the Central African Republic. Transactions of the Royal Society of Tropical Medicine and Hygiene. 1993;87: 530–535. doi:10.1016/0035-9203(93)90075-2
