# Supplementary material for: Systematic review and meta-analysis of the epidemiology of Lassa virus in humans, rodents and other mammals in sub-Saharan Africa
Source: PLoS Negl Trop Dis. 2020 Aug 26;14(8):e0008589. doi: 10.1371/journal.pntd.0008589 (PMC7478710; doi:10.1371/journal.pntd.0008589)
Supplement: S6 Table — (PDF) [file pntd.0008589.s006.pdf]

S6 Table: Characteristics of included studies

**Characteristics**	**Humans (159)**	**Rodents (83)**	**Other Mammals (16)**
Year of publication; range	1970-2020	1972-2019	1972-2019
Period of inclusion of participants; range	1965-2019	1972-2016	1972-2018
Study Design			
- Cross-sectional	127 (79.9)	115 (99.1	16 (100.0)
- Hospital outbreak	17 (10.7)		
- Case-control	6 (3.8)	1 (0.9)	
- Community outbreak	7 (4.4)		
- Cohort (Baseline data)	2 (1.3)		
Sampling			
- Non probabilistic	148 (93.1)	87 (75.0	14 (87.5)
- Probabilistic	11 (6.9)	29 (25.0	2 (12.5)
Sampling method			
- Consecutive sampling	103 (64.8)		8 (50.0)
- Trapping		116 (100.0)	4 (25.0)
- Convenience sampling	45 (28.3)		4 (25.0)
- Cluster sampling	8 (5.0)		
- Simple random sampling	2 (1.3)		
- Systematic sampling	1 (0.6)		
Number of sites			
- Multicenter	111 (69.8)	108 (93.1)	14 (87.5)
- Monocenter	47 (29.6)	8 (6.9)	2 (12.5)
- Unclear/Not reported	1 (0.6)		
Timing of data collection			
- Prospetively	129 (81.1)	113 (97.4)	15 (93.8)
- Retrospectively	29 (18.2)	3 (2.6)	1 (6.3)
- Unclear/Not reported	1 (0.6)		
UNSD Region			
- West Africa	130 (81.8)	113 (97.4)	9 (56.3)
- Central Africa	18 (11.3)	3 (2.6)	6 (37.5)
- Eastern Africa	10 (6.3)		1 (6.3)
- Northern Africa	1 (0.6)		
Country			
- Nigeria	51 (32.1)	34 (29.3)	6 (37.5)
- Sierra Leone	29 (18.2)	9 (7.8)	2 (12.5)
- Liberia	19 (12.0)		1 (6.3)
- Guinea	11 (6.9)	28 (24.1)	
- Central African Republic	7 (4.4)	2 (1.7)	5 (31.3)
- Guinea. Mali		14 (12.1)	
- Senegal		5 (4.3)	
- Guinea. Liberia	4 (2.5)		
- Kenya	4 (2.5)		1 (6.3)
- Mali	4 (2.5)	10 (8.6)	
- Cameroon	3 (1.9)	1 (0.9)	
- Gabon	3 (1.9)		1 (6.3)
- Benin	2 (1.3)	5 (4.3)	
- Democratic Republic of the Congo	2 (1.3)		
- Ghana	2 (1.3)		
- Guinea. Ghana	2 (1.3)		
- Guinea. Ivory Coast. Ghana. Benin. Nigeria	2 (1.3)		
- Ivory Coast	2 (1.3)	8 (6.9)	
- Uganda	2 (1.3)		

- Burkina Faso	1 (0.6)		
- Chad	1 (0.6)		
- Equatorial Guinea	1 (0.6)		
- Ethiopia	1 (0.6)		
- Madagascar	1 (0.6)		
- Niger	1 (0.6)		
- Republic of the Congo	1 (0.6)		
- Sudan	1 (0.6)		
- Tanzania	1 (0.6)		
- Zimbabwe	1 (0.6)		
%Male. Range	0-100	NA	NA
Age (years); Median [IQR]	29.1 [25.7-34.0]	NA	NA
Age range			
- < 5 years			1 (6.3)
- < 15 years	3 (1.9)		
- < 50 Years	13 (8.2)		
- All ages	26 (16.4)		
- Unclear/ Not reported	117 (73.6)	116 (100.0)	15 (93.8)
Recrutment setting			
- Rural	62 (39.0)	37 (31.9)	7 (43.8)
- Urban	25 (15.7)	6 (5.2)	4 (25.0)
- Urban/rural	12 (7.6)	31 (26.7)	2 (12.5)
- Unclear/Not reported	60 (37.7)	42 (36.2)	3 (18.8)
Setting			
- Hospital-based	63 (39.6)		
- Community-based	60 (37.7)	116 (100.0)	16 (100.0)
- Hospital/community based	29 (18.2)		
- Unclear/Not reported	7 (4.4)		
Hospitalization			
- Ambulatory	48 (30.2)		
- Hospitalized	39 (24.5)		
- Hospitalized/ambulatory	4 (2.5)		
- Not applicable (if not in the hospital)	54 (34.0)	116 (100.0)	16 (100.0)
- Unclear/Not reported	14 (8.8)		
Detection assay			
- Indirect immunofluorescence assay	58 (36.5)	33 (28.5)	8 (50.0)
- Classical RT-PCR	23 (14.5)	47 (40.5)	1 (6.3)
- Indirect ELISA	19 (12.0)	5 (4.3)	2 (12.5)
- Neutralization test	19 (12.0)		
- Complement fixation test	16 (10.1)	4 (3.5)	1 (6.3)
- Immunoflorescent assay, Culture	4 (2.5)		
- Culture	3 (1.9)	8 (6.9)	2 (12.5)
- Direct ELISA	3 (1.9)	4 (3.5)	1 (6.3)
- Indirect ELISA, Direct ELISA	2 (1.3)		
- PCR and sequencing	2 (1.3)		
- Real-time PCR	2 (1.3)	13 (11.2)	
- Classical RT-PCR, Direct ELISA	1 (0.6)		
- Classical RT-PCR, Rapid Diagnostic Test. Indirect ELISA, Direct ELISA	1 (0.6)		
- Complement fixation test, Immunoflorescent assay	1 (0.6)		
- Immunoblot Assay	1 (0.6)		
- Lateral flow immunoassays	1 (0.6)		
- Luminex Mag- Pix	1 (0.6)		
- Radioimmunoassay		1 (0.9)	
- RPM-TEI Microarray Analysis		1 (0.9)	
- Microsphere immunoassay			1 (6.3)

- Unclear/Not reported	2 (1.3)		
Target detected			
- Antibodies	69 (43.4)	14 (12.1)	8 (50.0)
- IgG	27 (17.0)	28 (24.1)	3 (18.8)
- RNA	27 (17.0)	61 (52.6)	1 (6.3)
- IgM	11 (6.9)		1 (6.3)
- IgM and IgG	9 (5.7)		
- Antigen	5 (3.1)	5 (4.3)	1 (6.3)
- IgG, IgM, Virus	4 (2.5)		
- Virus	3 (1.9)	8 (6.9)	2 (12.5)
- Antigen, IgM	2 (1.3)		
- RNA, Antigen	1 (0.6)		
- RNA, Antigen, IgM, IgG	1 (0.6)		
Sample types			
- Serum	143 (89.9)	69 (59.5)	13 (81.3)
- Serum, spleen fluid, liver fluid		14 (12.1)	
- Serum, Urine, Organ tissue		4 (3.5)	
- Pharyngeal, Serum, Urine	3 (1.9)		
- Organ tissue		3 (2.6)	1 (6.3)
- Spleen sections		3 (2.6)	
- Serum, urine, and occasionally cerebrospinal fluid	2 (1.3)		
- Serum, Urine, Organ tissue, ectoparasites		2 (1.7)	2 (12.5)
- Lung		2 (1.7)	
- Liver		2 (1.7)	
- Serum and/or homogenized spleen		6 (5.2)	
- Serum, Organ tissue		1 (0.9)	
- Liver and spleens		1 (0.9)	
- Cerebrospinal fluid, Serum	1 (0.6)		
- Serum, Urine	1 (0.6)		
- Serum, Urine, Organ tissue, lung, heart, spleen, kidney, bladder, urine, and ectoparasites		1 (0.9)	
- Broncho-alveolar		1 (0.9)	
- Serum and organ samples (kidney, liver, spleen)		1 (0.9)	
- Unclear/Not reported	9 (5.7)	1 (0.9)	
Infection Status			
- At least one past contact	96 (60.4)	42 (36.2)	11 (68.8)
- Current contact	43 (27.0)	74 (63.8)	4 (25.0)
- Recent contact	20 (12.6)		1 (6.3)
Rodent genotyping			
- Yes		81 (69.8)	
- No		13 (11.2)	
- Not applicable	159 (100.0)		16 (100.0)
- Unclear/ Not reported		22 (19.0)	
Risk of bias			
- High risk of bias	1 (0.6)		
- Moderate risk of bias	112 (70.4)	116 (100.0)	16 (100.0)
- Low risk of bias	46 (29.0)		
Humans			
- Apparently healthy individuals	57 (35.9)		
- LASV suspected cases	36 (22.6)		
- Febrile patients	23 (14.5)		
- Healthcare workers	19 (12.0)		
- LASV positive case contact	4 (2.5)		
- Patient with any illness	4 (2.5)		
- High risk individuals, people with regular animals contact	3 (1.9)		

- Patient with hemorrhagic fever	3 (1.9)		
- Patient with illnesses other than fever diseases	3 (1.9)		
- Apparently healthy individuals, Patient with any illness	3 (1.9)		
- Pregnant women	2 (1.3)		
- Blood donors	1 (0.6)		
- LASV Confirmed Cases	1 (0.6)		
Rodents			
- Unspecified rodents		39 (33.6)	
- Mastomys natalensis		13 (11.2)	
- Mastomys erythroleucus		7 (6.0)	
- Mastomys species		7 (6.0)	
- Rattus rattus		6 (5.2)	
- Praomys daltoni		5 (4.3)	
- Mus baoulei		3 (2.6)	
- Mus minutoides		3 (2.6)	
- Mus setulosus		3 (2.6)	
- Crocodura species		2 (1.7)	
- Gerbilliscus kempi		2 (1.7)	
- Lemniscomys species		2 (1.7)	
- Lemniscomys striatus		2 (1.7)	
- Lophuromys sikapusi		2 (1.7)	
- Myomys daltoni		2 (1.7)	
- Praomys jacksoni		2 (1.7)	
- Praomys rostratus		2 (1.7)	
- Uranomys ruddi		2 (1.7)	
- Crocidura buettikoferi		1 (0.9)	
- Gerbilliscus guineae		1 (0.9)	
- Gerbilliscus species		1 (0.9)	
- Hylomyscus pamfi		1 (0.9)	
- Lemniscomys bellieri/zebra		1 (0.9)	
- Lemniscomys striatus/linulus		1 (0.9)	
- Mus (Nannomys) species		1 (0.9)	
- Mus mattheyi		1 (0.9)	
- Nannomys minutoides/mattheyi		1 (0.9)	
- Praomys cf. rostratus		1 (0.9)	
- Praomys species		1 (0.9)	
- Tatera cf. guinea		1 (0.9)	
Other Mammals			
- Monkeys			3 (18.8)
- Bats			2 (12.5)
- Dog			2 (12.5)
- Insectivora. Crocidura spp.. Erinaceus albiventris			2 (12.5)
- Primates			2 (12.5)
- Cattle			1 (6.3)
- Goat			1 (6.3)
- Guinea-pig			1 (6.3)
- Insectivora. Crocidura spp.			1 (6.3)
- Sheep			1 (6.3)
