# Supplementary material for: Systematic review and meta-analysis of the epidemiology of Lassa virus in humans, rodents and other mammals in sub-Saharan Africa
Source: PLoS Negl Trop Dis. 2020 Aug 26;14(8):e0008589. doi: 10.1371/journal.pntd.0008589 (PMC7478710; doi:10.1371/journal.pntd.0008589)
Supplement: S7 Table — (PDF) [file pntd.0008589.s007.pdf]

S7 Table: Individual characteristics of included studies

Author, Year	Study Design	Sampling	Sampling method	Number of sites	Timing of data collection	Country	Study period	Mean or Median age	Age range	Gender	Recrutment setting	Setting	Type of hospital	Hospitalization	Study participants clinical case definition	LASV suspected case definition	LASV probable case definition	LASV confirmed case definition	Detection assay	Sample types	Target detected	Infection Status	species	Population characteristics	Rodent species identification approach	Rodent genotyping
Agbonlahor, 2017	Cross-sectional	Non probabilistic	Trapping	Multicenter	Prospetively	Nigeria	Nov/2015-Oct/2016	Unclear/ Not reported	Unclear/ Not reported	Unclear/ Not reported	Unclear/Not reported	Community-based	Not applicable (if not in the hospital)	Not applicable (if not in the hospital)	Not applicable (for animals and healty participants)	Not applicable (for animals and healty participants)	Not applicable (for animals and healty participants)	Unclear/Not reported	Classical RT-PCR	Serum	RNA	Current infection	Rodents	Unspecified rodents	Morphological characteristics such as colour, length of rodent, length of tail and body weight were used to identify trapped rodents as described by Fichet-Hoch et al12.	No
Agbonlahor, 2017	Cross-sectional	Non probabilistic	Trapping	Multicenter	Prospetively	Nigeria	Nov/2015-Oct/2016	Unclear/ Not reported	Unclear/ Not reported	Unclear/ Not reported	Unclear/Not reported	Community-based	Not applicable (if not in the hospital)	Not applicable (if not in the hospital)	Not applicable (for animals and healty participants)	Not applicable (for animals and healty participants)	Not applicable (for animals and healty participants)	Unclear/Not reported	Real-time PCR	Serum	RNA	Current infection	Rodents	Unspecified rodents	Morphological characteristics such as colour, length of rodent, length of tail and body weight were used to identify trapped rodents as described by Fichet-Hoch et al12.	No
Agbonlahor, 2017	Cross-sectional	Non probabilistic	Trapping	Multicenter	Prospetively	Nigeria	Nov/2015-Oct/2016	Unclear/ Not reported	Unclear/ Not reported	Unclear/ Not reported	Unclear/Not reported	Community-based	Not applicable (if not in the hospital)	Not applicable (if not in the hospital)	Not applicable (for animals and healty participants)	Not applicable (for animals and healty participants)	Not applicable (for animals and healty participants)	Unclear/Not reported	Real-time PCR	Serum	RNA	Current infection	Rodents	Unspecified rodents	Morphological characteristics such as colour, length of rodent, length of tail and body weight were used to identify trapped rodents as described by Fichet-Hoch et al12.	No
Agbonlahor, 2017	Cross-sectional	Non probabilistic	Trapping	Multicenter	Prospetively	Nigeria	Nov/2015-Oct/2016	Unclear/ Not reported	Unclear/ Not reported	Unclear/ Not reported	Unclear/Not reported	Community-based	Not applicable (if not in the hospital)	Not applicable (if not in the hospital)	Not applicable (for animals and healty participants)	Not applicable (for animals and healty participants)	Not applicable (for animals and healty participants)	Unclear/Not reported	Real-time PCR	Serum	RNA	Current infection	Rodents	Unspecified rodents	Morphological characteristics such as colour, length of rodent, length of tail and body weight were used to identify trapped rodents as described by Fichet-Hoch et al12.	No
Akhuemokhan, 2017	Case-control	Non probabilistic	Consecutive sampling	Monocenter	Prospetively	Nigeria	Dec/2009-Nov/2010	Unclear/ Not reported	0.1-15	58,5	Urban	Hospital-based	Emergency department	Hospitalized	For the purpose of this study, a 'Case' was defined as a child aged ≥1month to ≤15 years, with temperature ≥38°C on admission or a history of fever + temperature ≥38°C within 8 hours of admission, and a history of convulsions during the illness. A 'Control' met the same criteria but had no history of convulsions.	Unclear/Not reported	Unclear/Not reported	A positive LASV-RT-PCR test was taken as confirmatory of the diagnosis of LVD [48].	Classical RT-PCR	Serum	RNA	Current infection	Humans	Febrile patients	Not applicable	Not applicable
Akoua-Koffi, 2006	Cross-sectional	Non probabilistic	Consecutive sampling	Multicenter	Prospetively	Ivory Coast	Mar/2002	37.5	20-65	100	Unclear/Not reported	Hospital/community based	Health Centers	Ambulatory	Not applicable (for animals and healty participants)	Not applicable (for animals and healty participants)	Not applicable (for animals and healty participants)	Les puits dont au moins 5 cellules infectées présentent une fl u o rescence ve rt e gra nu l a i re de la nu cl é o p rotéine intra - cytoplasmique cara ctéristique ont été considérés comme positifs	Indirect immunofluorescence assay	Serum	IgG	At least one past contact	Humans	High risk individuals, people with regular animals contact	Not applicable	Not applicable
Arnold, 1977	Cross-sectional	Non probabilistic	Convenience sampling	Multicenter	Prospetively	Nigeria	Aug/1970	Unclear/ Not reported	Unclear/ Not reported	Unclear/ Not reported	Rural	Hospital/community based	Unclear/Not reported	Ambulatory	Not applicable (for animals and healty participants)	Not applicable (for animals and healty participants)	Not applicable (for animals and healty participants)	Sera were considered positive if they met these criteria:	Neutralization test	Serum	Antibodies	At least one past contact	Humans	Apparently healthy individuals	Not applicable	Not applicable
Arnold, 1977	Cross-sectional	Non probabilistic	Convenience sampling	Multicenter	Prospetively	Nigeria	Mar/1971	Unclear/ Not reported	Unclear/ Not reported	Unclear/ Not reported	Rural	Hospital/community based	Unclear/Not reported	Ambulatory	Not applicable (for animals and healty participants)	Not applicable (for animals and healty participants)	Not applicable (for animals and healty participants)	Sera were considered positive if they met these criteria:	Neutralization test	Serum	Antibodies	At least one past contact	Humans	Apparently healthy individuals	Not applicable	Not applicable
Asogun, 2012	Hospital outbreak	Non probabilistic	Convenience sampling	Monocenter	Prospetively	Nigeria	Jun/2009-Dec/2010	28	Unclear/ Not reported	50	Unclear/Not reported	Hospital-based	Tertiary hospital	Hospitalized	Unclear/Not reported	The following case definition, taking into account published signs and symptoms of Lassa fever, was used as a guideline for identifying suspected cases and requesting molecular testing for Lassa virus (from the diagnostic laboratory request form): 1. Patients with: a. 38uC fever for at least 2 days b. excluded typhoid fever and malaria negative or just 1+ in thick smear c. and some or one of the following symptoms: chest pain, sore throat, headache, muscle pain, vomiting, and diarrhea. 2. Or: patients with fever who show bleeding or facial edema. 3. Or: patients with fever who do not respond to anti-malarials or antibiotics after 2 days of treatment. 4. Or: patients with fever who had contact with a confirmed Lassa fever case within the last three weeks.	Unclear/Not reported	Unclear/Not reported	Classical RT-PCR	Serum	RNA	Current infection	Humans	LASV suspected cases	Not applicable	Not applicable
Babalola, 2019	Cross-sectional	Non probabilistic	Consecutive sampling	Multicenter	Retrospectively	Nigeria	2011-2017	Unclear/ Not reported	Unclear/ Not reported	43,4	Urban	Hospital-based	Unclear/Not reported	Hospitalized	Unclear/Not reported	Unclear/Not reported	Unclear/Not reported	Unclear/Not reported	Classical RT-PCR	Serum	RNA	Current infection	Humans	LASV suspected cases	Not applicable	Not applicable
Bajani, 1997	Cross-sectional	Non probabilistic	Consecutive sampling	Multicenter	Retrospectively	Nigeria	Dec/1992-Mar/1993	Unclear/ Not reported	Unclear/ Not reported	Unclear/ Not reported	Urban	Hospital-based	Primary hospital, Secondary hospital, Tertiary hospital	Ambulatory	Not applicable (for animals and healty participants)	Not applicable (for animals and healty participants)	Not applicable (for animals and healty participants)	The cut-off values defining a positive result in both assays were set as the mean of the adjusted ODs for all dilutions of negative reference sera in a series of tests, plus 3 standard deviations. The IgG ELISA cut-off value was an adjusted OD >0.2, and for the IgM assay was an adjusted OD >O. 1.	Indirect ELISA	Serum	IgM	Recent infection	Humans	Healthcare workers	Not applicable	Not applicable
Bajani, 1997	Cross-sectional	Non probabilistic	Consecutive sampling	Multicenter	Retrospectively	Nigeria	Dec/1992-Mar/1993	Unclear/ Not reported	Unclear/ Not reported	Unclear/ Not reported	Urban	Hospital-based	Primary hospital, Secondary hospital, Tertiary hospital	Ambulatory	Not applicable (for animals and healty participants)	Not applicable (for animals and healty participants)	Not applicable (for animals and healty participants)	The cut-off values defining a positive result in both assays were set as the mean of the adjusted ODs for all dilutions of negative reference sera in a series of tests, plus 3 standard deviations. The IgG ELISA cut-off value was an adjusted OD >0.2, and for the IgM assay was an adjusted OD >O. 1.	Indirect ELISA	Serum	IgG	At least one past contact	Humans	Healthcare workers	Not applicable	Not applicable
Baumann, 2019	Cross-sectional	Non probabilistic	Consecutive sampling	Monocenter	Prospetively	Mali	Apr/2016–May/2017	Unclear/ Not reported	0.4-14	Unclear/ Not reported	Urban	Hospital-based	Unclear/Not reported	Hospitalized	Unclear/Not reported	Unclear/Not reported	Unclear/Not reported	Unclear/Not reported	Classical RT-PCR	Serum	RNA	Current infection	Humans	Febrile patients	Not applicable	Not applicable
Bausch, 2001	Cross-sectional	Non probabilistic	Consecutive sampling	Multicenter	Prospetively	Guinea	Mar/1996-Dec/1999	29	0-50	46	Rural	Hospital-based	Unclear/Not reported	Hospitalized	Unclear/Not reported	Patients were enrolled who presented to one of the surveillance hospitals with a febrile illness compatible with LF (see below) from March 1996 through December 1999. Owing to political as well as logistic complications, enrollment was not continuous during this period, and surveillance activity was more consistent in some hospitals than others. Enrollment criteria were a temperature of $38°C lasting for at least 1 week that was not associated with trauma and did not respond to appropriate antimicrobial therapy for malaria or typhoid fever. In addition, individuals who presented with signs and symptoms deemed typical for LF, such as sore throat with retrosternal pain, bleeding, edema, sudden hearing loss, or febrile spontaneous abortion, were often enrolled even if their fever was less than a week in duration.	Unclear/Not reported	Unclear/Not reported	Indirect ELISA	Serum	IgG	At least one past contact	Humans	LASV suspected cases	Not applicable	Not applicable
Blackburn, 1982	Cross-sectional	Non probabilistic	Consecutive sampling	Multicenter	Prospetively	Zimbabwe	1980	Unclear/ Not reported	8-10	Unclear/ Not reported	Unclear/Not reported	Community-based	Not applicable (if not in the hospital)	Not applicable (if not in the hospital)	Not applicable (for animals and healty participants)	Not applicable (for animals and healty participants)	Not applicable (for animals and healty participants)	The degree of specific fluorescence was recorded as + (suspicious) + (positive) and + + (strongly positive).	Indirect immunofluorescence assay	Serum	Antibodies	At least one past contact	Humans	Apparently healthy individuals	Not applicable	Not applicable
Boiro, 1987	Cross-sectional	Non probabilistic	Trapping	Multicenter	Prospetively	Guinea	Mar/1982-Aug/1982, Jan/1983	Unclear/ Not reported	Unclear/ Not reported	Unclear/ Not reported	Rural	Community-based	Not applicable (if not in the hospital)	Not applicable (if not in the hospital)	Not applicable (for animals and healty participants)	Not applicable (for animals and healty participants)	Not applicable (for animals and healty participants)	Unclear/Not reported	Radioimmunoassay	Serum	Antigen	Current infection	Rodents	Unspecified rodents	Unclear/ Not reported	Unclear/ Not reported
Boiro, 1987	Community outbreak	Non probabilistic	Convenience sampling	Multicenter	Prospetively	Guinea	Mar/1982-Aug/1982, Jan/1983	Unclear/ Not reported	Unclear/ Not reported	Unclear/ Not reported	Rural	Hospital-based	Unclear/Not reported	Hospitalized/ambulatory	Unclear/Not reported	Unclear/Not reported	Unclear/Not reported	Unclear/Not reported	Indirect immunofluorescence assay	Serum	Antibodies	At least one past contact	Humans	Febrile patients	Not applicable	Not applicable
Bonney, 2013	Cross-sectional	Non probabilistic	Consecutive sampling	Multicenter	Prospetively	Ghana	2008-2011	23.5	Unclear/ Not reported	Unclear/ Not reported	Unclear/Not reported	Hospital-based	Unclear/Not reported	Ambulatory	Unclear/Not reported	Unclear/Not reported	Unclear/Not reported	Unclear/Not reported	Classical RT-PCR	Serum	RNA	Current infection	Humans	Febrile patients	Not applicable	Not applicable
Bowen, 1975	Hospital outbreak	Non probabilistic	Convenience sampling	Multicenter	Prospetively	Nigeria	Jan/1974-Feb/1974	Unclear/ Not reported	Unclear/ Not reported	Unclear/ Not reported	Unclear/Not reported	Hospital/community based	Unclear/Not reported	Ambulatory	Not applicable (for animals and healty participants)	Not applicable (for animals and healty participants)	Not applicable (for animals and healty participants)	Unclear/Not reported	Complement fixation test	Serum	Antibodies	At least one past contact	Humans	Healthcare workers	Not applicable	Not applicable
Bowen, 1975	Cross-sectional	Non probabilistic	Trapping	Multicenter	Prospetively	Nigeria	Jan/1974-Feb/1974	Unclear/ Not reported	Unclear/ Not reported	Unclear/ Not reported	Unclear/Not reported	Community-based	Not applicable (if not in the hospital)	Not applicable (if not in the hospital)	Not applicable (for animals and healty participants)	Not applicable (for animals and healty participants)	Not applicable (for animals and healty participants)	Unclear/Not reported	Complement fixation test	Serum and organ samples (kidney, liver, spleen)	Antibodies	At least one past contact	Rodents	Unspecified rodents	Unclear/ Not reported	Unclear/ Not reported
Branco, 2011	Cross-sectional	Non probabilistic	Consecutive sampling	Monocenter	Prospetively	Sierra Leone	Apr/2010	Unclear/ Not reported	Unclear/ Not reported	Unclear/ Not reported	Unclear/Not reported	Hospital/community based	Unclear/Not reported	Ambulatory	Not applicable (for animals and healty participants)	Not applicable (for animals and healty participants)	Not applicable (for animals and healty participants)	Patients were considered to have acute LF if a reaction above background levels developed on reLASV LFI modules and/or LASV Ag-capture ELISA, and/or LASV-specific RT-PCR generated a GPC gene-specific amplicon using oligonucleotides previously described by Olschläger et al. [13].	Direct ELISA	Serum	Antigen	Current infection	Humans	Apparently healthy individuals	Not applicable	Not applicable
Branco, 2011	Cross-sectional	Non probabilistic	Consecutive sampling	Monocenter	Prospetively	Sierra Leone	Apr/2010	Unclear/ Not reported	Unclear/ Not reported	Unclear/ Not reported	Unclear/Not reported	Hospital/community based	Unclear/Not reported	Ambulatory	Not applicable (for animals and healty participants)	Not applicable (for animals and healty participants)	Not applicable (for animals and healty participants)	Unclear/Not reported	Indirect ELISA	Serum	IgM	Recent infection	Humans	Apparently healthy individuals	Not applicable	Not applicable
Branco, 2011	Cross-sectional	Non probabilistic	Consecutive sampling	Monocenter	Prospetively	Sierra Leone	Apr/2010	Unclear/ Not reported	Unclear/ Not reported	Unclear/ Not reported	Unclear/Not reported	Hospital/community based	Unclear/Not reported	Ambulatory	Not applicable (for animals and healty participants)	Not applicable (for animals and healty participants)	Not applicable (for animals and healty participants)	Unclear/Not reported	Indirect ELISA	Serum	IgG	At least one past contact	Humans	Apparently healthy individuals	Not applicable	Not applicable
Branco, 2011	Cross-sectional	Non probabilistic	Consecutive sampling	Monocenter	Retrospectively	Sierra Leone	2007-2011	Unclear/ Not reported	Unclear/ Not reported	Unclear/ Not reported	Unclear/Not reported	Hospital/community based	Unclear/Not reported	Ambulatory	Not applicable (for animals and healty participants)	Not applicable (for animals and healty participants)	Not applicable (for animals and healty participants)	Patients were considered to have acute LF if a reaction above background levels developed on reLASV LFI modules and/or LASV Ag-capture ELISA, and/or LASV-specific RT-PCR generated a GPC gene-specific amplicon using oligonucleotides previously described by Olschläger et al. [13].	Lateral flow immunoassays	Serum	Antigen	Current infection	Humans	LASV suspected cases	Not applicable	Not applicable
Branco, 2011	Cross-sectional	Non probabilistic	Consecutive sampling	Monocenter	Retrospectively	Sierra Leone	2007-2011	Unclear/ Not reported	Unclear/ Not reported	Unclear/ Not reported	Unclear/Not reported	Hospital/community based	Unclear/Not reported	Ambulatory	Not applicable (for animals and healty participants)	Not applicable (for animals and healty participants)	Not applicable (for animals and healty participants)	Unclear/Not reported	Indirect ELISA	Serum	IgM	Recent infection	Humans	LASV suspected cases	Not applicable	Not applicable
Buba, 2018	Community outbreak	Non probabilistic	Convenience sampling	Multicenter	Retrospectively	Nigeria	2015-2016	Unclear/ Not reported	Unclear/ Not reported	30	Unclear/Not reported	Community-based	Not applicable (if not in the hospital)	Not applicable (if not in the hospital)	Unclear/Not reported	We defined suspected (patient had symptoms) and confirmed (reverse transcription polymerase chain reaction or enzyme-linked immunosorbent assay confirmed patient was infected with Lassa virus) cases on the basis of Technical Guidelines for Integrated Disease.	Unclear/Not reported	We defined suspected (patient had symptoms) and confirmed (reverse transcription polymerase chain reaction or enzyme-linked immunosorbent assay confirmed patient was infected with Lassa virus) cases on the basis of Technical Guidelines for Integrated Disease.	Classical RT-PCR, Direct ELISA	Unclear/Not reported	RNA, Antigen	Current infection	Humans	LASV Confirmed Cases	Not applicable	Not applicable
Bukbuk, 2014	Cross-sectional	Probabilistic	Simple random sampling	Multicenter	Prospetively	Nigeria	Sep/2011-Feb/2012	Unclear/ Not reported	Unclear/ Not reported	Unclear/ Not reported	Unclear/Not reported	Hospital-based	Clinic	Ambulatory	Unclear/Not reported	Unclear/Not reported	Unclear/Not reported	Unclear/Not reported	Indirect ELISA	Serum	IgG	At least one past contact	Humans	Febrile patients	Not applicable	Not applicable
Carey, 1972	Cross-sectional	Non probabilistic	Convenience sampling	Multicenter	Retrospectively	Nigeria	Jan/1970-Feb/1970, May/1970, 1966	Unclear/ Not reported	Unclear/ Not reported	Unclear/ Not reported	Unclear/Not reported	Community-based	Not applicable (if not in the hospital)	Not applicable (if not in the hospital)	Not applicable (for animals and healty participants)	Not applicable (for animals and healty participants)	Not applicable (for animals and healty participants)	Unclear/Not reported	Complement fixation test	Tissues	Antibodies	At least one past contact	Other mammals	Bats	Not applicable	Not applicable
Carey, 1972	Cross-sectional	Non probabilistic	Trapping	Multicenter	Prospetively	Nigeria	Jan/1970-Feb/1970, May/1970, 1966	Unclear/ Not reported	Unclear/ Not reported	Unclear/ Not reported	Unclear/Not reported	Community-based	Not applicable (if not in the hospital)	Not applicable (if not in the hospital)	Not applicable (for animals and healty participants)	Not applicable (for animals and healty participants)	Not applicable (for animals and healty participants)	Unclear/Not reported	Complement fixation test	Tissues	Antibodies	At least one past contact	Rodents	Unspecified rodents	Unclear/ Not reported	Unclear/ Not reported
Carey, 1972	Community outbreak	Non probabilistic	Convenience sampling	Multicenter	Prospetively	Nigeria	Jan/1970-Feb/1970, May/1970, 1966	Unclear/ Not reported	Unclear/ Not reported	Unclear/ Not reported	Unclear/Not reported	Community-based	Unclear/Not reported	Ambulatory	Unclear/Not reported	Unclear/Not reported	Unclear/Not reported	Unclear/Not reported	Complement fixation test	Serum	Antibodies	At least one past contact	Humans	LASV positive case contact	Not applicable	Not applicable
Carey, 1972	Hospital outbreak	Non probabilistic	Convenience sampling	Multicenter	Prospetively	Nigeria	Jan/1970-Feb/1970, May/1970, 1966	Unclear/ Not reported	Unclear/ Not reported	Unclear/ Not reported	Unclear/Not reported	Community-based	Unclear/Not reported	Ambulatory	Unclear/Not reported	Unclear/Not reported	Unclear/Not reported	Unclear/Not reported	Complement fixation test	Serum	Antibodies	At least one past contact	Humans	LASV positive case contact	Not applicable	Not applicable
Carey, 1972	Hospital outbreak	Non probabilistic	Convenience sampling	Multicenter	Prospetively	Nigeria	Jan/1970-Feb/1970, May/1970, 1966	Unclear/ Not reported	Unclear/ Not reported	Unclear/ Not reported	Unclear/Not reported	Hospital/community based	Unclear/Not reported	Hospitalized	Unclear/Not reported	Unclear/Not reported	Unclear/Not reported	Unclear/Not reported	Complement fixation test	Unclear/Not reported	Antibodies	At least one past contact	Humans	LASV suspected cases	Not applicable	Not applicable
Clements, 2019	Cross-sectional	Non probabilistic	Consecutive sampling	Multicenter	Prospetively	Uganda	2006-2007	Unclear/ Not reported	Unclear/ Not reported	Unclear/ Not reported	Unclear/Not reported	Unclear/Not reported	Not applicable (if not in the hospital)	Not applicable (if not in the hospital)	Not applicable (for animals and healty participants)	Not applicable (for animals and healty participants)	Not applicable (for animals and healty participants)	Unclear/Not reported	Indirect ELISA	Serum	IgG	At least one past contact	Humans	Apparently healthy individuals	Not applicable	Not applicable
Coulibaly-N'Golo, 2011	Cross-sectional	Non probabilistic	Trapping	Multicenter	Prospetively	Ivory Coast	2003-2005	Unclear/ Not reported	Unclear/ Not reported	Unclear/ Not reported	Unclear/Not reported	Community-based	Not applicable (if not in the hospital)	Not applicable (if not in the hospital)	Not applicable (for animals and healty participants)	Not applicable (for animals and healty participants)	Not applicable (for animals and healty participants)	Unclear/Not reported	Classical RT-PCR	Serum	RNA	Current infection	Rodents	Rattus rattus	Animals were taxonomically classified according to morphological criteria (weight, length of head and body, length of tail, length of hind food, length of the ear, color etc.) and dissected in the field.	Yes
Coulibaly-N'Golo, 2011	Cross-sectional	Non probabilistic	Trapping	Multicenter	Prospetively	Ivory Coast	2003-2005	Unclear/ Not reported	Unclear/ Not reported	Unclear/ Not reported	Unclear/Not reported	Community-based	Not applicable (if not in the hospital)	Not applicable (if not in the hospital)	Not applicable (for animals and healty participants)	Not applicable (for animals and healty participants)	Not applicable (for animals and healty participants)	Unclear/Not reported	Classical RT-PCR	Serum	RNA	Current infection	Rodents	Praomys daltoni	Animals were taxonomically classified according to morphological criteria (weight, length of head and body, length of tail, length of hind food, length of the ear, color etc.) and dissected in the field.	Yes
Coulibaly-N'Golo, 2011	Cross-sectional	Non probabilistic	Trapping	Multicenter	Prospetively	Ivory Coast	2003-2005	Unclear/ Not reported	Unclear/ Not reported	Unclear/ Not reported	Unclear/Not reported	Community-based	Not applicable (if not in the hospital)	Not applicable (if not in the hospital)	Not applicable (for animals and healty participants)	Not applicable (for animals and healty participants)	Not applicable (for animals and healty participants)	Unclear/Not reported	Classical RT-PCR	Serum	RNA	Current infection	Rodents	Mastomys natalensis	Animals were taxonomically classified according to morphological criteria (weight, length of head and body, length of tail, length of hind food, length of the ear, color etc.) and dissected in the field.	Yes
Coulibaly-N'Golo, 2011	Cross-sectional	Non probabilistic	Trapping	Multicenter	Prospetively	Ivory Coast	2003-2005	Unclear/ Not reported	Unclear/ Not reported	Unclear/ Not reported	Unclear/Not reported	Community-based	Not applicable (if not in the hospital)	Not applicable (if not in the hospital)	Not applicable (for animals and healty participants)	Not applicable (for animals and healty participants)	Not applicable (for animals and healty participants)	Unclear/Not reported	Classical RT-PCR	Serum	RNA	Current infection	Rodents	Gerbilliscus species	Animals were taxonomically classified according to morphological criteria (weight, length of head and body, length of tail, length of hind food, length of the ear, color etc.) and dissected in the field.	Yes
Coulibaly-N'Golo, 2011	Cross-sectional	Non probabilistic	Trapping	Multicenter	Prospetively	Ivory Coast	2003-2005	Unclear/ Not reported	Unclear/ Not reported	Unclear/ Not reported	Unclear/Not reported	Community-based	Not applicable (if not in the hospital)	Not applicable (if not in the hospital)	Not applicable (for animals and healty participants)	Not applicable (for animals and healty participants)	Not applicable (for animals and healty participants)	Unclear/Not reported	Classical RT-PCR	Serum	RNA	Current infection	Rodents	Lemniscomys species	Animals were taxonomically classified according to morphological criteria (weight, length of head and body, length of tail, length of hind food, length of the ear, color etc.) and dissected in the field.	Yes
Coulibaly-N'Golo, 2011	Cross-sectional	Non probabilistic	Trapping	Multicenter	Prospetively	Ivory Coast	2003-2005	Unclear/ Not reported	Unclear/ Not reported	Unclear/ Not reported	Unclear/Not reported	Community-based	Not applicable (if not in the hospital)	Not applicable (if not in the hospital)	Not applicable (for animals and healty participants)	Not applicable (for animals and healty participants)	Not applicable (for animals and healty participants)	Unclear/Not reported	Classical RT-PCR	Serum	RNA	Current infection	Rodents	Praomys species	Animals were taxonomically classified according to morphological criteria (weight, length of head and body, length of tail, length of hind food, length of the ear, color etc.) and dissected in the field.	Yes
Coulibaly-N'Golo, 2011	Cross-sectional	Non probabilistic	Trapping	Multicenter	Prospetively	Ivory Coast	2003-2005	Unclear/ Not reported	Unclear/ Not reported	Unclear/ Not reported	Unclear/Not reported	Community-based	Not applicable (if not in the hospital)	Not applicable (if not in the hospital)	Not applicable (for animals and healty participants)	Not applicable (for animals and healty participants)	Not applicable (for animals and healty participants)	Unclear/Not reported	Classical RT-PCR	Serum	RNA	Current infection	Rodents	Uranomys ruddi	Animals were taxonomically classified according to morphological criteria (weight, length of head and body, length of tail, length of hind food, length of the ear, color etc.) and dissected in the field.	Yes
Cummins, 1990	Cross-sectional	Non probabilistic	Convenience sampling	Monocenter	Prospetively	Sierra Leone	Jan-Apr/1987	Unclear/ Not reported	Unclear/ Not reported	40,6	Rural	Hospital/community based	Unclear/Not reported	Unclear/Not reported	A case of Lassa fever was defined as a patient with a febrile illness and an eightfold or greater rise in anti-Lassa IgG titer (by immunofluorescent as¬ say), an IgG titer on admission of 256 or greater and a Lassa antigen specific IgM titer (by immunofluorescent assay) of 16 or greater, and/or virus isolation from serum.4	A case of Lassa fever was defined as a patient with a febrile illness and an eightfold or greater rise in anti-Lassa IgG titer (by immunofluorescent as¬ say), an IgG titer on admission of 256 or greater and a Lassa antigen specific IgM titer (by immunofluorescent assay) of 16 or greater, and/or virus isolation from serum.4	Unclear/Not reported	A case of Lassa fever was defined as a patient with a febrile illness and an eightfold or greater rise in anti-Lassa IgG titer (by immunofluorescent assay), an IgG titer on admission of 256 or greater and a Lassa antigen specific IgM titer (by immunofluorescent assay) of 16 or greater, and/or virus isolation from serum.4,14	Immunoflorescent assay, Culture	Serum	IgG, IgM, Virus	Current infection	Humans	LASV suspected cases	Not applicable	Not applicable
Dahmane, 2014	Cross-sectional	Non probabilistic	Consecutive sampling	Monocenter	Retrospectively	Sierra Leone	Apr/2011-Feb/2012	Unclear/ Not reported	Unclear/ Not reported	Unclear/ Not reported	Rural	Hospital-based	Emergency department	Hospitalized	The clinical manifestations of LF are non specific (fever,sore,throat, headache, muscle spain, vomiting) in the early stage of the illness and mimic common conditions like flu and malaria.	A 'suspected' LF case is one that fits this definition without laboratory confirmation (either no laboratory test done or negative laboratory result for LF).	Unclear/Not reported	A confirmed LF case is one which has a positive laboratory test for LF (Lassa antigen or anti-Lassa IgM detected).	Indirect ELISA, Direct ELISA	Serum	Antigen, IgM	Current infection	Humans	LASV suspected cases	Not applicable	Not applicable
Dahmane, 2014	Cross-sectional	Non probabilistic	Consecutive sampling	Monocenter	Retrospectively	Sierra Leone	Apr/2011-Feb/2012	Unclear/ Not reported	Unclear/ Not reported	Unclear/ Not reported	Rural	Hospital-based	Emergency department	Hospitalized	The clinical manifestations of LF are non specific (fever,sore,throat, headache, muscle spain, vomiting) in the early stage of the illness and mimic common conditions like flu and malaria.	A 'suspected' LF case is one that fits this definition without laboratory confirmation (either no laboratory test done or negative laboratory result for LF).	Unclear/Not reported	A confirmed LF case is one which has a positive laboratory test for LF (Lassa antigen or anti-Lassa IgM detected).	Direct ELISA	Serum	Antigen	Current infection	Humans	LASV suspected cases	Not applicable	Not applicable
Dahmane, 2014	Cross-sectional	Non probabilistic	Consecutive sampling	Monocenter	Retrospectively	Sierra Leone	Apr/2011-Feb/2012	Unclear/ Not reported	Unclear/ Not reported	Unclear/ Not reported	Rural	Hospital-based	Emergency department	Hospitalized	The clinical manifestations of LF are non specific (fever,sore,throat, headache, muscle spain, vomiting) in the early stage of the illness and mimic common conditions like flu and malaria.	A 'suspected' LF case is one that fits this definition without laboratory confirmation (either no laboratory test done or negative laboratory result for LF).	Unclear/Not reported	A confirmed LF case is one which has a positive laboratory test for LF (Lassa antigen or anti-Lassa IgM detected).	Indirect ELISA	Serum	IgM	Recent infection	Humans	LASV suspected cases	Not applicable	Not applicable
Dan-Nwafor, 2019	Cross-sectional	Non probabilistic	Convenience sampling	Monocenter	Retrospectively	Nigeria	2018	37.5	20–51	68.8	Unclear/Not reported	Unclear/Not reported	Tertiary hospital	Ambulatory	Not applicable (for animals and healty participants)	Not applicable (for animals and healty participants)	Not applicable (for animals and healty participants)	A confirmed Lassa fever case was any suspected case with laboratory confirmation (positive IgM antibody, PCR or virus isolation).	Real-time PCR	Serum	RNA	Current infection	Humans	Healthcare workers	Not applicable	Not applicable
Dan-Nwafor, 2019	Community outbreak	Non probabilistic	Convenience sampling	Multicenter	Prospetively	Nigeria	Jan/2017-Apr/2019	Unclear/ Not reported	Unclear/ Not reported	Unclear/ Not reported	Unclear/Not reported	Unclear/Not reported	Unclear/Not reported	Unclear/Not reported	Unclear/Not reported	Unclear/Not reported	Unclear/Not reported	Unclear/Not reported	Classical RT-PCR	Serum	RNA	Current infection	Humans	LASV suspected cases	Not applicable	Not applicable
Demartini, 1975	Cross-sectional	Non probabilistic	Trapping	Multicenter	Retrospectively	Sierra Leone	Sep/1972-Oct/1972	Unclear/ Not reported	Unclear/ Not reported	Unclear/ Not reported	Rural	Community-based	Not applicable (if not in the hospital)	Not applicable (if not in the hospital)	Not applicable (for animals and healty participants)	Not applicable (for animals and healty participants)	Not applicable (for animals and healty participants)	Unclear/Not reported	Culture	Serum, Urine, Organ tissue, lung, heart, spleen, kidney, bladder urine and ectoparasites	Virus	Current infection	Rodents	Unspecified rodents	Unclear/ Not reported	Unclear/ Not reported
Demby, 1994	Cross-sectional	Non probabilistic	Consecutive sampling	Multicenter	Prospetively	Sierra Leone	1991	Unclear/ Not reported	Unclear/ Not reported	Unclear/ Not reported	Unclear/Not reported	Community-based	Unclear/Not reported	Ambulatory	Not applicable (for animals and healty participants)	Not applicable (for animals and healty participants)	Not applicable (for animals and healty participants)	Positive samples had a characteristic fluorescence at a >1:16 dilution in phosphate-buffered saline.	Classical RT-PCR	Serum	RNA	Current infection	Humans	Blood donors	Not applicable	Not applicable

Demby, 1994	Cross-sectional	Non probabilistic	Consecutive sampling	Multicenter	Prospetively	Sierra Leone	1991	Unclear/ Not reported	Unclear/ Not reported	Unclear/ Not reported	Unclear/Not reported	Hospital/community based	Unclear/Not reported	Hospitalized	Unclear/Not reported	Unclear/Not reported	Unclear/Not reported	Positive samples had a characteristic fluorescence at a >1:16 dilution in phosphate-buffered saline.	Classical RT-PCR	Serum	RNA	Current infection	Humans	LASV suspected cases	Not applicable	Not applicable
Demby, 1994	Cross-sectional	Non probabilistic	Consecutive sampling	Multicenter	Prospetively	Sierra Leone	1991	Unclear/ Not reported	Unclear/ Not reported	Unclear/ Not reported	Unclear/Not reported	Hospital/community based	Unclear/Not reported	Hospitalized	Unclear/Not reported	Unclear/Not reported	Unclear/Not reported	Positive samples had a characteristic fluorescence at a >1:16 dilution in phosphate-buffered saline.	Indirect immunofluorescence assay	Serum	IgM and IgG	Recent infection	Humans	LASV suspected cases	Not applicable	Not applicable
Demby, 2001	Cross-sectional	Probabilistic	Trapping	Multicenter	Prospetively	Guinea	Apr/1996-Apr/1997	Unclear/ Not reported	Unclear/ Not reported	Unclear/ Not reported	Urban/rural	Community-based	Not applicable (if not in the hospital)	Not applicable (if not in the hospital)	Not applicable (for animals and healty participants)	Not applicable (for animals and healty participants)	Not applicable (for animals and healty participants)	Unclear/Not reported	Direct ELISA	Serum	Antigen	Current infection	Rodents	Unspecified rodents	Preliminary genus identifications were made using conventional field classification guidelines (Rosevear 1969). The carcasses were immediately fixed in 10% formalin for 7 days and then stored in 70% alcohol until shipped to the Museum of Southwestern Biology of the University of New Mexico in Albuquerque, NM, for definitive identification, archiving, and storage.	No
Demby, 2001	Cross-sectional	Probabilistic	Trapping	Multicenter	Prospetively	Guinea	Apr/1996-Apr/1997	Unclear/ Not reported	Unclear/ Not reported	Unclear/ Not reported	Urban/rural	Community-based	Not applicable (if not in the hospital)	Not applicable (if not in the hospital)	Not applicable (for animals and healty participants)	Not applicable (for animals and healty participants)	Not applicable (for animals and healty participants)	Unclear/Not reported	Direct ELISA	Serum	Antigen	Current infection	Rodents	Unspecified rodents	Preliminary genus identifications were made using conventional field classification guidelines (Rosevear 1969). The carcasses were immediately fixed in 10% formalin for 7 days and then stored in 70% alcohol until shipped to the Museum of Southwestern Biology of the University of New Mexico in Albuquerque, NM, for definitive identification, archiving, and storage.	No
Demby, 2001	Cross-sectional	Probabilistic	Trapping	Multicenter	Prospetively	Guinea	Apr/1996-Apr/1997	Unclear/ Not reported	Unclear/ Not reported	Unclear/ Not reported	Urban/rural	Community-based	Not applicable (if not in the hospital)	Not applicable (if not in the hospital)	Not applicable (for animals and healty participants)	Not applicable (for animals and healty participants)	Not applicable (for animals and healty participants)	Unclear/Not reported	Direct ELISA	Serum	Antigen	Current infection	Rodents	Unspecified rodents	Preliminary genus identifications were made using conventional field classification guidelines (Rosevear 1969). The carcasses were immediately fixed in 10% formalin for 7 days and then stored in 70% alcohol until shipped to the Museum of Southwestern Biology of the University of New Mexico in Albuquerque, NM, for definitive identification, archiving, and storage.	No
Demby, 2001	Cross-sectional	Probabilistic	Trapping	Multicenter	Prospetively	Guinea	Apr/1996-Apr/1997	Unclear/ Not reported	Unclear/ Not reported	Unclear/ Not reported	Urban/rural	Community-based	Not applicable (if not in the hospital)	Not applicable (if not in the hospital)	Not applicable (for animals and healty participants)	Not applicable (for animals and healty participants)	Not applicable (for animals and healty participants)	Unclear/Not reported	Indirect ELISA	Serum	IgG	At least one past contact	Rodents	Unspecified rodents	Preliminary genus identifications were made using conventional field classification guidelines (Rosevear 1969). The carcasses were immediately fixed in 10% formalin for 7 days and then stored in 70% alcohol until shipped to the Museum of Southwestern Biology of the University of New Mexico in Albuquerque, NM, for definitive identification, archiving, and storage.	No
Demby, 2001	Cross-sectional	Probabilistic	Trapping	Multicenter	Prospetively	Guinea	Apr/1996-Apr/1997	Unclear/ Not reported	Unclear/ Not reported	Unclear/ Not reported	Urban/rural	Community-based	Not applicable (if not in the hospital)	Not applicable (if not in the hospital)	Not applicable (for animals and healty participants)	Not applicable (for animals and healty participants)	Not applicable (for animals and healty participants)	Unclear/Not reported	Indirect ELISA	Serum	IgG	At least one past contact	Rodents	Unspecified rodents	Preliminary genus identifications were made using conventional field classification guidelines (Rosevear 1969). The carcasses were immediately fixed in 10% formalin for 7 days and then stored in 70% alcohol until shipped to the Museum of Southwestern Biology of the University of New Mexico in Albuquerque, NM, for definitive identification, archiving, and storage.	No
Demby, 2001	Cross-sectional	Probabilistic	Trapping	Multicenter	Prospetively	Guinea	Apr/1996-Apr/1997	Unclear/ Not reported	Unclear/ Not reported	Unclear/ Not reported	Urban/rural	Community-based	Not applicable (if not in the hospital)	Not applicable (if not in the hospital)	Not applicable (for animals and healty participants)	Not applicable (for animals and healty participants)	Not applicable (for animals and healty participants)	Unclear/Not reported	Indirect ELISA	Serum	IgG	At least one past contact	Rodents	Unspecified rodents	Preliminary genus identifications were made using conventional field classification guidelines (Rosevear 1969). The carcasses were immediately fixed in 10% formalin for 7 days and then stored in 70% alcohol until shipped to the Museum of Southwestern Biology of the University of New Mexico in Albuquerque, NM, for definitive identification, archiving, and storage.	No
Ehichioya , 2012	Cross-sectional	Non probabilistic	Convenience sampling	Multicenter	Retrospectively	Nigeria	Nov/2005-Feb/2008	25	1.5- 61	Unclear/ Not reported	Unclear/Not reported	Hospital-based	Not applicable (if not in the hospital)	Hospitalized	Lassa fever was suspected in a febrile patient if malaria or bacterial infection were rendered unlikely by laboratory investigation or if fever persisted despite antimalarial and antibiotic treatment. In addition, known signs of Lassa fever were considered (McCormick et al. 1987). The doctor responsible for the patient's care raised the suspicion.	Unclear/Not reported	Unclear/Not reported	Unclear/Not reported	Classical RT-PCR	Serum	RNA	Current infection	Humans	Febrile patients	Not applicable	Not applicable
Ehichioya , 2012	Cross-sectional	Non probabilistic	Convenience sampling	Multicenter	Retrospectively	Nigeria	Nov/2005-Feb/2008	25	1.5- 61	Unclear/ Not reported	Unclear/Not reported	Hospital-based	Not applicable (if not in the hospital)	Hospitalized	Lassa fever was suspected in a febrile patient if malaria or bacterial infection were rendered unlikely by laboratory investigation or if fever persisted despite antimalarial and antibiotic treatment. In addition, known signs of Lassa fever were considered (McCormick et al. 1987). The doctor responsible for the patient's care raised the suspicion.	Unclear/Not reported	Unclear/Not reported	Unclear/Not reported	Classical RT-PCR	Serum	RNA	Current infection	Humans	LASV suspected cases	Not applicable	Not applicable
Ehichioya , 2012	Cross-sectional	Non probabilistic	Convenience sampling	Multicenter	Retrospectively	Nigeria	Nov/2005-Feb/2008	25	1.5- 61	Unclear/ Not reported	Unclear/Not reported	Hospital-based	Not applicable (if not in the hospital)	Hospitalized	Lassa fever was suspected in a febrile patient if malaria or bacterial infection were rendered unlikely by laboratory investigation or if fever persisted despite antimalarial and antibiotic treatment. In addition, known signs of Lassa fever were considered (McCormick et al. 1987). The doctor responsible for the patient's care raised the suspicion.	Unclear/Not reported	Unclear/Not reported	Unclear/Not reported	Indirect immunofluorescence assay	Serum	IgM	Recent infection	Humans	LASV suspected cases	Not applicable	Not applicable
Ehichioya , 2012	Cross-sectional	Non probabilistic	Convenience sampling	Multicenter	Retrospectively	Nigeria	Nov/2005-Feb/2008	25	1.5- 61	Unclear/ Not reported	Unclear/Not reported	Hospital-based	Not applicable (if not in the hospital)	Hospitalized	Lassa fever was suspected in a febrile patient if malaria or bacterial infection were rendered unlikely by laboratory investigation or if fever persisted despite antimalarial and antibiotic treatment. In addition, known signs of Lassa fever were considered (McCormick et al. 1987). The doctor responsible for the patient's care raised the suspicion.	Unclear/Not reported	Unclear/Not reported	Unclear/Not reported	Indirect immunofluorescence assay	Serum	IgG	At least one past contact	Humans	LASV suspected cases	Not applicable	Not applicable
Emmerich, 2006	Cross-sectional	Non probabilistic	Consecutive sampling	Multicenter	Prospetively	Guinea, Ghana	Unclear/ Not reported	Unclear/ Not reported	Unclear/ Not reported	Unclear/ Not reported	Unclear/Not reported	Community-based	Not applicable (if not in the hospital)	Not applicable (if not in the hospital)	Not applicable (for animals and healty participants)	Not applicable (for animals and healty participants)	Not applicable (for animals and healty participants)	A confirmed anti-Lassa positive serum at a dilution of 1:1000 (IgG antibody titer in IFA 1:5120) and a negative serum samples diluted 1:10 were applied.	Indirect ELISA	Unclear/Not reported	IgG	At least one past contact	Humans	Apparently healthy individuals	Not applicable	Not applicable
Emmerich, 2006	Cross-sectional	Non probabilistic	Consecutive sampling	Multicenter	Prospetively	Guinea, Ghana	Unclear/ Not reported	Unclear/ Not reported	Unclear/ Not reported	Unclear/ Not reported	Unclear/Not reported	Community-based	Not applicable (if not in the hospital)	Not applicable (if not in the hospital)	Not applicable (for animals and healty participants)	Not applicable (for animals and healty participants)	Not applicable (for animals and healty participants)	A confirmed anti-Lassa positive serum at a dilution of 1:1000 (IgG antibody titer in IFA 1:5120) and a negative serum samples diluted 1:10 were applied.	Indirect immunofluorescence assay	Unclear/Not reported	IgG	At least one past contact	Humans	Apparently healthy individuals	Not applicable	Not applicable
Emmerich, 2008	Cross-sectional	Non probabilistic	Consecutive sampling	Multicenter	Prospetively	Guinea, Ivory Coast, Ghana, Benin, Nigeria	Unclear/ Not reported	Unclear/ Not reported	18–53	52.4	Unclear/Not reported	Unclear/Not reported	Not applicable (if not in the hospital)	Not applicable (if not in the hospital)	Not applicable (for animals and healty participants)	Not applicable (for animals and healty participants)	Not applicable (for animals and healty participants)	The cut-off OD for a positive result was calculated as mean OD of the negative samples +3 standard deviations.	Indirect ELISA	Unclear/Not reported	IgG	At least one past contact	Humans	Apparently healthy individuals	Not applicable	Not applicable
Emmerich, 2008	Cross-sectional	Non probabilistic	Consecutive sampling	Multicenter	Prospetively	Guinea, Ivory Coast, Ghana, Benin, Nigeria	Unclear/ Not reported	Unclear/ Not reported	18–53	52.4	Unclear/Not reported	Unclear/Not reported	Not applicable (if not in the hospital)	Not applicable (if not in the hospital)	Not applicable (for animals and healty participants)	Not applicable (for animals and healty participants)	Not applicable (for animals and healty participants)	The cut-off OD for a positive result was calculated as mean OD of the negative samples +3 standard deviations.	Indirect immunofluorescence assay	Unclear/Not reported	IgM and IgG	Recent infection	Humans	Apparently healthy individuals	Not applicable	Not applicable
Fabiyi, 1975	Cross-sectional	Non probabilistic	Convenience sampling	Multicenter	Prospetively	Sierra Leone	1971-1974	Unclear/ Not reported	Unclear/ Not reported	Unclear/ Not reported	Unclear/Not reported	Hospital-based	Unclear/Not reported	Hospitalized/ambulatory	Not applicable (for animals and healty participants)	Not applicable (for animals and healty participants)	Not applicable (for animals and healty participants)	Unclear/Not reported	Complement fixation test	Serum	Antibodies	At least one past contact	Humans	Healthcare workers	Not applicable	Not applicable
Fabiyi, 1979	Cross-sectional	Non probabilistic	Consecutive sampling	Multicenter	Prospetively	Nigeria	Unclear/ Not reported	Unclear/ Not reported	Unclear/ Not reported	Unclear/ Not reported	Unclear/Not reported	Hospital-based	State hospital	Ambulatory	Not applicable (for animals and healty participants)	Not applicable (for animals and healty participants)	Not applicable (for animals and healty participants)	Unclear/Not reported	Complement fixation test	Serum	Antibodies	At least one past contact	Humans	Healthcare workers	Not applicable	Not applicable
Fabiyi, 1979	Cross-sectional	Non probabilistic	Consecutive sampling	Multicenter	Prospetively	Nigeria	Unclear/ Not reported	Unclear/ Not reported	3-56	Unclear/ Not reported	Unclear/Not reported	Hospital-based	State hospital	Unclear/Not reported	Unclear/Not reported	Unclear/Not reported	Unclear/Not reported	Unclear/Not reported	Complement fixation test	Serum	Antibodies	At least one past contact	Humans	LASV suspected cases	Not applicable	Not applicable
Fair, 2007	Cross-sectional	Non probabilistic	Trapping	Monocenter	Prospetively	Guinea	Jul/2004-Oct/2004	Unclear/ Not reported	Unclear/ Not reported	Unclear/ Not reported	Unclear/Not reported	Community-based	Not applicable (if not in the hospital)	Not applicable (if not in the hospital)	Not applicable (for animals and healty participants)	Not applicable (for animals and healty participants)	Not applicable (for animals and healty participants)	Unclear/Not reported	Direct ELISA	Serum	Antigen	Current infection	Rodents	Unspecified rodents	Captured animals were euthanized, identified as Mastomys spp. by standard morphometric assessment, and blood and spleen were harvested. Identification was made only to the genus level because the M. natalensis speciescomplex consists of several cryptic species that can only be distinguished by molecular and cellular techniques not routinely available at the International Center for Research on Tropical Infections, in N'Zérékoré, Guinea, where the laboratory work was performed (Lecompte et al. 2005).	No
Fichet-Calvet, 2007	Cross-sectional	Non probabilistic	Trapping	Monocenter	Prospetively	Guinea	May/2003-Jan/2005	Unclear/ Not reported	Unclear/ Not reported	Unclear/ Not reported	Unclear/Not reported	Community-based	Not applicable (if not in the hospital)	Not applicable (if not in the hospital)	Not applicable (for animals and healty participants)	Not applicable (for animals and healty participants)	Not applicable (for animals and healty participants)	Unclear/Not reported	Classical RT-PCR	Serum and/or homogenized spleen	RNA	Current infection	Rodents	Lemniscomys striatus/linulus	They were described morphologically, weighed, and measured (length of head and body, tail, hind foot, and ear) so that a preliminary identification could be made according to the keys published for West Africa (Duplantier and Granjon 1993, Rosevear 1969). Confirmation of Mastomys species was made by cytochrome b-based polymerase chain reaction (PCR; Lecompte et al. 2005).	Yes
Fichet-Calvet, 2007	Cross-sectional	Non probabilistic	Trapping	Monocenter	Prospetively	Guinea	May/2003-Jan/2005	Unclear/ Not reported	Unclear/ Not reported	Unclear/ Not reported	Unclear/Not reported	Community-based	Not applicable (if not in the hospital)	Not applicable (if not in the hospital)	Not applicable (for animals and healty participants)	Not applicable (for animals and healty participants)	Not applicable (for animals and healty participants)	Unclear/Not reported	Classical RT-PCR	Serum and/or homogenized spleen	RNA	Current infection	Rodents	Myomys daltoni	They were described morphologically, weighed, and measured (length of head and body, tail, hind foot, and ear) so that a preliminary identification could be made according to the keys published for West Africa (Duplantier and Granjon 1993, Rosevear 1969). Confirmation of Mastomys species was made by cytochrome b-based polymerase chain reaction (PCR; Lecompte et al. 2005).	Yes
Fichet-Calvet, 2007	Cross-sectional	Non probabilistic	Trapping	Monocenter	Prospetively	Guinea	May/2003-Jan/2005	Unclear/ Not reported	Unclear/ Not reported	Unclear/ Not reported	Unclear/Not reported	Community-based	Not applicable (if not in the hospital)	Not applicable (if not in the hospital)	Not applicable (for animals and healty participants)	Not applicable (for animals and healty participants)	Not applicable (for animals and healty participants)	Unclear/Not reported	Classical RT-PCR	Serum and/or homogenized spleen	RNA	Current infection	Rodents	Mastomys natalensis	They were described morphologically, weighed, and measured (length of head and body, tail, hind foot, and ear) so that a preliminary identification could be made according to the keys published for West Africa (Duplantier and Granjon 1993, Rosevear 1969). Confirmation of Mastomys species was made by cytochrome b-based polymerase chain reaction (PCR; Lecompte et al. 2005).	Yes
Fichet-Calvet, 2007	Cross-sectional	Non probabilistic	Trapping	Monocenter	Prospetively	Guinea	May/2003-Jan/2005	Unclear/ Not reported	Unclear/ Not reported	Unclear/ Not reported	Unclear/Not reported	Community-based	Not applicable (if not in the hospital)	Not applicable (if not in the hospital)	Not applicable (for animals and healty participants)	Not applicable (for animals and healty participants)	Not applicable (for animals and healty participants)	Unclear/Not reported	Classical RT-PCR	Serum and/or homogenized spleen	RNA	Current infection	Rodents	Mastomys erythroleucus	They were described morphologically, weighed, and measured (length of head and body, tail, hind foot, and ear) so that a preliminary identification could be made according to the keys published for West Africa (Duplantier and Granjon 1993, Rosevear 1969). Confirmation of Mastomys species was made by cytochrome b-based polymerase chain reaction (PCR; Lecompte et al. 2005).	Yes
Fichet-Calvet, 2007	Cross-sectional	Non probabilistic	Trapping	Monocenter	Prospetively	Guinea	May/2003-Jan/2005	Unclear/ Not reported	Unclear/ Not reported	Unclear/ Not reported	Unclear/Not reported	Community-based	Not applicable (if not in the hospital)	Not applicable (if not in the hospital)	Not applicable (for animals and healty participants)	Not applicable (for animals and healty participants)	Not applicable (for animals and healty participants)	Unclear/Not reported	Classical RT-PCR	Serum and/or homogenized spleen	RNA	Current infection	Rodents	Nannomys minutoides/mattheyi	They were described morphologically, weighed, and measured (length of head and body, tail, hind foot, and ear) so that a preliminary identification could be made according to the keys published for West Africa (Duplantier and Granjon 1993, Rosevear 1969). Confirmation of Mastomys species was made by cytochrome b-based polymerase chain reaction (PCR; Lecompte et al. 2005).	Yes
Fichet-Calvet, 2007	Cross-sectional	Non probabilistic	Trapping	Monocenter	Prospetively	Guinea	May/2003-Jan/2005	Unclear/ Not reported	Unclear/ Not reported	Unclear/ Not reported	Unclear/Not reported	Community-based	Not applicable (if not in the hospital)	Not applicable (if not in the hospital)	Not applicable (for animals and healty participants)	Not applicable (for animals and healty participants)	Not applicable (for animals and healty participants)	Unclear/Not reported	Classical RT-PCR	Serum and/or homogenized spleen	RNA	Current infection	Rodents	Praomys rostratus	They were described morphologically, weighed, and measured (length of head and body, tail, hind foot, and ear) so that a preliminary identification could be made according to the keys published for West Africa (Duplantier and Granjon 1993, Rosevear 1969). Confirmation of Mastomys species was made by cytochrome b-based polymerase chain reaction (PCR; Lecompte et al. 2005).	Yes
Fichet-Calvet, 2014	Cross-sectional	Non probabilistic	Trapping	Multicenter	Prospetively	Guinea, Mali	Oct/2002-Oct/2004	Unclear/ Not reported	Unclear/ Not reported	Unclear/ Not reported	Unclear/Not reported	Community-based	Not applicable (if not in the hospital)	Not applicable (if not in the hospital)	Not applicable (for animals and healty participants)	Not applicable (for animals and healty participants)	Not applicable (for animals and healty participants)	Animals were considered positive when the signal was two or three intensity units in the two initial tests (regular slides), or one intensity unit in all three tests (regular plus mixed slides).	Indirect immunofluorescence assay	Serum, spleen fluid, liver fluid	IgG	At least one past contact	Rodents	Crocidura buettikoferi	After trapping, the animals were necropsied in the field and identified morphologically (weight and body length). The rodent species was identified molecularly by sequencing the cytochrome b gene.	Yes
Fichet-Calvet, 2014	Cross-sectional	Non probabilistic	Trapping	Multicenter	Prospetively	Guinea, Mali	Oct/2002-Oct/2004	Unclear/ Not reported	Unclear/ Not reported	Unclear/ Not reported	Unclear/Not reported	Community-based	Not applicable (if not in the hospital)	Not applicable (if not in the hospital)	Not applicable (for animals and healty participants)	Not applicable (for animals and healty participants)	Not applicable (for animals and healty participants)	Animals were considered positive when the signal was two or three intensity units in the two initial tests (regular slides), or one intensity unit in all three tests (regular plus mixed slides).	Indirect immunofluorescence assay	Serum, spleen fluid, liver fluid	IgG	At least one past contact	Rodents	Gerbilliscus guineae	After trapping, the animals were necropsied in the field and identified morphologically (weight and body length). The rodent species was identified molecularly by sequencing the cytochrome b gene.	Yes
Fichet-Calvet, 2014	Cross-sectional	Non probabilistic	Trapping	Multicenter	Prospetively	Guinea, Mali	Oct/2002-Oct/2004	Unclear/ Not reported	Unclear/ Not reported	Unclear/ Not reported	Unclear/Not reported	Community-based	Not applicable (if not in the hospital)	Not applicable (if not in the hospital)	Not applicable (for animals and healty participants)	Not applicable (for animals and healty participants)	Not applicable (for animals and healty participants)	Animals were considered positive when the signal was two or three intensity units in the two initial tests (regular slides), or one intensity unit in all three tests (regular plus mixed slides).	Indirect immunofluorescence assay	Serum, spleen fluid, liver fluid	IgG	At least one past contact	Rodents	Lemniscomys bellieri/zebra	After trapping, the animals were necropsied in the field and identified morphologically (weight and body length). The rodent species was identified molecularly by sequencing the cytochrome b gene.	Yes
Fichet-Calvet, 2014	Cross-sectional	Non probabilistic	Trapping	Multicenter	Prospetively	Guinea, Mali	Oct/2002-Oct/2004	Unclear/ Not reported	Unclear/ Not reported	Unclear/ Not reported	Unclear/Not reported	Community-based	Not applicable (if not in the hospital)	Not applicable (if not in the hospital)	Not applicable (for animals and healty participants)	Not applicable (for animals and healty participants)	Not applicable (for animals and healty participants)	Animals were considered positive when the signal was two or three intensity units in the two initial tests (regular slides), or one intensity unit in all three tests (regular plus mixed slides).	Indirect immunofluorescence assay	Serum, spleen fluid, liver fluid	IgG	At least one past contact	Rodents	Lemniscomys striatus	After trapping, the animals were necropsied in the field and identified morphologically (weight and body length). The rodent species was identified molecularly by sequencing the cytochrome b gene.	Yes

Fichet-Calvet, 2014	Cross-sectional	Non probabilistic	Trapping	Multicenter	Prospetively	Guinea, Mali	Oct/2002-Oct/2004	Unclear/ Not reported	Unclear/ Not reported	Unclear/ Not reported	Unclear/Not reported	Community-based	Not applicable (if not in the hospital)	Not applicable (if not in the hospital)	Not applicable (for animals and healty participants)	Not applicable (for animals and healty participants)	Not applicable (for animals and healty participants)	Animals were considered positive when the signal was two or three intensity units in the two initial tests (regular slides), or one intensity unit in all three tests (regular plus mixed slides).	Indirect immunofluorescence assay	Serum, spleen fluid, liver fluid	IgG	At least one past contact	Rodents	Lophuromys sikapusi	After trapping, the animals were necropsied in the field and identified morphologically (weight and body length). The rodent species was identified molecularly by sequencing the cytochrome b gene.	Yes
Fichet-Calvet, 2014	Cross-sectional	Non probabilistic	Trapping	Multicenter	Prospetively	Guinea, Mali	Oct/2002-Oct/2004	Unclear/ Not reported	Unclear/ Not reported	Unclear/ Not reported	Unclear/Not reported	Community-based	Not applicable (if not in the hospital)	Not applicable (if not in the hospital)	Not applicable (for animals and healty participants)	Not applicable (for animals and healty participants)	Not applicable (for animals and healty participants)	Animals were considered positive when the signal was two or three intensity units in the two initial tests (regular slides), or one intensity unit in all three tests (regular plus mixed slides).	Indirect immunofluorescence assay	Serum, spleen fluid, liver fluid	IgG	At least one past contact	Rodents	Mastomys erythroleucus	After trapping, the animals were necropsied in the field and identified morphologically (weight and body length). The rodent species was identified molecularly by sequencing the cytochrome b gene.	Yes
Fichet-Calvet, 2014	Cross-sectional	Non probabilistic	Trapping	Multicenter	Prospetively	Guinea, Mali	Oct/2002-Oct/2004	Unclear/ Not reported	Unclear/ Not reported	Unclear/ Not reported	Unclear/Not reported	Community-based	Not applicable (if not in the hospital)	Not applicable (if not in the hospital)	Not applicable (for animals and healty participants)	Not applicable (for animals and healty participants)	Not applicable (for animals and healty participants)	Animals were considered positive when the signal was two or three intensity units in the two initial tests (regular slides), or one intensity unit in all three tests (regular plus mixed slides).	Indirect immunofluorescence assay	Serum, spleen fluid, liver fluid	IgG	At least one past contact	Rodents	Mastomys natalensis	After trapping, the animals were necropsied in the field and identified morphologically (weight and body length). The rodent species was identified molecularly by sequencing the cytochrome b gene.	Yes
Fichet-Calvet, 2014	Cross-sectional	Non probabilistic	Trapping	Multicenter	Prospetively	Guinea, Mali	Oct/2002-Oct/2004	Unclear/ Not reported	Unclear/ Not reported	Unclear/ Not reported	Unclear/Not reported	Community-based	Not applicable (if not in the hospital)	Not applicable (if not in the hospital)	Not applicable (for animals and healty participants)	Not applicable (for animals and healty participants)	Not applicable (for animals and healty participants)	Animals were considered positive when the signal was two or three intensity units in the two initial tests (regular slides), or one intensity unit in all three tests (regular plus mixed slides).	Indirect immunofluorescence assay	Serum, spleen fluid, liver fluid	IgG	At least one past contact	Rodents	Mus mattheyi	After trapping, the animals were necropsied in the field and identified morphologically (weight and body length). The rodent species was identified molecularly by sequencing the cytochrome b gene.	Yes
Fichet-Calvet, 2014	Cross-sectional	Non probabilistic	Trapping	Multicenter	Prospetively	Guinea, Mali	Oct/2002-Oct/2004	Unclear/ Not reported	Unclear/ Not reported	Unclear/ Not reported	Unclear/Not reported	Community-based	Not applicable (if not in the hospital)	Not applicable (if not in the hospital)	Not applicable (for animals and healty participants)	Not applicable (for animals and healty participants)	Not applicable (for animals and healty participants)	Animals were considered positive when the signal was two or three intensity units in the two initial tests (regular slides), or one intensity unit in all three tests (regular plus mixed slides).	Indirect immunofluorescence assay	Serum, spleen fluid, liver fluid	IgG	At least one past contact	Rodents	Mus minutoides	After trapping, the animals were necropsied in the field and identified morphologically (weight and body length). The rodent species was identified molecularly by sequencing the cytochrome b gene.	Yes
Fichet-Calvet, 2014	Cross-sectional	Non probabilistic	Trapping	Multicenter	Prospetively	Guinea, Mali	Oct/2002-Oct/2004	Unclear/ Not reported	Unclear/ Not reported	Unclear/ Not reported	Unclear/Not reported	Community-based	Not applicable (if not in the hospital)	Not applicable (if not in the hospital)	Not applicable (for animals and healty participants)	Not applicable (for animals and healty participants)	Not applicable (for animals and healty participants)	Animals were considered positive when the signal was two or three intensity units in the two initial tests (regular slides), or one intensity unit in all three tests (regular plus mixed slides).	Indirect immunofluorescence assay	Serum, spleen fluid, liver fluid	IgG	At least one past contact	Rodents	Mus setulosus	After trapping, the animals were necropsied in the field and identified morphologically (weight and body length). The rodent species was identified molecularly by sequencing the cytochrome b gene.	Yes
Fichet-Calvet, 2014	Cross-sectional	Non probabilistic	Trapping	Multicenter	Prospetively	Guinea, Mali	Oct/2002-Oct/2004	Unclear/ Not reported	Unclear/ Not reported	Unclear/ Not reported	Unclear/Not reported	Community-based	Not applicable (if not in the hospital)	Not applicable (if not in the hospital)	Not applicable (for animals and healty participants)	Not applicable (for animals and healty participants)	Not applicable (for animals and healty participants)	Animals were considered positive when the signal was two or three intensity units in the two initial tests (regular slides), or one intensity unit in all three tests (regular plus mixed slides).	Indirect immunofluorescence assay	Serum, spleen fluid, liver fluid	IgG	At least one past contact	Rodents	Praomys daltoni	After trapping, the animals were necropsied in the field and identified morphologically (weight and body length). The rodent species was identified molecularly by sequencing the cytochrome b gene.	Yes
Fichet-Calvet, 2014	Cross-sectional	Non probabilistic	Trapping	Multicenter	Prospetively	Guinea, Mali	Oct/2002-Oct/2004	Unclear/ Not reported	Unclear/ Not reported	Unclear/ Not reported	Unclear/Not reported	Community-based	Not applicable (if not in the hospital)	Not applicable (if not in the hospital)	Not applicable (for animals and healty participants)	Not applicable (for animals and healty participants)	Not applicable (for animals and healty participants)	Animals were considered positive when the signal was two or three intensity units in the two initial tests (regular slides), or one intensity unit in all three tests (regular plus mixed slides).	Indirect immunofluorescence assay	Serum, spleen fluid, liver fluid	IgG	At least one past contact	Rodents	Praomys rostratus	After trapping, the animals were necropsied in the field and identified morphologically (weight and body length). The rodent species was identified molecularly by sequencing the cytochrome b gene.	Yes
Fichet-Calvet, 2014	Cross-sectional	Non probabilistic	Trapping	Multicenter	Prospetively	Guinea, Mali	Oct/2002-Oct/2004	Unclear/ Not reported	Unclear/ Not reported	Unclear/ Not reported	Unclear/Not reported	Community-based	Not applicable (if not in the hospital)	Not applicable (if not in the hospital)	Not applicable (for animals and healty participants)	Not applicable (for animals and healty participants)	Not applicable (for animals and healty participants)	Animals were considered positive when the signal was two or three intensity units in the two initial tests (regular slides), or one intensity unit in all three tests (regular plus mixed slides).	Indirect immunofluorescence assay	Serum, spleen fluid, liver fluid	IgG	At least one past contact	Rodents	Rattus rattus	After trapping, the animals were necropsied in the field and identified morphologically (weight and body length). The rodent species was identified molecularly by sequencing the cytochrome b gene.	Yes
Fichet-Calvet, 2014	Cross-sectional	Non probabilistic	Trapping	Multicenter	Prospetively	Guinea, Mali	Oct/2002-Oct/2004	Unclear/ Not reported	Unclear/ Not reported	Unclear/ Not reported	Unclear/Not reported	Community-based	Not applicable (if not in the hospital)	Not applicable (if not in the hospital)	Not applicable (for animals and healty participants)	Not applicable (for animals and healty participants)	Not applicable (for animals and healty participants)	Animals were considered positive when the signal was two or three intensity units in the two initial tests (regular slides), or one intensity unit in all three tests (regular plus mixed slides).	Indirect immunofluorescence assay	Serum, spleen fluid, liver fluid	IgG	At least one past contact	Rodents	Uranomys ruddi	After trapping, the animals were necropsied in the field and identified morphologically (weight and body length). The rodent species was identified molecularly by sequencing the cytochrome b gene.	Yes
Fisher-Hoch, 1988	Cross-sectional	Non probabilistic	Convenience sampling	Monocenter	Prospetively	Sierra Leone	Jan-Apr/1985	Unclear/ Not reported	Unclear/ Not reported	Unclear/ Not reported	Unclear/Not reported	Hospital-based	Unclear/Not reported	Hospitalized	Unclear/Not reported	Unclear/Not reported	Unclear/Not reported	Patients were diagnosed as having Lassa fever if they had an eightfold or greater rise in titer to Lassa antigen by immunofluorescent antibody assay (IFA) or a titer of 256 or more with Lassa specific IgM, and/or virus isolation from serum	Immunoflorescent assay, Culture	Serum	IgG, IgM, Virus	Current infection	Humans	LASV suspected cases	Not applicable	Not applicable
Fisher-Hoch, 1995	Cross-sectional	Non probabilistic	Consecutive sampling	Multicenter	Retrospectively	Nigeria	Jan/1989-Mar/1989	Unclear/ Not reported	Unclear/ Not reported	Unclear/ Not reported	Unclear/Not reported	Hospital/community based	University of Nigeria Teaching Hospital	Ambulatory	Not applicable (for animals and healty participants)	Not applicable (for animals and healty participants)	Not applicable (for animals and healty participants)	Unclear/Not reported	Indirect immunofluorescence assay	Serum	IgM and IgG	Recent infection	Humans	Apparently healthy individuals	Not applicable	Not applicable
Fisher-Hoch, 1995	Cross-sectional	Non probabilistic	Consecutive sampling	Multicenter	Retrospectively	Nigeria	Jan/1989-Mar/1989	Unclear/ Not reported	Unclear/ Not reported	Unclear/ Not reported	Unclear/Not reported	Hospital/community based	University of Nigeria Teaching Hospital	Ambulatory	Not applicable (for animals and healty participants)	Not applicable (for animals and healty participants)	Not applicable (for animals and healty participants)	Unclear/Not reported	Indirect immunofluorescence assay	Serum	IgG	At least one past contact	Humans	Healthcare workers	Not applicable	Not applicable
Fisher-Hoch, 1995	Cross-sectional	Non probabilistic	Consecutive sampling	Multicenter	Retrospectively	Nigeria	Jan/1989-Mar/1989	Unclear/ Not reported	Unclear/ Not reported	Unclear/ Not reported	Unclear/Not reported	Hospital/community based	University of Nigeria Teaching Hospital	Unclear/Not reported	Unclear/Not reported	Unclear/Not reported	Presumptive Lassa fever was defined by history of prolonged fever (more than five days), sore throat, proteinuria, oedema, bleeding or convulsions, and lack of response to antimicrobial and antimalarial agents between 1 January and 31 March 1989.'	Unclear/Not reported	Indirect immunofluorescence assay	Unclear/Not reported	IgM and IgG	Recent infection	Humans	LASV suspected cases	Not applicable	Not applicable
Frame, 1979	Cross-sectional	Non probabilistic	Convenience sampling	Multicenter	Prospetively	Liberia	Oct/1976-Oct/1977	Unclear/ Not reported	Unclear/ Not reported	Unclear/ Not reported	Unclear/Not reported	Hospital-based	Unclear/Not reported	Ambulatory	Not applicable (for animals and healty participants)	Not applicable (for animals and healty participants)	Not applicable (for animals and healty participants)	Unclear/Not reported	Indirect immunofluorescence assay	Serum	Antibodies	At least one past contact	Humans	Healthcare workers	Not applicable	Not applicable
Frame, 1984	Cross-sectional	Non probabilistic	Convenience sampling	Multicenter	Prospetively	Liberia	1979-1982	Unclear/ Not reported	Unclear/ Not reported	Unclear/ Not reported	Unclear/Not reported	Hospital-based	Unclear/Not reported	Ambulatory	Not applicable (for animals and healty participants)	Not applicable (for animals and healty participants)	Not applicable (for animals and healty participants)	Unclear/Not reported	Indirect immunofluorescence assay	Serum	Antibodies	At least one past contact	Humans	Healthcare workers	Not applicable	Not applicable
Frame, 1984	Cross-sectional	Non probabilistic	Consecutive sampling	Multicenter	Prospetively	Liberia	Jul/1980-Apr/1982	Unclear/ Not reported	Unclear/ Not reported	Unclear/ Not reported	Unclear/Not reported	Hospital-based	Unclear/Not reported	Hospitalized	Unclear/Not reported	Unclear/Not reported	Presumptive Lassa fever: Serum pairs were not always available if patients were discharged or removed from the hospital by their families too soon to allow a diagnostic rise in LVA. Under these circumstances a titre of 1164 or higher was considered to reflect current or recent LF in a patient with an illness clinically compatible with LF.	Lassa fever: (i) Isolation of LV from specimens tested at USAMRIID. (ii) A four-fold or greater rise in LV antibody (LVA) titres by the IFA technique obtained at the LIBR laboratory and confirmed by tests at USAMRIID was considered diagnostic. With a few patients one or another of the serum specimens was not tested at USAMRIID and in these circumstances a four-fold antibody rise at the LIBR was accepted as diagnostic.	Culture	Serum	Virus	Current infection	Humans	Febrile patients	Not applicable	Not applicable
Frame, 1984	Cross-sectional	Non probabilistic	Consecutive sampling	Multicenter	Prospetively	Liberia	Jul/1980-Apr/1982	Unclear/ Not reported	Unclear/ Not reported	Unclear/ Not reported	Unclear/Not reported	Hospital-based	Unclear/Not reported	Hospitalized	Unclear/Not reported	Unclear/Not reported	Presumptive Lassa fever: Serum pairs were not always available if patients were discharged or removed from the hospital by their families too soon to allow a diagnostic rise in LVA. Under these circumstances a titre of 1164 or higher was considered to reflect current or recent LF in a patient with an illness clinically compatible with LF.	Lassa fever: (i) Isolation of LV from specimens tested at USAMRIID. (ii) A four-fold or greater rise in LV antibody (LVA) titres by the IFA technique obtained at the LIBR laboratory and confirmed by tests at USAMRIID was considered diagnostic. With a few patients one or another of the serum specimens was not tested at USAMRIID and in these circumstances a four-fold antibody rise at the LIBR was accepted as diagnostic.	Indirect immunofluorescence assay	Serum	Antibodies	At least one past contact	Humans	Febrile patients	Not applicable	Not applicable
Georges, 1985	Cross-sectional	Non probabilistic	Consecutive sampling	Multicenter	Prospetively	Central African Republic	1985	Unclear/ Not reported	Unclear/ Not reported	Unclear/ Not reported	Rural	Community-based	Not applicable (if not in the hospital)	Not applicable (if not in the hospital)	Not applicable (for animals and healty participants)	Not applicable (for animals and healty participants)	Not applicable (for animals and healty participants)	Unclear/Not reported	Indirect immunofluorescence assay	Serum	Antibodies	At least one past contact	Humans	Apparently healthy individuals	Not applicable	Not applicable
Georges, 1985	Cross-sectional	Non probabilistic	Consecutive sampling	Multicenter	Prospetively	Central African Republic	1985	Unclear/ Not reported	Unclear/ Not reported	Unclear/ Not reported	Rural	Community-based	Not applicable (if not in the hospital)	Not applicable (if not in the hospital)	Not applicable (for animals and healty participants)	Not applicable (for animals and healty participants)	Not applicable (for animals and healty participants)	Unclear/Not reported	Indirect immunofluorescence assay	Serum	Antibodies	At least one past contact	Other mammals	cattle	Not applicable	Not applicable
Georges, 1985	Cross-sectional	Non probabilistic	Consecutive sampling	Multicenter	Prospetively	Central African Republic	1985	Unclear/ Not reported	Unclear/ Not reported	Unclear/ Not reported	Rural	Community-based	Not applicable (if not in the hospital)	Not applicable (if not in the hospital)	Not applicable (for animals and healty participants)	Not applicable (for animals and healty participants)	Not applicable (for animals and healty participants)	Unclear/Not reported	Indirect immunofluorescence assay	Serum	Antibodies	At least one past contact	Other mammals	dog	Not applicable	Not applicable
Georges, 1985	Cross-sectional	Non probabilistic	Consecutive sampling	Multicenter	Prospetively	Central African Republic	1985	Unclear/ Not reported	Unclear/ Not reported	Unclear/ Not reported	Rural	Community-based	Not applicable (if not in the hospital)	Not applicable (if not in the hospital)	Not applicable (for animals and healty participants)	Not applicable (for animals and healty participants)	Not applicable (for animals and healty participants)	Unclear/Not reported	Indirect immunofluorescence assay	Serum	Antibodies	At least one past contact	Other mammals	goat	Not applicable	Not applicable
Georges, 1985	Cross-sectional	Non probabilistic	Consecutive sampling	Multicenter	Prospetively	Central African Republic	1985	Unclear/ Not reported	Unclear/ Not reported	Unclear/ Not reported	Rural	Community-based	Not applicable (if not in the hospital)	Not applicable (if not in the hospital)	Not applicable (for animals and healty participants)	Not applicable (for animals and healty participants)	Not applicable (for animals and healty participants)	Unclear/Not reported	Indirect immunofluorescence assay	Serum	Antibodies	At least one past contact	Other mammals	guinea-pig	Not applicable	Not applicable
Georges, 1985	Cross-sectional	Non probabilistic	Consecutive sampling	Multicenter	Prospetively	Central African Republic	1985	Unclear/ Not reported	Unclear/ Not reported	Unclear/ Not reported	Rural	Community-based	Not applicable (if not in the hospital)	Not applicable (if not in the hospital)	Not applicable (for animals and healty participants)	Not applicable (for animals and healty participants)	Not applicable (for animals and healty participants)	Unclear/Not reported	Indirect immunofluorescence assay	Serum	Antibodies	At least one past contact	Other mammals	sheep	Not applicable	Not applicable
Gonzalez, 1983	Cross-sectional	Non probabilistic	Trapping	Multicenter	Prospetively	Central African Republic	Unclear/ Not reported	Unclear/ Not reported	Unclear/ Not reported	Unclear/ Not reported	Rural	Community-based	Not applicable (if not in the hospital)	Not applicable (if not in the hospital)	Not applicable (for animals and healty participants)	Not applicable (for animals and healty participants)	Not applicable (for animals and healty participants)	Unclear/Not reported	Indirect immunofluorescence assay	Serum	Antibodies	At least one past contact	Rodents	Unspecified rodents	Identification of the rodents was done on the dissecting board where their total length, tail length, length of back foot, and ear length were measured. In some cases, especially with juvenile other measures were made to assure proper identification (cranial length, tooth and toe measurements). A key for rodent species identification in the CAR has been established principally using the basic data from Rosevar [11] modified by more recent efforts to systematically classify the small mammals of the CAR [12, 13].	No
Gonzalez, 1983	Cross-sectional	Non probabilistic	Trapping	Multicenter	Prospetively	Central African Republic	Unclear/ Not reported	Unclear/ Not reported	Unclear/ Not reported	Unclear/ Not reported	Rural	Community-based	Not applicable (if not in the hospital)	Not applicable (if not in the hospital)	Not applicable (for animals and healty participants)	Not applicable (for animals and healty participants)	Not applicable (for animals and healty participants)	Unclear/Not reported	Indirect immunofluorescence assay	Serum	Antibodies	At least one past contact	Rodents	Unspecified rodents	Identification of the rodents was done on the dissecting board where their total length, tail length, length of back foot, and ear length were measured. In some cases, especially with juvenile other measures were made to assure proper identification (cranial length, tooth and toe measurements). A key for rodent species identification in the CAR has been established principally using the basic data from Rosevar [11] modified by more recent efforts to systematically classify the small mammals of the CAR [12, 13].	No
Gonzalez, 1983	Cross-sectional	Non probabilistic	Consecutive sampling	Multicenter	Prospetively	Central African Republic	Unclear/ Not reported	Unclear/ Not reported	Unclear/ Not reported	Unclear/ Not reported	Rural	Community-based	Not applicable (if not in the hospital)	Not applicable (if not in the hospital)	Unclear/Not reported	Unclear/Not reported	Unclear/Not reported	Unclear/Not reported	Indirect immunofluorescence assay	Serum	Antibodies	At least one past contact	Humans	Apparently healthy individuals, Patient with any illness	Not applicable	Not applicable
Gonzalez, 1989	Cross-sectional	Probabilistic	Cluster sampling	Multicenter	Prospetively	Cameroon	Jan/1985-Jun/1987	Unclear/ Not reported	Unclear/ Not reported	Unclear/ Not reported	Rural	Community-based	Not applicable (if not in the hospital)	Not applicable (if not in the hospital)	Not applicable (for animals and healty participants)	Not applicable (for animals and healty participants)	Not applicable (for animals and healty participants)	Unclear/Not reported	Indirect immunofluorescence assay	Serum	Antibodies	At least one past contact	Humans	Apparently healthy individuals	Not applicable	Not applicable
Gonzalez, 1989	Cross-sectional	Probabilistic	Trapping	Multicenter	Prospetively	Cameroon	Jan/1985-Jun/1987	Unclear/ Not reported	Unclear/ Not reported	Unclear/ Not reported	Rural	Community-based	Not applicable (if not in the hospital)	Not applicable (if not in the hospital)	Not applicable (for animals and healty participants)	Not applicable (for animals and healty participants)	Not applicable (for animals and healty participants)	Unclear/Not reported	Indirect immunofluorescence assay	Serum	Antibodies	At least one past contact	Rodents	Unspecified rodents	Unclear/ Not reported	Unclear/ Not reported
Gonzalez, 1989	Cross-sectional	Probabilistic	Cluster sampling	Multicenter	Prospetively	Central African Republic	Jan/1985-Jun/1987	Unclear/ Not reported	Unclear/ Not reported	Unclear/ Not reported	Rural	Community-based	Not applicable (if not in the hospital)	Not applicable (if not in the hospital)	Not applicable (for animals and healty participants)	Not applicable (for animals and healty participants)	Not applicable (for animals and healty participants)	Unclear/Not reported	Indirect immunofluorescence assay	Serum	Antibodies	At least one past contact	Humans	Apparently healthy individuals	Not applicable	Not applicable
Gonzalez, 1989	Cross-sectional	Probabilistic	Cluster sampling	Multicenter	Prospetively	Chad	Jan/1985-Jun/1987	Unclear/ Not reported	Unclear/ Not reported	Unclear/ Not reported	Rural	Community-based	Not applicable (if not in the hospital)	Not applicable (if not in the hospital)	Not applicable (for animals and healty participants)	Not applicable (for animals and healty participants)	Not applicable (for animals and healty participants)	Unclear/Not reported	Indirect immunofluorescence assay	Serum	Antibodies	At least one past contact	Humans	Apparently healthy individuals	Not applicable	Not applicable
Gonzalez, 1989	Cross-sectional	Probabilistic	Cluster sampling	Multicenter	Prospetively	Equatorial Guinea	Jan/1985-Jun/1987	Unclear/ Not reported	Unclear/ Not reported	Unclear/ Not reported	Rural	Community-based	Not applicable (if not in the hospital)	Not applicable (if not in the hospital)	Not applicable (for animals and healty participants)	Not applicable (for animals and healty participants)	Not applicable (for animals and healty participants)	Unclear/Not reported	Indirect immunofluorescence assay	Serum	Antibodies	At least one past contact	Humans	Apparently healthy individuals	Not applicable	Not applicable
Gonzalez, 1989	Cross-sectional	Probabilistic	Cluster sampling	Multicenter	Prospetively	Gabon	Jan/1985-Jun/1987	Unclear/ Not reported	Unclear/ Not reported	Unclear/ Not reported	Rural	Community-based	Not applicable (if not in the hospital)	Not applicable (if not in the hospital)	Not applicable (for animals and healty participants)	Not applicable (for animals and healty participants)	Not applicable (for animals and healty participants)	Unclear/Not reported	Indirect immunofluorescence assay	Serum	Antibodies	At least one past contact	Humans	Apparently healthy individuals	Not applicable	Not applicable
Gonzalez, 1989	Cross-sectional	Probabilistic	Cluster sampling	Multicenter	Prospetively	Republic of the Congo	Jan/1985-Jun/1987	Unclear/ Not reported	Unclear/ Not reported	Unclear/ Not reported	Rural	Community-based	Not applicable (if not in the hospital)	Not applicable (if not in the hospital)	Not applicable (for animals and healty participants)	Not applicable (for animals and healty participants)	Not applicable (for animals and healty participants)	Unclear/Not reported	Indirect immunofluorescence assay	Serum	Antibodies	At least one past contact	Humans	Apparently healthy individuals	Not applicable	Not applicable
Hamblion, 2018	Cross-sectional	Non probabilistic	Consecutive sampling	Multicenter	Prospetively	Liberia	Jan/2016-jun/2016	30	1-57	30	Unclear/Not reported	Hospital/community based	Health care facility	Hospitalized/ambulatory	Unclear/Not reported	Suspected case of Lassa fever: illness with gradual onset with one or more of the following: malaise, fever, headache, sore throat, cough, nausea, vomiting, diarrhea, myalgia, chest pain, hearing loss, and a history of contact with excreta of rodents or with a case of Lassa fever.	Unclear/Not reported	Confirmed case of Lassa fever: a suspected case that is laboratory-confirmed (positive IgM antibody, PCR or virus isolation).	Classical RT-PCR, Rapid Diagnostic Test, Indirect ELISA, Direct ELISA	Serum	RNA, Antigen, IgM, IgG	Current infection	Humans	LASV suspected cases	Not applicable	Not applicable
Haun, 2019	Cross-sectional	Non probabilistic	Consecutive sampling	Monocenter	Prospetively	Liberia	Apr/2018-May/2018	Unclear/ Not reported	< 4	Unclear/ Not reported	Unclear/Not reported	Community-based	Not applicable (if not in the hospital)	Not applicable (if not in the hospital)	Not applicable (for animals and healty participants)	Not applicable (for animals and healty participants)	Not applicable (for animals and healty participants)	Serum samples showing MFI values greater than the cutoff value were considered positive.	Microsphere immunoassay	Serum	IgG	At least one past contact	Other mammals	dog	Not applicable	Not applicable
Helmick, 1986	Cross-sectional	Non probabilistic	Convenience sampling	Multicenter	Prospetively	Sierra Leone	1972, 1977, 1978-1983	Unclear/ Not reported	Unclear/ Not reported	51,51	Rural	Hospital/community based	Emergency department	Ambulatory	Not applicable (for animals and healty participants)	Not applicable (for animals and healty participants)	Not applicable (for animals and healty participants)	Unclear/Not reported	Indirect immunofluorescence assay	Serum	Antibodies	At least one past contact	Humans	Apparently healthy individuals	Not applicable	Not applicable
Helmick, 1986	Cross-sectional	Non probabilistic	Convenience sampling	Multicenter	Prospetively	Sierra Leone	1972, 1977, 1978-1983	Unclear/ Not reported	Unclear/ Not reported	51,51	Rural	Hospital/community based	Emergency department	Ambulatory	Not applicable (for animals and healty participants)	Not applicable (for animals and healty participants)	Not applicable (for animals and healty participants)	Unclear/Not reported	Indirect immunofluorescence assay	Serum	Antibodies	At least one past contact	Humans	Healthcare workers	Not applicable	Not applicable
Henderson, 1972	Cross-sectional	Non probabilistic	Consecutive sampling	Multicenter	Prospetively	Benin	1965-1970	Unclear/ Not reported	Unclear/ Not reported	Unclear/ Not reported	Rural	Community-based	Not applicable (if not in the hospital)	Not applicable (if not in the hospital)	Not applicable (for animals and healty participants)	Not applicable (for animals and healty participants)	Not applicable (for animals and healty participants)	Unclear/Not reported	Neutralization test	Serum	Antibodies	At least one past contact	Humans	Apparently healthy individuals	Not applicable	Not applicable
Henderson, 1972	Cross-sectional	Non probabilistic	Consecutive sampling	Multicenter	Prospetively	Burkina Faso	1965-1970	Unclear/ Not reported	Unclear/ Not reported	Unclear/ Not reported	Rural	Community-based	Not applicable (if not in the hospital)	Not applicable (if not in the hospital)	Not applicable (for animals and healty participants)	Not applicable (for animals and healty participants)	Not applicable (for animals and healty participants)	Unclear/Not reported	Neutralization test	Serum	Antibodies	At least one past contact	Humans	Apparently healthy individuals	Not applicable	Not applicable
Henderson, 1972	Cross-sectional	Non probabilistic	Consecutive sampling	Multicenter	Prospetively	Democratic Republic of the Congo	1965-1970	Unclear/ Not reported	Unclear/ Not reported	Unclear/ Not reported	Rural	Community-based	Not applicable (if not in the hospital)	Not applicable (if not in the hospital)	Not applicable (for animals and healty participants)	Not applicable (for animals and healty participants)	Not applicable (for animals and healty participants)	Unclear/Not reported	Neutralization test	Serum	Antibodies	At least one past contact	Humans	Apparently healthy individuals	Not applicable	Not applicable
Henderson, 1972	Cross-sectional	Non probabilistic	Consecutive sampling	Multicenter	Prospetively	Ethiopia	1965-1970	Unclear/ Not reported	Unclear/ Not reported	Unclear/ Not reported	Rural	Community-based	Not applicable (if not in the hospital)	Not applicable (if not in the hospital)	Not applicable (for animals and healty participants)	Not applicable (for animals and healty participants)	Not applicable (for animals and healty participants)	Unclear/Not reported	Neutralization test	Serum	Antibodies	At least one past contact	Humans	Apparently healthy individuals	Not applicable	Not applicable
Henderson, 1972	Cross-sectional	Non probabilistic	Consecutive sampling	Multicenter	Prospetively	Gabon	1965-1970	Unclear/ Not reported	Unclear/ Not reported	Unclear/ Not reported	Rural	Community-based	Not applicable (if not in the hospital)	Not applicable (if not in the hospital)	Not applicable (for animals and healty participants)	Not applicable (for animals and healty participants)	Not applicable (for animals and healty participants)	Unclear/Not reported	Neutralization test	Serum	Antibodies	At least one past contact	Humans	Apparently healthy individuals	Not applicable	Not applicable
Henderson, 1972	Cross-sectional	Non probabilistic	Consecutive sampling	Multicenter	Prospetively	Guinea	1965-1970	Unclear/ Not reported	Unclear/ Not reported	Unclear/ Not reported	Rural	Community-based	Not applicable (if not in the hospital)	Not applicable (if not in the hospital)	Not applicable (for animals and healty participants)	Not applicable (for animals and healty participants)	Not applicable (for animals and healty participants)	Unclear/Not reported	Neutralization test	Serum	Antibodies	At least one past contact	Humans	Apparently healthy individuals	Not applicable	Not applicable
Henderson, 1972	Cross-sectional	Non probabilistic	Consecutive sampling	Multicenter	Prospetively	Ivory Coast	1965-1970	Unclear/ Not reported	Unclear/ Not reported	Unclear/ Not reported	Rural	Community-based	Not applicable (if not in the hospital)	Not applicable (if not in the hospital)	Not applicable (for animals and healty participants)	Not applicable (for animals and healty participants)	Not applicable (for animals and healty participants)	Unclear/Not reported	Neutralization test	Serum	Antibodies	At least one past contact	Humans	Apparently healthy individuals	Not applicable	Not applicable
Henderson, 1972	Cross-sectional	Non probabilistic	Consecutive sampling	Multicenter	Prospetively	Kenya	1965-1970	Unclear/ Not reported	Unclear/ Not reported	Unclear/ Not reported	Rural	Community-based	Not applicable (if not in the hospital)	Not applicable (if not in the hospital)	Not applicable (for animals and healty participants)	Not applicable (for animals and healty participants)	Not applicable (for animals and healty participants)	Unclear/Not reported	Neutralization test	Serum	Antibodies	At least one past contact	Humans	Apparently healthy individuals	Not applicable	Not applicable
Henderson, 1972	Cross-sectional	Non probabilistic	Consecutive sampling	Multicenter	Prospetively	Liberia	1965-1970	Unclear/ Not reported	Unclear/ Not reported	Unclear/ Not reported	Rural	Community-based	Not applicable (if not in the hospital)	Not applicable (if not in the hospital)	Not applicable (for animals and healty participants)	Not applicable (for animals and healty participants)	Not applicable (for animals and healty participants)	Unclear/Not reported	Neutralization test	Serum	Antibodies	At least one past contact	Humans	Apparently healthy individuals	Not applicable	Not applicable
Henderson, 1972	Cross-sectional	Non probabilistic	Consecutive sampling	Multicenter	Prospetively	Mali	1965-1970	Unclear/ Not reported	Unclear/ Not reported	Unclear/ Not reported	Rural	Community-based	Not applicable (if not in the hospital)	Not applicable (if not in the hospital)	Not applicable (for animals and healty participants)	Not applicable (for animals and healty participants)	Not applicable (for animals and healty participants)	Unclear/Not reported	Neutralization test	Serum	Antibodies	At least one past contact	Humans	Apparently healthy individuals	Not applicable	Not applicable
Henderson, 1972	Cross-sectional	Non probabilistic	Consecutive sampling	Multicenter	Prospetively	Niger	1965-1970	Unclear/ Not reported	Unclear/ Not reported	Unclear/ Not reported	Rural	Community-based	Not applicable (if not in the hospital)	Not applicable (if not in the hospital)	Not applicable (for animals and healty participants)	Not applicable (for animals and healty participants)	Not applicable (for animals and healty participants)	Unclear/Not reported	Neutralization test	Serum	Antibodies	At least one past contact	Humans	Apparently healthy individuals	Not applicable	Not applicable
Henderson, 1972	Cross-sectional	Non probabilistic	Consecutive sampling	Multicenter	Prospetively	Nigeria	1965-1970	Unclear/ Not reported	Unclear/ Not reported	Unclear/ Not reported	Rural	Community-based	Not applicable (if not in the hospital)	Not applicable (if not in the hospital)	Not applicable (for animals and healty participants)	Not applicable (for animals and healty participants)	Not applicable (for animals and healty participants)	Unclear/Not reported	Neutralization test	Serum	Antibodies	At least one past contact	Humans	Apparently healthy individuals	Not applicable	Not applicable
Henderson, 1972	Cross-sectional	Non probabilistic	Consecutive sampling	Multicenter	Prospetively	Nigeria	1965-1970	Unclear/ Not reported	Unclear/ Not reported	Unclear/ Not reported	Rural	Community-based	Not applicable (if not in the hospital)	Not applicable (if not in the hospital)	Not applicable (for animals and healty participants)	Not applicable (for animals and healty participants)	Not applicable (for animals and healty participants)	Unclear/Not reported	Neutralization test	Serum	Antibodies	At least one past contact	Humans	High risk individuals, people with regular animals contact	Not applicable	Not applicable
Henderson, 1972	Cross-sectional	Non probabilistic	Consecutive sampling	Multicenter	Prospetively	Tanzania	1965-1970	Unclear/ Not reported	Unclear/ Not reported	Unclear/ Not reported	Rural	Community-based	Not applicable (if not in the hospital)	Not applicable (if not in the hospital)	Not applicable (for animals and healty participants)	Not applicable (for animals and healty participants)	Not applicable (for animals and healty participants)	Unclear/Not reported	Neutralization test	Serum	Antibodies	At least one past contact	Humans	Apparently healthy individuals	Not applicable	Not applicable
Henderson, 1972	Cross-sectional	Non probabilistic	Consecutive sampling	Multicenter	Prospetively	Nigeria	1965-1970	Unclear/ Not reported	Unclear/ Not reported	Unclear/ Not reported	Rural	Community-based	Not applicable (if not in the hospital)	Not applicable (if not in the hospital)	Unclear/Not reported	Unclear/Not reported	Unclear/Not reported	Unclear/Not reported	Neutralization test	Serum	Antibodies	At least one past contact	Humans	Patient with any illness	Not applicable	Not applicable
Henderson, 1972	Cross-sectional	Non probabilistic	Consecutive sampling	Multicenter	Prospetively	Nigeria	1965-1966	Unclear/ Not reported	Unclear/ Not reported	Unclear/ Not reported	Rural	Community-based	Not applicable (if not in the hospital)	Not applicable (if not in the hospital)	Unclear/Not reported	Unclear/Not reported	Unclear/Not reported	Unclear/Not reported	Neutralization test	Serum	Antibodies	At least one past contact	Humans	Patient with illnesses other than fever diseases	Not applicable	Not applicable
Ibekwe, 2012	Case-control	Non probabilistic	Consecutive sampling	Monocenter	Prospetively	Nigeria	Jul/2007-Mar/2011	Unclear/ Not reported	11-61	48.6	Unclear/Not reported	Hospital-based	Unclear/Not reported	Hospitalized	Unclear/Not reported	The initial diagnosis of acute LF was made based on the suggestive clinical features.17,18	Unclear/Not reported	Unclear/Not reported	Classical RT-PCR	Serum	RNA	Current infection	Humans	LASV suspected cases	Not applicable	Not applicable
Ibekwe, 2012	Case-control	Non probabilistic	Consecutive sampling	Monocenter	Prospetively	Nigeria	Jul/2007-Mar/2011	Unclear/ Not reported	11-61	48.6	Unclear/Not reported	Hospital-based	Unclear/Not reported	Hospitalized	Unclear/Not reported	The initial diagnosis of acute LF was made based on the suggestive clinical features.17,18	Unclear/Not reported	Unclear/Not reported	Unclear/Not reported	Serum	IgM	Recent infection	Humans	LASV suspected cases	Not applicable	Not applicable
Ipadeola, 2020	Cross-sectional	Non probabilistic	Consecutive sampling	Multicenter	Prospetively	Nigeria	Jan/2018-Oct/2019	Unclear/ Not reported	Unclear/ Not reported	Unclear/ Not reported	Unclear/Not reported	Hospital/community based	Unclear/Not reported	Unclear/Not reported	Unclear/Not reported	Unclear/Not reported	Unclear/Not reported	Unclear/Not reported	Classical RT-PCR	Serum	RNA	Current infection	Humans	LASV suspected cases	Not applicable	Not applicable

Isere, 2018	Cross-sectional	Non probabilistic	Consecutive sampling	Multicenter	Prospetively	Nigeria	Dec/2015-Apr/2016	29.3	Unclear/ Not reported	50	Urban/rural	Hospital-based	Primary hospital	Hospitalized/ambulatory	Unclear/Not reported	The definition was any illness or death following a gradual onset and one or more of the following symptoms: malaise, fever, headache, sore throat, cough, nausea, vomiting, diarrhoea, myalgia, chest pain, hearing loss and a history of contact with excreta of rodents or with a case of LF from December 1, 2015 to April 30, 2016.	Likewise, for any confirmed case or death of LF, contacts tracing and identification were carried out using the WHO definition for a contact, which was, "any person without any disease signs and symptoms but had physical contact which includes sharing the same room/bed, caring for a patient, touching body fluids, or closely participating in a burial with a case (alive or dead) or the body fluids of a case within the last three weeks from the onset of symptoms, within the hospital or community".	Unclear/Not reported	Real-time PCR	Serum	RNA	Current infection	Humans	LASV suspected cases	Not applicable	Not applicable
Ivanoff, 1982	Cross-sectional	Non probabilistic	Consecutive sampling	Multicenter	Prospetively	Gabon	Feb/1980-Mar/1980	Unclear/ Not reported	Unclear/ Not reported	Unclear/ Not reported	Urban	Hospital-based	Unclear/Not reported	Ambulatory	Not applicable (for animals and healty participants)	Not applicable (for animals and healty participants)	Not applicable (for animals and healty participants)	Unclear/Not reported	Indirect immunofluorescence assay	Serum	Antibodies	At least one past contact	Humans	Apparently healthy individuals	Not applicable	Not applicable
Ivanoff, 1982	Cross-sectional	Non probabilistic	Consecutive sampling	Multicenter	Prospetively	Gabon	Feb/1980-Mar/1980	Unclear/ Not reported	Unclear/ Not reported	Unclear/ Not reported	Urban	Community-based	Not applicable (if not in the hospital)	Not applicable (if not in the hospital)	Not applicable (for animals and healty participants)	Not applicable (for animals and healty participants)	Not applicable (for animals and healty participants)	Unclear/Not reported	Indirect immunofluorescence assay	Serum	Antibodies	At least one past contact	Other mammals	Primates	Not applicable	Not applicable
Jahrling, 1985	Cross-sectional	Non probabilistic	Consecutive sampling	Monocenter	Prospetively	Liberia	Jul/1980-Oct/1983	Unclear/ Not reported	Unclear/ Not reported	Unclear/ Not reported	Rural	Hospital-based	Unclear/Not reported	Hospitalized	Unclear/Not reported	Criteria for selection of suswcted LF patients at CLH were described by FRAME et al.; 1984).	Unclear/Not reported	Virus isolates obtained in Vero cells were identified as LV in a neutralization test using dilutions of a Lassa-immune antiserum as described (JAHRLING, 1983).	Neutralization test	Serum	Antigen	Current infection	Humans	LASV suspected cases	Not applicable	Not applicable
Johnson, 1982	Cross-sectional	Non probabilistic	Consecutive sampling	Monocenter	Prospetively	Kenya	Unclear/ Not reported	Unclear/ Not reported	Unclear/ Not reported	Unclear/ Not reported	Unclear/Not reported	Unclear/Not reported	Unclear/Not reported	Ambulatory	Not applicable (for animals and healty participants)	Not applicable (for animals and healty participants)	Not applicable (for animals and healty participants)	Unclear/Not reported	Indirect immunofluorescence assay	Serum	Antibodies	At least one past contact	Humans	High risk individuals, people with regular animals contact	Not applicable	Not applicable
Johnson, 1982	Cross-sectional	Non probabilistic	Consecutive sampling	Monocenter	Prospetively	Kenya	Unclear/ Not reported	Unclear/ Not reported	Unclear/ Not reported	Unclear/ Not reported	Unclear/Not reported	Community-based	Not applicable (if not in the hospital)	Not applicable (if not in the hospital)	Not applicable (for animals and healty participants)	Not applicable (for animals and healty participants)	Not applicable (for animals and healty participants)	Unclear/Not reported	Indirect immunofluorescence assay	Serum	Antibodies	At least one past contact	Other mammals	Primates	Not applicable	Not applicable
Johnson, 1983	Cross-sectional	Non probabilistic	Consecutive sampling	Multicenter	Prospetively	Kenya	Unclear/ Not reported	Unclear/ Not reported	1.5-70	Unclear/ Not reported	Rural	Community-based	Not applicable (if not in the hospital)	Not applicable (if not in the hospital)	Not applicable (for animals and healty participants)	Not applicable (for animals and healty participants)	Not applicable (for animals and healty participants)	Unclear/Not reported	Indirect immunofluorescence assay	Serum	Antibodies	At least one past contact	Humans	Apparently healthy individuals	Not applicable	Not applicable
Johnson, 1983	Cross-sectional	Non probabilistic	Convenience sampling	Multicenter	Prospetively	Kenya	1980-1981	Unclear/ Not reported	Unclear/ Not reported	Unclear/ Not reported	Unclear/Not reported	Unclear/Not reported	community, hospital	Unclear/Not reported	Unclear/Not reported	Unclear/Not reported	Unclear/Not reported	Unclear/Not reported	Indirect immunofluorescence assay	Serum	Antibodies	At least one past contact	Humans	Patient with any illness	Not applicable	Not applicable
Johnson, 1993	Cross-sectional	Non probabilistic	Convenience sampling	Multicenter	Prospetively	Central African Republic	Unclear/ Not reported	Unclear/ Not reported	Unclear/ Not reported	49.96	Rural	Community-based	Not applicable (if not in the hospital)	Not applicable (if not in the hospital)	Not applicable (for animals and healty participants)	Not applicable (for animals and healty participants)	Not applicable (for animals and healty participants)	Unclear/Not reported	Indirect immunofluorescence assay	Serum	Antibodies	At least one past contact	Humans	Apparently healthy individuals	Not applicable	Not applicable
Keane, 1977	Cross-sectional	Non probabilistic	Consecutive sampling	Multicenter	Retrospectively	Sierra Leone	Jan/1973-Mar/1976	Unclear/ Not reported	Unclear/ Not reported	Unclear/ Not reported	Rural	Hospital-based	Unclear/Not reported	Hospitalized	Unclear/Not reported	Unclear/Not reported	Unclear/Not reported	Unclear/Not reported	Complement fixation test, Immunoflorescent assay	Serum	Antibodies	At least one past contact	Humans	LASV suspected cases	Not applicable	Not applicable
Keenlyside, 1983	Case-control	Probabilistic	Trapping	Monocenter	Prospetively	Sierra Leone	Unclear/ Not reported	Unclear/ Not reported	Unclear/ Not reported	Unclear/ Not reported	Rural	Community-based	Not applicable (if not in the hospital)	Not applicable (if not in the hospital)	Not applicable (for animals and healty participants)	Not applicable (for animals and healty participants)	Not applicable (for animals and healty participants)	Unclear/Not reported	Culture	Organ tissue	Virus	Current infection	Rodents	Unspecified rodents	Unclear/ Not reported	Unclear/ Not reported
Keenlyside, 1983	Case-control	Probabilistic	Systematic sampling	Monocenter	Prospetively	Sierra Leone	Unclear/ Not reported	Unclear/ Not reported	Unclear/ Not reported	Unclear/ Not reported	Rural	Community-based	Not applicable (if not in the hospital)	Not applicable (if not in the hospital)	Not applicable (for animals and healty participants)	Not applicable (for animals and healty participants)	Not applicable (for animals and healty participants)	Unclear/Not reported	Indirect immunofluorescence assay	Serum	Antibodies	At least one past contact	Humans	Apparently healthy individuals	Not applicable	Not applicable
Kernéis, 2009	Cross-sectional	Probabilistic	Cluster sampling	Multicenter	Prospetively	Guinea	Apr/2000	30	1-99	44	Urban/rural	Community-based	Not applicable (if not in the hospital)	Not applicable (if not in the hospital)	Not applicable (for animals and healty participants)	Not applicable (for animals and healty participants)	Not applicable (for animals and healty participants)	Unclear/Not reported	Indirect immunofluorescence assay	Serum	IgG	At least one past contact	Humans	Apparently healthy individuals	Not applicable	Not applicable
Klempa, 2013	Cross-sectional	Non probabilistic	Consecutive sampling	Multicenter	Prospetively	Guinea	Oct/2004	Unclear/ Not reported	1-90	54.1	Rural	Community-based	Not applicable (if not in the hospital)	Not applicable (if not in the hospital)	Unclear/Not reported	Unclear/Not reported	Unclear/Not reported	Reciprocal titres of 40 by ELISA and 20 by IFA were considered as positive (Wulff & Lange 1975; Emmerich et al. 2006).	Classical RT-PCR	Serum	RNA	Current infection	Humans	Febrile patients	Not applicable	Not applicable
Klempa, 2013	Cross-sectional	Non probabilistic	Consecutive sampling	Multicenter	Prospetively	Guinea	Oct/2004	Unclear/ Not reported	1-90	54.1	Rural	Community-based	Not applicable (if not in the hospital)	Not applicable (if not in the hospital)	Unclear/Not reported	Unclear/Not reported	Unclear/Not reported	Reciprocal titres of 40 by ELISA and 20 by IFA were considered as positive (Wulff & Lange 1975; Emmerich et al. 2006).	Indirect ELISA	Serum	IgG	At least one past contact	Humans	Febrile patients	Not applicable	Not applicable
Klempa, 2013	Cross-sectional	Non probabilistic	Consecutive sampling	Multicenter	Prospetively	Guinea	Oct/2004	Unclear/ Not reported	1-90	54.1	Rural	Community-based	Not applicable (if not in the hospital)	Not applicable (if not in the hospital)	Unclear/Not reported	Unclear/Not reported	Unclear/Not reported	Reciprocal titres of 40 by ELISA and 20 by IFA were considered as positive (Wulff & Lange 1975; Emmerich et al. 2006).	Indirect immunofluorescence assay	Serum	IgG	At least one past contact	Humans	Febrile patients	Not applicable	Not applicable
Knobloch, 1982	Cross-sectional	Non probabilistic	Consecutive sampling	Multicenter	Prospetively	Liberia	Oct/1974-Dec/1974	Unclear/ Not reported	20-49	31	Rural	Hospital-based	Maternity Centre	Ambulatory	Not applicable (for animals and healty participants)	Not applicable (for animals and healty participants)	Not applicable (for animals and healty participants)	Unclear/Not reported	Complement fixation test	Serum	Antibodies	At least one past contact	Humans	Healthcare workers	Not applicable	Not applicable
Knobloch, 1982	Cross-sectional	Non probabilistic	Consecutive sampling	Multicenter	Prospetively	Liberia	Oct/1974-Dec/1974	Unclear/ Not reported	20-49	31	Rural	Hospital-based	Maternity Centre	Ambulatory	Not applicable (for animals and healty participants)	Not applicable (for animals and healty participants)	Not applicable (for animals and healty participants)	Unclear/Not reported	Indirect immunofluorescence assay	Serum	Antibodies	At least one past contact	Humans	Healthcare workers	Not applicable	Not applicable
Knobloch, 1982	Cross-sectional	Non probabilistic	Consecutive sampling	Multicenter	Prospetively	Liberia	Oct/1974-Dec/1974	Unclear/ Not reported	20-49	31	Rural	Hospital-based	Maternity Centre	Unclear/Not reported	Unclear/Not reported	Unclear/Not reported	Unclear/Not reported	Unclear/Not reported	Complement fixation test	Serum	Antibodies	At least one past contact	Humans	Patient with any illness	Not applicable	Not applicable
Knobloch, 1982	Cross-sectional	Non probabilistic	Consecutive sampling	Multicenter	Prospetively	Liberia	Oct/1974-Dec/1974	Unclear/ Not reported	20-49	31	Rural	Hospital-based	Maternity Centre	Unclear/Not reported	Unclear/Not reported	Unclear/Not reported	Unclear/Not reported	Unclear/Not reported	Indirect immunofluorescence assay	Serum	Antibodies	At least one past contact	Humans	Patient with any illness	Not applicable	Not applicable
Kouadio , 2015	Cross-sectional	Probabilistic	Trapping	Multicenter	Prospetively	Ivory Coast	Oct/2013	Unclear/ Not reported	Unclear/ Not reported	Unclear/ Not reported	Rural	Community-based	Not applicable (if not in the hospital)	Not applicable (if not in the hospital)	Not applicable (for animals and healty participants)	Not applicable (for animals and healty participants)	Not applicable (for animals and healty participants)	Unclear/Not reported	Real-time PCR	Broncho-alveolar	RNA	Current infection	Rodents	Mastomys natalensis	Animals were assigned at the genus level in the field on the basis of morphology. For 88% of them, assignment could later be refined to the species level by sequencing a fragment of the mitochondrial cytochrome b gene.	Yes
Lalis , 2012	Cross-sectional	Probabilistic	Trapping	Multicenter	Prospetively	Guinea	2002-2006	Unclear/ Not reported	Unclear/ Not reported	Unclear/ Not reported	Rural	Community-based	Not applicable (if not in the hospital)	Not applicable (if not in the hospital)	Not applicable (for animals and healty participants)	Not applicable (for animals and healty participants)	Not applicable (for animals and healty participants)	Unclear/Not reported	Real-time PCR	Liver and spleens	RNA	Current infection	Rodents	Mastomys natalensis	Cytogenetic analyses, PCR and DNA sequencing of cytochrome b were conducted for accurate identification of the species M. natalensis and M. erythroleucus since M. natalensis has been likely identified as the only host for LASV in Guinea [7].	Yes
Lecompte, 2006	Cross-sectional	Non probabilistic	Trapping	Multicenter	Prospetively	Guinea	2002-2005	Unclear/ Not reported	Unclear/ Not reported	Unclear/ Not reported	Rural	Community-based	Not applicable (if not in the hospital)	Not applicable (if not in the hospital)	Not applicable (for animals and healty participants)	Not applicable (for animals and healty participants)	Not applicable (for animals and healty participants)	Unclear/Not reported	Classical RT-PCR	Serum	RNA	Current infection	Rodents	Crocodura species	In addition, we karyotyped 12 members of the genus Mastomys in the field by using standard procedures (14). We PCR-typed all Lassa virus–positive rodents unequivocally as M. natalensis, with 1 male (no. BA686) additionally confirmed by karyotyping (2n = 32, autosomal fundamental number = 53).	Yes
Lecompte, 2006	Cross-sectional	Non probabilistic	Trapping	Multicenter	Prospetively	Guinea	2002-2005	Unclear/ Not reported	Unclear/ Not reported	Unclear/ Not reported	Rural	Community-based	Not applicable (if not in the hospital)	Not applicable (if not in the hospital)	Not applicable (for animals and healty participants)	Not applicable (for animals and healty participants)	Not applicable (for animals and healty participants)	Unclear/Not reported	Classical RT-PCR	Serum	RNA	Current infection	Rodents	Myomys daltoni	In addition, we karyotyped 12 members of the genus Mastomys in the field by using standard procedures (14). We PCR-typed all Lassa virus–positive rodents unequivocally as M. natalensis, with 1 male (no. BA686) additionally confirmed by karyotyping (2n = 32, autosomal fundamental number = 53).	Yes
Lecompte, 2006	Cross-sectional	Non probabilistic	Trapping	Multicenter	Prospetively	Guinea	2002-2005	Unclear/ Not reported	Unclear/ Not reported	Unclear/ Not reported	Rural	Community-based	Not applicable (if not in the hospital)	Not applicable (if not in the hospital)	Not applicable (for animals and healty participants)	Not applicable (for animals and healty participants)	Not applicable (for animals and healty participants)	Unclear/Not reported	Classical RT-PCR	Serum	RNA	Current infection	Rodents	Rattus rattus	In addition, we karyotyped 12 members of the genus Mastomys in the field by using standard procedures (14). We PCR-typed all Lassa virus–positive rodents unequivocally as M. natalensis, with 1 male (no. BA686) additionally confirmed by karyotyping (2n = 32, autosomal fundamental number = 53).	Yes
Lecompte, 2006	Cross-sectional	Non probabilistic	Trapping	Multicenter	Prospetively	Guinea	2002-2005	Unclear/ Not reported	Unclear/ Not reported	Unclear/ Not reported	Rural	Community-based	Not applicable (if not in the hospital)	Not applicable (if not in the hospital)	Not applicable (for animals and healty participants)	Not applicable (for animals and healty participants)	Not applicable (for animals and healty participants)	Unclear/Not reported	Classical RT-PCR	Serum	RNA	Current infection	Rodents	Mastomys natalensis	In addition, we karyotyped 12 members of the genus Mastomys in the field by using standard procedures (14). We PCR-typed all Lassa virus–positive rodents unequivocally as M. natalensis, with 1 male (no. BA686) additionally confirmed by karyotyping (2n = 32, autosomal fundamental number = 53).	Yes
Lecompte, 2006	Cross-sectional	Non probabilistic	Trapping	Multicenter	Prospetively	Guinea	2002-2005	Unclear/ Not reported	Unclear/ Not reported	Unclear/ Not reported	Rural	Community-based	Not applicable (if not in the hospital)	Not applicable (if not in the hospital)	Not applicable (for animals and healty participants)	Not applicable (for animals and healty participants)	Not applicable (for animals and healty participants)	Unclear/Not reported	Classical RT-PCR	Serum	RNA	Current infection	Rodents	Mastomys erythroleucus	In addition, we karyotyped 12 members of the genus Mastomys in the field by using standard procedures (14). We PCR-typed all Lassa virus–positive rodents unequivocally as M. natalensis, with 1 male (no. BA686) additionally confirmed by karyotyping (2n = 32, autosomal fundamental number = 53).	Yes
Lecompte, 2006	Cross-sectional	Non probabilistic	Trapping	Multicenter	Prospetively	Guinea	2002-2005	Unclear/ Not reported	Unclear/ Not reported	Unclear/ Not reported	Rural	Community-based	Not applicable (if not in the hospital)	Not applicable (if not in the hospital)	Not applicable (for animals and healty participants)	Not applicable (for animals and healty participants)	Not applicable (for animals and healty participants)	Unclear/Not reported	Classical RT-PCR	Serum	RNA	Current infection	Rodents	Lemniscomys species	In addition, we karyotyped 12 members of the genus Mastomys in the field by using standard procedures (14). We PCR-typed all Lassa virus–positive rodents unequivocally as M. natalensis, with 1 male (no. BA686) additionally confirmed by karyotyping (2n = 32, autosomal fundamental number = 53).	Yes
Lecompte, 2006	Cross-sectional	Non probabilistic	Trapping	Multicenter	Prospetively	Guinea	2002-2005	Unclear/ Not reported	Unclear/ Not reported	Unclear/ Not reported	Rural	Community-based	Not applicable (if not in the hospital)	Not applicable (if not in the hospital)	Not applicable (for animals and healty participants)	Not applicable (for animals and healty participants)	Not applicable (for animals and healty participants)	Unclear/Not reported	Classical RT-PCR	Serum	RNA	Current infection	Rodents	Lophuromys sikapusi	In addition, we karyotyped 12 members of the genus Mastomys in the field by using standard procedures (14). We PCR-typed all Lassa virus–positive rodents unequivocally as M. natalensis, with 1 male (no. BA686) additionally confirmed by karyotyping (2n = 32, autosomal fundamental number = 53).	Yes
Lecompte, 2006	Cross-sectional	Non probabilistic	Trapping	Multicenter	Prospetively	Guinea	2002-2005	Unclear/ Not reported	Unclear/ Not reported	Unclear/ Not reported	Rural	Community-based	Not applicable (if not in the hospital)	Not applicable (if not in the hospital)	Not applicable (for animals and healty participants)	Not applicable (for animals and healty participants)	Not applicable (for animals and healty participants)	Unclear/Not reported	Classical RT-PCR	Serum	RNA	Current infection	Rodents	Mus (Nannomys) species	In addition, we karyotyped 12 members of the genus Mastomys in the field by using standard procedures (14). We PCR-typed all Lassa virus–positive rodents unequivocally as M. natalensis, with 1 male (no. BA686) additionally confirmed by karyotyping (2n = 32, autosomal fundamental number = 53).	Yes
Lecompte, 2006	Cross-sectional	Non probabilistic	Trapping	Multicenter	Prospetively	Guinea	2002-2005	Unclear/ Not reported	Unclear/ Not reported	Unclear/ Not reported	Rural	Community-based	Not applicable (if not in the hospital)	Not applicable (if not in the hospital)	Not applicable (for animals and healty participants)	Not applicable (for animals and healty participants)	Not applicable (for animals and healty participants)	Unclear/Not reported	Classical RT-PCR	Serum	RNA	Current infection	Rodents	Praomys cf. rostratus	In addition, we karyotyped 12 members of the genus Mastomys in the field by using standard procedures (14). We PCR-typed all Lassa virus–positive rodents unequivocally as M. natalensis, with 1 male (no. BA686) additionally confirmed by karyotyping (2n = 32, autosomal fundamental number = 53).	Yes
Lecompte, 2006	Cross-sectional	Non probabilistic	Trapping	Multicenter	Prospetively	Guinea	2002-2005	Unclear/ Not reported	Unclear/ Not reported	Unclear/ Not reported	Rural	Community-based	Not applicable (if not in the hospital)	Not applicable (if not in the hospital)	Not applicable (for animals and healty participants)	Not applicable (for animals and healty participants)	Not applicable (for animals and healty participants)	Unclear/Not reported	Classical RT-PCR	Serum	RNA	Current infection	Rodents	Tatera cf. guinea	In addition, we karyotyped 12 members of the genus Mastomys in the field by using standard procedures (14). We PCR-typed all Lassa virus–positive rodents unequivocally as M. natalensis, with 1 male (no. BA686) additionally confirmed by karyotyping (2n = 32, autosomal fundamental number = 53).	Yes
Leski (GPC gene), 2015	Cross-sectional	Non probabilistic	Trapping	Multicenter	Prospetively	Sierra Leone	2009	Unclear/ Not reported	Unclear/ Not reported	Unclear/ Not reported	Unclear/Not reported	Community-based	Not applicable (if not in the hospital)	Not applicable (if not in the hospital)	Not applicable (for animals and healty participants)	Not applicable (for animals and healty participants)	Not applicable (for animals and healty participants)	Unclear/Not reported	Classical RT-PCR	Spleen sections	RNA	Current infection	Rodents	Mastomys species	Rodents were identified to the genus level in the field. Animals identified as Mastomys sp. were further identified down to species level by using molecular methods as described previously (27).	Yes
Leski (NP gene), 2015	Cross-sectional	Non probabilistic	Trapping	Multicenter	Prospetively	Sierra Leone	2009	Unclear/ Not reported	Unclear/ Not reported	Unclear/ Not reported	Unclear/Not reported	Community-based	Not applicable (if not in the hospital)	Not applicable (if not in the hospital)	Not applicable (for animals and healty participants)	Not applicable (for animals and healty participants)	Not applicable (for animals and healty participants)	Unclear/Not reported	Classical RT-PCR	Spleen sections	RNA	Current infection	Rodents	Mastomys species	Rodents were identified to the genus level in the field. Animals identified as Mastomys sp. were further identified down to species level by using molecular methods as described previously (27).	Yes
Leski (NP gene), 2015	Cross-sectional	Non probabilistic	Trapping	Multicenter	Prospetively	Sierra Leone	2009	Unclear/ Not reported	Unclear/ Not reported	Unclear/ Not reported	Unclear/Not reported	Community-based	Not applicable (if not in the hospital)	Not applicable (if not in the hospital)	Not applicable (for animals and healty participants)	Not applicable (for animals and healty participants)	Not applicable (for animals and healty participants)	Unclear/Not reported	Classical RT-PCR	Spleen sections	RNA	Current infection	Rodents	Mastomys species	Rodents were identified to the genus level in the field. Animals identified as Mastomys sp. were further identified down to species level by using molecular methods as described previously (27).	Yes
Leski, 2015	Cross-sectional	Non probabilistic	Trapping	Multicenter	Prospetively	Sierra Leone	2009	Unclear/ Not reported	Unclear/ Not reported	Unclear/ Not reported	Unclear/Not reported	Community-based	Not applicable (if not in the hospital)	Not applicable (if not in the hospital)	Not applicable (for animals and healty participants)	Not applicable (for animals and healty participants)	Not applicable (for animals and healty participants)	Unclear/Not reported	RPM-TEI Microarray Analysis	Unclear/Not reported	RNA	Current infection	Rodents	Mastomys species	Rodents were identified to the genus level in the field. Animals identified as Mastomys sp. were further identified down to species level by using molecular methods as described previously (27).	Yes
Li, 2016	Cross-sectional	Non probabilistic	Consecutive sampling	Monocenter	Prospetively	Sierra Leone	Nov/2014-Mar/2015	42.3	2-75	58.27	Urban	Hospital-based	Primary hospital	Hospitalized	Suspected cases of EVD were defined according to World Health Organization (WHO) [22]	Unclear/Not reported	Unclear/Not reported	Unclear/Not reported	Indirect ELISA, Direct ELISA	Serum	Antigen, IgM	Current infection	Humans	Patient with hemorrhagic fever	Not applicable	Not applicable
Lukashevich, 1993	Cross-sectional	Non probabilistic	Consecutive sampling	Multicenter	Prospetively	Guinea	Jun/1990-Jan/1992	Unclear/ Not reported	Unclear/ Not reported	Unclear/ Not reported	Urban/rural	Hospital/community based	Tertiary hospital	Ambulatory	Not applicable (for animals and healty participants)	Not applicable (for animals and healty participants)	Not applicable (for animals and healty participants)	Unclear/Not reported	Indirect ELISA	Serum	IgG	At least one past contact	Humans	Apparently healthy individuals	Not applicable	Not applicable
Lukashevich, 1993	Cross-sectional	Non probabilistic	Consecutive sampling	Multicenter	Prospetively	Guinea	Jun/1990-Jan/1992	Unclear/ Not reported	Unclear/ Not reported	Unclear/ Not reported	Urban/rural	Hospital/community based	Tertiary hospital	Ambulatory	Not applicable (for animals and healty participants)	Not applicable (for animals and healty participants)	Not applicable (for animals and healty participants)	Unclear/Not reported	Indirect immunofluorescence assay	Serum	Antibodies	At least one past contact	Humans	Healthcare workers	Not applicable	Not applicable
Lukashevich, 1993	Cross-sectional	Non probabilistic	Consecutive sampling	Multicenter	Prospetively	Guinea	Jun/1990-Jan/1992	Unclear/ Not reported	Unclear/ Not reported	Unclear/ Not reported	Urban/rural	Hospital/community based	Tertiary hospital	Ambulatory	Unclear/Not reported	Unclear/Not reported	Unclear/Not reported	Unclear/Not reported	Indirect ELISA	Serum	Antibodies	At least one past contact	Humans	Febrile patients	Not applicable	Not applicable
Maigari, 2018	Cross-sectional	Non probabilistic	Consecutive sampling	Monocenter	Retrospectively	Nigeria	Oct/2016-Apr/2018	Unclear/ Not reported	Unclear/ Not reported	Unclear/ Not reported	Unclear/Not reported	Hospital-based	Tertiary hospital	Hospitalized	Unclear/Not reported	Unclear/Not reported	Unclear/Not reported	Unclear/Not reported	Classical RT-PCR	Unclear/Not reported	RNA	Current infection	Humans	LASV suspected cases	Not applicable	Not applicable
Mariën, 2017	Cross-sectional	Non probabilistic	Trapping	Multicenter	Prospetively	Guinea	2003-2015, 2008-2012	Unclear/ Not reported	Unclear/ Not reported	Unclear/ Not reported	Rural	Community-based	Not applicable (if not in the hospital)	Not applicable (if not in the hospital)	Not applicable (for animals and healty participants)	Not applicable (for animals and healty participants)	Not applicable (for animals and healty participants)	Unclear/Not reported	Real-time PCR	Serum	RNA	Current infection	Rodents	Unspecified rodents	Unclear/ Not reported	Unclear/ Not reported
Mariën, 2017	Cross-sectional	Non probabilistic	Trapping	Multicenter	Prospetively	Guinea	2003-2015, 2008-2012	Unclear/ Not reported	Unclear/ Not reported	Unclear/ Not reported	Rural	Community-based	Not applicable (if not in the hospital)	Not applicable (if not in the hospital)	Not applicable (for animals and healty participants)	Not applicable (for animals and healty participants)	Not applicable (for animals and healty participants)	Unclear/Not reported	Indirect immunofluorescence assay	Serum	Antibodies	At least one past contact	Rodents	Unspecified rodents	Unclear/ Not reported	Unclear/ Not reported
Mathiot, 1989	Cross-sectional	Non probabilistic	Consecutive sampling	Multicenter	Prospetively	Madagascar	Unclear/ Not reported	Unclear/ Not reported	Unclear/ Not reported	Unclear/ Not reported	Urban/rural	Community-based	Not applicable (if not in the hospital)	Not applicable (if not in the hospital)	Not applicable (for animals and healty participants)	Not applicable (for animals and healty participants)	Not applicable (for animals and healty participants)	Unclear/Not reported	Indirect immunofluorescence assay	Serum	Antibodies	At least one past contact	Humans	Apparently healthy individuals	Not applicable	Not applicable
McCarthy, 1996	Case-control	Non probabilistic	Consecutive sampling	Monocenter	Prospetively	Sudan	Oct/1988-Nov/1988	20	1-89	74	Urban	Hospital-based	Tertiary hospital	Ambulatory	A case of acute fever was defined as any person with a temperature of 100°F or greater upon presentation and a history of fever of no more than 5 days duration.	Unclear/Not reported	Unclear/Not reported	Unclear/Not reported	Indirect immunofluorescence assay	Serum	IgG	At least one past contact	Humans	Apparently healthy individuals, Patient with any illness	Not applicable	Not applicable
McCormick, 1987	Cross-sectional	Probabilistic	Simple random sampling	Multicenter	Prospetively	Sierra Leone	Unclear/ Not reported	Unclear/ Not reported	Unclear/ Not reported	Unclear/ Not reported	Rural	Community-based	Not applicable (if not in the hospital)	Not applicable (if not in the hospital)	Not applicable (for animals and healty participants)	Not applicable (for animals and healty participants)	Not applicable (for animals and healty participants)	Unclear/Not reported	Indirect immunofluorescence assay	Serum	Antibodies	At least one past contact	Humans	Apparently healthy individuals	Not applicable	Not applicable
McCormick, 1987	Cross-sectional	Probabilistic	Trapping	Multicenter	Prospetively	Sierra Leone	Unclear/ Not reported	Unclear/ Not reported	Unclear/ Not reported	Unclear/ Not reported	Rural	Community-based	Not applicable (if not in the hospital)	Not applicable (if not in the hospital)	Not applicable (for animals and healty participants)	Not applicable (for animals and healty participants)	Not applicable (for animals and healty participants)	Unclear/Not reported	Indirect immunofluorescence assay	Serum	Antibodies	At least one past contact	Rodents	Unspecified rodents	Unclear/ Not reported	Unclear/ Not reported
McCormick, 1987	Case-control	Non probabilistic	Consecutive sampling	Multicenter	Prospetively	Sierra Leone	Feb/1977-Jan/1979	Unclear/ Not reported	> 10	43.5	Rural	Hospital-based	Unclear/Not reported	Hospitalized	Unclear/Not reported	Unclear/Not reported	Unclear/Not reported	For this study, a case of Lassa fever was defined as an adult with a febrile illness (oral or axillary temperature ~38 C) and one or more of the following criteria: (1) isolation of Lassa virus from serum or other body fluid or organ [9]; (2) seroconversion to Lassa virus (as measured by immunofluorescent-antibody [IFA] test), with titers of antibody rising from <1:4 to ~1 :16 [12]; or (3) an IgG titer (by IFA) ~1:256 on admission and a Lassa antigen-specific IgM titer (by IFA) ~1:4.	Indirect immunofluorescence assay	Serum, Urine	IgM and IgG	Recent infection	Humans	Febrile patients	Not applicable	Not applicable
Meunier, 1987	Cross-sectional	Non probabilistic	Consecutive sampling	Multicenter	Prospetively	Central African Republic	1984-1985	Unclear/ Not reported	Unclear/ Not reported	Unclear/ Not reported	Rural	Community-based	Not applicable (if not in the hospital)	Not applicable (if not in the hospital)	Not applicable (for animals and healty participants)	Not applicable (for animals and healty participants)	Not applicable (for animals and healty participants)	Unclear/Not reported	Indirect immunofluorescence assay	Serum	Antibodies	At least one past contact	Humans	Apparently healthy individuals	Not applicable	Not applicable
Monath, 1973	Cross-sectional	Non probabilistic	Convenience sampling	Monocenter	Prospetively	Liberia	Mar/1972-Apr/1972	Unclear/ Not reported	8-49	Unclear/ Not reported	Rural	Community-based	Not applicable (if not in the hospital)	Ambulatory	Not applicable (for animals and healty participants)	Not applicable (for animals and healty participants)	Not applicable (for animals and healty participants)	Unclear/Not reported	Complement fixation test	Pharyngeal, Serum, Urine	Antibodies	At least one past contact	Humans	Apparently healthy individuals	Not applicable	Not applicable
Monath, 1973	Hospital outbreak	Non probabilistic	Convenience sampling	Monocenter	Prospetively	Liberia	Mar/1972-Apr/1972	Unclear/ Not reported	8-49	Unclear/ Not reported	Rural	Community-based	Not applicable (if not in the hospital)	Ambulatory	Not applicable (for animals and healty participants)	Not applicable (for animals and healty participants)	Not applicable (for animals and healty participants)	Unclear/Not reported	Complement fixation test	Pharyngeal, Serum, Urine	Antibodies	At least one past contact	Humans	Healthcare workers	Not applicable	Not applicable
Monath, 1973	Hospital outbreak	Non probabilistic	Convenience sampling	Monocenter	Prospetively	Liberia	Mar/1972-Apr/1972	Unclear/ Not reported	8-49	Unclear/ Not reported	Rural	Community-based	Not applicable (if not in the hospital)	Ambulatory	Unclear/Not reported	Unclear/Not reported	Unclear/Not reported	Unclear/Not reported	Complement fixation test	Pharyngeal, Serum, Urine	Antibodies	At least one past contact	Humans	LASV positive case contact	Not applicable	Not applicable
Monath, 1974	Cross-sectional	Non probabilistic	Trapping	Multicenter	Prospetively	Sierra Leone	1972	Unclear/ Not reported	Unclear/ Not reported	Unclear/ Not reported	Rural	Community-based	Not applicable (if not in the hospital)	Not applicable (if not in the hospital)	Not applicable (for animals and healty participants)	Not applicable (for animals and healty participants)	Not applicable (for animals and healty participants)	Unclear/Not reported	Culture	Serum, Urine, Organ tissue, ectoparasites	Virus	Current infection	Rodents	Unspecified rodents	Unclear/ Not reported	Unclear/ Not reported
Monath, 1974	Cross-sectional	Non probabilistic	Trapping	Multicenter	Prospetively	Sierra Leone	1972	Unclear/ Not reported	Unclear/ Not reported	Unclear/ Not reported	Rural	Community-based	Not applicable (if not in the hospital)	Not applicable (if not in the hospital)	Not applicable (for animals and healty participants)	Not applicable (for animals and healty participants)	Not applicable (for animals and healty participants)	Unclear/Not reported	Culture	Serum, Urine, Organ tissue, ectoparasites	Virus	Current infection	Rodents	Unspecified rodents	Unclear/ Not reported	Unclear/ Not reported
Monath, 1974	Cross-sectional	Non probabilistic	Trapping	Multicenter	Prospetively	Sierra Leone	1972	Unclear/ Not reported	Unclear/ Not reported	Unclear/ Not reported	Rural	Community-based	Not applicable (if not in the hospital)	Not applicable (if not in the hospital)	Not applicable (for animals and healty participants)	Not applicable (for animals and healty participants)	Not applicable (for animals and healty participants)	Unclear/Not reported	Culture	Serum, Urine, Organ tissue, ectoparasites	Virus	Current infection	Other mammals	Bats	Not applicable	Not applicable
Monath, 1974	Cross-sectional	Non probabilistic	Trapping	Multicenter	Prospetively	Sierra Leone	1972	Unclear/ Not reported	Unclear/ Not reported	Unclear/ Not reported	Rural	Community-based	Not applicable (if not in the hospital)	Not applicable (if not in the hospital)	Not applicable (for animals and healty participants)	Not applicable (for animals and healty participants)	Not applicable (for animals and healty participants)	Unclear/Not reported	Culture	Serum, Urine, Organ tissue, ectoparasites	Virus	Current infection	Other mammals	Insectivora, Crocidura spp.	Not applicable	Not applicable
Nakounné, 2000	Cross-sectional	Non probabilistic	Consecutive sampling	Multicenter	Prospetively	Central African Republic	1992-1997	Unclear/ Not reported	Unclear/ Not reported	Unclear/ Not reported	Rural	Community-based	Not applicable (if not in the hospital)	Not applicable (if not in the hospital)	Not applicable (for animals and healty participants)	Not applicable (for animals and healty participants)	Not applicable (for animals and healty participants)	Les sérums dont le titre était supérieur à 16 ont été considérés comme positif.	Indirect immunofluorescence assay	Serum	IgG	At least one past contact	Humans	Apparently healthy individuals	Not applicable	Not applicable
Nakounné, 2000	Cross-sectional	Non probabilistic	Consecutive sampling	Multicenter	Prospetively	Cameroon	1998	Unclear/ Not reported	Unclear/ Not reported	Unclear/ Not reported	Rural	Community-based	Not applicable (if not in the hospital)	Not applicable (if not in the hospital)	Unclear/Not reported	Unclear/Not reported	Unclear/Not reported	Les sérums dont le titre était supérieur à 16 ont été considérés comme positif.	Indirect immunofluorescence assay	Serum	IgG	At least one past contact	Humans	Patient with illnesses other than fever diseases	Not applicable	Not applicable

Nakounné, 2000	Cross-sectional	Non probabilistic	Consecutive sampling	Multicenter	Prospetively	Central African Republic	1998	Unclear/ Not reported	Unclear/ Not reported	Unclear/ Not reported	Rural	Community-based	Not applicable (if not in the hospital)	Not applicable (if not in the hospital)	Unclear/Not reported	Unclear/Not reported	Unclear/Not reported	Les sérums dont le titre était supérieur à 16 ont été considérés comme positif.	Indirect immunofluorescence assay	Serum	IgG	At least one past contact	Humans	Febrile patients	Not applicable	Not applicable
Nakounné, 2000	Cross-sectional	Non probabilistic	Consecutive sampling	Multicenter	Prospetively	Democratic Republic of the Congo	1990	Unclear/ Not reported	Unclear/ Not reported	Unclear/ Not reported	Rural	Community-based	Not applicable (if not in the hospital)	Not applicable (if not in the hospital)	Unclear/Not reported	Unclear/Not reported	Unclear/Not reported	Les sérums dont le titre était supérieur à 16 ont été considérés comme positif.	Indirect immunofluorescence assay	Serum	IgG	At least one past contact	Humans	Patient with illnesses other than fever diseases	Not applicable	Not applicable
Nimo-Paintsil, 2019	Cross-sectional	Non probabilistic	Consecutive sampling	Multicenter	Prospetively	Ghana	Aug/2010-Aug/2011	41.4	18-98	45.4	Rural	Community-based	Not applicable (if not in the hospital)	Not applicable (if not in the hospital)	Not applicable (for animals and healty participants)	Not applicable (for animals and healty participants)	Not applicable (for animals and healty participants)	Serum was considered positive for anti-LASV IgG when the optical density (OD) was greater than 0.2 over background [determined by subtraction of the average of OD values of the LASV (+) and (-) slurry of the same sample].	Indirect ELISA	Serum	IgG	At least one past contact	Humans	Apparently healthy individuals	Not applicable	Not applicable
Ogunro, 2019	Cross-sectional	Non probabilistic	Convenience sampling	Multicenter	Prospetively	Nigeria	Aug/2015-Aug/2018	Unclear/ Not reported	Unclear/ Not reported	Unclear/ Not reported	Urban	Community-based	Not applicable (if not in the hospital)	Not applicable (if not in the hospital)	Not applicable (for animals and healty participants)	Not applicable (for animals and healty participants)	Not applicable (for animals and healty participants)	Unclear/Not reported	Direct ELISA	Serum	Antigen	Current infection	Other mammals	Monkeys	Not applicable	Not applicable
Ogunro, 2019	Cross-sectional	Non probabilistic	Convenience sampling	Multicenter	Prospetively	Nigeria	Aug/2015-Aug/2018	Unclear/ Not reported	Unclear/ Not reported	Unclear/ Not reported	Urban	Community-based	Not applicable (if not in the hospital)	Not applicable (if not in the hospital)	Not applicable (for animals and healty participants)	Not applicable (for animals and healty participants)	Not applicable (for animals and healty participants)	Unclear/Not reported	Indirect ELISA	Serum	IgM	Recent infection	Other mammals	Monkeys	Not applicable	Not applicable
Ogunro, 2019	Cross-sectional	Non probabilistic	Convenience sampling	Multicenter	Prospetively	Nigeria	Aug/2015-Aug/2018	Unclear/ Not reported	Unclear/ Not reported	Unclear/ Not reported	Urban	Community-based	Not applicable (if not in the hospital)	Not applicable (if not in the hospital)	Not applicable (for animals and healty participants)	Not applicable (for animals and healty participants)	Not applicable (for animals and healty participants)	Unclear/Not reported	Indirect ELISA	Serum	IgG	At least one past contact	Other mammals	Monkeys	Not applicable	Not applicable
O'Hearn, 2016	Cross-sectional	Non probabilistic	Convenience sampling	Multicenter	Prospetively	Sierra Leone	2007-2014	Unclear/ Not reported	Unclear/ Not reported	Unclear/ Not reported	Urban	Hospital-based	Emergency department	Unclear/Not reported	Unclear/Not reported	Unclear/Not reported	Unclear/Not reported	Unclear/Not reported	Luminex Mag- Pix	Serum	IgG	At least one past contact	Humans	LASV suspected cases	Not applicable	Not applicable
Okogbenin, 2019	Cohort (Baseline data)	Non probabilistic	Consecutive sampling	Monocenter	Retrospectively	Nigeria	Jan/2009-Mar/2018	28.1	16 - 39	0	Urban	Hospital-based	Tertiary hospital	Ambulatory	Not applicable (for animals and healty participants)	Not applicable (for animals and healty participants)	Not applicable (for animals and healty participants)	Unclear/Not reported	Classical RT-PCR	Serum	RNA	Current infection	Humans	Pregnant women	Not applicable	Not applicable
Okokhere, 2018	Cohort (Baseline data)	Non probabilistic	Consecutive sampling	Monocenter	Retrospectively	Nigeria	Jan/2011-Nov/2015	35	Unclear/ Not reported	58.4	Urban	Hospital-based	Tertiary hospital	Hospitalized	We identified clinical and laboratory features significantly associated with LF outcome (Tables 1 and and 2, Suppl. Figure 3). Severe central nervous system (CNS) symptoms (coma, seizure; irrational talk/behavior, altered sensorium, tremors, and disorientation/confusion: which suggest encephalitis, meningitis, or encephalopathy), face and neck (F/N) swelling, jaundice, bleeding, hematuria, proteinuria, and non-severe CNS symptoms (dizziness, lethargy, drowsiness) are the clinical features associated with outcome at P<0.05. These designations of severe and non-severe CNS features were based on known symptoms and signs of viral encephalitis, meningitis, and encephalopathy, and our previous observations18,19 at ISTH that certain CNS features were associated with excess mortality while others were not. Overall incidence of severe and non-severe CNS manifestations at presentation was 30% (84/284).	Unclear/Not reported	Unclear/Not reported	Unclear/Not reported	Classical RT-PCR	Serum	RNA	Current infection	Humans	LASV suspected cases	Not applicable	Not applicable
Okoror, 2005	Cross-sectional	Non probabilistic	Trapping	Multicenter	Retrospectively	Nigeria	Aug/1999-Nov/1999, Nov2000-Feb/2001, Jul/2002-Dec/2002	Unclear/ Not reported	Unclear/ Not reported	Unclear/ Not reported	Urban/rural	Community-based	Not applicable (if not in the hospital)	Not applicable (if not in the hospital)	Not applicable (for animals and healty participants)	Not applicable (for animals and healty participants)	Not applicable (for animals and healty participants)	Unclear/Not reported	Complement fixation test	Serum	Antibodies	At least one past contact	Rodents	Unspecified rodents	Unclear/ Not reported	Unclear/ Not reported
Okoror, 2005	Cross-sectional	Non probabilistic	Trapping	Multicenter	Retrospectively	Nigeria	Aug/1999-Nov/1999, Nov2000-Feb/2001, Jul/2002-Dec/2002	Unclear/ Not reported	Unclear/ Not reported	Unclear/ Not reported	Urban/rural	Community-based	Not applicable (if not in the hospital)	Not applicable (if not in the hospital)	Not applicable (for animals and healty participants)	Not applicable (for animals and healty participants)	Not applicable (for animals and healty participants)	Unclear/Not reported	Complement fixation test	Serum	Antibodies	At least one past contact	Rodents	Unspecified rodents	Unclear/ Not reported	Unclear/ Not reported
Olayemi, 2016	Cross-sectional	Non probabilistic	Trapping	Multicenter	Prospetively	Guinea	Oct/2008-May/2014	Unclear/ Not reported	Unclear/ Not reported	Unclear/ Not reported	Urban/rural	Community-based	Not applicable (if not in the hospital)	Not applicable (if not in the hospital)	Not applicable (for animals and healty participants)	Not applicable (for animals and healty participants)	Not applicable (for animals and healty participants)	Unclear/Not reported	Classical RT-PCR	Serum	RNA	Current infection	Rodents	Mastomys erythroleucus	Cytochrome b gene sequencing of all Lassa virus-positive rodents unambiguously identified them as Hylomyscus pamfi and Mastomys erythroleucus. Additionally, all Mastomys from Nigeria were sequenced for cytochrome b to exactly distinguish specimens of M. natalensis from M. erythroleucus.	Yes
Olayemi, 2016	Cross-sectional	Non probabilistic	Trapping	Multicenter	Prospetively	Nigeria	Oct/2008-May/2014	Unclear/ Not reported	Unclear/ Not reported	Unclear/ Not reported	Urban/rural	Community-based	Not applicable (if not in the hospital)	Not applicable (if not in the hospital)	Not applicable (for animals and healty participants)	Not applicable (for animals and healty participants)	Not applicable (for animals and healty participants)	Unclear/Not reported	Classical RT-PCR	Serum	RNA	Current infection	Rodents	Mastomys natalensis	Cytochrome b gene sequencing of all Lassa virus-positive rodents unambiguously identified them as Hylomyscus pamfi and Mastomys erythroleucus. Additionally, all Mastomys from Nigeria were sequenced for cytochrome b to exactly distinguish specimens of M. natalensis from M. erythroleucus.	Yes
Olayemi, 2016	Cross-sectional	Non probabilistic	Trapping	Multicenter	Prospetively	Nigeria	Oct/2008-May/2014	Unclear/ Not reported	Unclear/ Not reported	Unclear/ Not reported	Urban/rural	Community-based	Not applicable (if not in the hospital)	Not applicable (if not in the hospital)	Not applicable (for animals and healty participants)	Not applicable (for animals and healty participants)	Not applicable (for animals and healty participants)	Unclear/Not reported	Classical RT-PCR	Serum	RNA	Current infection	Rodents	Mastomys erythroleucus	Cytochrome b gene sequencing of all Lassa virus-positive rodents unambiguously identified them as Hylomyscus pamfi and Mastomys erythroleucus. Additionally, all Mastomys from Nigeria were sequenced for cytochrome b to exactly distinguish specimens of M. natalensis from M. erythroleucus.	Yes
Olayemi, 2016	Cross-sectional	Non probabilistic	Trapping	Multicenter	Prospetively	Nigeria	Oct/2008-May/2014	Unclear/ Not reported	Unclear/ Not reported	Unclear/ Not reported	Urban/rural	Community-based	Not applicable (if not in the hospital)	Not applicable (if not in the hospital)	Not applicable (for animals and healty participants)	Not applicable (for animals and healty participants)	Not applicable (for animals and healty participants)	Unclear/Not reported	Classical RT-PCR	Serum	RNA	Current infection	Rodents	Hylomyscus pamfi	Cytochrome b gene sequencing of all Lassa virus-positive rodents unambiguously identified them as Hylomyscus pamfi and Mastomys erythroleucus. Additionally, all Mastomys from Nigeria were sequenced for cytochrome b to exactly distinguish specimens of M. natalensis from M. erythroleucus.	Yes
Olayemi, 2016	Cross-sectional	Non probabilistic	Trapping	Multicenter	Prospetively	Nigeria	Jan/2011-Mar/2013	Unclear/ Not reported	Unclear/ Not reported	Unclear/ Not reported	Urban/rural	Community-based	Not applicable (if not in the hospital)	Not applicable (if not in the hospital)	Not applicable (for animals and healty participants)	Not applicable (for animals and healty participants)	Not applicable (for animals and healty participants)	Unclear/Not reported	Classical RT-PCR	Serum	RNA	Current infection	Rodents	Mastomys natalensis	Cytochrome b gene sequencing of all Lassa virus-positive rodents unambiguously identified them as Hylomyscus pamfi and Mastomys erythroleucus. Additionally, all Mastomys from Nigeria were sequenced for cytochrome b to exactly distinguish specimens of M. natalensis from M. erythroleucus.	Yes
Olayemi, 2018	Cross-sectional	Probabilistic	Trapping	Multicenter	Prospetively	Nigeria	Jan/2011-Oct/2012, Nov/2015	Unclear/ Not reported	Unclear/ Not reported	Unclear/ Not reported	Urban/rural	Community-based	Not applicable (if not in the hospital)	Not applicable (if not in the hospital)	Not applicable (for animals and healty participants)	Not applicable (for animals and healty participants)	Not applicable (for animals and healty participants)	Unclear/Not reported	Classical RT-PCR	Serum	RNA	Current infection	Rodents	Rattus rattus	Morphological identification of small mammals was done using external criteria such as fur color, nipple formula, and standard measurements: body weight, head-and-body length, tail length, hindfoot length and ear length [21]. Molecular identification was facilitated by DNA cytochrome b sequencing, especially for the Mastomys spp. and other sibling species such as those within Mus (Nannomys) spp. We did not designate specimens from Crocidura to species level because, even with the benefit of cytochrome b sequencing, the taxonomy of this genus is not well established. For example, the species currently known as C. olivieri (for which a large number of specimens were morphologically identified in our collection) has very recently been described as polyphyletic [22].	Yes
Olayemi, 2018	Cross-sectional	Probabilistic	Trapping	Multicenter	Prospetively	Nigeria	Jan/2011-Oct/2012, Nov/2015	Unclear/ Not reported	Unclear/ Not reported	Unclear/ Not reported	Urban/rural	Community-based	Not applicable (if not in the hospital)	Not applicable (if not in the hospital)	Not applicable (for animals and healty participants)	Not applicable (for animals and healty participants)	Not applicable (for animals and healty participants)	Unclear/Not reported	Classical RT-PCR	Serum	RNA	Current infection	Rodents	Praomys daltoni	Morphological identification of small mammals was done using external criteria such as fur color, nipple formula, and standard measurements: body weight, head-and-body length, tail length, hindfoot length and ear length [21]. Molecular identification was facilitated by DNA cytochrome b sequencing, especially for the Mastomys spp. and other sibling species such as those within Mus (Nannomys) spp. We did not designate specimens from Crocidura to species level because, even with the benefit of cytochrome b sequencing, the taxonomy of this genus is not well established. For example, the species currently known as C. olivieri (for which a large number of specimens were morphologically identified in our collection) has very recently been described as polyphyletic [22].	Yes
Olayemi, 2018	Cross-sectional	Probabilistic	Trapping	Multicenter	Prospetively	Nigeria	Jan/2011-Oct/2012, Nov/2015	Unclear/ Not reported	Unclear/ Not reported	Unclear/ Not reported	Urban/rural	Community-based	Not applicable (if not in the hospital)	Not applicable (if not in the hospital)	Not applicable (for animals and healty participants)	Not applicable (for animals and healty participants)	Not applicable (for animals and healty participants)	Unclear/Not reported	Classical RT-PCR	Serum	RNA	Current infection	Rodents	Mastomys natalensis	Morphological identification of small mammals was done using external criteria such as fur color, nipple formula, and standard measurements: body weight, head-and-body length, tail length, hindfoot length and ear length [21]. Molecular identification was facilitated by DNA cytochrome b sequencing, especially for the Mastomys spp. and other sibling species such as those within Mus (Nannomys) spp. We did not designate specimens from Crocidura to species level because, even with the benefit of cytochrome b sequencing, the taxonomy of this genus is not well established. For example, the species currently known as C. olivieri (for which a large number of specimens were morphologically identified in our collection) has very recently been described as polyphyletic [22].	Yes
Olayemi, 2018	Cross-sectional	Probabilistic	Trapping	Multicenter	Prospetively	Nigeria	Jan/2011-Oct/2012, Nov/2015	Unclear/ Not reported	Unclear/ Not reported	Unclear/ Not reported	Urban/rural	Community-based	Not applicable (if not in the hospital)	Not applicable (if not in the hospital)	Not applicable (for animals and healty participants)	Not applicable (for animals and healty participants)	Not applicable (for animals and healty participants)	Unclear/Not reported	Classical RT-PCR	Serum	RNA	Current infection	Rodents	Mastomys erythroleucus	Morphological identification of small mammals was done using external criteria such as fur color, nipple formula, and standard measurements: body weight, head-and-body length, tail length, hindfoot length and ear length [21]. Molecular identification was facilitated by DNA cytochrome b sequencing, especially for the Mastomys spp. and other sibling species such as those within Mus (Nannomys) spp. We did not designate specimens from Crocidura to species level because, even with the benefit of cytochrome b sequencing, the taxonomy of this genus is not well established. For example, the species currently known as C. olivieri (for which a large number of specimens were morphologically identified in our collection) has very recently been described as polyphyletic [22].	Yes
Olayemi, 2018	Cross-sectional	Probabilistic	Trapping	Multicenter	Prospetively	Nigeria	Jan/2011-Oct/2012, Nov/2015	Unclear/ Not reported	Unclear/ Not reported	Unclear/ Not reported	Urban/rural	Community-based	Not applicable (if not in the hospital)	Not applicable (if not in the hospital)	Not applicable (for animals and healty participants)	Not applicable (for animals and healty participants)	Not applicable (for animals and healty participants)	Unclear/Not reported	Classical RT-PCR	Serum	RNA	Current infection	Rodents	Gerbilliscus kempi	Morphological identification of small mammals was done using external criteria such as fur color, nipple formula, and standard measurements: body weight, head-and-body length, tail length, hindfoot length and ear length [21]. Molecular identification was facilitated by DNA cytochrome b sequencing, especially for the Mastomys spp. and other sibling species such as those within Mus (Nannomys) spp. We did not designate specimens from Crocidura to species level because, even with the benefit of cytochrome b sequencing, the taxonomy of this genus is not well established. For example, the species currently known as C. olivieri (for which a large number of specimens were morphologically identified in our collection) has very recently been described as polyphyletic [22].	Yes
Olayemi, 2018	Cross-sectional	Probabilistic	Trapping	Multicenter	Prospetively	Nigeria	Jan/2011-Oct/2012, Nov/2015	Unclear/ Not reported	Unclear/ Not reported	Unclear/ Not reported	Urban/rural	Community-based	Not applicable (if not in the hospital)	Not applicable (if not in the hospital)	Not applicable (for animals and healty participants)	Not applicable (for animals and healty participants)	Not applicable (for animals and healty participants)	Unclear/Not reported	Indirect immunofluorescence assay	Serum	IgG	At least one past contact	Rodents	Gerbilliscus kempi	Morphological identification of small mammals was done using external criteria such as fur color, nipple formula, and standard measurements: body weight, head-and-body length, tail length, hindfoot length and ear length [21]. Molecular identification was facilitated by DNA cytochrome b sequencing, especially for the Mastomys spp. and other sibling species such as those within Mus (Nannomys) spp. We did not designate specimens from Crocidura to species level because, even with the benefit of cytochrome b sequencing, the taxonomy of this genus is not well established. For example, the species currently known as C. olivieri (for which a large number of specimens were morphologically identified in our collection) has very recently been described as polyphyletic [22].	Yes
Olayemi, 2018	Cross-sectional	Probabilistic	Trapping	Multicenter	Prospetively	Nigeria	Jan/2011-Oct/2012, Nov/2015	Unclear/ Not reported	Unclear/ Not reported	Unclear/ Not reported	Urban/rural	Community-based	Not applicable (if not in the hospital)	Not applicable (if not in the hospital)	Not applicable (for animals and healty participants)	Not applicable (for animals and healty participants)	Not applicable (for animals and healty participants)	Unclear/Not reported	Classical RT-PCR	Serum	RNA	Current infection	Other mammals	Insectivora, Crocidura spp., Erinaceus albiventris	Not applicable	Not applicable
Olayemi, 2018	Cross-sectional	Probabilistic	Trapping	Multicenter	Prospetively	Nigeria	Jan/2011-Oct/2012, Nov/2015	Unclear/ Not reported	Unclear/ Not reported	Unclear/ Not reported	Urban/rural	Community-based	Not applicable (if not in the hospital)	Not applicable (if not in the hospital)	Not applicable (for animals and healty participants)	Not applicable (for animals and healty participants)	Not applicable (for animals and healty participants)	Unclear/Not reported	Indirect immunofluorescence assay	Serum	IgG	At least one past contact	Other mammals	Insectivora, Crocidura spp., Erinaceus albiventris	Not applicable	Not applicable

Olayemi, 2018	Cross-sectional	Probabilistic	Trapping	Multicenter	Prospetively	Nigeria	Jan/2011-Oct/2012, Nov/2015	Unclear/ Not reported	Unclear/ Not reported	Unclear/ Not reported	Urban/rural	Community-based	Not applicable (if not in the hospital)	Not applicable (if not in the hospital)	Not applicable (for animals and healty participants)	Not applicable (for animals and healty participants)	Not applicable (for animals and healty participants)	Unclear/Not reported	Indirect immunofluorescence assay	Serum	IgG	At least one past contact	Rodents	Mastomys erythroleucus	Morphological identification of small mammals was done using external criteria such as fur color, nipple formula, and standard measurements: body weight, head-and-body length, tail length, hindfoot length and ear length [21]. Molecular identification was facilitated by DNA cytochrome b sequencing, especially for the Mastomys spp. and other sibling species such as those within Mus (Nannomys) spp. We did not designate specimens from Crocidura to species level because, even with the benefit of cytochrome b sequencing, the taxonomy of this genus is not well established. For example, the species currently known as C. olivieri (for which a large number of specimens were morphologically identified in our collection) has very recently been described as polyphyletic [22].	Yes
Olayemi, 2018	Cross-sectional	Probabilistic	Trapping	Multicenter	Prospetively	Nigeria	Jan/2011-Oct/2012, Nov/2015	Unclear/ Not reported	Unclear/ Not reported	Unclear/ Not reported	Urban/rural	Community-based	Not applicable (if not in the hospital)	Not applicable (if not in the hospital)	Not applicable (for animals and healty participants)	Not applicable (for animals and healty participants)	Not applicable (for animals and healty participants)	Unclear/Not reported	Indirect immunofluorescence assay	Serum	IgG	At least one past contact	Rodents	Mastomys natalensis	Morphological identification of small mammals was done using external criteria such as fur color, nipple formula, and standard measurements: body weight, head-and-body length, tail length, hindfoot length and ear length [21]. Molecular identification was facilitated by DNA cytochrome b sequencing, especially for the Mastomys spp. and other sibling species such as those within Mus (Nannomys) spp. We did not designate specimens from Crocidura to species level because, even with the benefit of cytochrome b sequencing, the taxonomy of this genus is not well established. For example, the species currently known as C. olivieri (for which a large number of specimens were morphologically identified in our collection) has very recently been described as polyphyletic [22].	Yes
Olayemi, 2018	Cross-sectional	Probabilistic	Trapping	Multicenter	Prospetively	Nigeria	Jan/2011-Oct/2012, Nov/2015	Unclear/ Not reported	Unclear/ Not reported	Unclear/ Not reported	Urban/rural	Community-based	Not applicable (if not in the hospital)	Not applicable (if not in the hospital)	Not applicable (for animals and healty participants)	Not applicable (for animals and healty participants)	Not applicable (for animals and healty participants)	Unclear/Not reported	Classical RT-PCR	Serum	RNA	Current infection	Rodents	Mus baoulei	Morphological identification of small mammals was done using external criteria such as fur color, nipple formula, and standard measurements: body weight, head-and-body length, tail length, hindfoot length and ear length [21]. Molecular identification was facilitated by DNA cytochrome b sequencing, especially for the Mastomys spp. and other sibling species such as those within Mus (Nannomys) spp. We did not designate specimens from Crocidura to species level because, even with the benefit of cytochrome b sequencing, the taxonomy of this genus is not well established. For example, the species currently known as C. olivieri (for which a large number of specimens were morphologically identified in our collection) has very recently been described as polyphyletic [22].	Yes
Olayemi, 2018	Cross-sectional	Probabilistic	Trapping	Multicenter	Prospetively	Nigeria	Jan/2011-Oct/2012, Nov/2015	Unclear/ Not reported	Unclear/ Not reported	Unclear/ Not reported	Urban/rural	Community-based	Not applicable (if not in the hospital)	Not applicable (if not in the hospital)	Not applicable (for animals and healty participants)	Not applicable (for animals and healty participants)	Not applicable (for animals and healty participants)	Unclear/Not reported	Indirect immunofluorescence assay	Serum	IgG	At least one past contact	Rodents	Mus baoulei	Morphological identification of small mammals was done using external criteria such as fur color, nipple formula, and standard measurements: body weight, head-and-body length, tail length, hindfoot length and ear length [21]. Molecular identification was facilitated by DNA cytochrome b sequencing, especially for the Mastomys spp. and other sibling species such as those within Mus (Nannomys) spp. We did not designate specimens from Crocidura to species level because, even with the benefit of cytochrome b sequencing, the taxonomy of this genus is not well established. For example, the species currently known as C. olivieri (for which a large number of specimens were morphologically identified in our collection) has very recently been described as polyphyletic [22].	Yes
Olayemi, 2018	Cross-sectional	Probabilistic	Trapping	Multicenter	Prospetively	Nigeria	Jan/2011-Oct/2012, Nov/2015	Unclear/ Not reported	Unclear/ Not reported	Unclear/ Not reported	Urban/rural	Community-based	Not applicable (if not in the hospital)	Not applicable (if not in the hospital)	Not applicable (for animals and healty participants)	Not applicable (for animals and healty participants)	Not applicable (for animals and healty participants)	Unclear/Not reported	Classical RT-PCR	Serum	RNA	Current infection	Rodents	Mus minutoides	Morphological identification of small mammals was done using external criteria such as fur color, nipple formula, and standard measurements: body weight, head-and-body length, tail length, hindfoot length and ear length [21]. Molecular identification was facilitated by DNA cytochrome b sequencing, especially for the Mastomys spp. and other sibling species such as those within Mus (Nannomys) spp. We did not designate specimens from Crocidura to species level because, even with the benefit of cytochrome b sequencing, the taxonomy of this genus is not well established. For example, the species currently known as C. olivieri (for which a large number of specimens were morphologically identified in our collection) has very recently been described as polyphyletic [22].	Yes
Olayemi, 2018	Cross-sectional	Probabilistic	Trapping	Multicenter	Prospetively	Nigeria	Jan/2011-Oct/2012, Nov/2015	Unclear/ Not reported	Unclear/ Not reported	Unclear/ Not reported	Urban/rural	Community-based	Not applicable (if not in the hospital)	Not applicable (if not in the hospital)	Not applicable (for animals and healty participants)	Not applicable (for animals and healty participants)	Not applicable (for animals and healty participants)	Unclear/Not reported	Indirect immunofluorescence assay	Serum	IgG	At least one past contact	Rodents	Mus minutoides	Morphological identification of small mammals was done using external criteria such as fur color, nipple formula, and standard measurements: body weight, head-and-body length, tail length, hindfoot length and ear length [21]. Molecular identification was facilitated by DNA cytochrome b sequencing, especially for the Mastomys spp. and other sibling species such as those within Mus (Nannomys) spp. We did not designate specimens from Crocidura to species level because, even with the benefit of cytochrome b sequencing, the taxonomy of this genus is not well established. For example, the species currently known as C. olivieri (for which a large number of specimens were morphologically identified in our collection) has very recently been described as polyphyletic [22].	Yes
Olayemi, 2018	Cross-sectional	Probabilistic	Trapping	Multicenter	Prospetively	Nigeria	Jan/2011-Oct/2012, Nov/2015	Unclear/ Not reported	Unclear/ Not reported	Unclear/ Not reported	Urban/rural	Community-based	Not applicable (if not in the hospital)	Not applicable (if not in the hospital)	Not applicable (for animals and healty participants)	Not applicable (for animals and healty participants)	Not applicable (for animals and healty participants)	Unclear/Not reported	Classical RT-PCR	Serum	RNA	Current infection	Rodents	Mus setulosus	Morphological identification of small mammals was done using external criteria such as fur color, nipple formula, and standard measurements: body weight, head-and-body length, tail length, hindfoot length and ear length [21]. Molecular identification was facilitated by DNA cytochrome b sequencing, especially for the Mastomys spp. and other sibling species such as those within Mus (Nannomys) spp. We did not designate specimens from Crocidura to species level because, even with the benefit of cytochrome b sequencing, the taxonomy of this genus is not well established. For example, the species currently known as C. olivieri (for which a large number of specimens were morphologically identified in our collection) has very recently been described as polyphyletic [22].	Yes
Olayemi, 2018	Cross-sectional	Probabilistic	Trapping	Multicenter	Prospetively	Nigeria	Jan/2011-Oct/2012, Nov/2015	Unclear/ Not reported	Unclear/ Not reported	Unclear/ Not reported	Urban/rural	Community-based	Not applicable (if not in the hospital)	Not applicable (if not in the hospital)	Not applicable (for animals and healty participants)	Not applicable (for animals and healty participants)	Not applicable (for animals and healty participants)	Unclear/Not reported	Indirect immunofluorescence assay	Serum	IgG	At least one past contact	Rodents	Mus setulosus	Morphological identification of small mammals was done using external criteria such as fur color, nipple formula, and standard measurements: body weight, head-and-body length, tail length, hindfoot length and ear length [21]. Molecular identification was facilitated by DNA cytochrome b sequencing, especially for the Mastomys spp. and other sibling species such as those within Mus (Nannomys) spp. We did not designate specimens from Crocidura to species level because, even with the benefit of cytochrome b sequencing, the taxonomy of this genus is not well established. For example, the species currently known as C. olivieri (for which a large number of specimens were morphologically identified in our collection) has very recently been described as polyphyletic [22].	Yes
Olayemi, 2018	Cross-sectional	Probabilistic	Trapping	Multicenter	Prospetively	Nigeria	Jan/2011-Oct/2012, Nov/2015	Unclear/ Not reported	Unclear/ Not reported	Unclear/ Not reported	Urban/rural	Community-based	Not applicable (if not in the hospital)	Not applicable (if not in the hospital)	Not applicable (for animals and healty participants)	Not applicable (for animals and healty participants)	Not applicable (for animals and healty participants)	Unclear/Not reported	Indirect immunofluorescence assay	Serum	IgG	At least one past contact	Rodents	Praomys daltoni	Morphological identification of small mammals was done using external criteria such as fur color, nipple formula, and standard measurements: body weight, head-and-body length, tail length, hindfoot length and ear length [21]. Molecular identification was facilitated by DNA cytochrome b sequencing, especially for the Mastomys spp. and other sibling species such as those within Mus (Nannomys) spp. We did not designate specimens from Crocidura to species level because, even with the benefit of cytochrome b sequencing, the taxonomy of this genus is not well established. For example, the species currently known as C. olivieri (for which a large number of specimens were morphologically identified in our collection) has very recently been described as polyphyletic [22].	Yes
Olayemi, 2018	Cross-sectional	Probabilistic	Trapping	Multicenter	Prospetively	Nigeria	Jan/2011-Oct/2012, Nov/2015	Unclear/ Not reported	Unclear/ Not reported	Unclear/ Not reported	Urban/rural	Community-based	Not applicable (if not in the hospital)	Not applicable (if not in the hospital)	Not applicable (for animals and healty participants)	Not applicable (for animals and healty participants)	Not applicable (for animals and healty participants)	Unclear/Not reported	Classical RT-PCR	Serum	RNA	Current infection	Rodents	Praomys jacksoni	Morphological identification of small mammals was done using external criteria such as fur color, nipple formula, and standard measurements: body weight, head-and-body length, tail length, hindfoot length and ear length [21]. Molecular identification was facilitated by DNA cytochrome b sequencing, especially for the Mastomys spp. and other sibling species such as those within Mus (Nannomys) spp. We did not designate specimens from Crocidura to species level because, even with the benefit of cytochrome b sequencing, the taxonomy of this genus is not well established. For example, the species currently known as C. olivieri (for which a large number of specimens were morphologically identified in our collection) has very recently been described as polyphyletic [22].	Yes

Olayemi, 2018	Cross-sectional	Probabilistic	Trapping	Multicenter	Prospetively	Nigeria	Jan/2011-Oct/2012, Nov/2015	Unclear/ Not reported	Unclear/ Not reported	Unclear/ Not reported	Urban/rural	Community-based	Not applicable (if not in the hospital)	Not applicable (if not in the hospital)	Not applicable (for animals and healty participants)	Not applicable (for animals and healty participants)	Not applicable (for animals and healty participants)	Unclear/Not reported	Indirect immunofluorescence assay	Serum	IgG	At least one past contact	Rodents	Praomys jacksoni	Morphological identification of small mammals was done using external criteria such as fur color, nipple formula, and standard measurements: body weight, head-and-body length, tail length, hindfoot length and ear length [21]. Molecular identification was facilitated by DNA cytochrome b sequencing, especially for the Mastomys spp. and other sibling species such as those within Mus (Nannomys) spp. We did not designate specimens from Crocidura to species level because, even with the benefit of cytochrome b sequencing, the taxonomy of this genus is not well established. For example, the species currently known as C. olivieri (for which a large number of specimens were morphologically identified in our collection) has very recently been described as polyphyletic [22].	Yes
Olayemi, 2018	Cross-sectional	Probabilistic	Trapping	Multicenter	Prospetively	Nigeria	Jan/2011-Oct/2012, Nov/2015	Unclear/ Not reported	Unclear/ Not reported	Unclear/ Not reported	Urban/rural	Community-based	Not applicable (if not in the hospital)	Not applicable (if not in the hospital)	Not applicable (for animals and healty participants)	Not applicable (for animals and healty participants)	Not applicable (for animals and healty participants)	Unclear/Not reported	Indirect immunofluorescence assay	Serum	IgG	At least one past contact	Rodents	Rattus rattus	Morphological identification of small mammals was done using external criteria such as fur color, nipple formula, and standard measurements: body weight, head-and-body length, tail length, hindfoot length and ear length [21]. Molecular identification was facilitated by DNA cytochrome b sequencing, especially for the Mastomys spp. and other sibling species such as those within Mus (Nannomys) spp. We did not designate specimens from Crocidura to species level because, even with the benefit of cytochrome b sequencing, the taxonomy of this genus is not well established. For example, the species currently known as C. olivieri (for which a large number of specimens were morphologically identified in our collection) has very recently been described as polyphyletic [22].	Yes
Oloniniyi, 2018	Cross-sectional	Non probabilistic	Consecutive sampling	Multicenter	Prospetively	Nigeria	2012-2016	Unclear/ Not reported	Unclear/ Not reported	Unclear/ Not reported	Urban/rural	Hospital-based	Unclear/Not reported	Hospitalized	A diagnosis of acute LF was made based on clinical signs and symptoms of fever, headache, and/or hemorrhage, contact history with a suspected or confirmed LF patient, as well as the discretion of the managing physician.	Unclear/Not reported	Unclear/Not reported	Unclear/Not reported	Classical RT-PCR	Serum	RNA	Current infection	Humans	LASV suspected cases	Not applicable	Not applicable
Omilabu, 2005	Hospital outbreak	Non probabilistic	Convenience sampling	Monocenter	Prospetively	Nigeria	Sep/2003-Jan/2004	Unclear/ Not reported	Unclear/ Not reported	Unclear/ Not reported	Urban	Hospital-based	Tertiary hospital	Ambulatory	Not applicable (for animals and healty participants)	Not applicable (for animals and healty participants)	Not applicable (for animals and healty participants)	Unclear/Not reported	Classical RT-PCR	Serum	RNA	Current infection	Humans	Apparently healthy individuals	Not applicable	Not applicable
Omilabu, 2005	Hospital outbreak	Non probabilistic	Convenience sampling	Monocenter	Prospetively	Nigeria	Sep/2003-Jan/2004	Unclear/ Not reported	Unclear/ Not reported	Unclear/ Not reported	Urban	Hospital-based	Tertiary hospital	Ambulatory	Not applicable (for animals and healty participants)	Not applicable (for animals and healty participants)	Not applicable (for animals and healty participants)	Unclear/Not reported	Indirect immunofluorescence assay	Serum	IgM	Recent infection	Humans	Apparently healthy individuals	Not applicable	Not applicable
Omilabu, 2005	Hospital outbreak	Non probabilistic	Convenience sampling	Monocenter	Prospetively	Nigeria	Sep/2003-Jan/2004	Unclear/ Not reported	Unclear/ Not reported	Unclear/ Not reported	Urban	Hospital-based	Tertiary hospital	Ambulatory	Not applicable (for animals and healty participants)	Not applicable (for animals and healty participants)	Not applicable (for animals and healty participants)	Unclear/Not reported	Indirect immunofluorescence assay	Serum	IgG	At least one past contact	Humans	Apparently healthy individuals	Not applicable	Not applicable
Omilabu, 2005	Hospital outbreak	Non probabilistic	Convenience sampling	Monocenter	Prospetively	Nigeria	Sep/2003-Jan/2004	Unclear/ Not reported	Unclear/ Not reported	Unclear/ Not reported	Urban	Hospital-based	Tertiary hospital	Ambulatory	Not applicable (for animals and healty participants)	Not applicable (for animals and healty participants)	Not applicable (for animals and healty participants)	Unclear/Not reported	Classical RT-PCR	Serum	RNA	Current infection	Humans	Healthcare workers	Not applicable	Not applicable
Omilabu, 2005	Hospital outbreak	Non probabilistic	Convenience sampling	Monocenter	Prospetively	Nigeria	Sep/2003-Jan/2004	Unclear/ Not reported	Unclear/ Not reported	Unclear/ Not reported	Urban	Hospital-based	Tertiary hospital	Ambulatory	Not applicable (for animals and healty participants)	Not applicable (for animals and healty participants)	Not applicable (for animals and healty participants)	Unclear/Not reported	Indirect immunofluorescence assay	Serum	IgM	Recent infection	Humans	Healthcare workers	Not applicable	Not applicable
Omilabu, 2005	Hospital outbreak	Non probabilistic	Convenience sampling	Monocenter	Prospetively	Nigeria	Sep/2003-Jan/2004	Unclear/ Not reported	Unclear/ Not reported	Unclear/ Not reported	Urban	Hospital-based	Tertiary hospital	Ambulatory	Not applicable (for animals and healty participants)	Not applicable (for animals and healty participants)	Not applicable (for animals and healty participants)	Unclear/Not reported	Indirect immunofluorescence assay	Serum	IgG	At least one past contact	Humans	Healthcare workers	Not applicable	Not applicable
Omilabu, 2005	Hospital outbreak	Non probabilistic	Convenience sampling	Monocenter	Prospetively	Nigeria	Sep/2003-Jan/2004	Unclear/ Not reported	Unclear/ Not reported	Unclear/ Not reported	Urban	Hospital-based	Tertiary hospital	Hospitalized	Unclear/Not reported	Unclear/Not reported	Unclear/Not reported	Unclear/Not reported	Classical RT-PCR	Serum	RNA	Current infection	Humans	Febrile patients	Not applicable	Not applicable
Omilabu, 2005	Hospital outbreak	Non probabilistic	Convenience sampling	Monocenter	Prospetively	Nigeria	Sep/2003-Jan/2004	Unclear/ Not reported	Unclear/ Not reported	Unclear/ Not reported	Urban	Hospital-based	Tertiary hospital	Hospitalized	Unclear/Not reported	Unclear/Not reported	Unclear/Not reported	Unclear/Not reported	Indirect immunofluorescence assay	Serum	IgM	Recent infection	Humans	Febrile patients	Not applicable	Not applicable
Omilabu, 2005	Hospital outbreak	Non probabilistic	Convenience sampling	Monocenter	Prospetively	Nigeria	Sep/2003-Jan/2004	Unclear/ Not reported	Unclear/ Not reported	Unclear/ Not reported	Urban	Hospital-based	Tertiary hospital	Hospitalized	Unclear/Not reported	Unclear/Not reported	Unclear/Not reported	Unclear/Not reported	Indirect immunofluorescence assay	Serum	IgG	At least one past contact	Humans	Febrile patients	Not applicable	Not applicable
Paix, 1988	Cross-sectional	Non probabilistic	Consecutive sampling	Unclear/Not reported	Unclear/Not reported	Cameroon	Unclear/ Not reported	Unclear/ Not reported	Unclear/ Not reported	Unclear/ Not reported	Urban	Community-based	Not applicable (if not in the hospital)	Not applicable (if not in the hospital)	Not applicable (for animals and healty participants)	Not applicable (for animals and healty participants)	Not applicable (for animals and healty participants)	Unclear/Not reported	Unclear/Not reported	Unclear/Not reported	Antibodies	At least one past contact	Humans	Apparently healthy individuals	Not applicable	Not applicable
Panning, 2010	Cross-sectional	Non probabilistic	Consecutive sampling	Monocenter	Prospetively	Liberia	Unclear/ Not reported	Unclear/ Not reported	Unclear/ Not reported	Unclear/ Not reported	Unclear/Not reported	Hospital-based	Unclear/Not reported	Unclear/Not reported	Patients were suspected of having Lassa fever on clinical grounds by physicians of the United Nations peacekeeping troops and other international relief organizations.	Unclear/Not reported	Unclear/Not reported	Unclear/Not reported	Classical RT-PCR	Serum	RNA	Current infection	Humans	LASV suspected cases	Not applicable	Not applicable
Panning, 2010	Cross-sectional	Non probabilistic	Consecutive sampling	Monocenter	Prospetively	Liberia	Unclear/ Not reported	Unclear/ Not reported	Unclear/ Not reported	Unclear/ Not reported	Unclear/Not reported	Hospital-based	Unclear/Not reported	Unclear/Not reported	Patients were suspected of having Lassa fever on clinical grounds by physicians of the United Nations peacekeeping troops and other international relief organizations.	Unclear/Not reported	Unclear/Not reported	Unclear/Not reported	Culture	Serum	Virus	Current infection	Humans	LASV suspected cases	Not applicable	Not applicable
Panning, 2010	Cross-sectional	Non probabilistic	Consecutive sampling	Monocenter	Prospetively	Liberia	Unclear/ Not reported	Unclear/ Not reported	Unclear/ Not reported	Unclear/ Not reported	Unclear/Not reported	Hospital-based	Unclear/Not reported	Unclear/Not reported	Patients were suspected of having Lassa fever on clinical grounds by physicians of the United Nations peacekeeping troops and other international relief organizations.	Unclear/Not reported	Unclear/Not reported	Unclear/Not reported	Indirect immunofluorescence assay	Serum	IgM and IgG	Recent infection	Humans	LASV suspected cases	Not applicable	Not applicable
Price, 1988	Cross-sectional	Non probabilistic	Consecutive sampling	Monocenter	Prospetively	Sierra Leone	1981-1985	Unclear/ Not reported	Unclear/ Not reported	0	Urban	Hospital-based	antenatal clinic or through the medical outpatient clinic	Ambulatory	Not applicable (for animals and healty participants)	Not applicable (for animals and healty participants)	Not applicable (for animals and healty participants)	Lassa fever was confirmed by finding on immunofluorescence a fourfold rise in the titre of IgG to Lassa fever virus (minimum final titre of 16) or an IgG titre of -256 with IgM antibody to Lassa fever, or by isolating the virus.	Immunoflorescent assay, Culture	Serum	IgG, IgM, Virus	Current infection	Humans	Pregnant women	Not applicable	Not applicable
Price, 1988	Cross-sectional	Non probabilistic	Consecutive sampling	Monocenter	Prospetively	Sierra Leone	1981-1985	Unclear/ Not reported	Unclear/ Not reported	0	Urban	Hospital-based	antenatal clinic or through the medical outpatient clinic	Hospitalized	Unclear/Not reported	Unclear/Not reported	Unclear/Not reported	Lassa fever was confirmed by finding on immunofluorescence a fourfold rise in the titre of IgG to Lassa fever virus (minimum final titre of 16) or an IgG titre of -256 with IgM antibody to Lassa fever, or by isolating the virus.	Immunoflorescent assay, Culture	Serum	IgG, IgM, Virus	Current infection	Humans	LASV suspected cases	Not applicable	Not applicable
Rodhain, 1989	Cross-sectional	Non probabilistic	Consecutive sampling	Multicenter	Prospetively	Uganda	1984	Unclear/ Not reported	20-40	49.2	Rural	Hospital/community based	Unclear/Not reported	Ambulatory	Not applicable (for animals and healty participants)	Not applicable (for animals and healty participants)	Not applicable (for animals and healty participants)	Unclear/Not reported	Indirect immunofluorescence assay	Serum	Antibodies	At least one past contact	Humans	Apparently healthy individuals	Not applicable	Not applicable
Safronetz, 2010	Cross-sectional	Non probabilistic	Trapping	Multicenter	Prospetively	Mali	Jun/2009	Unclear/ Not reported	Unclear/ Not reported	Unclear/ Not reported	Urban	Community-based	Not applicable (if not in the hospital)	Not applicable (if not in the hospital)	Not applicable (for animals and healty participants)	Not applicable (for animals and healty participants)	Not applicable (for animals and healty participants)	Unclear/Not reported	Real-time PCR	Serum	RNA	Current infection	Rodents	Unspecified rodents	Rats were the predominant rodent captured (82/103, 79.6%), with all but 4 of these animals identified as M. natalensis rat by cytochrome B sequence analysis of DNA isolated from ear punch specimens (11; GenBank accession nos. HM130517-HM130519).	Yes
Safronetz, 2010	Cross-sectional	Non probabilistic	Trapping	Multicenter	Prospetively	Mali	Jun/2009	Unclear/ Not reported	Unclear/ Not reported	Unclear/ Not reported	Urban	Community-based	Not applicable (if not in the hospital)	Not applicable (if not in the hospital)	Not applicable (for animals and healty participants)	Not applicable (for animals and healty participants)	Not applicable (for animals and healty participants)	Unclear/Not reported	Real-time PCR	liver	RNA	Current infection	Rodents	Unspecified rodents	Rats were the predominant rodent captured (82/103, 79.6%), with all but 4 of these animals identified as M. natalensis rat by cytochrome B sequence analysis of DNA isolated from ear punch specimens (11; GenBank accession nos. HM130517-HM130519).	Yes
Safronetz, 2010	Cross-sectional	Non probabilistic	Trapping	Multicenter	Prospetively	Mali	Jun/2009	Unclear/ Not reported	Unclear/ Not reported	Unclear/ Not reported	Urban	Community-based	Not applicable (if not in the hospital)	Not applicable (if not in the hospital)	Not applicable (for animals and healty participants)	Not applicable (for animals and healty participants)	Not applicable (for animals and healty participants)	Unclear/Not reported	Real-time PCR	lung	RNA	Current infection	Rodents	Unspecified rodents	Rats were the predominant rodent captured (82/103, 79.6%), with all but 4 of these animals identified as M. natalensis rat by cytochrome B sequence analysis of DNA isolated from ear punch specimens (11; GenBank accession nos. HM130517-HM130519).	Yes
Safronetz, 2010	Cross-sectional	Non probabilistic	Trapping	Multicenter	Prospetively	Mali	Jun/2009	Unclear/ Not reported	Unclear/ Not reported	Unclear/ Not reported	Urban	Community-based	Not applicable (if not in the hospital)	Not applicable (if not in the hospital)	Not applicable (for animals and healty participants)	Not applicable (for animals and healty participants)	Not applicable (for animals and healty participants)	Unclear/Not reported	Real-time PCR	Serum	RNA	Current infection	Rodents	Mastomys species	Rats were the predominant rodent captured (82/103, 79.6%), with all but 4 of these animals identified as M. natalensis rat by cytochrome B sequence analysis of DNA isolated from ear punch specimens (11; GenBank accession nos. HM130517-HM130519).	Yes
Safronetz, 2010	Cross-sectional	Non probabilistic	Trapping	Multicenter	Prospetively	Mali	Jun/2009	Unclear/ Not reported	Unclear/ Not reported	Unclear/ Not reported	Urban	Community-based	Not applicable (if not in the hospital)	Not applicable (if not in the hospital)	Not applicable (for animals and healty participants)	Not applicable (for animals and healty participants)	Not applicable (for animals and healty participants)	Unclear/Not reported	Real-time PCR	liver	RNA	Current infection	Rodents	Mastomys species	Rats were the predominant rodent captured (82/103, 79.6%), with all but 4 of these animals identified as M. natalensis rat by cytochrome B sequence analysis of DNA isolated from ear punch specimens (11; GenBank accession nos. HM130517-HM130519).	Yes
Safronetz, 2010	Cross-sectional	Non probabilistic	Trapping	Multicenter	Prospetively	Mali	Jun/2009	Unclear/ Not reported	Unclear/ Not reported	Unclear/ Not reported	Urban	Community-based	Not applicable (if not in the hospital)	Not applicable (if not in the hospital)	Not applicable (for animals and healty participants)	Not applicable (for animals and healty participants)	Not applicable (for animals and healty participants)	Unclear/Not reported	Real-time PCR	lung	RNA	Current infection	Rodents	Mastomys species	Rats were the predominant rodent captured (82/103, 79.6%), with all but 4 of these animals identified as M. natalensis rat by cytochrome B sequence analysis of DNA isolated from ear punch specimens (11; GenBank accession nos. HM130517-HM130519).	Yes
Safronetz, 2013	Cross-sectional	Non probabilistic	Trapping	Multicenter	Prospetively	Mali	Dec/2007-Mar/2012	Unclear/ Not reported	Unclear/ Not reported	Unclear/ Not reported	Rural	Community-based	Not applicable (if not in the hospital)	Not applicable (if not in the hospital)	Not applicable (for animals and healty participants)	Not applicable (for animals and healty participants)	Not applicable (for animals and healty participants)	Unclear/Not reported	Classical RT-PCR	Organ tissue	RNA	Current infection	Rodents	Mastomys natalensis	The weight, sex and relative age (inferred from body weight and developmental stage) were recorded for each rodent and an ear punch collected and stored in 70% ethanol for cytochrome B sequencing.	Yes
Safronetz, 2013	Cross-sectional	Non probabilistic	Trapping	Multicenter	Prospetively	Mali	Dec/2007-Mar/2012	Unclear/ Not reported	Unclear/ Not reported	Unclear/ Not reported	Rural	Community-based	Not applicable (if not in the hospital)	Not applicable (if not in the hospital)	Not applicable (for animals and healty participants)	Not applicable (for animals and healty participants)	Not applicable (for animals and healty participants)	Unclear/Not reported	Real-time PCR	Organ tissue	RNA	Current infection	Rodents	Mastomys natalensis	The weight, sex and relative age (inferred from body weight and developmental stage) were recorded for each rodent and an ear punch collected and stored in 70% ethanol for cytochrome B sequencing.	Yes
Safronetz, 2013	Cross-sectional	Non probabilistic	Trapping	Multicenter	Prospetively	Mali	Dec/2007-Mar/2012	Unclear/ Not reported	Unclear/ Not reported	Unclear/ Not reported	Rural	Community-based	Not applicable (if not in the hospital)	Not applicable (if not in the hospital)	Not applicable (for animals and healty participants)	Not applicable (for animals and healty participants)	Not applicable (for animals and healty participants)	Unclear/Not reported	Indirect ELISA	Serum	IgG	At least one past contact	Rodents	Mastomys natalensis	The weight, sex and relative age (inferred from body weight and developmental stage) were recorded for each rodent and an ear punch collected and stored in 70% ethanol for cytochrome B sequencing.	Yes
Safronetz, 2013	Cross-sectional	Non probabilistic	Trapping	Multicenter	Prospetively	Mali	Dec/2007-Mar/2012	Unclear/ Not reported	Unclear/ Not reported	Unclear/ Not reported	Rural	Community-based	Not applicable (if not in the hospital)	Not applicable (if not in the hospital)	Not applicable (for animals and healty participants)	Not applicable (for animals and healty participants)	Not applicable (for animals and healty participants)	Unclear/Not reported	Indirect ELISA	Serum	IgG	At least one past contact	Rodents	Unspecified rodents	The weight, sex and relative age (inferred from body weight and developmental stage) were recorded for each rodent and an ear punch collected and stored in 70% ethanol for cytochrome B sequencing.	Yes
Salu, 2019	Hospital outbreak	Non probabilistic	Convenience sampling	Multicenter	Prospetively	Benin	Nov/2014-Jan/2016	Unclear/ Not reported	Unclear/ Not reported	Unclear/ Not reported	Urban/rural	Community-based	Not applicable (if not in the hospital)	Not applicable (if not in the hospital)	Unclear/Not reported	Unclear/Not reported	Unclear/Not reported	Unclear/Not reported	Classical RT-PCR	Serum	RNA	Current infection	Humans	Patient with hemorrhagic fever	Not applicable	Not applicable
Saluzzo, 1988	Cross-sectional	Non probabilistic	Trapping	Multicenter	Prospetively	Senegal	Unclear/ Not reported	Unclear/ Not reported	Unclear/ Not reported	Unclear/ Not reported	Rural	Community-based	Not applicable (if not in the hospital)	Not applicable (if not in the hospital)	Not applicable (for animals and healty participants)	Not applicable (for animals and healty participants)	Not applicable (for animals and healty participants)	Unclear/Not reported	Indirect immunofluorescence assay	Serum	Antibodies	At least one past contact	Rodents	Unspecified rodents	Unclear/ Not reported	Unclear/ Not reported
Saluzzo, 1988	Cross-sectional	Non probabilistic	Trapping	Multicenter	Prospetively	Senegal	Unclear/ Not reported	Unclear/ Not reported	Unclear/ Not reported	Unclear/ Not reported	Rural	Community-based	Not applicable (if not in the hospital)	Not applicable (if not in the hospital)	Not applicable (for animals and healty participants)	Not applicable (for animals and healty participants)	Not applicable (for animals and healty participants)	Unclear/Not reported	Indirect immunofluorescence assay	Serum	Antibodies	At least one past contact	Rodents	Unspecified rodents	Unclear/ Not reported	Unclear/ Not reported
Saluzzo, 1988	Cross-sectional	Non probabilistic	Trapping	Multicenter	Prospetively	Senegal	Unclear/ Not reported	Unclear/ Not reported	Unclear/ Not reported	Unclear/ Not reported	Rural	Community-based	Not applicable (if not in the hospital)	Not applicable (if not in the hospital)	Not applicable (for animals and healty participants)	Not applicable (for animals and healty participants)	Not applicable (for animals and healty participants)	Unclear/Not reported	Indirect immunofluorescence assay	Serum	Antibodies	At least one past contact	Rodents	Unspecified rodents	Unclear/ Not reported	Unclear/ Not reported
Saluzzo, 1988	Cross-sectional	Non probabilistic	Trapping	Multicenter	Prospetively	Senegal	Unclear/ Not reported	Unclear/ Not reported	Unclear/ Not reported	Unclear/ Not reported	Rural	Community-based	Not applicable (if not in the hospital)	Not applicable (if not in the hospital)	Not applicable (for animals and healty participants)	Not applicable (for animals and healty participants)	Not applicable (for animals and healty participants)	Unclear/Not reported	Indirect immunofluorescence assay	Serum	Antibodies	At least one past contact	Rodents	Unspecified rodents	Unclear/ Not reported	Unclear/ Not reported
Saluzzo, 1988	Cross-sectional	Non probabilistic	Trapping	Multicenter	Prospetively	Senegal	Unclear/ Not reported	Unclear/ Not reported	Unclear/ Not reported	Unclear/ Not reported	Rural	Community-based	Not applicable (if not in the hospital)	Not applicable (if not in the hospital)	Not applicable (for animals and healty participants)	Not applicable (for animals and healty participants)	Not applicable (for animals and healty participants)	Unclear/Not reported	Indirect immunofluorescence assay	Serum	Antibodies	At least one past contact	Rodents	Unspecified rodents	Unclear/ Not reported	Unclear/ Not reported
Schoepp, 2014	Cross-sectional	Non probabilistic	Consecutive sampling	Monocenter	Prospetively	Sierra Leone	Oct/2006-Oct/2008	Unclear/ Not reported	Unclear/ Not reported	Unclear/ Not reported	Urban	Hospital-based	Health Centers	Hospitalized	Unclear/Not reported	Samples were from patients whose illness met a surveillance case definition for Lassa fever (Table 1) and were processed as described elsewhere (5). To be tested for suspected Lassa fever, a patient had to have a fever >38□C and not respond to appropriate antimalarial and antimicrobial drug treatment within 72 h.	Unclear/Not reported	Unclear/Not reported	Indirect ELISA	Serum	IgM	Recent infection	Humans	Febrile patients	Not applicable	Not applicable
Schoepp, 2014	Cross-sectional	Non probabilistic	Consecutive sampling	Monocenter	Prospetively	Sierra Leone	Oct/2006-Oct/2008	Unclear/ Not reported	Unclear/ Not reported	Unclear/ Not reported	Urban	Hospital-based	Health Centers	Hospitalized	Unclear/Not reported	Samples were from patients whose illness met a surveillance case definition for Lassa fever (Table 1) and were processed as described elsewhere (5). To be tested for suspected Lassa fever, a patient had to have a fever >38□C and not respond to appropriate antimalarial and antimicrobial drug treatment within 72 h.	Unclear/Not reported	Unclear/Not reported	Indirect ELISA	Serum	IgG	At least one past contact	Humans	Febrile patients	Not applicable	Not applicable
Shaffer, 2019	Cross-sectional	Non probabilistic	Consecutive sampling	Monocenter	Retrospectively	Sierra Leone	Jan/2008-Dec/2016	Unclear/ Not reported	Unclear/ Not reported	Unclear/ Not reported	Urban/rural	Hospital-based	Primary hospital	Hospitalized	Unclear/Not reported	Known exposure to a suspected case of Lassa fever or temperature over 38°C for less than three weeks and absence of signs of local inflammation and two major signs (bleeding, Neck or facial swelling, conjunctivitis or sub-conjunctival hemorrhage, spontaneous abortion) or one major sign and two minor signs (Headache, sore throat, vomiting, diffuse abdominal pain or tenderness, retrosternal or chest pain, cough, diarrhea, genralized myalgia or arthralgia, profuse weakness	Unclear/Not reported	Ag+/IgM+= Acute Lassa exposure. Ag-/IgM+= Récent Lassa exposure	Direct ELISA	Serum	Antigen	Current infection	Humans	LASV suspected cases	Not applicable	Not applicable
Shehu, 2018	Hospital outbreak	Non probabilistic	Convenience sampling	Monocenter	Retrospectively	Nigeria	Jan/2016-Aug/2016	31	15-35	47	Urban/rural	Hospital-based	University Teaching Hospital	Hospitalized	Unclear/Not reported	The study population consisted of patients with suspected LF based on WHO case definition of suspected LF as follows: Presence of fever of >38◦C for <3 weeks, absence of clear focus of infection and lack of response to broad spectrum antibiotics or anti-malarials for 48 h. Additionally, presence of any two major signs (bleeding from orifice(s), facial/neck swelling, conjunctivitis, spontaneous abortion, petechial rashes, persistent hypotension, elevated liver enzymes, known contact with LF patients or one major criterion with any two minor criteria (headache, vomiting, cough, sore throat, diffuse abdominal pain, retrosternal pain, profound weakness, myalgia/arthralgia and proteinuria) (12).	Unclear/Not reported	Unclear/Not reported	Classical RT-PCR	Serum	RNA	Current infection	Humans	LASV suspected cases	Not applicable	Not applicable
Smith, 1979	Cross-sectional	Non probabilistic	Convenience sampling	Multicenter	Retrospectively	Nigeria	Unclear/ Not reported	Unclear/ Not reported	Unclear/ Not reported	Unclear/ Not reported	Urban/rural	Hospital/community based	Unclear/Not reported	Ambulatory	Not applicable (for animals and healty participants)	Not applicable (for animals and healty participants)	Not applicable (for animals and healty participants)	Unclear/Not reported	Complement fixation test	Serum	Antibodies	At least one past contact	Humans	Healthcare workers	Not applicable	Not applicable
Smith, 1979	Community outbreak	Non probabilistic	Convenience sampling	Multicenter	Retrospectively	Nigeria	Unclear/ Not reported	Unclear/ Not reported	3-56	Unclear/ Not reported	Urban/rural	Hospital/community based	Unclear/Not reported	Unclear/Not reported	Unclear/Not reported	Unclear/Not reported	Unclear/Not reported	Unclear/Not reported	Complement fixation test	Serum	Antibodies	At least one past contact	Humans	Febrile patients	Not applicable	Not applicable
Sogoba, 2016	Cross-sectional	Non probabilistic	Convenience sampling	Multicenter	Prospetively	Mali	Feb/2015	21	0.6-83	47.5	Rural	Community-based	Not applicable (if not in the hospital)	Not applicable (if not in the hospital)	Not applicable (for animals and healty participants)	Not applicable (for animals and healty participants)	Not applicable (for animals and healty participants)	Serum samples collected in the current study were considered serologically positive if they yielded optical densities (OD450) >2 SDs above the baseline value (>0.8).	Indirect ELISA	Serum	IgM	Recent infection	Humans	Apparently healthy individuals	Not applicable	Not applicable

Sogoba, 2016	Cross-sectional	Non probabilistic	Convenience sampling	Multicenter	Prospetively	Mali	Feb/2015	21	0.6-83	47.5	Rural	Community-based	Not applicable (if not in the hospital)	Not applicable (if not in the hospital)	Not applicable (for animals and healty participants)	Not applicable (for animals and healty participants)	Not applicable (for animals and healty participants)	Serum samples collected in the current study were considered serologically positive if they yielded optical densities (OD450) >2 SDs above the baseline value (>0.8).	Indirect ELISA	Serum	IgG	At least one past contact	Humans	Apparently healthy individuals	Not applicable	Not applicable
Ter Meulen, 1994	Cross-sectional	Probabilistic	Cluster sampling	Multicenter	Prospetively	Guinea	Feb/1993-Apr/1993	Unclear/ Not reported	4-99	50	Rural	Community-based	Not applicable (if not in the hospital)	Ambulatory	Not applicable (for animals and healty participants)	Not applicable (for animals and healty participants)	Not applicable (for animals and healty participants)	Unclear/Not reported	Indirect immunofluorescence assay	Serum	Antibodies	At least one past contact	Humans	Apparently healthy individuals	Not applicable	Not applicable
Ter Meulen, 1998	Cross-sectional	Non probabilistic	Consecutive sampling	Multicenter	Retrospectively	Guinea, Liberia	1985, 1993, 1995-1996	Unclear/ Not reported	Unclear/ Not reported	Unclear/ Not reported	Unclear/Not reported	Hospital/community based	Unclear/Not reported	Hospitalized	FUO was defined as any febrile condition (.38.5°C) not responsive to antimalarial or antibiotic treatment for more than 3 days.	Unclear/Not reported	Unclear/Not reported	Unclear/Not reported	PCR and sequencing	Serum	RNA	Current infection	Humans	Febrile patients	Not applicable	Not applicable
Ter Meulen, 1998	Cross-sectional	Non probabilistic	Consecutive sampling	Multicenter	Retrospectively	Guinea, Liberia	1985, 1993, 1995-1996	Unclear/ Not reported	Unclear/ Not reported	Unclear/ Not reported	Unclear/Not reported	Hospital/community based	Unclear/Not reported	Hospitalized	FUO was defined as any febrile condition (.38.5°C) not responsive to antimalarial or antibiotic treatment for more than 3 days.	Unclear/Not reported	Unclear/Not reported	Unclear/Not reported	PCR and sequencing	Serum	RNA	Current infection	Humans	Patient with hemorrhagic fever	Not applicable	Not applicable
Ter Meulen, 1998	Cross-sectional	Non probabilistic	Consecutive sampling	Multicenter	Retrospectively	Guinea, Liberia	1985, 1993, 1995-1996	Unclear/ Not reported	Unclear/ Not reported	Unclear/ Not reported	Unclear/Not reported	Community-based	Unclear/Not reported	Ambulatory	Not applicable (for animals and healty participants)	Not applicable (for animals and healty participants)	Not applicable (for animals and healty participants)	Unclear/Not reported	Immunoblot Assay	Serum, urine, and, occasionally, cerebrospinal fluid	IgM and IgG	Recent infection	Humans	Apparently healthy individuals	Not applicable	Not applicable
Ter Meulen, 1998	Cross-sectional	Non probabilistic	Consecutive sampling	Multicenter	Retrospectively	Guinea, Liberia	1985, 1993, 1995-1996	Unclear/ Not reported	Unclear/ Not reported	Unclear/ Not reported	Unclear/Not reported	Community-based	Unclear/Not reported	Ambulatory	Not applicable (for animals and healty participants)	Not applicable (for animals and healty participants)	Not applicable (for animals and healty participants)	Unclear/Not reported	Indirect immunofluorescence assay	Serum, urine, and, occasionally, cerebrospinal fluid	IgM and IgG	Recent infection	Humans	Apparently healthy individuals	Not applicable	Not applicable
Tomori, 1988	Cross-sectional	Non probabilistic	Consecutive sampling	Multicenter	Prospetively	Nigeria	Unclear/ Not reported	Unclear/ Not reported	5-70	56.6	Unclear/Not reported	Community-based	Not applicable (if not in the hospital)	Not applicable (if not in the hospital)	Not applicable (for animals and healty participants)	Not applicable (for animals and healty participants)	Not applicable (for animals and healty participants)	Unclear/Not reported	Indirect immunofluorescence assay	Serum	Antibodies	At least one past contact	Humans	Apparently healthy individuals	Not applicable	Not applicable
Troup, 1970	Community outbreak	Non probabilistic	Convenience sampling	Monocenter	Prospetively	Nigeria	Jan/1970-Feb/1970	Unclear/ Not reported	0.4-46	34.6	Unclear/Not reported	Community-based	Not applicable (if not in the hospital)	Not applicable (if not in the hospital)	Not applicable (for animals and healty participants)	Not applicable (for animals and healty participants)	Not applicable (for animals and healty participants)	Unclear/Not reported	Complement fixation test	Serum	Antibodies	At least one past contact	Humans	Healthcare workers	Not applicable	Not applicable
Troup, 1970	Community outbreak	Non probabilistic	Convenience sampling	Monocenter	Prospetively	Nigeria	Jan/1970-Feb/1970	Unclear/ Not reported	0.4-46	34.6	Unclear/Not reported	Community-based	Not applicable (if not in the hospital)	Not applicable (if not in the hospital)	Unclear/Not reported	Unclear/Not reported	Unclear/Not reported	Unclear/Not reported	Complement fixation test	Serum	Antibodies	At least one past contact	Humans	LASV positive case contact	Not applicable	Not applicable
Van der Waals, 1986	Cross-sectional	Non probabilistic	Consecutive sampling	Multicenter	Prospetively	Liberia	Unclear/ Not reported	Unclear/ Not reported	Unclear/ Not reported	Unclear/ Not reported	Unclear/Not reported	Hospital/community based	Unclear/Not reported	Unclear/Not reported	Unclear/Not reported	Unclear/Not reported	Unclear/Not reported	Unclear/Not reported	Indirect immunofluorescence assay	Serum	Antibodies	At least one past contact	Humans	Apparently healthy individuals, Patient with any illness	Not applicable	Not applicable
Webb, 1986	Cross-sectional	Non probabilistic	Consecutive sampling	Multicenter	Prospetively	Sierra Leone	Aug/1977-Aug/1981	Unclear/ Not reported	Unclear/ Not reported	Unclear/ Not reported	Unclear/Not reported	Hospital-based	Unclear/Not reported	Hospitalized	A case was defined as a febrile child hospital in-patient; (i) from whom Lassa virus was isolated, (ii) who showed either a seroconversion (~4 to 216) or four-fold rise (S, with antibody, S2 four-fold increase over S,) in IgG antibody to Lassav irus by immunofluorescenat ntibody assay(IFA), or (iii) a child with an anti-Lassa virus IgG antibody titre al:256 with IgM spectic for Lassa virus of 3 1:8 in a single specimen.	Unclear/Not reported	Unclear/Not reported	Unclear/Not reported	Culture	Cerebrospinal fluid, Serum	Virus	Current infection	Humans	Febrile patients	Not applicable	Not applicable
Webb, 1986	Cross-sectional	Non probabilistic	Consecutive sampling	Multicenter	Prospetively	Sierra Leone	Aug/1977-Aug/1981	Unclear/ Not reported	Unclear/ Not reported	Unclear/ Not reported	Unclear/Not reported	Hospital-based	Unclear/Not reported	Hospitalized	A case was defined as a febrile child hospital in-patient; (i) from whom Lassa virus was isolated, (ii) who showed either a seroconversion (~4 to 216) or four-fold rise (S, with antibody, S2 four-fold increase over S,) in IgG antibody to Lassav irus by immunofluorescenat ntibody assay(IFA), or (iii) a child with an anti-Lassa virus IgG antibody titre al:256 with IgM spectic for Lassa virus of 3 1:8 in a single specimen.	Unclear/Not reported	Unclear/Not reported	Unclear/Not reported	Indirect immunofluorescence assay	Serum	IgM and IgG	Recent infection	Humans	Febrile patients	Not applicable	Not applicable
Wulff, 1975	Cross-sectional	Non probabilistic	Trapping	Multicenter	Prospetively	Nigeria	1972-1973	Unclear/ Not reported	Unclear/ Not reported	Unclear/ Not reported	Unclear/Not reported	Community-based	Not applicable (if not in the hospital)	Not applicable (if not in the hospital)	Not applicable (for animals and healty participants)	Not applicable (for animals and healty participants)	Not applicable (for animals and healty participants)	Unclear/Not reported	Culture	Serum, Urine, Organ tissue	Virus	Current infection	Rodents	Unspecified rodents	Unclear/ Not reported	Unclear/ Not reported
Wulff, 1975	Cross-sectional	Non probabilistic	Trapping	Multicenter	Prospetively	Nigeria	1972-1973	Unclear/ Not reported	Unclear/ Not reported	Unclear/ Not reported	Unclear/Not reported	Community-based	Not applicable (if not in the hospital)	Not applicable (if not in the hospital)	Not applicable (for animals and healty participants)	Not applicable (for animals and healty participants)	Not applicable (for animals and healty participants)	Unclear/Not reported	Culture	Serum, Urine, Organ tissue	Virus	Current infection	Rodents	Unspecified rodents	Unclear/ Not reported	Unclear/ Not reported
Wulff, 1975	Cross-sectional	Non probabilistic	Trapping	Multicenter	Prospetively	Nigeria	1972-1973	Unclear/ Not reported	Unclear/ Not reported	Unclear/ Not reported	Unclear/Not reported	Community-based	Not applicable (if not in the hospital)	Not applicable (if not in the hospital)	Not applicable (for animals and healty participants)	Not applicable (for animals and healty participants)	Not applicable (for animals and healty participants)	Unclear/Not reported	Culture	Serum, Urine, Organ tissue	Virus	Current infection	Rodents	Unspecified rodents	Unclear/ Not reported	Unclear/ Not reported
Wulff, 1975	Cross-sectional	Non probabilistic	Trapping	Multicenter	Prospetively	Nigeria	1972-1973	Unclear/ Not reported	Unclear/ Not reported	Unclear/ Not reported	Unclear/Not reported	Community-based	Not applicable (if not in the hospital)	Not applicable (if not in the hospital)	Not applicable (for animals and healty participants)	Not applicable (for animals and healty participants)	Not applicable (for animals and healty participants)	Unclear/Not reported	Culture	Serum, Urine, Organ tissue	Virus	Current infection	Rodents	Unspecified rodents	Unclear/ Not reported	Unclear/ Not reported
Yadouleton, 2019	Cross-sectional	Non probabilistic	Trapping	Multicenter	Prospetively	Benin	2016-2017	Unclear/ Not reported	Unclear/ Not reported	Unclear/ Not reported	Rural	Community-based	Not applicable (if not in the hospital)	Not applicable (if not in the hospital)	Not applicable (for animals and healty participants)	Not applicable (for animals and healty participants)	Not applicable (for animals and healty participants)	Unclear/Not reported	Classical RT-PCR	Serum, Organ tissue	RNA	Current infection	Rodents	Crocodura species	We collected blood and organs (including spleen and liver) and identified the animals morphologically, according to standard measurements: body weight; body, tail, hindfoot, and ear lengths. Because of possible sibling species among Mastomys spp. and Mus spp. rodents, we performed molecular identification through a PCR targeting cytochrome b.	Yes
Yadouleton, 2019	Cross-sectional	Non probabilistic	Trapping	Multicenter	Prospetively	Benin	2016-2017	Unclear/ Not reported	Unclear/ Not reported	Unclear/ Not reported	Rural	Community-based	Not applicable (if not in the hospital)	Not applicable (if not in the hospital)	Not applicable (for animals and healty participants)	Not applicable (for animals and healty participants)	Not applicable (for animals and healty participants)	Unclear/Not reported	Classical RT-PCR	Serum, Organ tissue	RNA	Current infection	Rodents	Lemniscomys striatus	We collected blood and organs (including spleen and liver) and identified the animals morphologically, according to standard measurements: body weight; body, tail, hindfoot, and ear lengths. Because of possible sibling species among Mastomys spp. and Mus spp. rodents, we performed molecular identification through a PCR targeting cytochrome b.	Yes
Yadouleton, 2019	Cross-sectional	Non probabilistic	Trapping	Multicenter	Prospetively	Benin	2016-2017	Unclear/ Not reported	Unclear/ Not reported	Unclear/ Not reported	Rural	Community-based	Not applicable (if not in the hospital)	Not applicable (if not in the hospital)	Not applicable (for animals and healty participants)	Not applicable (for animals and healty participants)	Not applicable (for animals and healty participants)	Unclear/Not reported	Classical RT-PCR	Serum, Organ tissue	RNA	Current infection	Rodents	Rattus rattus	We collected blood and organs (including spleen and liver) and identified the animals morphologically, according to standard measurements: body weight; body, tail, hindfoot, and ear lengths. Because of possible sibling species among Mastomys spp. and Mus spp. rodents, we performed molecular identification through a PCR targeting cytochrome b.	Yes
Yadouleton, 2019	Cross-sectional	Non probabilistic	Trapping	Multicenter	Prospetively	Benin	2016-2017	Unclear/ Not reported	Unclear/ Not reported	Unclear/ Not reported	Rural	Community-based	Not applicable (if not in the hospital)	Not applicable (if not in the hospital)	Not applicable (for animals and healty participants)	Not applicable (for animals and healty participants)	Not applicable (for animals and healty participants)	Unclear/Not reported	Classical RT-PCR	Serum, Organ tissue	RNA	Current infection	Rodents	Praomys daltoni	We collected blood and organs (including spleen and liver) and identified the animals morphologically, according to standard measurements: body weight; body, tail, hindfoot, and ear lengths. Because of possible sibling species among Mastomys spp. and Mus spp. rodents, we performed molecular identification through a PCR targeting cytochrome b.	Yes
Yadouleton, 2019	Cross-sectional	Non probabilistic	Trapping	Multicenter	Prospetively	Benin	2016-2017	Unclear/ Not reported	Unclear/ Not reported	Unclear/ Not reported	Rural	Community-based	Not applicable (if not in the hospital)	Not applicable (if not in the hospital)	Not applicable (for animals and healty participants)	Not applicable (for animals and healty participants)	Not applicable (for animals and healty participants)	Unclear/Not reported	Classical RT-PCR	Serum, Organ tissue	RNA	Current infection	Rodents	Mus baoulei	We collected blood and organs (including spleen and liver) and identified the animals morphologically, according to standard measurements: body weight; body, tail, hindfoot, and ear lengths. Because of possible sibling species among Mastomys spp. and Mus spp. rodents, we performed molecular identification through a PCR targeting cytochrome b.	Yes
Yalley-Ogunro, 1984	Cross-sectional	Non probabilistic	Consecutive sampling	Multicenter	Prospetively	Liberia	1980-1982	Unclear/ Not reported	Unclear/ Not reported	Unclear/ Not reported	Rural	Community-based	Not applicable (if not in the hospital)	Not applicable (if not in the hospital)	Not applicable (for animals and healty participants)	Not applicable (for animals and healty participants)	Not applicable (for animals and healty participants)	Unclear/Not reported	Indirect immunofluorescence assay	Serum	Antibodies	At least one past contact	Humans	Apparently healthy individuals	Not applicable	Not applicable

Reference

1. Fisher-Hoch SP, Tomori O, Nasidi A, Perez-Oronoz GI, Fakile Y, Hutwagner L, et al. Review of cases of nosocomial Lassa fever in Nigeria: the high price of poor medical practice. BMJ. 1995;311: 857–859.

2. Kouadio L, Nowak K, Akoua-Koffi C, Weiss S, Allali BK, Witkowski PT, et al. Lassa Virus in Multimammate Rats, Côte d'Ivoire, 2013. Emerg Infect Dis. 2015;21: 1481–1483. doi:10.3201/eid2108.150312

3. Lecompte E, Fichet-Calvet E, Daffis S, Koulémou K, Sylla O, Kourouma F, et al. Mastomys natalensis and Lassa Fever, West Africa. Emerg Infect Dis. 2006;12: 1971–1974. doi:10.3201/eid1212.060812

4. Monath TP, Newhouse VF, Kemp GE, Setzer HW, Cacciapuoti A. Lassa virus isolation from Mastomys natalensis rodents during an epidemic in Sierra Leone. Science. 1974;185: 263–265. doi:10.1126/science.185.4147.263

5. Olayemi A, Cadar D, Magassouba N, Obadare A, Kourouma F, Oyeyiola A, et al. New Hosts of The Lassa Virus. Sci Rep. 2016;6: 25280. doi:10.1038/srep25280

6. Monath TP, Mertens PE, Patton R, Moser CR, Baum JJ, Pinneo L, et al. A hospital epidemic of Lassa fever in Zorzor, Liberia, March-April 1972. Am J Trop Med Hyg. 1973;22: 773–779. doi:10.4269/ajtmh.1973.22.773

7. Agbonlahor DE, Erah A, Agba IM, Oviasogie FE, Ehiaghe AF, Wankasi M, et al. Prevalence of Lassa virus among rodents trapped in three South-South States of Nigeria. J Vector Borne Dis. 2017; 5.

8. Akhuemokhan OC, Ewah-Odiase RO, Akpede N, Ehimuan J, Adomeh DI, Odia I, et al. Prevalence of Lassa Virus Disease (LVD) in Nigerian children with fever or fever and convulsions in an endemic area. PLoS Negl Trop Dis. 2017;11. doi:10.1371/journal.pntd.0005711

9. Akoua-Koffi C, Ter Meulen J, Legros D, Akran V, Aïdara M, Nahounou N, et al. [Detection of anti-Lassa antibodies in the Western Forest area of the Ivory Coast]. Med Trop (Mars). 2006;66: 465–468.

10. Arnold RB, Gary GW. A neutralization test survey for Lassa fever activity in Lassa, Nigeria. Transactions of the Royal Society of Tropical Medicine and Hygiene. 1977;71: 152–154. doi:10.1016/0035-9203(77)90085-2

11. Asogun DA, Adomeh DI, Ehimuan J, Odia I, Hass M, Gabriel M, et al. Molecular Diagnostics for Lassa Fever at Irrua Specialist Teaching Hospital, Nigeria: Lessons Learnt from Two Years of Laboratory Operation. PLoS Negl Trop Dis. 2012;6. doi:10.1371/journal.pntd.0001839

12. Babalola SO, Babatunde JA, Remilekun OM, Amaobichukwu AR, Abiodun AM, Jide I, et al. Lassa virus RNA detection from suspected cases in Nigeria, 2011-2017. Pan Afr Med J. 2019;34. doi:10.11604/pamj.2019.34.76.16425

13. Bajani MD, Tomori O, Rollin PE, Harry TO, Bukbuk ND, Wilson L, et al. A survey for antibodies to Lassa virus among health workers in Nigeria. Transactions of the Royal Society of Tropical Medicine and Hygiene. 1997;91: 379–381. doi:10.1016/S0035-9203(97)90247-9

14. Baumann J, Knüpfer M, Ouedraogo J, Traoré BY, Heitzer A, Kané B, et al. Lassa and Crimean-Congo Hemorrhagic Fever Viruses, Mali. Emerging Infect Dis. 2019;25: 999–1002. doi:10.3201/eid2505.181047

15. Bausch DG, Demby AH, Coulibaly M, Kanu J, Goba A, Bah A, et al. Lassa fever in Guinea: I. Epidemiology of human disease and clinical observations. Vector Borne Zoonotic Dis. 2001;1: 269–281. doi:10.1089/15303660160025903

16. Blackburn NK, Searle L, Taylor P. Viral haemorrhagic fever antibodies in Zimbabwe schoolchildren. Transactions of the Royal Society of Tropical Medicine and Hygiene. 1982;76: 803–805. doi:10.1016/0035-9203(82)90113-4

17. Boiro I, Lomonossov NN, Sotsinski VA, Constantinov OK, Tkachenko EA, Inapogui AP, et al. [Clinico-epidemiologic and laboratory research on hemorrhagic fevers in Guinea]. Bull Soc Pathol Exot Filiales. 1987;80: 607–612.

18. Bonney JHK, Osei-Kwasi M, Adiku TK, Barnor JS, Amesiya R, Kubio C, et al. Hospital-Based Surveillance for Viral Hemorrhagic Fevers and Hepatitides in Ghana. Kasper M, editor. PLoS Negl Trop Dis. 2013;7: e2435. doi:10.1371/journal.pntd.0002435

19. Bowen GS, Wulff H, Casals J, Noonan A, Downs WG. Lassa fever in Onitsha, East Central State, Nigeria, in 1974. 1975; 6.

20. Branco LM, Grove JN, Boisen ML, Shaffer JG, Goba A, Fullah M, et al. Emerging trends in Lassa fever: redefining the role of immunoglobulin M and inflammation in diagnosing acute infection. Virol J. 2011;8: 478. doi:10.1186/1743-422X-8-478

21. Buba MI, Dalhat MM, Nguku PM, Waziri N, Mohammad JO, Bomoi IM, et al. Mortality Among Confirmed Lassa Fever Cases During the 2015–2016 Outbreak in Nigeria. Am J Public Health. 2018;108: 262–264. doi:10.2105/AJPH.2017.304186

22. Bukbuk DN, Fukushi S, Tani H, Yoshikawa T, Taniguchi S, Iha K, et al. Development and validation of serological assays for viral hemorrhagic fevers and determination of the prevalence of Rift Valley fever in Borno State, Nigeria. Transactions of The Royal Society of Tropical Medicine and Hygiene. 2014;108: 768–773. doi:10.1093/trstmh/tru163

23. Carey DE, Kemp GE, White HA, Pinneo L, Addy RF, Fom ALMD, et al. Lassa fever Epidemiological aspects of the 1970 epidemic, Jos, Nigeria. Trans R Soc Trop Med Hyg. 1972;66: 402–408. doi:10.1016/0035-9203(72)90271-4

24. Clements TL, Rossi CA, Irish AK, Kibuuka H, Eller LA, Robb ML, et al. Chikungunya and O’nyong-nyong Viruses in Uganda: Implications for Diagnostics. Open Forum Infectious Diseases. 2019;6. doi:10.1093/ofid/ofz001

25. Coulibaly-N’Golo D, Allali B, Rieger T, Akoua-Koffi C. Novel Arenavirus Sequences in Hylomyscus sp. and Mus (Nannomys) setulosus from Coˆ te d’Ivoire: Implications for Evolution of Arenaviruses in Africa. PLoS ONE. 2011;6: 9.

26. Dan-Nwafor CC, Ipadeola O, Smout E, Ilori E, Adeyemo A, Umeokonkwo C, et al. A cluster of nosocomial Lassa fever cases in a tertiary health facility in Nigeria: Description and lessons learned, 2018. International Journal of Infectious Diseases. 2019;83: 88–94. doi:10.1016/j.ijid.2019.03.030

27. Dahmane A, van Griensven J, Van Herp M, Van den Bergh R, Nzomukunda Y, Prior J, et al. Constraints in the diagnosis and treatment of Lassa Fever and the effect on mortality in

hospitalized children and women with obstetric conditions in a rural district hospital in Sierra Leone. Trans R Soc Trop Med Hyg. 2014;108: 126–132. doi:10.1093/trstmh/tru009

28. Demartini JC, Green DE, Monath TP. Lassa virus infection in Mastomys natalensis in Sierra Leone. 1975; 12.

29. Demby AH, Inapogui A, Kargbo K, Koninga J, Kourouma K, Kanu J, et al. Lassa fever in Guinea: II. Distribution and prevalence of Lassa virus infection in small mammals. Vector Borne Zoonotic Dis. 2001;1: 283–297. doi:10.1089/15303660160025912

30. Demby AH, Chamberlain J, Brown DW, Clegg CS. Early diagnosis of Lassa fever by reverse transcription-PCR. Journal of Clinical Microbiology. 1994;32: 2898–2903. doi:10.1128/JCM.32.12.2898-2903.1994

31. Ehichioya DU, Asogun DA, Ehimuan J, Okokhere PO, Pahlmann M, Ölschläger S, et al. Hospital-based surveillance for Lassa fever in Edo State, Nigeria, 2005-2008: Lassa fever in Edo State, Nigeria. Tropical Medicine & International Health. 2012;17: 1001–1004. doi:10.1111/j.1365-3156.2012.03010.x

32. Emmerich P, Günther S, Schmitz H. Strain-specific antibody response to Lassa virus in the local population of west Africa. Journal of Clinical Virology. 2008;42: 40–44. doi:10.1016/j.jcv.2007.11.019

33. Emmerich P, Thome-Bolduan C, Drosten C, Gunther S, Ban E, Sawinsky I, et al. Reverse ELISA for IgG and IgM antibodies to detect Lassa virus infections in Africa. Journal of Clinical Virology. 2006;37: 277–281. doi:10.1016/j.jcv.2006.08.015

34. Fabiyi A, Tomori O, Pinneo P. Lassa fever antibodies in hospital personnel in the Plateau State of Nigeria. Niger Med J. 1979;9: 23–25.

35. Fabiyi A. Use of the complement fixation (CF) test in Lassa fever surveillance. 1975; 4.

36. Fair J, Jentes E, Inapogui A, Kourouma K, Goba A, Bah A, et al. Lassa Virus-Infected Rodents in Refugee Camps in Guinea: A Looming Threat to Public Health in a Politically Unstable Region. Vector-Borne and Zoonotic Diseases. 2007;7: 167–171. doi:10.1089/vbz.2006.0581

37. Fichet-Calvet E, Becker-Ziaja B, Koivogui L, Günther S. Lassa Serology in Natural Populations of Rodents and Horizontal Transmission. Vector-Borne and Zoonotic Diseases. 2014;14: 665–674. doi:10.1089/vbz.2013.1484

38. Fichet-Calvet E, Lecompte E, Koivogui L, Soropogui B, Doré A, Kourouma F, et al. Fluctuation of Abundance and Lassa Virus Prevalence in *Mastomys natalensis* in Guinea, West Africa. Vector-Borne and Zoonotic Diseases. 2007;7: 119–128. doi:10.1089/vbz.2006.0520

39. Fisher-Hoch S, McCormick JB, Sasso D, Craven RB. Hematologic dysfunction in Lassa fever. J Med Virol. 1988;26: 127–135. doi:10.1002/jmv.1890260204

40. Frame JD, Jahrling PB, Yalley-Ogunro JE, Monson MH. Endemic Lassa fever in Liberia. II. Serological and virological findings in hospital patients. Trans R Soc Trop Med Hyg. 1984;78: 656–660. doi:10.1016/0035-9203(84)90232-3

41. Frame JD, Casals J, Dennis EA. Lassa virus antibodies in hospital personnel in western Liberia. Transactions of the Royal Society of Tropical Medicine and Hygiene. 1979;73: 219–224. doi:10.1016/0035-9203(79)90218-9

42. Georges AJ, Gonzalez JP, Abdul-Wahid S, Saluzzo JF, Meunier DMY, McCormick JB. Antibodies to Lassa and lassa-like viruses in man and mammals in the Central African Republic. Transactions of the Royal Society of Tropical Medicine and Hygiene. 1985;79: 78–79. doi:10.1016/0035-9203(85)90242-1

43. Gonzalez JP, Josse R, Johnson ED, Merlin M, Georges AJ, Abandja J, et al. Antibody prevalence against haemorrhagic fever viruses in randomized representative central African populations. Research in Virology. 1989;140: 319–331. doi:10.1016/S0923-2516(89)80112-8

44. Hamblion EL, Raftery P, Wendland A, Dweh E, Williams GS, George RNC, et al. The challenges of detecting and responding to a Lassa fever outbreak in an Ebola-affected setting. International Journal of Infectious Diseases. 2018;66: 65–73. doi:10.1016/j.ijid.2017.11.007

45. Haun BK, Kamara V, Dweh AS, Garalde-Machida K, Forkay SSE, Takaaze M, et al. Serological evidence of Ebola virus exposure in dogs from affected communities in Liberia: A preliminary report. Rimoin AW, editor. PLoS Negl Trop Dis. 2019;13: e0007614. doi:10.1371/journal.pntd.0007614

46. Helmick C, Scribner C, Webb P, Krebs J, Mccormick J. NO EVIDENCE FOR INCREASED RISK OF LASSA FEVER INFECTION IN HOSPITAL STAFF. The Lancet. 1986;328: 1202–1205. doi:10.1016/S0140-6736(86)92206-3

47. Henderson BE, Gary GW, Kissling RE, Frame JD, Carey DE. Lassa fever virological and serological studies. Transactions of the Royal Society of Tropical Medicine and Hygiene. 1972;66: 409–416. doi:10.1016/0035-9203(72)90272-6

48. Ibekwe T, Nwegbu M, Okokhere P, Adomeh D, Asogun D. The sensitivity and specificity of Lassa virus IgM by ELISA as screening tool at early phase of Lassa fever infection. Niger Med J. 2012;53: 196. doi:10.4103/0300-1652.107552

49. Ipadeola O, Furuse Y, Ilori EA, Dan-Nwafor CC, Akabike KO, Ahumibe A, et al. Epidemiology and case-control study of Lassa fever outbreak in Nigeria from 2018 to 2019. Journal of Infection. 2020; S0163445320300013. doi:10.1016/j.jinf.2019.12.020

50. Isere EE, Fatiregun A, Ilesanmi O, Ijarotimi I, Egube B, Adejugbagbe A, et al. Lessons Learnt from Epidemiological Investigation of Lassa Fever Outbreak in a Southwest State of Nigeria December 2015 to April 2016. PLoS Curr. 2018 [cited 25 Feb 2020]. doi:10.1371/currents.outbreaks.bc4396a6650d0ed1985d731583bf5ded

51. Ivanoff' B, Duquesnoy' P, Languillat' G, Saluzzo' JF, Georges' A, Gonzalez' JP, et al. Haemorrhagic fever in Gabon. I. Incidence of Lassa, Ebola and Marburg viruses in Haut-Ogoou6. 1982; 2.

52. Jahrling PB, Frame JD, Smith SB, Monson MH. Endemic Lassa fever in Liberia. III. Characterization of Lassa virus isolates. Transactions of the Royal Society of Tropical Medicine and Hygiene. 1985;79: 374–379. doi:10.1016/0035-9203(85)90386-4

53. Johnson BK, Ocheng D, Gichogo A, Okiro M, Libondo D, Tukei PM, et al. Antibodies against haemorrhagic fever viruses in Kenya populations. Transactions of the Royal Society of Tropical Medicine and Hygiene. 1983;77: 731–733. doi:10.1016/0035-9203(83)90216-X

54. Johnson BK, Ocheng D, Gitau LG, Gichogo A, Tukei PM, Ngindu A, et al. Viral Haemorrhagic Fever Surveillance in Kenya, 1980-198. 1983; 1.

55. Johnson' BK, Gitau LG, Gichogop A, Tukei' M, Else' JG, Suleman MA, et al. Marburg, Ebota and Rift Valley fever virus antibodies in East African primates. Transactions of the Royal Society of Tropical Medicine and Hygiene. 1982; 4.

56. Keane E, Gilles HM. Lassa fever in Panguma Hospital, Sierra Leone, 1973-6. BMJ. 1977;1: 1399–1402. doi:10.1136/bmj.1.6073.1399

57. Kernéis S, Koivogui L, Magassouba N, Koulemou K, Lewis R, Aplogan A, et al. Prevalence and Risk Factors of Lassa Seropositivity in Inhabitants of the Forest Region of Guinea: A Cross-Sectional Study. Aksoy S, editor. PLoS Negl Trop Dis. 2009;3: e548. doi:10.1371/journal.pntd.0000548

58. Klempa B, Koulemou K, Auste B, Emmerich P, Thomé-Bolduan C, Günther S, et al. Seroepidemiological study reveals regional co-occurrence of Lassa- and Hantavirus antibodies in Upper Guinea, West Africa. Trop Med Int Health. 2012; n/a-n/a. doi:10.1111/tmi.12045

59. Lalis A, Leblois R, Lecompte E, Denys C, ter Meulen J, Wirth T. The Impact of Human Conflict on the Genetics of Mastomys natalensis and Lassa Virus in West Africa. Mores CN, editor. PLoS ONE. 2012;7: e37068. doi:10.1371/journal.pone.0037068

60. Leski TA, Stockelman MG, Moses LM, Park M, Stenger DA, Ansumana R, et al. Sequence Variability and Geographic Distribution of Lassa Virus, Sierra Leone - Volume 21, Number 4—April 2015 - Emerging Infectious Diseases journal - CDC. 2015 [cited 26 Oct 2019]. doi:10.3201/eid2104.141469

61. Li W-G, Chen W-W, Li L, Ji D, Ji Y-J, Li C, et al. The etiology of Ebola virus disease-like illnesses in Ebola virusnegative patients from Sierra Leone. Oncotarget. 2016;7. doi:10.18632/oncotarget.8558

62. Lukashevich IS, Clegg JC, Sidibe K. Lassa virus activity in Guinea: distribution of human antiviral antibody defined using enzyme-linked immunosorbent assay with recombinant antigen. J Med Virol. 1993;40: 210–217. doi:10.1002/jmv.1890400308

63. Maigari IM, Jibrin YB, Umar MS, Lawal SM, Gandi AY. Descriptive features of Lassa fever in Bauchi, Northeastern Nigeria - a retrospective review. Research Journal of Health Sciences. 2018;6: 149. doi:10.4314/rejhs.v6i3.7

64. Mariën J, Borremans B, Gryseels S, Soropogui B, De Bruyn L, Bongo GN, et al. No measurable adverse effects of Lassa, Morogoro and Gairo arenaviruses on their rodent reservoir host in natural conditions. Parasites Vectors. 2017;10: 210. doi:10.1186/s13071-017-2146-0

65. Mathiot CC, Fontenille D, Georges AJ, Coulanges P. Antibodies to haemorrhagic fever viruses in Madagascar populations. Transactions of the Royal Society of Tropical Medicine and Hygiene. 1989;83: 407–409. doi:10.1016/0035-9203(89)90519-1

66. McCarthy MC, Haberberger RL, Salib AW, Soliman BA, El-Tigani A, Watts DM. Evaluation of arthropod-borne viruses and other infectious disease pathogens as the causes of febrile illnesses in the Khartoum Province of Sudan. 1996; 6.

67. McCormick JB, King IJ, Webb PA, Johnson KM, O'Sullivan R, Smith ES, et al. A Case-Control Study of the Clinical Diagnosis and Course of Lassa Fever. J Infect Dis. 1987;155: 445–455. doi:10.1093/infdis/155.3.445

68. McCormick JB, Webb PA, Krebs JW, Johnson KM, Smith ES. A Prospective Study of the Epidemiology and Ecology of Lassa Fever. J Infect Dis. 1987;155: 437–444. doi:10.1093/infdis/155.3.437

69. Meunier DM, Johnson ED, Gonzalez JP, Georges-Courbot MC, Madelon MC, Georges AJ. [Current serologic data on viral hemorrhagic fevers in the Central African Republic]. Bull Soc Pathol Exot Filiales. 1987;80: 51–61.

70. Nakounné E, Selekon B, Morvan J. Veille microbiologique : les fièvres hémorragiques virales en République centrafricaine ; 2000; 8.

71. Nimo-Paintsil SC, Fichet-Calvet E, Borremans B, Letizia AG, Mohareb E, Bonney JHK, et al. Rodent-borne infections in rural Ghanaian farming communities. Schieffelin J, editor. PLoS ONE. 2019;14: e0215224. doi:10.1371/journal.pone.0215224

72. Ogunro BN, Olugasa BO, Kayode A, Ishola OO, Kolawole ON, Odigie EA, et al. Detection of Antibody and Antigen for Lassa Virus Nucleoprotein in Monkeys from Southern Nigeria. JEGH. 2019 [cited 25 Feb 2020]. doi:10.2991/jegh.k.190421.001

73. O'Hearn AE, Voorhees MA, Fetterer DP, Wauquier N, Coomber MR, Bangura J, et al. Serosurveillance of viral pathogens circulating in West Africa. Virol J. 2016;13: 163. doi:10.1186/s12985-016-0621-4

74. Okogbenin S, Okoeguale J, Akpede G, Colubri A, Barnes KG, Mehta S, et al. Retrospective Cohort Study of Lassa Fever in Pregnancy, Southern Nigeria. Emerg Infect Dis. 2019;25: 1494–1500. doi:10.3201/eid2508.181299

75. Okokhere P, Colubri A, Azubike C, Iruolagbe C, Osazuwa O, Tabrizi S, et al. Clinical and laboratory predictors of Lassa fever outcome in a dedicated treatment facility in Nigeria: a retrospective, observational cohort study. The Lancet Infectious Diseases. 2018;18: 684–695. doi:10.1016/S1473-3099(18)30121-X

76. Okoror LE, Esumeh FI, Agbonlahor DE, Umolu PI. Lassa virus: Seroepidemiological survey of rodents caught in Ekpoma and environs. Trop Doct. 2005;35: 16–17. doi:10.1258/0049475053001912

77. Olayemi A, Oyeyiola A, Obadare A, Igbokwe J, Adesina AS, Onwe F, et al. Widespread arenavirus occurrence and seroprevalence in small mammals, Nigeria. Parasites Vectors. 2018;11: 416. doi:10.1186/s13071-018-2991-5

78. Olayemi A, Obadare A, Oyeyiola A, Igbokwe J, Fasogbon A, Igbahenah F, et al. Arenavirus Diversity and Phylogeography of *Mastomys natalensis* Rodents, Nigeria. Emerg Infect Dis. 2016;22: 687–690. doi:10.3201/eid2204.150155

79. Oloniniyi OK, Unigwe US, Okada S, Kimura M, Koyano S, Miyazaki Y, et al. Genetic characterization of Lassa virus strains isolated from 2012 to 2016 in southeastern Nigeria. PLoS Negl Trop Dis. 2018;12. doi:10.1371/journal.pntd.0006971

80. Omilabu SA, Badaru SO, Okokhere P, Asogun D, Drosten C, Emmerich P, et al. Lassa Fever, Nigeria, 2003 and 2004. Emerg Infect Dis. 2005;11: 1642–1644. doi:10.3201/eid1110.041343

81. Paix MA, Poveda JD, Malvy D, Bailly C, Merlin M, Fleury HJ. [Serological study of the virus responsible for hemorrhagic fever in an urban population of Cameroon]. Bull Soc Pathol Exot Filiales. 1988;81: 679–682.

82. Panning M, Emmerich P, Ölschläger S, Bojenko S, Koivogui L, Marx A, et al. Laboratory Diagnosis of Lassa Fever, Liberia. Emerg Infect Dis. 2010;16: 1041–1043. doi:10.3201/eid1606.100040

83. Price ME, Fisher-Hoch SP, Craven RB, McCormick JB. A prospective study of maternal and fetal outcome in acute Lassa fever infection during pregnancy. 1988;297: 4.

84. Rodhain F, Gonzalez JP, Mercier E, Helynck B, Larouze B, Hannoun C. Arbovirus infections and viral haemorrhagic fevers in Uganda: a serological survey in Karamoja district, 1984. Transactions of the Royal Society of Tropical Medicine and Hygiene. 1989;83: 851–854. doi:10.1016/0035-9203(89)90352-0

85. Safronetz D, Sogoba N, Lopez JE, Maiga O, Dahlstrom E, Zivcec M, et al. Geographic Distribution and Genetic Characterization of Lassa Virus in Sub-Saharan Mali. PLoS Negl Trop Dis. 2013;7. doi:10.1371/journal.pntd.0002582

86. Safronetz D, Lopez JE, Sogoba N, Traore' SF, Raffel SJ, Fischer ER, et al. Detection of Lassa virus, Mali. Emerging Infect Dis. 2010;16: 1123–1126. doi:10.3201/eid1607.100146

87. Salu OB, James AB, Bankolé HS, Agbla JM, Da Silva M, Gbaguidi F, et al. Molecular confirmation and phylogeny of Lassa fever virus in Benin Republic 2014–2016. African Journal of Laboratory Medicine. 2019;8. doi:10.4102/ajlm.v8i1.803

88. Saluzzo JF, Adam F, McCormick JB, Digoutte JP. Lassa Fever Virus in Senegal. Journal of Infectious Diseases. 1988;157: 605–605. doi:10.1093/infdis/157.3.605

89. Schoepp RJ, Rossi CA, Khan SH, Goba A, Fair JN. Undiagnosed Acute Viral Febrile Illnesses, Sierra Leone. Emerg Infect Dis. 2014;20: 1176–1182. doi:10.3201/eid2007.131265

90. Shaffer JG, Schieffelin JS, Gbakie M, Alhasan F, Roberts NB, Goba A, et al. A medical records and data capture and management system for Lassa fever in Sierra Leone: Approach, implementation, and challenges. Verdonck K, editor. PLoS ONE. 2019;14: e0214284. doi:10.1371/journal.pone.0214284

91. Shehu NY, Gomerep SS, Isa SE, Iraoyah KO, Mafuka J, Bitrus N, et al. Lassa Fever 2016 Outbreak in Plateau State, Nigeria—The Changing Epidemiology and Clinical Presentation. Front Public Health. 2018;6: 232. doi:10.3389/fpubh.2018.00232

92. Smith EA, Fabiyi A, Kuteyi OE, Tomori O. Epidemiological aspect of the 1976 Pankshin Lassa fever outbreak. Niger Med J. 1979;9: 20–22.

93. Sogoba N, Rosenke K, Adjemian J, Diawara SI, Maiga O, Keita M, et al. Lassa Virus Seroprevalence in Sibirilia Commune, Bougouni District, Southern Mali. Emerg Infect Dis. 2016;22: 657–663. doi:10.3201/eid2204.151814

94. ter Meulen J, Koulemou K, Wittekindt T, Windisch K, Strigl S, Conde S, et al. Detection of Lassa Virus Antinucleoprotein Immunoglobulin G (IgG) and IgM Antibodies by a Simple Recombinant Immunoblot Assay for Field Use. Journal of Clinical Microbiology. 1998;36: 3143–3148. doi:10.1128/JCM.36.11.3143-3148.1998

95. Tomori O, Fabiyi A, Sorungbe A, Smith A, McCormick JB. Viral hemorrhagic fever antibodies in Nigerian populations. Am J Trop Med Hyg. 1988;38: 407–410. doi:10.4269/ajtmh.1988.38.407

96. Van der Waals FW, Pomeroy KL, Goudsmit J, Asher DM, Gajdusek DC. Hemorrhagic fever virus infections in an isolated rainforest area of central Liberia. Limitations of the indirect immunofluorescence slide test for antibody screening in Africa. Trop Geogr Med. 1986;38: 209–214.

97. Webb PA, McCormick JB, King IJ, Bosman I, Johnson KM, Elliott LH, et al. Lassa fever in children in Sierra Leone, West Africa. Trans R Soc Trop Med Hyg. 1986;80: 577–582. doi:10.1016/0035-9203(86)90147-1

98. Wulff H, Fabiyi A, Monath TP. Recent isolations of Lassa virus from Nigerian rodents. Bull World Health Organ. 1975;52: 609–613.

99. Yadouleton A, Agolinou A, Kourouma F, Saizonou R, Pahlmann M, Bedié SK, et al. Lassa Virus in Pygmy Mice, Benin, 2016–2017. Emerg Infect Dis. 2019;25: 1977–1979. doi:10.3201/eid2510.180523

100. Yalley-Ogunro JE, Frame JD, Hanson AP. Endemic Lassa fever in Liberia. VI. Village serological surveys for evidence of Lassa virus activity in Lofa County, Liberia. Transactions of the Royal Society of Tropical Medicine and Hygiene. 1984;78: 764–770. doi:10.1016/0035-9203(84)90013-0

101. Cummins D, McCormick JB, Bennett D, Samba JA, Farrar B, Machin SJ, et al. Acute sensorineural deafness in Lassa fever. JAMA. 1990;264: 2093–2096.

102. Frame JD, Yalley-Ogunro JE, Hanson AP. Endemic Lassa fever in Liberia. V. Distribution of Lassa virus activity in Liberia: hospital staff surveys. Transactions of the Royal Society of Tropical Medicine and Hygiene. 1984;78: 761–763. doi:10.1016/0035-9203(84)90012-9

103. Gonzalez JP, McCormick JB, Saluzzo JF, Herve JP, Georges AJ, Johnson KM. An arenavirus isolated from wild-caught rodents (Pramys species) in the Central African Republic. Intervirology. 1983;19: 105–112. doi:10.1159/000149344

104. Keenlyside RA, McCormick JB, Webb PA, Smith E, Elliott L, Johnson KM. Case-control study of Mastomys natalensis and humans in Lassa virus-infected households in Sierra Leone. Am J Trop Med Hyg. 1983;32: 829–837. doi:10.4269/ajtmh.1983.32.829

105. Meulen JT, Lukashevich I, Sidibe K, Inapogui A, Marx M, Dorlemann A, et al. Hunting of Peridomestic Rodents and Consumption of Their Meat as Possible Risk Factors for Rodent-to-Human Transmission of Lassa Virus in the Republic of Guinea. The American Journal of Tropical Medicine and Hygiene. 1996;55: 661–666. doi:10.4269/ajtmh.1996.55.661

106. Troup JM, White HA, Fom AL, Carey DE. An outbreak of Lassa fever on the Jos plateau, Nigeria, in January-February 1970. A preliminary report. Am J Trop Med Hyg. 1970;19: 695–696. doi:10.4269/ajtmh.1970.19.695

107. Bloch A. A serological survey of Lassa fever in Liberia. 1978; 3.

108. Dan-Nwafor CC, Furuse Y, Ilori EA, Ipadeola O, Akabike KO, Ahumibe A, et al. Measures to control protracted large Lassa fever outbreak in Nigeria, 1 January to 28 April 2019. Eurosurveillance. 2019;24. doi:10.2807/1560-7917.ES.2019.24.20.1900272

109. Johnson ED, Gonzalez JP, Georges A. Haemorrhagic fever virus activity in equatorial Africa: distribution and prevalence of filovirus reactive antibody in the Central African Republic. Transactions of the Royal Society of Tropical Medicine and Hygiene. 1993;87: 530–535. doi:10.1016/0035-9203(93)90075-2
