# Supplementary material for: Systematic review and meta-analysis of the epidemiology of Lassa virus in humans, rodents and other mammals in sub-Saharan Africa
Source: PLoS Negl Trop Dis. 2020 Aug 26;14(8):e0008589. doi: 10.1371/journal.pntd.0008589 (PMC7478710; doi:10.1371/journal.pntd.0008589)
Supplement: S8 Table — (PDF) [file pntd.0008589.s008.pdf]

S8 Table: Subgroup of human case fatality rate and prevalence of Lassa virus in humans. rodents. and other animals in sub-Saharan Africa

	Prevalence. % (95%CI)	95% Prediction interval	N Studies	N Participants	H (95%CI)	I² (95%CI)	P heterogeneity	P difference subtypes
**Case fatality rate in humans**								
**Current contact**								
**Positive among LASV suspected cases**								
**Study Design**								0.036
Cohort (Baseline data)	23.9 [19.1-29.1]	NA	1	284	NA	NA	1	
Community outbreak	39.5 [8.4-76.3]	NA	2	601	5.1 [3.1-8.5]	96.2 [89.3-98.6]	< 0.001	
Cross-sectional	35 [17-55.5]	[0-96.7]	7	1778	5.8 [4.7-7.1]	97 [95.5-98]	< 0.001	
Hospital outbreak	35.4 [28.3-42.8]	NA	2	181	1 NA	0	0.919	
**Timing of data collection**								0.018
Prospetively	23.2 [18.4-28.4]	[10.3-39.3]	6	2194	1.9 [1.3-2.9]	73.7 [39.8-88.5]	0.002	
Retrospectively	49 [27.8-70.4]	[0-100]	6	650	4.8 [3.7-6.2]	95.6 [92.8-97.4]	< 0.001	
**Country**								0.927
Liberia	28.6 [7.4-55.4]	NA	1	14	NA	NA	1	
Nigeria	31.2 [24.8-38]	[12.9-53]	7	2447	2.7 [1.9-3.7]	85.8 [72.7-92.6]	< 0.001	
Sierra Leone	37.3 [9.5-70.6]	[0-100]	4	383	5.7 [4.2-7.6]	96.9 [94.4-98.3]	< 0.001	
**Recrutment Setting**								< 0.001
Rural	61.1 [44.5-76.5]	NA	1	36	NA	NA	1	
Urban	19.8 [11.5-29.7]	NA	2	354	1.8 [1-3.7]	68.1 [0-92.8]	0.077	
Urban/rural	56.2 [26.7-83.6]	NA	2	256	2 [1-4.2]	75.2 [0-94.4]	0.044	
**Setting**								< 0.001
Community-based	59.6 [45.1-73.3]	NA	1	47	NA	NA	1	
Hospital-based	36 [20.4-53.2]	[0-91.3]	8	875	4.7 [3.8-5.8]	95.4 [92.9-97]	< 0.001	
Hospital/community based	23.2 [20.9-25.5]	NA	2	1368	1 NA	0	0.613	
**Hospitalization**								0.665

	Prevalence. % (95%CI)	95% Prediction interval	N Studies	N Participants	H (95%CI)	I² (95%CI)	P heterogeneity	P difference subtypes
Hospitalized	36 [20.4-53.2]	[0-91.3]	8	875	4.7 [3.8-5.8]	95.4 [92.9-97]	< 0.001	
Hospitalized/ambulatory	28.6 [7.4-55.4]	NA	1	14	NA	NA	1	
**LASV prevalence in humans**								
**Current contact**								
**Febrile patients**								
**Study Design**								0.391
Case-control	5.3 [2.8-8.6]	NA	1	243	NA	NA	1	
Cross-sectional	2.6 [0.3-6.6]	[0-23.7]	7	1927	4.1 [3.2-5.3]	94.2 [90.3-96.5]	< 0.001	
Hospital outbreak	6.5 [0.1-18.5]	NA	1	31	NA	NA	1	
**Timing of data collection**								0.702
Prospetively	2.6 [0.3-6.8]	[0-24.1]	7	1695	3.9 [3-5.1]	93.4 [88.8-96.1]	< 0.001	
Retrospectively	5.4 [0-30.5]	NA	2	506	4.9 [2.9-8.3]	95.9 [88.3-98.6]	< 0.001	
**Country**								< 0.001
Ghana	0 [0-0.7]	NA	1	258	NA	NA	1	
Guinea	0 [0-0.7]	NA	1	253	NA	NA	1	
Liberia	8 [4.4-12.5]	NA	1	176	NA	NA	1	
Mali	0.4 [0-1.2]	NA	1	489	NA	NA	1	
Nigeria	2.8 [0-9.5]	[0-100]	3	725	3.1 [1.9-5.1]	89.8 [72.6-96.2]	< 0.001	
Sierra Leone	9.4 [6-13.4]	NA	1	245	NA	NA	1	
**Recrutment Setting**								0.049
Rural	0 [0-0.7]	NA	1	253	NA	NA	1	
Urban	2.8 [0-9.6]	[0-100]	3	763	3.2 [2-5.3]	90.4 [74.7-96.4]	< 0.001	
**Setting**								< 0.001
Community-based	0 [0-0.7]	NA	1	253	NA	NA	1	
Hospital-based	2.8 [0.5-6.7]	[0-22.4]	7	1893	3.8 [2.9-5]	93.1 [88.3-95.9]	< 0.001	
Hospital/community based	16.4 [7.6-27.5]	NA	1	55	NA	NA	1	
**Hospitalization**								0.001

	Prevalence. % (95%CI)	95% Prediction interval	N Studies	N Participants	H (95%CI)	I^2 (95%CI)	P heterogeneity	P difference subtypes
Ambulatory	0 [0-0.7]	NA	1	258	NA	NA	1	
Hospitalized	4.9 [1.4-10.1]	[0-29.2]	7	1690	3.8 [2.9-5]	93.1 [88.2-95.9]	< 0.001	
**LASV suspected cases**								
**Study Design**								< 0.001
Case-control	51.4 [39.8-62.9]	NA	1	72	NA	NA	1	
Community outbreak	23.8 [22.1-25.6]	NA	1	2323	NA	NA	1	
Cross-sectional	24.8 [19.6-30.4]	[6.2-50.2]	16	15929	6.5 [5.8-7.4]	97.7 [97-98.2]	< 0.001	
Hospital outbreak	19.9 [4.1-43.2]	NA	2	1684	3 [1.5-5.8]	88.7 [57.3-97]	0.003	
**Timing of data collection**								0.24
Prospetively	27.3 [21.3-33.8]	[7.2-54.2]	13	13667	6.6 [5.7-7.5]	97.7 [97-98.2]	< 0.001	
Retrospectively	21.5 [14.6-29.2]	[2.7-50.6]	7	6341	5.2 [4.2-6.5]	96.3 [94.3-97.6]	< 0.001	
**Country**								0.247
Liberia	17.9 [6.3-33.5]	[0-92.9]	4	1692	5.4 [4-7.3]	96.5 [93.7-98.1]	< 0.001	
Nigeria	22.6 [17.8-27.7]	[7.8-42.1]	10	13257	5.3 [4.4-6.3]	96.4 [94.8-97.5]	< 0.001	
Sierra Leone	36.2 [19.5-54.9]	[0-94.8]	6	5059	7.9 [6.5-9.5]	98.4 [97.7-98.9]	< 0.001	
**Recrutment Setting**								0.048
Rural	32 [1.4-77.2]	[0-100]	3	1438	9.8 [7.6-12.6]	99 [98.3-99.4]	< 0.001	
Urban	8.9 [7.4-10.5]	NA	1	1263	NA	NA	1	
Urban/rural	19.9 [9.3-33]	[0-84.1]	4	4476	3.7 [2.5-5.4]	92.7 [84.5-96.6]	< 0.001	
**Setting**								0.023
Hospital-based	21.4 [16.7-26.6]	[5.6-43.6]	14	9453	4.9 [4.2-5.8]	95.9 [94.4-97]	< 0.001	
Hospital/community based	37.5 [24.4-51.5]	[1.1-86.8]	5	8232	5.6 [4.4-7.3]	96.8 [94.7-98.1]	< 0.001	
**Hospitalization**								0.887
Ambulatory	24 [20.5-27.7]	NA	1	546	NA	NA	1	
Hospitalized	23.6 [18-29.7]	[5.8-48.4]	12	9056	5.4 [4.6-6.4]	96.6 [95.4-97.5]	< 0.001	
Hospitalized/ambulatory	27.2 [14-42.6]	NA	2	129	1.7 [1-3.6]	66.5 [0-92.4]	0.084	
**Recent contact**								

	Prevalence. % (95%CI)	95% Prediction interval	N Studies	N Participants	H (95%CI)	I² (95%CI)	P heterogeneity	P difference subtypes
**Apparently healthy individuals**								
**Study Design**								0.928
Cross-sectional	12.8 [3.9-25.8]	[0-69.5]	6	3435	9.7 [8.3-11.4]	98.9 [98.5-99.2]	< 0.001	
Hospital outbreak	11.8 [0.3-32.3]	NA	1	17	NA	NA	1	
**Timing of data collection**								0.926
Prospetively	12.8 [1.4-31.9]	[0-99.5]	4	1691	7.9 [6.3-10]	98.4 [97.4-99]	< 0.001	
Retrospectively	12.3 [1.2-32.6]	[0-100]	3	1761	10.5 [8.3-13.3]	99.1 [98.5-99.4]	< 0.001	
**Country**								< 0.001
Mali	0.7 [0.1-1.5]	NA	1	600	NA	NA	1	
Nigeria	3.7 [0-19.6]	NA	2	432	2.1 [1-4.4]	77.1 [0-94.8]	0.037	
Sierra Leone	45.6 [36.5-54.8]	NA	1	114	NA	NA	1	
**Recrutment Setting**								0.015
Rural	0.7 [0.1-1.5]	NA	1	600	NA	NA	1	
Urban	11.8 [0.3-32.3]	NA	1	17	NA	NA	1	
**Setting**								0.964
Community-based	11.5 [0.4-34.3]	[0-100]	3	1946	12.6 [10.2-15.5]	99.4 [99-99.6]	< 0.001	
Hospital-based	11.8 [0.3-32.3]	NA	1	17	NA	NA	1	
Hospital/community based	17.4 [0-74.2]	NA	2	529	11.7 [8.6-15.9]	99.3 [98.6-99.6]	< 0.001	
**Febrile patients**								
**Study Design**								0.036
Case-control	40.6 [37.7-43.5]	NA	1	1087	NA	NA	1	
Cross-sectional	10.1 [0-33.5]	NA	2	482	6.5 [4.2-10.2]	97.7 [94.3-99]	< 0.001	
Hospital outbreak	32.3 [16.8-49.9]	NA	1	31	NA	NA	1	
**Country**								0.371
Nigeria	32.3 [16.8-49.9]	NA	1	31	NA	NA	1	
Sierra Leone	18.7 [1.9-47]	[0-100]	3	1569	10.4 [8.2-13.3]	99.1 [98.5-99.4]	< 0.001	

	Prevalence. % (95%CI)	95% Prediction interval	N Studies	N Participants	H (95%CI)	I² (95%CI)	P heterogeneity	P difference subtypes
**Recrutment Setting**								0.159
Rural	40.6 [37.7-43.5]	NA	1	1087	NA	NA	1	
Urban	13.5 [0-52.4]	NA	2	268	4.6 [2.6-7.9]	95.2 [85.6-98.4]	< 0.001	
**LASV suspected cases**								
**Study Design**								0.169
Case-control	40.3 [29.2-51.9]	NA	1	72	NA	NA	1	
Cross-sectional	19.3 [1.9-48.1]	[0-100]	5	1326	10.6 [9-12.6]	99.1 [98.8-99.4]	< 0.001	
**Timing of data collection**								0.533
Prospetively	30.2 [7.2-60.3]	[0-100]	3	317	5.1 [3.5-7.5]	96.2 [92-98.2]	< 0.001	
Retrospectively	15.6 [0-58.7]	[0-100]	3	1081	14.3 [11.8-17.4]	99.5 [99.3-99.7]	< 0.001	
**Country**								< 0.001
Liberia	9.2 [5.4-13.9]	NA	1	184	NA	NA	1	
Nigeria	11.7 [0-71.5]	NA	2	523	10.5 [7.5-14.7]	99.1 [98.2-99.5]	< 0.001	
Sierra Leone	36.6 [27.4-46.4]	[0-100]	3	691	1.9 [1-3.5]	72.6 [7.8-91.9]	0.026	
**Setting**								0.054
Hospital-based	14 [0.5-39.9]	[0-100]	4	791	7.6 [6-9.7]	98.3 [97.2-98.9]	< 0.001	
Hospital/community based	40.8 [31.9-49.9]	NA	2	607	1.5 [1-3]	54.4 [0-88.8]	0.139	
**Hospitalization**								0.447
Ambulatory	37.7 [33.7-41.8]	NA	1	546	NA	NA	1	
Hospitalized	22.8 [0.4-62.7]	[0-100]	4	668	8.9 [7.2-11.1]	98.8 [98.1-99.2]	< 0.001	
**Past contact**								
**Apparently healthy individuals**								
**Study Design**								< 0.001
Case-control	26 [23.3-28.9]	NA	1	953	NA	NA	1	
Cross-sectional	3.4 [1.2-6.5]	[0-37.4]	46	37989	12.7 [12.2-13.3]	99.4 [99.3-99.4]	< 0.001	
Hospital outbreak	11.8 [0.3-32.3]	NA	1	17	NA	NA	1	

	Prevalence. % (95%CI)	95% Prediction interval	N Studies	N Participants	H (95%CI)	I² (95%CI)	P heterogeneity	P difference subtypes
**Sampling**								0.887
Non probabilistic	3.8 [1.2-7.4]	[0-39.1]	38	25763	11.5 [10.9-12.1]	99.2 [99.2-99.3]	< 0.001	
Probabilistic	4 [0.2-11.9]	[0-50.6]	10	13196	16.6 [15.3-18.1]	99.6 [99.6-99.7]	< 0.001	
**Country**								< 0.001
Benin	0 [0-11.9]	NA	1	14	NA	NA	1	
Burkina Faso	0 [0-6.8]	NA	1	25	NA	NA	1	
Cameroon	0.1 [0-0.4]	NA	2	1527	1 NA	0	0.489	
Central African Republic	0.1 [0-0.4]	[0-1.3]	5	7916	1.8 [1.1-2.8]	68.1 [17.6-87.6]	0.014	
Chad	0 [0-0.5]	NA	1	334	NA	NA	1	
Democratic Republic of the Congo	0 [0-2.6]	NA	1	67	NA	NA	1	
Equatorial Guinea	0.1 [0-0.6]	NA	1	688	NA	NA	1	
Ethiopia	0 [0-1.6]	NA	1	108	NA	NA	1	
Gabon	0 [0-0]	[0-1]	3	2104	1 [1-2.4]	0 [0-82]	0.561	
Ghana	5.2 [3.6-7]	NA	1	657	NA	NA	1	
Guinea	17.2 [10.5-25]	[0-57.5]	4	5103	5.4 [4-7.3]	96.6 [93.7-98.1]	< 0.001	
Ivory Coast	0 [0-10.5]	NA	1	16	NA	NA	1	
Kenya	0 [0-0]	NA	2	1929	1 NA	0	0.388	
Liberia	2.8 [0.5-6.4]	[0-84.2]	3	2043	2 [1.1-3.6]	74.7 [16-92.4]	0.019	
Madagascar	0 [0-0.5]	NA	1	381	NA	NA	1	
Mali	12.7 [0-58.1]	NA	2	617	4.1 [2.3-7.3]	94 [81-98.1]	< 0.001	
Niger	0 [0-4.6]	NA	1	37	NA	NA	1	
Nigeria	7 [0.6-18.4]	[0-64.9]	5	2721	7.4 [6-9.2]	98.2 [97.2-98.8]	< 0.001	
Republic of the Congo	0.4 [0.1-1]	NA	1	728	NA	NA	1	
Sierra Leone	40.9 [27.9-54.6]	[0-95.6]	4	7304	9.2 [7.4-11.4]	98.8 [98.2-99.2]	< 0.001	
Tanzania	0 [0-5.3]	NA	1	32	NA	NA	1	
Uganda	1.8 [0-12]	NA	2	1876	4.8 [2.8-8.2]	95.7 [87.7-98.5]	< 0.001	
Zimbabwe	0.2 [0-0.9]	NA	1	486	NA	NA	1	

	Prevalence. % (95%CI)	95% Prediction interval	N Studies	N Participants	H (95%CI)	I² (95%CI)	P heterogeneity	P difference subtypes
**UNSD Region**								< 0.001
Central Africa	0 [0-0]	[0-0.2]	14	13364	1.3 [1-1.8]	41.6 [0-68.9]	0.052	
Eastern Africa	0 [0-0.5]	[0-2.6]	8	4812	2.1 [1.5-3]	77.9 [56.5-88.8]	< 0.001	
West Africa	11.3 [7.6-15.6]	[0-37.7]	26	20783	8.1 [7.4-8.7]	98.5 [98.2-98.7]	< 0.001	
**Recrutment Setting**								0.122
Rural	2.4 [0.3-5.7]	[0-36.2]	35	27563	12.4 [11.8-13.1]	99.4 [99.3-99.4]	< 0.001	
Urban	0 [0-2]	[0-85.4]	3	645	2.1 [1.1-3.7]	76.4 [22.6-92.8]	0.015	
Urban/rural	8.4 [0.3-25.4]	[0-100]	3	4484	13.3 [10.8-16.3]	99.4 [99.1-99.6]	< 0.001	
**Setting**								0.001
Community-based	2.1 [0.3-4.9]	[0-32.3]	38	30819	11.9 [11.3-12.6]	99.3 [99.2-99.4]	< 0.001	
Hospital-based	2.2 [0-24.5]	NA	2	270	2.8 [1.4-5.6]	87.4 [51.1-96.8]	0.005	
Hospital/community based	23.2 [10.8-38.5]	[0-81.2]	6	5166	10.1 [8.7-11.8]	99 [98.7-99.3]	< 0.001	
**Apparently healthy individuals. Patient with any illness**								
**Study Design**								0.026
Case-control	2 [0.7-3.9]	NA	1	300	NA	NA	1	
Cross-sectional	0.3 [0-1.1]	[0-33.1]	3	2864	2.1 [1.2-3.9]	78.4 [30.4-93.3]	0.01	
**Country**								< 0.001
Central African Republic	0.4 [0.2-0.8]	NA	1	1898	NA	NA	1	
Kenya	0 [0-0.2]	NA	1	741	NA	NA	1	
Liberia	1.3 [0.2-3.4]	NA	1	225	NA	NA	1	
Sudan	2 [0.7-3.9]	NA	1	300	NA	NA	1	
**UNSD Region**								< 0.001
Central Africa	0.4 [0.2-0.8]	NA	1	1898	NA	NA	1	
Eastern Africa	0 [0-0.2]	NA	1	741	NA	NA	1	
Northern Africa	2 [0.7-3.9]	NA	1	300	NA	NA	1	

	Prevalence. % (95%CI)	95% Prediction interval	N Studies	N Participants	H (95%CI)	I² (95%CI)	P heterogeneity	P difference subtypes
West Africa	1.3 [0.2-3.4]	NA	1	225	NA	NA	1	
**Recrutment Setting**								0.01
Rural	0.4 [0.2-0.8]	NA	1	1898	NA	NA	1	
Urban	2 [0.7-3.9]	NA	1	300	NA	NA	1	
**Setting**								0.014
Community-based	0.4 [0.2-0.8]	NA	1	1898	NA	NA	1	
Hospital-based	2 [0.7-3.9]	NA	1	300	NA	NA	1	
Hospital/community based	1.3 [0.2-3.4]	NA	1	225	NA	NA	1	
**Febrile patients**								
**Study Design**								0.558
Community outbreak	14.8 [8.7-22]	NA	2	119	1 NA	0	0.938	
Cross-sectional	19.9 [9.6-32.6]	[0-69.5]	7	1291	5.1 [4.1-6.3]	96.1 [93.9-97.5]	< 0.001	
Hospital outbreak	22.6 [9.3-39.2]	NA	1	31	NA	NA	1	
**Sampling**								< 0.001
Non probabilistic	21.2 [13.4-30.3]	[0.9-55.8]	9	1144	3.3 [2.5-4.2]	90.6 [84.3-94.3]	< 0.001	
Probabilistic	7.4 [4.7-10.7]	NA	1	297	NA	NA	1	
**Timing of data collection**								0.499
Prospetively	19.5 [10.4-30.6]	[0-63.4]	9	1344	4.4 [3.6-5.4]	94.9 [92.1-96.6]	< 0.001	
Retrospectively	15.5 [8.9-23.4]	NA	1	97	NA	NA	1	
**Country**								< 0.001
Central African Republic	0 [0-5.8]	NA	1	29	NA	NA	1	
Guinea	32.1 [22.9-42]	[2.4-74]	4	574	2.1 [1.3-3.4]	76.8 [36.8-91.5]	0.005	
Liberia	17 [11.8-23]	NA	1	176	NA	NA	1	
Nigeria	13.1 [5.5-23.1]	[0-100]	3	425	2.2 [1.2-3.9]	78.8 [32-93.4]	0.009	
Sierra Leone	25.7 [20.4-31.5]	NA	1	237	NA	NA	1	
**UNSD Region**								< 0.001
Central Africa	0 [0-5.8]	NA	1	29	NA	NA	1	

	Prevalence. % (95%CI)	95% Prediction interval	N Studies	N Participants	H (95%CI)	I² (95%CI)	P heterogeneity	P difference subtypes
West Africa	22.1 [13.2-32.5]	[0.1-62.5]	9	1412	4.2 [3.3-5.2]	94.2 [91.1-96.3]	< 0.001	
**Recrutment Setting**								0.189
Rural	21.6 [7.9-39.3]	[0-96.9]	4	557	3.9 [2.7-5.6]	93.4 [86.3-96.8]	< 0.001	
Urban	25.1 [20-30.6]	NA	2	268	1 NA	0	0.767	
Urban/rural	17.3 [11.4-24.1]	NA	2	143	1 NA	0	0.35	
**Setting**								0.758
Community-based	24 [8.4-44.1]	[0-100]	3	535	4.5 [3-6.7]	95 [88.6-97.8]	< 0.001	
Hospital-based	16.5 [8.3-26.5]	[0-57.9]	5	763	3 [2.1-4.4]	89 [77-94.7]	< 0.001	
Hospital/community based	17.3 [11.4-24.1]	NA	2	143	1 NA	0	0.35	
**Hospitalization**								0.498
Ambulatory	12.9 [2.2-30.1]	NA	2	343	2.7 [1.4-5.4]	86.4 [46-96.6]	0.007	
Hospitalized	21.5 [15.3-28.4]	[0-93.7]	3	444	1.5 [1-2.8]	55.7 [0-87.4]	0.105	
Hospitalized/ambulatory	13.6 [1.9-31.7]	NA	1	22	NA	NA	1	
**Healthcare workers**								
**Study Design**								0.191
Community outbreak	0 [0-7.7]	NA	1	22	NA	NA	1	
Cross-sectional	8.3 [5.3-11.9]	[0.3-24.7]	11	5940	4.5 [3.7-5.4]	95 [92.7-96.5]	< 0.001	
Hospital outbreak	3.9 [0-14]	[0-100]	3	393	2.7 [1.6-4.6]	86 [59.2-95.2]	0.001	
**Timing of data collection**								0.515
Prospetively	7.3 [4-11.3]	[0-25.9]	12	4318	4.1 [3.4-4.9]	94 [91.2-95.9]	< 0.001	
Retrospectively	5.6 [1.5-12]	[0-100]	3	2037	5 [3.5-7.4]	96.1 [91.6-98.2]	< 0.001	
**Country**								0.046
Guinea	11.1 [8.2-14.3]	NA	1	406	NA	NA	1	
Liberia	7.3 [3.8-11.8]	[0-27.9]	5	1874	2.9 [2-4.2]	88.1 [74.9-94.4]	< 0.001	
Nigeria	4.1 [1.4-7.7]	[0-19.2]	7	3409	3.7 [2.8-4.8]	92.5 [87.1-95.7]	< 0.001	
Sierra Leone	13.8 [2.8-30.9]	NA	2	666	4.8 [2.8-8.1]	95.6 [87.4-98.5]	< 0.001	
**Recrutment Setting**								0.454

	Prevalence. % (95%CI)	95% Prediction interval	N Studies	N Participants	H (95%CI)	I² (95%CI)	P heterogeneity	P difference subtypes
Rural	8 [1.2-19.7]	[0-80]	4	824	4.4 [3.1-6.2]	94.8 [89.7-97.4]	< 0.001	
Urban	18.3 [2.4-42.3]	NA	2	564	1.9 [1-4]	71.7 [0-93.6]	0.06	
Urban/rural	7.6 [2.7-14.7]	NA	2	1476	4 [2.2-7.1]	93.6 [79.5-98]	< 0.001	
**Setting**								0.016
Community-based	1.4 [0-4.6]	NA	2	142	1 NA	0	0.569	
Hospital-based	8.5 [5.5-12.1]	[0.8-22.3]	8	3565	3 [2.3-4]	89.2 [81.1-93.8]	< 0.001	
Hospital/community based	6.4 [1.5-14.1]	[0-48]	5	2648	6.5 [5.2-8.2]	97.7 [96.3-98.5]	< 0.001	
**Hospitalization**								0.869
Ambulatory	7.3 [4.4-10.9]	[0-24.5]	13	6163	4.5 [3.8-5.4]	95.2 [93.2-96.6]	< 0.001	
Hospitalized/ambulatory	7.1 [3.6-11.5]	NA	1	170	NA	NA	1	
**High risk individuals**								
**Country**								< 0.001
Ivory Coast	26.1 [19.6-33.2]	NA	1	161	NA	NA	1	
Kenya	0 [0-2.9]	NA	1	58	NA	NA	1	
Nigeria	8.2 [5.2-11.7]	NA	1	281	NA	NA	1	
**UNSD Region**								0.012
Eastern Africa	0 [0-2.9]	NA	1	58	NA	NA	1	
West Africa	16 [2.8-36.9]	NA	2	442	5 [2.9-8.4]	95.9 [88.5-98.6]	< 0.001	
**Setting**								< 0.001
Community-based	8.2 [5.2-11.7]	NA	1	281	NA	NA	1	
Hospital/community based	26.1 [19.6-33.2]	NA	1	161	NA	NA	1	
**LASV positive case contact**								
**Study Design**								0.916
Community outbreak	4.3 [0.8-10.1]	NA	2	193	1.4 NA	49.5	0.159	
Hospital outbreak	3.3 [0-14.5]	NA	2	102	1.8 [1-3.7]	67.5 [0-92.7]	0.079	
**Country**								0.196
Liberia	7.9 [2.7-15.2]	NA	1	76	NA	NA	1	

	Prevalence. % (95%CI)	95% Prediction interval	N Studies	N Participants	H (95%CI)	I² (95%CI)	P heterogeneity	P difference subtypes
Nigeria	3 [0.3-7.4]	[0-78.3]	3	219	1.3 [1-2.2]	37.3 [0-80.2]	0.203	
**LASV suspected cases**								
**Timing of data collection**								0.273
Prospetively	25.5 [6-52.3]	[0-100]	3	1083	8.2 [6.2-10.9]	98.5 [97.4-99.2]	< 0.001	
Retrospectively	8.4 [0-32.7]	NA	2	715	8.2 [5.6-12.1]	98.5 [96.8-99.3]	< 0.001	
**Country**								0.25
Guinea	15.4 [11.6-19.7]	NA	1	311	NA	NA	1	
Nigeria	6.8 [0-25.9]	NA	2	548	4.9 [2.9-8.2]	95.8 [87.8-98.5]	< 0.001	
Sierra Leone	33.2 [9-63.7]	NA	2	939	8.7 [6-12.6]	98.7 [97.2-99.4]	< 0.001	
**Recrutment Setting**								< 0.001
Rural	17.2 [13.6-21.2]	NA	2	575	1.2 NA	33.5	0.22	
Urban	48.6 [44.8-52.4]	NA	1	675	NA	NA	1	
**Patient with any illness**								
**Country**								< 0.001
Liberia	0 [0-1.6]	NA	2	122	1 NA	0	1	
Nigeria	92.3 [69.9-100]	NA	1	13	NA	NA	1	
**Setting**								< 0.001
Community-based	92.3 [69.9-100]	NA	1	13	NA	NA	1	
Hospital-based	0 [0-1.6]	NA	2	122	1 NA	0	1	
**Patient with illnesses other than fever diseases**								
**Country**								0.032
Cameroon	0 [0-8]	NA	1	21	NA	NA	1	
Democratic Republic of the Congo	0 [0-0.9]	NA	1	181	NA	NA	1	
Nigeria	2.2 [1-3.8]	NA	1	458	NA	NA	1	
**UNSD Region**								0.011
Central Africa	0 [0-0.3]	NA	2	202	1 NA	0	0.537	

	Prevalence. % (95%CI)	95% Prediction interval	N Studies	N Participants	H (95%CI)	I² (95%CI)	P heterogeneity	P difference subtypes
West Africa	2.2 [1-3.8]	NA	1	458	NA	NA	1	
**LASV prevalence in rodents**								
**Current contact**								
**Mastomys natalensis**								
**Sampling**								0.128
Non probabilistic	4.1 [0.8-9.5]	[0-31.8]	7	3626	6.4 [5.3-7.8]	97.6 [96.4-98.3]	< 0.001	
Probabilistic	8.9 [4.3-14.7]	[0-92.6]	3	947	2 [1.1-3.7]	75.9 [20.7-92.7]	0.016	
**Country**								< 0.001
Guinea	12 [9.9-14.2]	[0-42.7]	3	2056	1.5 [1-2.9]	57.6 [0-87.9]	0.094	
Ivory Coast	4.9 [0-44.1]	NA	2	747	4.2 [2.3-7.4]	94.2 [81.8-98.2]	< 0.001	
Mali	3.2 [2.3-4.3]	NA	2	1182	1 NA	0	1	
Nigeria	4.3 [1.9-7.5]	[0-61.5]	3	588	1.5 [1-2.7]	53.5 [0-86.7]	0.116	
**Recrutment Setting**								0.254
Rural	7.3 [3.3-12.5]	[0-31.6]	5	2703	4.1 [3.1-5.6]	94.2 [89.3-96.9]	< 0.001	
Urban/rural	4.3 [1.9-7.5]	[0-61.5]	3	588	1.5 [1-2.7]	53.5 [0-86.7]	0.116	
**Sample types**								0,818
Organ tissue	3.4 [1.1-6.7]	[0-68.1]	3	1200	2 [1.1-3.6]	74 [13.4-92.2]	0,021	
Serum	3.2 [0-10.8]	[0-48.8]	5	2164	6.6 [5.2-8.3]	97.7 [96.3-98.5]	< 0.001	
**Mastomys species**								
**Country**								< 0.001
Mali	7.3 [4.3-11]	[0-40.1]	3	246	1 [1-1]	0 [0-0]	1	
Sierra Leone	16.5 [13.8-19.4]	[10.8-23.1]	4	693	1 [1-1.3]	0 [0-40]	0.858	
**Sample types**								0.004
liver	7.3 [2.5-14.1]	NA	1	82	NA	NA	1	
lung	7.3 [2.5-14.1]	NA	1	82	NA	NA	1	
Serum	7.3 [2.5-14.1]	NA	1	82	NA	NA	1	
Spleen sections	16.5 [13.7-19.5]	[2.6-38.6]	3	642	1 [1-1.8]	0 [0-69.4]	0.712	

	Prevalence. % (95%CI)	95% Prediction interval	N Studies	N Participants	H (95%CI)	I² (95%CI)	P heterogeneity	P difference subtypes
**Mastomys erythroleucus**								
**Sampling**								0.669
Non probabilistic	3.7 [0-15]	[0-80]	4	346	3.1 [2.1-4.7]	89.9 [76.9-95.5]	< 0.001	
Probabilistic	2.6 [0.3-6.5]	NA	1	116	NA	NA	1	
**Country**								0.838
Guinea	3.7 [0-20.5]	[0-100]	3	283	3.5 [2.2-5.6]	91.8 [79.2-96.8]	< 0.001	
Nigeria	3.2 [1-6.5]	NA	2	179	1 NA	0	0.426	
**Recrutment Setting**								0.01
Rural	0 [0-0.8]	NA	1	202	NA	NA	1	
Urban/rural	9.2 [0.3-25.5]	[0-100]	3	195	2.7 [1.6-4.5]	85.8 [58.5-95.1]	0.001	
**Past contact**								
**Mastomys natalensis**								
**Sampling**								0.668
Non probabilistic	12 [3.2-25.3]	NA	2	1107	5.8 [3.6-9.4]	97.1 [92.4-98.9]	< 0.001	
Probabilistic	14.7 [10.7-19.1]	NA	1	273	NA	NA	1	
**Country**								0.001
Mali	6.8 [4.8-9.2]	NA	1	511	NA	NA	1	
Nigeria	14.7 [10.7-19.1]	NA	1	273	NA	NA	1	
**Recrutment Setting**								0.001
Rural	6.8 [4.8-9.2]	NA	1	511	NA	NA	1	
Urban/rural	14.7 [10.7-19.1]	NA	1	273	NA	NA	1	

LASV: Lassa virus; CI: confidence interval; NA: not applicable; UNSD: United Nations Statistics Division.
