# Supplementary material for: Systematic review and meta-analysis of the epidemiology of Lassa virus in humans, rodents and other mammals in sub-Saharan Africa
Source: PLoS Negl Trop Dis. 2020 Aug 26;14(8):e0008589. doi: 10.1371/journal.pntd.0008589 (PMC7478710; doi:10.1371/journal.pntd.0008589)
Supplement: S9 Table — (PDF) [file pntd.0008589.s009.pdf]

S9 Table: Univariable and multivariable meta-regression analysis on the human case fatality rate and prevalence of Lassa virus in humans. rodents. and other animals.

	**Bivariate Model**			**Multivariate Model**		
	**P-Value**	**P-Value Global**	**OR(95% CI)**	**P-Value**	**OR [95% CI]**	**R2**
**Case fatality rate in humans**						
**Current contact**						
**Positive among LASV suspected cases**						**32.56%**
**Study Design**		0.962				
• Cohort (Baseline data)			1			
• Community outbreak	0.6		1.18 [0.63-2.21]			
• Cross-sectional	0.653		1.13 [0.66-1.96]			
• Hospital outbreak	0.672		1.15 [0.61-2.17]			
**Timing of data collection**		**0.002**				
• Prospetively			1		1	
• Retrospectively	**0.002**		1.32 [1.11-1.57]	**0.002**	1.32 [1.11-1.57]	
**Country**		0.816				
• Liberia			1			
• Nigeria	0.847		1.04 [0.7-1.53]			
• Sierra Leone	0.653		1.1 [0.73-1.64]			
**Recrutment setting**		< **0.001**				
• Rural			1			
• Urban	**0.004**		0.65 [0.48-0.87]			
• Urban/rural	0.953		0.99 [0.73-1.35]			
**Setting**		0.513				
• Community-based			1			
• Hospital-based	0.357		0.79 [0.48-1.3]			

	Bivariate Model			Multivariate Model		
	**P-Value**	**P-Value Global**	**OR(95% CI)**	**P-Value**	**OR [95% CI]**	**R2**
• Hospital/community based	0.248		0.71 [0.4-1.27]			
**Hospitalization**		0.808				
• Hospitalized			1			
• Hospitalized/ambulatory	0.808		0.93 [0.54-1.62]			
**LASV prevalence in humans**						
**Current contact**						
**Febrile patients**						
**Study Design**		0.711				
• Case-control			1			
• Cross-sectional	0.616		0.93 [0.71-1.22]			
• Hospital outbreak	0.823		1.05 [0.71-1.54]			
**Timing of data collection**		0.614				
• Prospetively			1			
• Retrospectively	0.614		1.06 [0.85-1.33]			
**Country**		0.274				
• Ghana			1			
• Guinea	0.998		1 [0.73-1.37]			
• Liberia	**0.112**		1.3 [0.94-1.78]			
• Mali	0.803		1.04 [0.76-1.43]			
• Nigeria	0.25		1.17 [0.9-1.52]			
• Sierra Leone	**0.081**		1.33 [0.97-1.82]			
**Recrutment setting**		0.264				
• Rural			1			
• Urban	0.264		1.17 [0.89-1.53]			
**Setting**		**0.084**				

	**Bivariate Model**			**Multivariate Model**		
	**P-Value**	**P-Value Global**	**OR(95% CI)**	**P-Value**	**OR [95% CI]**	**R2**
• Community-based			1			
• Hospital-based	0.244		1.16 [0.9-1.49]			
• Hospital/community based	**0.027**		1.48 [1.05-2.1]			
**Hospitalization**		**0.137**				
• Ambulatory			1			
• Hospitalized	**0.137**		1.22 [0.94-1.6]			
**LASV suspected cases**						**0.00%**
**Study Design**		**0.188**				
• Case-control			1		1	
• Community outbreak	**0.106**		0.75 [0.53-1.06]			
• Cross-sectional	**0.044**		0.76 [0.58-0.99]	**0.016**	0.72 [0.55-0.94]	
• Hospital outbreak	**0.035**		0.71 [0.52-0.98]	**0.033**	0.71 [0.52-0.97]	
**Timing of data collection**		0.268				
• Prospetively			1			
• Retrospectively	0.268		0.94 [0.83-1.05]			
**Country**		**0.042**				
• Liberia			1			
• Nigeria	0.334		1.08 [0.93-1.25]			
• Sierra Leone	**0.017**		1.22 [1.04-1.44]			
**Recrutment setting**		0.627				
• Rural			1			
• Urban	0.351		0.74 [0.4-1.39]			
• Urban/rural	0.556		0.88 [0.58-1.34]			
**Setting**		**0.01**				
• Hospital-based			1		1	

	Bivariate Model			Multivariate Model		
	**P-Value**	**P-Value Global**	**OR(95% CI)**	**P-Value**	**OR [95% CI]**	**R2**
• Hospital/community based	**0.01**		1.18 [1.04-1.34]	**0.007**	1.2 [1.05-1.38]	
**Hospitalization**		0.902				
• Ambulatory			1			
• Hospitalized	0.989		1 [0.79-1.26]			
• Hospitalized/ambulatory	0.77		1.04 [0.79-1.39]			
**Recent contact**						
**Apparently healthy individuals**						**60.38%**
**Study Design**		0.958				
• Cross-sectional			1			
• Hospital outbreak	0.958		1.01 [0.62-1.66]			
**Timing of data collection**		0.927				
• Prospetively			1			
• Retrospectively	0.927		0.98 [0.7-1.38]			
**Country**		**0.01**				
• Mali			1		1	
• Nigeria	0.491		1.15 [0.77-1.71]	0.491	1.15 [0.77-1.71]	
• Sierra Leone	**0.004**		1.93 [1.23-3.02]	**0.004**	1.93 [1.23-3.02]	
**Setting**		0.947				
• Community-based			1			
• Hospital-based	0.922		1.03 [0.53-2.02]			
• Hospital/community based	0.741		1.09 [0.66-1.79]			
**Febrile patients**						
**Study Design**		0.298				
• Case-control			1			
• Cross-sectional	**0.155**		0.69 [0.42-1.15]			

	Bivariate Model			Multivariate Model		
	**P-Value**	**P-Value Global**	**OR(95% CI)**	**P-Value**	**OR [95% CI]**	**R2**
• Hospital outbreak	0.792		0.92 [0.51-1.68]			
**Country**		0.619				
• Nigeria			1			
• Sierra Leone	0.619		0.85 [0.45-1.6]			
**Recrutment setting**		0.408				
• Rural			1			
• Urban	0.408		0.74 [0.36-1.52]			
**LASV suspected cases**						
**Study Design**		0.551				
• Case-control			1			
• Cross-sectional	0.551		0.79 [0.37-1.7]			
**Timing of data collection**		0.569				
• Prospetively			1			
• Retrospectively	0.569		0.84 [0.45-1.54]			
**Country**		0.387				
• Liberia			1			
• Nigeria	0.913		1.04 [0.53-2.04]			
• Sierra Leone	0.293		1.41 [0.74-2.66]			
**Setting**		**0.175**				
• Hospital-based			1			
• Hospital/community based	**0.175**		1.38 [0.87-2.2]			
**Hospitalization**		0.731				
• Ambulatory			1			
• Hospitalized	0.731		0.85 [0.34-2.13]			
**Past contact**						

	Bivariate Model			Multivariate Model		
	**P-Value**	**P-Value Global**	**OR(95% CI)**	**P-Value**	**OR [95% CI]**	**R2**
**Apparently healthy individuals**						**75.98%**
**Study Design**		0.274				
• Case-control			1			
• Cross-sectional	**0.143**		0.72 [0.46-1.12]			
• Hospital outbreak	0.648		0.86 [0.44-1.67]			
**Sampling**		0.884				
• Non probabilistic			1			
• Probabilistic	0.884		0.99 [0.84-1.16]			
**Country**		< **0.001**				
• Benin			1			
• Burki Faso	0.884		0.97 [0.63-1.49]			
• Cameroon	0.604		0.91 [0.64-1.3]			
• Central African Republic	0.611		0.92 [0.65-1.29]			
• Chad	0.6		0.9 [0.61-1.33]			
• Democratic Republic of the Congo	0.733		0.93 [0.63-1.39]			
• Equatorial Guinea	0.666		0.92 [0.63-1.35]			
• Ethiopia	0.681		0.92 [0.62-1.36]			
• Gabon	0.612		0.91 [0.64-1.3]			
• Ghana	0.609		1.11 [0.75-1.62]			
• Guinea	**0.082**		1.36 [0.96-1.91]			
• Ivory Coast	0.972		0.99 [0.63-1.56]			
• Kenya	0.625		0.91 [0.64-1.31]			
• Liberia	0.83		1.04 [0.73-1.47]			
• Madagascar	0.594		0.9 [0.61-1.32]			
• Mali	**0.076**		1.39 [0.97-2.01]			

	**Bivariate Model**			**Multivariate Model**		
	**P-Value**	**P-Value Global**	**OR(95% CI)**	**P-Value**	**OR [95% CI]**	**R2**
• Niger	0.816		0.95 [0.63-1.44]			
• Nigeria	0.407		1.15 [0.82-1.62]			
• Republic of the Congo	0.754		0.94 [0.64-1.38]			
• Sierra Leone	**0.001**		1.74 [1.24-2.45]			
• Tanzania	0.84		0.96 [0.63-1.46]			
• Uganda	0.966		1.01 [0.7-1.44]			
• Zimbabwe	0.699		0.93 [0.63-1.36]			
**UNSD Region**		**< 0.001**				
• Central Africa			1		1	
• Eastern Africa	0.582		1.03 [0.93-1.14]	0.979	1 [0.89-1.12]	
• West Africa	**0**		1.37 [1.27-1.48]	**0**	1.3 [1.2-1.41]	
**Recrutment setting**		0.635				
• Rural			1			
• Urban	0.732		0.95 [0.72-1.26]			
• Urban/rural	0.39		1.12 [0.86-1.47]			
**Setting**		**0.002**				
• Community-based			1		1	
• Hospital-based	0.918		1.02 [0.73-1.41]	0.858	1.02 [0.84-1.23]	
• Hospital/community based	**0**		1.4 [1.16-1.69]	**0**	1.26 [1.14-1.4]	
**Apparently healthy individuals. Patient with any illness**						
**Study Design**		**0.107**				
• Case-control			1			
• Cross-sectional	**0.107**		0.92 [0.83-1.02]			
**Febrile patients**						**54.14%**
**Study Design**		0.902				

	Bivariate Model			Multivariate Model		
	**P-Value**	**P-Value Global**	**OR(95% CI)**	**P-Value**	**OR [95% CI]**	**R2**
• Community outbreak			1			
• Cross-sectional	0.699		1.06 [0.78-1.45]			
• Hospital outbreak	0.686		1.11 [0.68-1.8]			
**Sampling**		**0.18**				
• Non probabilistic			1		1	
• Probabilistic	**0.18**		0.81 [0.6-1.1]	**0.05**	0.81 [0.6-1.01]	
**Timing of data collection**		0.782				
• Prospetively			1			
• Retrospectively	0.782		0.95 [0.64-1.4]			
**Country**		**0.001**				
• Central African Republic			1			
• Guinea	**0**		1.67 [1.27-2.19]			
• Liberia	**0.037**		1.4 [1.02-1.92]			
• Nigeria	**0.045**		1.33 [1.01-1.76]			
• Sierra Leone	**0.006**		1.55 [1.14-2.13]			
**UNSD Region**		**0.042**				
• Central Africa			1		1	
• West Africa	**0.042**		1.5 [1.01-2.21]	**0.006**	1.55 [1.13-2.1]	
**Recrutment setting**		0.901				
• Rural			1			
• Urban	0.875		1.02 [0.77-1.36]			
• Urban/rural	0.727		0.95 [0.72-1.26]			
**Setting**		0.647				
• Community-based			1			
• Hospital-based	0.356		0.9 [0.73-1.12]			

	Bivariate Model			Multivariate Model		
	**P-Value**	**P-Value Global**	**OR(95% CI)**	**P-Value**	**OR [95% CI]**	**R2**
• Hospital/community based	0.563		0.92 [0.7-1.21]			
**Hospitalization**		0.351				
• Ambulatory			1			
• Hospitalized	**0.154**		1.13 [0.95-1.35]			
• Hospitalized/ambulatory	0.795		1.04 [0.77-1.4]			
**Healthcare workers**						
**Study Design**		0.248				
• Community outbreak			1			
• Cross-sectional	**0.194**		1.21 [0.91-1.61]			
• Hospital outbreak	0.51		1.11 [0.81-1.51]			
**Timing of data collection**		0.534				
• Prospetively			1			
• Retrospectively	0.534		0.96 [0.83-1.1]			
**Country**		**0.162**				
• Guinea			1			
• Liberia	0.528		0.94 [0.77-1.14]			
• Nigeria	0.238		0.89 [0.73-1.08]			
• Sierra Leone	0.692		1.05 [0.84-1.31]			
**Recrutment setting**		0.436				
• Rural			1			
• Urban	0.236		1.16 [0.91-1.49]			
• Urban/rural	0.915		0.99 [0.79-1.23]			
**Setting**		0.239				
• Community-based			1			
• Hospital-based	**0.101**		1.18 [0.97-1.44]			

	Bivariate Model			Multivariate Model		
	**P-Value**	**P-Value Global**	**OR(95% CI)**	**P-Value**	**OR [95% CI]**	**R2**
• Hospital/community based	0.285		1.12 [0.91-1.37]			
**Hospitalization**		0.944				
• Ambulatory			1			
• Hospitalized/ambulatory	0.944		0.99 [0.79-1.24]			
**High risk individuals**						
**UNSD Region**		**0.112**				
• Eastern Africa			1			
• West Africa	**0.112**		1.42 [0.92-2.18]			
**LASV positive case contact**						
**Study Design**		0.938				
• Community outbreak			1			
• Hospital outbreak	0.938		0.99 [0.81-1.22]			
**Country**		0.286				
• Liberia			1			
• Nigeria	0.286		0.91 [0.76-1.08]			
**LASV suspected cases**						**99.09%**
**Timing of data collection**		0.282				
• Prospetively			1			
• Retrospectively	0.282		0.79 [0.52-1.21]			
**Country**		0.271				
• Guinea			1			
• Nigeria	0.607		0.87 [0.52-1.46]			
• Sierra Leone	0.423		1.23 [0.74-2.06]			
**Recrutment setting**		**< 0.001**				
• Rural			1		1	

	Bivariate Model			Multivariate Model		
	**P-Value**	**P-Value Global**	**OR(95% CI)**	**P-Value**	**OR [95% CI]**	**R2**
• Urban	**< 0.001**		1.41 [1.31-1.52]	**< 0.001**	1.41 [1.31-1.52]	
**Patient with any illness**						**100.00%**
**Country**		**< 0.001**				
• Liberia			1			
• Nigeria	**< 0.001**		3.25 [2.45-4.3]			
**Setting**		**< 0.001**				
• Community-based			1		1	
• Hospital-based	**< 0.001**		0.31 [0.23-0.41]	**< 0.001**	0.31 [0.23-0.41]	
**Patient with illnesses other than fever diseases**						**100.00%**
**UNSD Region**		**0.011**				
• Central Africa			1		1	
• West Africa	**0.011**		1.11 [1.03-1.21]	**0.011**	1.11 [1.03-1.21]	
**LASV prevalence in rodents**						
**Current contact**						
**Mastomys species**						**100.00%**
**Country**		**< 0.001**				
• Mali			1		1	
• Sierra Leone	**< 0.001**		1.15 [1.07-1.23]	**< 0.001**	1.15 [1.07-1.23]	
**Sample types**		**0.004**				
• liver			1			
• lung	1		1 [0.86-1.17]			
• Serum	1		1 [0.86-1.17]			
• Spleen sections	**0.019**		1.15 [1.02-1.29]			
**Mastomys talensis**						**82.06%**
**Sampling**		**0.191**				

	Bivariate Model			Multivariate Model		
	**P-Value**	**P-Value Global**	**OR(95% CI)**	**P-Value**	**OR [95% CI]**	**R2**
• Non probabilistic			1			
• Probabilistic	**0.191**		1.14 [0.94-1.39]			
**Country**		**< 0.001**				
• Guinea			1		1	
• Ivory Coast	**< 0.001**		0.76 [0.69-0.85]	**< 0.001**	0.76 [0.69-0.85]	
• Mali	**< 0.001**		0.84 [0.77-0.92]	**< 0.001**	0.84 [0.77-0.92]	
• Nigeria	**0.003**		0.87 [0.8-0.95]	**0.003**	0.87 [0.8-0.95]	
**Recrutment setting**		0.271				
• Rural			1			
• Urban/rural	0.271		0.93 [0.81-1.06]			
**Sample types**		0.245				
• Broncho-alveolar			1			
• Organ tissue	**0.119**		0.72 [0.48-1.09]			
• Serum	**0.102**		0.73 [0.5-1.07]			
**Mastomys erythroleucus**						
**Sampling**		0.826				
• Non probabilistic			1			
• Probabilistic	0.826		0.96 [0.63-1.44]			
• Country		0.979				
• Guinea			1			
• Nigeria	0.979		1 [0.73-1.36]			
**Recrutment setting**		**0.152**				
• Rural			1			
• Urban/rural	**0.152**		1.34 [0.9-1.99]			
**Past contact**						

	Bivariate Model			Multivariate Model		
	**P-Value**	**P-Value Global**	**OR(95% CI)**	**P-Value**	**OR [95% CI]**	**R2**
**Mastomys talensis**						
**Sampling**		0.796				
• Non probabilistic			1			
• Probabilistic	0.796		1.04 [0.77-1.41]			
