# Supplementary material for: Systematic review and meta-analysis of the epidemiology of Lassa virus in humans, rodents and other mammals in sub-Saharan Africa
Source: PLoS Negl Trop Dis. 2020 Aug 26;14(8):e0008589. doi: 10.1371/journal.pntd.0008589 (PMC7478710; doi:10.1371/journal.pntd.0008589)
Supplement: S1 Fig — (PDF) [file pntd.0008589.s010.pdf]

S1 Fig: Prevalence of Lassa virus infections in humans in sub-Saharan Africa

Study	Positive	Total	Prevalence (%)	95% CI	Weight
**Past contact _ Apparently healthy individuals (48 Studies)**					
Arnold, 1977 − Nigeria	25	434	5.76	[3.76; 8.39]	0.7%
Arnold, 1977 − Nigeria	18	336	5.36	[3.21; 8.33]	0.7%
Blackburn, 1982 − Zimbabwe	1	486	0.21	[0.01; 1.14]	0.7%
Branco, 2011 − Sierra Leone	96	114	84.21	[76.20; 90.37]	0.7%
Clements, 2019 − Uganda	2	1744	0.11	[0.01; 0.41]	0.7%
Emmerich, 2006 − Guinea, Ghana	59	643	9.18	[7.06; 11.68]	0.7%
Emmerich, 2006 − Guinea, Ghana	53	643	8.24	[6.24; 10.64]	0.7%
Emmerich, 2008 − Guinea, Ivory Coast, Ghana, Benin, Nigeria	94	960	9.79	[7.99; 11.85]	0.7%
Georges, 1985 − Central African Republic	5	715	0.70	[0.23; 1.62]	0.7%
Gonzalez, 1989 − Cameroon	2	1152	0.17	[0.02; 0.63]	0.7%
Gonzalez, 1989 − Central African Republic	0	327	0.00	[0.00; 1.12]	0.7%
Gonzalez, 1989 − Chad	0	334	0.00	[0.00; 1.10]	0.7%
Gonzalez, 1989 − Equatorial Guinea	1	688	0.15	[0.00; 0.81]	0.7%
Gonzalez, 1989 − Gabon	0	1841	0.00	[0.00; 0.20]	0.7%
Gonzalez, 1989 − Republic of the Congo	3	728	0.41	[0.09; 1.20]	0.7%
Helmick, 1986 − Sierra Leone	360	1024	35.16	[32.23; 38.17]	0.7%
Henderson, 1972 − Benin	0	14	0.00	[0.00; 23.16]	0.5%
Henderson, 1972 − Burkina Faso	0	25	0.00	[0.00; 13.72]	0.6%
Henderson, 1972 − Democratic Republic of the Congo	0	67	0.00	[0.00; 5.36]	0.7%
Henderson, 1972 − Ethiopia	0	108	0.00	[0.00; 3.36]	0.7%
Henderson, 1972 − Gabon	0	10	0.00	[0.00; 30.85]	0.5%
Henderson, 1972 − Guinea	4	17	23.53	[6.81; 49.90]	0.5%
Henderson, 1972 − Ivory Coast	0	16	0.00	[0.00; 20.59]	0.5%
Henderson, 1972 − Kenya	0	30	0.00	[0.00; 11.57]	0.6%
Henderson, 1972 − Liberia	0	62	0.00	[0.00; 5.78]	0.6%
Henderson, 1972 − Mali	0	17	0.00	[0.00; 19.51]	0.5%
Henderson, 1972 − Niger	0	37	0.00	[0.00; 9.49]	0.6%
Henderson, 1972 − Nigeria	1	257	0.39	[0.01; 2.15]	0.7%
Henderson, 1972 − Tanzania	0	32	0.00	[0.00; 10.89]	0.6%
Ivanoff, 1982 − Gabon	0	253	0.00	[0.00; 1.45]	0.7%
Johnson, 1983 − Kenya	0	1899	0.00	[0.00; 0.19]	0.7%
Johnson, 1993 − Central African Republic	8	4295	0.19	[0.08; 0.37]	0.7%
Keenlyside, 1983 − Sierra Leone	248	953	26.02	[23.26; 28.93]	0.7%
Kernéis, 2009 − Guinea	112	977	11.46	[9.53; 13.63]	0.7%
Lukashevich, 1993 − Guinea	738	3126	23.61	[22.13; 25.14]	0.7%
Mathiot, 1989 − Madagascar	0	381	0.00	[0.00; 0.96]	0.7%
McCormick, 1987 − Sierra Leone	1172	5213	22.48	[21.36; 23.64]	0.7%
Meunier, 1987 − Central African Republic	1	836	0.12	[0.00; 0.66]	0.7%
Monath, 1973 − Liberia	4	133	3.01	[0.83; 7.52]	0.7%
Nakounné, 2000 − Central African Republic	0	1743	0.00	[0.00; 0.21]	0.7%
Nimo−Paintsil, 2019 − Ghana	34	657	5.18	[3.61; 7.16]	0.7%
Omilabu, 2005 − Nigeria	2	17	11.76	[1.46; 36.44]	0.5%
Paix, 1988 − Cameroon	0	375	0.00	[0.00; 0.98]	0.7%
Rodhain, 1989 − Uganda	8	132	6.06	[2.65; 11.59]	0.7%
Sogoba, 2016 − Mali	199	600	33.17	[29.41; 37.09]	0.7%
Ter Meulen, 1994 − Guinea	159	983	16.17	[13.93; 18.63]	0.7%
Tomori, 1988 − Nigeria	357	1677	21.29	[19.35; 23.33]	0.7%
Yalley−Ogunro, 1984 − Liberia	98	1848	5.30	[4.33; 6.42]	0.7%
**Random effect meta−analysis**		**38959**	**3.82**	**[1.51; 6.95]**	**31.9%**
Heterogeneity: $I^2 = 99.4\%$ [99.3%; 99.4%], $\tau^2 = 0.0515$, $p = 0$					
**Past contact _ Apparently healthy individuals, Patient with any illness (4 Studies)**					
Gonzalez, 1983 − Central African Republic	8	1898	0.42	[0.18; 0.83]	0.7%
Johnson, 1983 − Kenya	0	741	0.00	[0.00; 0.50]	0.7%
McCarthy, 1996 − Sudan	6	300	2.00	[0.74; 4.30]	0.7%
Van der Waals, 1986 − Liberia	3	225	1.33	[0.28; 3.85]	0.7%
**Random effect meta−analysis**		**3164**	**0.58**	**[0.03; 1.66]**	**2.8%**
Heterogeneity: $I^2 = 83.1\%$ [56.9%; 93.4%], $\tau^2 = 0.0020$, $p = 0.0005$					
**Past contact _ Febrile patients (10 Studies)**					
Boiro, 1987 − Guinea	3	22	13.64	[2.91; 34.91]	0.6%
Bukbuk, 2014 − Nigeria	22	297	7.41	[4.70; 11.00]	0.7%
Frame, 1984 − Liberia	30	176	17.05	[11.81; 23.43]	0.7%
Klempa, 2013 − Guinea	102	253	40.32	[34.22; 46.64]	0.7%
Klempa, 2013 − Guinea	102	253	40.32	[34.22; 46.64]	0.7%
Lukashevich, 1993 − Guinea	10	46	21.74	[10.95; 36.36]	0.6%
Nakounné, 2000 − Central African Republic	0	29	0.00	[0.00; 11.94]	0.6%
Omilabu, 2005 − Nigeria	7	31	22.58	[9.59; 41.10]	0.6%
Schoepp, 2014 − Sierra Leone	61	237	25.74	[20.30; 31.80]	0.7%
Smith, 1979 − Nigeria	15	97	15.46	[8.92; 24.22]	0.7%
**Random effect meta−analysis**		**1441**	**19.12**	**[10.75; 29.11]**	**6.5%**
Heterogeneity: $I^2 = 94.3\%$ [91.5%; 96.2%], $\tau^2 = 0.0306$, $p < 0.0001$					
**Past contact _ Healthcare workers (17 Studies)**					
Bajani, 1997 − Nigeria	68	552	12.32	[9.69; 15.35]	0.7%
Bowen, 1975 − Nigeria	2	261	0.77	[0.09; 2.74]	0.7%
Fabiyi, 1975 − Sierra Leone	12	170	7.06	[3.70; 12.01]	0.7%
Fabiyi, 1979 − Nigeria	53	1077	4.92	[3.71; 6.39]	0.7%
Fisher−Hoch, 1995 − Nigeria	7	415	1.69	[0.68; 3.44]	0.7%
Frame, 1979 − Liberia	72	909	7.92	[6.25; 9.87]	0.7%
Frame, 1984 − Liberia	95	637	14.91	[12.24; 17.92]	0.7%
Helmick, 1986 − Sierra Leone	108	496	21.77	[18.22; 25.67]	0.7%
Knobloch, 1982 − Liberia	6	104	5.77	[2.15; 12.13]	0.7%
Knobloch, 1982 − Liberia	6	104	5.77	[2.15; 12.13]	0.7%
Lukashevich, 1993 − Guinea	45	406	11.08	[8.20; 14.55]	0.7%
Monath, 1973 − Liberia	3	120	2.50	[0.52; 7.13]	0.7%
Omilabu, 2005 − Nigeria	4	12	33.33	[9.92; 65.11]	0.5%
Smith, 1979 − Nigeria	53	1070	4.95	[3.73; 6.43]	0.7%
Troup, 1970 − Nigeria	0	22	0.00	[0.00; 15.44]	0.6%
**Random effect meta−analysis**		**6355**	**6.84**	**[4.16; 10.07]**	**10.0%**
Heterogeneity: $I^2 = 94.4\%$ [92.3%; 96.0%], $\tau^2 = 0.0105$, $p < 0.0001$					
**Past contact _ High risk individuals (3 Studies)**					
Akoua−Koffi, 2006 − Ivory Coast	42	161	26.09	[19.49; 33.58]	0.7%
Henderson, 1972 − Nigeria	23	281	8.19	[5.26; 12.03]	0.7%
Johnson, 1982 − Kenya	0	58	0.00	[0.00; 6.16]	0.6%
**Random effect meta−analysis**		**500**	**8.58**	**[0.23; 25.40]**	**2.0%**
Heterogeneity: $I^2 = 95.6\%$ [90.3%; 98.0%], $\tau^2 = 0.0381$, $p < 0.0001$					
**Past contact _ LASV positive case contact (4 Studies)**					
Carey, 1972 − Nigeria	4	140	2.86	[0.78; 7.15]	0.7%
Carey, 1972 − Nigeria	0	26	0.00	[0.00; 13.23]	0.6%
Monath, 1973 − Liberia	6	76	7.89	[2.95; 16.40]	0.7%
Troup, 1970 − Nigeria	4	53	7.55	[2.09; 18.21]	0.6%
**Random effect meta−analysis**		**295**	**4.15**	**[1.24; 8.32]**	**2.6%**
Heterogeneity: $I^2 = 44\%$ [0.0%; 81.3%], $\tau^2 = 0.0030$, $p = 0.1474$					
**Past contact _ LASV suspected cases (5 Studies)**					
Bausch, 2001 − Guinea	48	311	15.43	[11.60; 19.94]	0.7%
Ehichioya , 2012 − Nigeria	8	451	1.77	[0.77; 3.47]	0.7%
Fabiyi, 1979 − Nigeria	15	97	15.46	[8.92; 24.22]	0.7%
Keane, 1977 − Sierra Leone	51	264	19.32	[14.74; 24.60]	0.7%
O'Hearn, 2016 − Sierra Leone	328	675	48.59	[44.76; 52.44]	0.7%
**Random effect meta−analysis**		**1798**	**17.72**	**[3.05; 40.59]**	**3.5%**
Heterogeneity: $I^2 = 99.1\%$ [98.8%; 99.4%], $\tau^2 = 0.0836$, $p < 0.0001$					
**Past contact _ Patient with any illness (5 Studies)**					
Henderson, 1972 − Nigeria	12	13	92.31	[63.97; 99.81]	0.5%
Knobloch, 1982 − Liberia	0	61	0.00	[0.00; 5.87]	0.6%
Knobloch, 1982 − Liberia	0	61	0.00	[0.00; 5.87]	0.6%
**Random effect meta−analysis**		**135**	**16.94**	**[0.00; 67.98]**	**1.8%**
Heterogeneity: $I^2 = 97\%$ [94.1%; 98.5%], $\tau^2 = 0.2054$, $p < 0.0001$					
**Past contact _ Patient with illnesses other than fever diseases (3 Studies)**					
Henderson, 1972 − Nigeria	10	458	2.18	[1.05; 3.98]	0.7%
Nakounné, 2000 − Cameroon	0	21	0.00	[0.00; 16.11]	0.6%
Nakounné, 2000 − Democratic Republic of the Congo	0	181	0.00	[0.00; 2.02]	0.7%
**Random effect meta−analysis**		**660**	**0.31**	**[0.00; 2.84]**	**1.9%**
Heterogeneity: $I^2 = 70.8\%$ [0.6%; 91.4%], $\tau^2 = 0.0041$, $p = 0.0325$					
**Recent contact _ Apparently healthy individuals (7 Studies)**					
Branco, 2011 − Sierra Leone	52	114	45.61	[36.26; 55.21]	0.7%
Emmerich, 2008 − Guinea, Ivory Coast, Ghana, Benin, Nigeria	88	960	9.17	[7.42; 11.17]	0.7%
Fisher−Hoch, 1995 − Nigeria	6	415	1.45	[0.53; 3.12]	0.7%
Omilabu, 2005 − Nigeria	2	17	11.76	[1.46; 36.44]	0.5%
Sogoba, 2016 − Mali	4	600	0.67	[0.18; 1.70]	0.7%
Ter Meulen, 1998 − Guinea, Liberia	205	673	30.46	[27.00; 34.09]	0.7%
Ter Meulen, 1998 − Guinea, Liberia	87	673	12.93	[10.49; 15.70]	0.7%
**Random effect meta−analysis**		**3452**	**12.57**	**[3.98; 24.78]**	**4.7%**
Heterogeneity: $I^2 = 98.7\%$ [98.3%; 99.1%], $\tau^2 = 0.0420$, $p < 0.0001$					
**Recent contact _ Febrile patients (4 Studies)**					
McCormick, 1987 − Sierra Leone	441	1087	40.57	[37.63; 43.56]	0.7%
Omilabu, 2005 − Nigeria	10	31	32.26	[16.68; 51.37]	0.6%
Schoepp, 2014 − Sierra Leone	7	237	2.95	[1.20; 5.99]	0.7%
Webb, 1986 − Sierra Leone	51	245	20.82	[15.91; 26.44]	0.7%
**Random effect meta−analysis**		**1600**	**21.55**	**[4.59; 46.03]**	**2.7%**
Heterogeneity: $I^2 = 98.6\%$ [97.8%; 99.1%], $\tau^2 = 0.0678$, $p < 0.0001$					
**Recent contact _ Healthcare workers (2 Studies)**					
Bajani, 1997 − Nigeria	6	552	1.09	[0.40; 2.35]	0.7%
Omilabu, 2005 − Nigeria	0	12	0.00	[0.00; 26.46]	0.5%
**Random effect meta−analysis**		**564**	**0.02**	**[0.00; 0.69]**	**1.2%**
Heterogeneity: $I^2 = 0\%$, $\tau^2 = 0$, $p = 0.8231$					
**Recent contact _ LASV suspected cases (5 Studies)**					
Branco, 2011 − Sierra Leone	206	546	37.73	[33.65; 41.94]	0.7%
Dahmane, 2014 − Sierra Leone	22	84	26.19	[17.20; 36.93]	0.7%
Demby, 1994 − Sierra Leone	29	61	47.54	[34.60; 60.73]	0.6%
Ehichioya , 2012 − Nigeria	0	451	0.00	[0.00; 0.81]	0.7%
Ibekwe, 2012 − Nigeria	29	72	40.28	[28.88; 52.50]	0.7%
Panning, 2010 − Liberia	17	184	9.24	[5.47; 14.38]	0.7%
**Random effect meta−analysis**		**1398**	**22.42**	**[4.40; 48.61]**	**4.0%**
Heterogeneity: $I^2 = 98.9\%$ [98.5%; 99.2%], $\tau^2 = 0.1165$, $p < 0.0001$					
**Current contact _ Apparently healthy individuals (2 Studies)**					
Branco, 2011 − Sierra Leone	0	114	0.00	[0.00; 3.18]	0.7%
Omilabu, 2005 − Nigeria	1	17	5.88	[0.15; 28.69]	0.5%
**Random effect meta−analysis**		**131**	**0.74**	**[0.00; 12.23]**	**1.2%**
Heterogeneity: $I^2 = 71.9\%$ [0.0%; 93.7%], $\tau^2 = 0.0211$, $p = 0.0591$					
**Current contact _ Blood donors (1 Study)**					
Demby, 1994 − Sierra Leone	0	127	0.00	[0.00; 2.86]	0.7%
**Random effect meta−analysis**		**127**	**0.00**	**[0.00; 1.35]**	**0.7%**
Heterogeneity: not applicable					
**Current contact _ Febrile patients (9 Studies)**					
Akhuemokhan, 2017 − Nigeria	13	243	5.35	[2.88; 8.97]	0.7%
Baumann, 2019 − Mali	2	489	0.41	[0.05; 1.47]	0.7%
Bonney, 2013 − Ghana	0	258	0.00	[0.00; 1.42]	0.7%
Ehichioya , 2012 − Nigeria	2	451	0.44	[0.05; 1.59]	0.7%
Frame, 1984 − Liberia	14	176	7.95	[4.42; 12.99]	0.7%
Klempa, 2013 − Guinea	0	253	0.00	[0.00; 1.45]	0.7%
Omilabu, 2005 − Nigeria	2	31	6.45	[0.79; 21.42]	0.6%
Ter Meulen, 1998 − Guinea, Liberia	9	55	16.36	[7.77; 28.80]	0.6%
Webb, 1986 − Sierra Leone	23	245	9.39	[6.04; 13.75]	0.7%
**Random effect meta−analysis**		**2201**	**3.03**	**[0.67; 6.72]**	**6.1%**
Heterogeneity: $I^2 = 93.1\%$ [89.1%; 95.7%], $\tau^2 = 0.0145$, $p < 0.0001$					
**Current contact _ Healthcare workers (2 Studies)**					
Dan−Nwafor, 2019 − Nigeria	5	16	31.25	[11.02; 58.66]	0.5%
Omilabu, 2005 − Nigeria	0	12	0.00	[0.00; 26.46]	0.5%
**Random effect meta−analysis**		**28**	**11.15**	**[0.00; 55.04]**	**1.0%**
Heterogeneity: $I^2 = 83.7\%$ [32.4%; 96.1%], $\tau^2 = 0.0901$, $p = 0.0133$					
**Current contact _ LASV suspected cases (19 Studies)**					
Asogun, 2012 − Nigeria	198	1650	12.00	[10.47; 13.67]	0.7%
Babalola, 2019 − Nigeria	112	1263	8.87	[7.36; 10.57]	0.7%
Branco, 2011 − Sierra Leone	131	546	23.99	[20.47; 27.80]	0.7%
Cummins, 1990 − Sierra Leone	49	69	71.01	[58.84; 81.31]	0.7%
Dahmane, 2014 − Sierra Leone	26	84	30.95	[21.31; 41.98]	0.7%
Dan−Nwafor, 2019 − Nigeria	554	2323	23.85	[22.13; 25.64]	0.7%
Demby, 1994 − Sierra Leone	29	61	47.54	[34.60; 60.73]	0.6%
Ehichioya , 2012 − Nigeria	25	60	41.67	[29.07; 55.12]	0.6%
Fisher−Hoch, 1988 − Sierra Leone	32	70	45.71	[33.74; 58.06]	0.7%
Hamblion, 2018 − Liberia	14	39	35.90	[21.20; 52.82]	0.6%
Ibekwe, 2012 − Nigeria	37	72	51.39	[39.31; 63.35]	0.7%
Ipadeola, 2020 − Nigeria	1354	7517	18.01	[17.15; 18.90]	0.7%
Isere, 2018 − Nigeria	19	90	21.11	[13.21; 30.99]	0.7%
Jahrling, 1985 − Liberia	63	1285	4.90	[3.79; 6.23]	0.7%
Maigari, 2018 − Nigeria	27	125	21.60	[14.74; 29.85]	0.7%
Oloniniyi, 2018 − Nigeria	29	123	23.58	[16.39; 32.07]	0.7%
Panning, 2010 − Liberia	35	184	19.02	[13.62; 25.45]	0.7%
Panning, 2010 − Liberia	38	184	20.65	[15.05; 27.23]	0.7%
Shaffer, 2019 − Sierra Leone	389	4229	9.20	[8.34; 10.11]	0.7%
Shehu, 2018 − Nigeria	11	34	32.35	[17.39; 50.53]	0.6%
**Random effect meta−analysis**		**20008**	**25.17**	**[20.62; 30.01]**	**13.4%**
Heterogeneity: $I^2 = 97.7\%$ [97.2%; 98.1%], $\tau^2 = 0.0129$, $p < 0.0001$					
**Current contact _ Patient with hemorrhagic fever (2 Studies)**					
Li, 2016 − Sierra Leone	36	278	12.95	[9.24; 17.47]	0.7%
Salu, 2019 − Benin	5	70	7.14	[2.36; 15.89]	0.7%
**Random effect meta−analysis**		**348**	**10.84**	**[6.20; 16.51]**	**1.3%**
Heterogeneity: $I^2 = 43\%$, $\tau^2 = 0.0017$, $p = 0.1853$					
**Current contact _ Pregnant women (1 Study)**					
Okogbenin, 2019 − Nigeria	44	5048	0.87	[0.63; 1.17]	0.7%
**Random effect meta−analysis**		**5048**	**0.87**	**[0.63; 1.15]**	**0.7%**
Heterogeneity: not applicable					
**Overall random effect meta−analysis**		**88212**	**8.77**	**[6.85; 10.88]**	**100.0%**
Residual heterogeneity: $I^2 = 98.8\%$ [98.7%; 98.9%], $p = 0$					

0 20 40 60 80

Reference

1. Akhuemokhan OC, Ewah-Odiase RO, Akpede N, Ehimuan J, Adomeh DI, Odia I, et al. Prevalence of Lassa Virus Disease (LVD) in Nigerian children with fever or fever and convulsions in an endemic area. PLoS Negl Trop Dis. 2017;11. doi:10.1371/journal.pntd.0005711

2. Akoua-Koffi C, Ter Meulen J, Legros D, Akran V, Aïdara M, Nahounou N, et al. [Detection of anti-Lassa antibodies in the Western Forest area of the Ivory Coast]. Med Trop (Mars). 2006;66: 465–468.

3. Arnold RB, Gary GW. A neutralization test survey for Lassa fever activity in Lassa, Nigeria. Transactions of the Royal Society of Tropical Medicine and Hygiene. 1977;71: 152–154. doi:10.1016/0035-9203(77)90085-2

4. Asogun DA, Adomeh DI, Ehimuan J, Odia I, Hass M, Gabriel M, et al. Molecular Diagnostics for Lassa Fever at Irrua Specialist Teaching Hospital, Nigeria: Lessons Learnt from Two Years of Laboratory Operation. PLoS Negl Trop Dis. 2012;6. doi:10.1371/journal.pntd.0001839

5. Babalola SO, Babatunde JA, Remilekun OM, Amaobichukwu AR, Abiodun AM, Jide I, et al. Lassa virus RNA detection from suspected cases in Nigeria, 2011-2017. Pan Afr Med J. 2019;34. doi:10.11604/pamj.2019.34.76.16425

6. Bajani MD, Tomori O, Rollin PE, Harry TO, Bukbuk ND, Wilson L, et al. A survey for antibodies to Lassa virus among health workers in Nigeria. Transactions of the Royal Society of Tropical Medicine and Hygiene. 1997;91: 379–381. doi:10.1016/S0035-9203(97)90247-9

7. Baumann J, Knüpfer M, Ouedraogo J, Traoré BY, Heitzer A, Kané B, et al. Lassa and Crimean-Congo Hemorrhagic Fever Viruses, Mali. Emerging Infect Dis. 2019;25: 999–1002. doi:10.3201/eid2505.181047

8. Bausch DG, Demby AH, Coulibaly M, Kanu J, Goba A, Bah A, et al. Lassa fever in Guinea: I. Epidemiology of human disease and clinical observations. Vector Borne Zoonotic Dis. 2001;1: 269–281. doi:10.1089/15303660160025903

9. Blackburn NK, Searle L, Taylor P. Viral haemorrhagic fever antibodies in Zimbabwe schoolchildren. Transactions of the Royal Society of Tropical Medicine and Hygiene. 1982;76: 803–805. doi:10.1016/0035-9203(82)90113-4

10. Boiro I, Lomonossov NN, Sotsinski VA, Constantinov OK, Tkachenko EA, Inapogui AP, et al. [Clinico-epidemiologic and laboratory research on hemorrhagic fevers in Guinea]. Bull Soc Pathol Exot Filiales. 1987;80: 607–612.

11. Bonney JHK, Osei-Kwasi M, Adiku TK, Barnor JS, Amesiya R, Kubio C, et al. Hospital-Based Surveillance for Viral Hemorrhagic Fevers and Hepatitides in Ghana. Kasper M, editor. PLoS Negl Trop Dis. 2013;7: e2435. doi:10.1371/journal.pntd.0002435

12. Bowen GS, Wulff H, Casals J, Noonan A, Downs WG. Lassa fever in Onitsha, East Central State, Nigeria, in 1974. 1975; 6.

13. Branco LM, Grove JN, Boisen ML, Shaffer JG, Goba A, Fullah M, et al. Emerging trends in Lassa fever: redefining the role of immunoglobulin M and inflammation in diagnosing acute infection. Virol J. 2011;8: 478. doi:10.1186/1743-422X-8-478

14. Bukbuk DN, Fukushi S, Tani H, Yoshikawa T, Taniguchi S, Iha K, et al. Development and validation of serological assays for viral hemorrhagic fevers and determination of the prevalence

of Rift Valley fever in Borno State, Nigeria. Transactions of The Royal Society of Tropical Medicine and Hygiene. 2014;108: 768–773. doi:10.1093/trstmh/tru163

15. Carey DE, Kemp GE, White HA, Pinneo L, Addy RF, Fom ALMD, et al. Lassa fever Epidemiological aspects of the 1970 epidemic, Jos, Nigeria. Trans R Soc Trop Med Hyg. 1972;66: 402–408. doi:10.1016/0035-9203(72)90271-4

16. Clements TL, Rossi CA, Irish AK, Kibuuka H, Eller LA, Robb ML, et al. Chikungunya and O'nyong-nyong Viruses in Uganda: Implications for Diagnostics. Open Forum Infectious Diseases. 2019;6. doi:10.1093/ofid/ofz001

17. Cummins D, McCormick JB, Bennett D, Samba JA, Farrar B, Machin SJ, et al. Acute sensorineural deafness in Lassa fever. JAMA. 1990;264: 2093–2096.

18. Dahmane A, van Griensven J, Van Herp M, Van den Bergh R, Nzomukunda Y, Prior J, et al. Constraints in the diagnosis and treatment of Lassa Fever and the effect on mortality in hospitalized children and women with obstetric conditions in a rural district hospital in Sierra Leone. Trans R Soc Trop Med Hyg. 2014;108: 126–132. doi:10.1093/trstmh/tru009

19. Dan-Nwafor CC, Furuse Y, Ilori EA, Ipadeola O, Akabike KO, Ahumibe A, et al. Measures to control protracted large Lassa fever outbreak in Nigeria, 1 January to 28 April 2019. Eurosurveillance. 2019;24. doi:10.2807/1560-7917.ES.2019.24.20.1900272

20. Dan-Nwafor CC, Ipadeola O, Smout E, Ilori E, Adeyemo A, Umeokonkwo C, et al. A cluster of nosocomial Lassa fever cases in a tertiary health facility in Nigeria: Description and lessons learned, 2018. International Journal of Infectious Diseases. 2019;83: 88–94. doi:10.1016/j.ijid.2019.03.030

21. Demby AH, Chamberlain J, Brown DW, Clegg CS. Early diagnosis of Lassa fever by reverse transcription-PCR. Journal of Clinical Microbiology. 1994;32: 2898–2903. doi:10.1128/JCM.32.12.2898-2903.1994

22. Ehichioya DU, Asogun DA, Ehimuan J, Okokhere PO, Pahlmann M, Ölschläger S, et al. Hospital-based surveillance for Lassa fever in Edo State, Nigeria, 2005-2008: Lassa fever in Edo State, Nigeria. Tropical Medicine & International Health. 2012;17: 1001–1004. doi:10.1111/j.1365-3156.2012.03010.x

23. Emmerich P, Thome-Bolduan C, Drosten C, Gunther S, Ban E, Sawinsky I, et al. Reverse ELISA for IgG and IgM antibodies to detect Lassa virus infections in Africa. Journal of Clinical Virology. 2006;37: 277–281. doi:10.1016/j.jcv.2006.08.015

24. Emmerich P, Günther S, Schmitz H. Strain-specific antibody response to Lassa virus in the local population of west Africa. Journal of Clinical Virology. 2008;42: 40–44. doi:10.1016/j.jcv.2007.11.019

25. Fabiyi A. Use of the complement fixation (CF) test in Lassa fever surveillance. 1975; 4.

26. Fabiyi A, Tomori O, Pinneo P. Lassa fever antibodies in hospital personnel in the Plateau State of Nigeria. Niger Med J. 1979;9: 23–25.

27. Fisher-Hoch S, McCormick JB, Sasso D, Craven RB. Hematologic dysfunction in Lassa fever. J Med Virol. 1988;26: 127–135. doi:10.1002/jmv.1890260204

28. Fisher-Hoch SP, Tomori O, Nasidi A, Perez-Oronoz GI, Fakile Y, Hutwagner L, et al. Review of cases of nosocomial Lassa fever in Nigeria: the high price of poor medical practice. BMJ. 1995;311: 857–859.

29. Frame JD, Casals J, Dennis EA. Lassa virus antibodies in hospital personnel in western Liberia. Transactions of the Royal Society of Tropical Medicine and Hygiene. 1979;73: 219–224. doi:10.1016/0035-9203(79)90218-9

30. Frame JD, Jahrling PB, Yalley-Ogunro JE, Monson MH. Endemic Lassa fever in Liberia. II. Serological and virological findings in hospital patients. Trans R Soc Trop Med Hyg. 1984;78: 656–660. doi:10.1016/0035-9203(84)90232-3

31. Frame JD, Yalley-Ogunro JE, Hanson AP. Endemic Lassa fever in Liberia. V. Distribution of Lassa virus activity in Liberia: hospital staff surveys. Transactions of the Royal Society of Tropical Medicine and Hygiene. 1984;78: 761–763. doi:10.1016/0035-9203(84)90012-9

32. Georges AJ, Gonzalez JP, Abdul-Wahid S, Saluzzo JF, Meunier DMY, McCormick JB. Antibodies to Lassa and lassa-like viruses in man and mammals in the Central African Republic. Transactions of the Royal Society of Tropical Medicine and Hygiene. 1985;79: 78–79. doi:10.1016/0035-9203(85)90242-1

33. Gonzalez JP, McCormick JB, Saluzzo JF, Herve JP, Georges AJ, Johnson KM. An arenavirus isolated from wild-caught rodents (Pramys species) in the Central African Republic. Intervirology. 1983;19: 105–112. doi:10.1159/000149344

34. Gonzalez JP, Josse R, Johnson ED, Merlin M, Georges AJ, Abandja J, et al. Antibody prevalence against haemorrhagic fever viruses in randomized representative central African populations. Research in Virology. 1989;140: 319–331. doi:10.1016/S0923-2516(89)80112-8

35. Hamblion EL, Raftery P, Wendland A, Dweh E, Williams GS, George RNC, et al. The challenges of detecting and responding to a Lassa fever outbreak in an Ebola-affected setting. International Journal of Infectious Diseases. 2018;66: 65–73. doi:10.1016/j.ijid.2017.11.007

36. Helmick C, Scribner C, Webb P, Krebs J, Mccormick J. NO EVIDENCE FOR INCREASED RISK OF LASSA FEVER INFECTION IN HOSPITAL STAFF. The Lancet. 1986;328: 1202–1205. doi:10.1016/S0140-6736(86)92206-3

37. Henderson BE, Gary GW, Kissling RE, Frame JD, Carey DE. Lassa fever virological and serological studies. Transactions of the Royal Society of Tropical Medicine and Hygiene. 1972;66: 409–416. doi:10.1016/0035-9203(72)90272-6

38. Ibekwe T, Nwegbu M, Okokhere P, Adomeh D, Asogun D. The sensitivity and specificity of Lassa virus IgM by ELISA as screening tool at early phase of Lassa fever infection. Niger Med J. 2012;53: 196. doi:10.4103/0300-1652.107552

39. Ipadeola O, Furuse Y, Ilori EA, Dan-Nwafor CC, Akabike KO, Ahumibe A, et al. Epidemiology and case-control study of Lassa fever outbreak in Nigeria from 2018 to 2019. Journal of Infection. 2020; S0163445320300013. doi:10.1016/j.jinf.2019.12.020

40. Isere EE, Fatiregun A, Ilesanmi O, Ijarotimi I, Egube B, Adejugbagbe A, et al. Lessons Learnt from Epidemiological Investigation of Lassa Fever Outbreak in a Southwest State of Nigeria December 2015 to April 2016. PLoS Curr. 2018 [cited 25 Feb 2020]. doi:10.1371/currents.outbreaks.bc4396a6650d0ed1985d731583bf5ded

41. Ivanoff' B, Duquesnoy' P, Languillat' G, Saluzzo' JF, Georges' A, Gonzalez' JP, et al. Haemorrhagic fever in Gabon. I. Incidence of Lassa, Ebola and Marburg viruses in Haut-Ogoou6. 1982; 2.

42. Jahrling PB, Frame JD, Smith SB, Monson MH. Endemic Lassa fever in Liberia. III. Characterization of Lassa virus isolates. Transactions of the Royal Society of Tropical Medicine and Hygiene. 1985;79: 374–379. doi:10.1016/0035-9203(85)90386-4

43. Johnson' BK, Gitau LG, Gichogop A, Tukei' M, Else' JG, Suleman MA, et al. Marburg, Ebota and Rift Valley fever virus antibodies in East African primates. Transactions of the Royal Society of Tropical Medicine and Hygiene. 1982; 4.

44. Johnson BK, Ocheng D, Gichogo A, Okiro M, Libondo D, Tukei PM, et al. Antibodies against haemorrhagic fever viruses in Kenya populations. Transactions of the Royal Society of Tropical Medicine and Hygiene. 1983;77: 731–733. doi:10.1016/0035-9203(83)90216-X

45. Johnson BK, Ocheng D, Gitau LG, Gichogo A, Tukei PM, Ngindu A, et al. Viral Haemorrhagic Fever Surveillance in Kenya, 1980-198. 1983; 1.

46. Johnson ED, Gonzalez JP, Georges A. Haemorrhagic fever virus activity in equatorial Africa: distribution and prevalence of filovirus reactive antibody in the Central African Republic. Transactions of the Royal Society of Tropical Medicine and Hygiene. 1993;87: 530–535. doi:10.1016/0035-9203(93)90075-2

47. Keane E, Gilles HM. Lassa fever in Panguma Hospital, Sierra Leone, 1973-6. BMJ. 1977;1: 1399–1402. doi:10.1136/bmj.1.6073.1399

48. Keenlyside RA, McCormick JB, Webb PA, Smith E, Elliott L, Johnson KM. Case-control study of Mastomys natalensis and humans in Lassa virus-infected households in Sierra Leone. Am J Trop Med Hyg. 1983;32: 829–837. doi:10.4269/ajtmh.1983.32.829

49. Kernéis S, Koivogui L, Magassouba N, Koulemou K, Lewis R, Aplogan A, et al. Prevalence and Risk Factors of Lassa Seropositivity in Inhabitants of the Forest Region of Guinea: A Cross-Sectional Study. Aksoy S, editor. PLoS Negl Trop Dis. 2009;3: e548. doi:10.1371/journal.pntd.0000548

50. Klempa B, Koulemou K, Auste B, Emmerich P, Thomé-Bolduan C, Günther S, et al. Seroepidemiological study reveals regional co-occurrence of Lassa- and Hantavirus antibodies in Upper Guinea, West Africa. Trop Med Int Health. 2012; n/a-n/a. doi:10.1111/tmi.12045

51. Bloch A. A serological survey of Lassa fever in Liberia. 1978; 3.

52. Li W-G, Chen W-W, Li L, Ji D, Ji Y-J, Li C, et al. The etiology of Ebola virus disease-like illnesses in Ebola virusnegative patients from Sierra Leone. Oncotarget. 2016;7. doi:10.18632/oncotarget.8558

53. Lukashevich IS, Clegg JC, Sidibe K. Lassa virus activity in Guinea: distribution of human antiviral antibody defined using enzyme-linked immunosorbent assay with recombinant antigen. J Med Virol. 1993;40: 210–217. doi:10.1002/jmv.1890400308

54. Maigari IM, Jibrin YB, Umar MS, Lawal SM, Gandi AY. Descriptive features of Lassa fever in Bauchi, Northeastern Nigeria - a retrospective review. Research Journal of Health Sciences. 2018;6: 149. doi:10.4314/rejhs.v6i3.7

55. Mathiot CC, Fontenille D, Georges AJ, Coulanges P. Antibodies to haemorrhagic fever viruses in Madagascar populations. Transactions of the Royal Society of Tropical Medicine and Hygiene. 1989;83: 407–409. doi:10.1016/0035-9203(89)90519-1

56. McCarthy MC, Haberberger RL, Salib AW, Soliman BA, El-Tigani A, Watts DM. Evaluation of arthropod-borne viruses and other infectious disease pathogens as the causes of febrile illnesses in the Khartoum Province of Sudan. 1996; 6.

57. McCormick JB, King IJ, Webb PA, Johnson KM, O'Sullivan R, Smith ES, et al. A Case-Control Study of the Clinical Diagnosis and Course of Lassa Fever. J Infect Dis. 1987;155: 445–455. doi:10.1093/infdis/155.3.445

58. McCormick JB, Webb PA, Krebs JW, Johnson KM, Smith ES. A Prospective Study of the Epidemiology and Ecology of Lassa Fever. J Infect Dis. 1987;155: 437–444. doi:10.1093/infdis/155.3.437

59. Meunier DM, Johnson ED, Gonzalez JP, Georges-Courbot MC, Madelon MC, Georges AJ. [Current serologic data on viral hemorrhagic fevers in the Central African Republic]. Bull Soc Pathol Exot Filiales. 1987;80: 51–61.

60. Monath TP, Mertens PE, Patton R, Moser CR, Baum JJ, Pinneo L, et al. A hospital epidemic of Lassa fever in Zorzor, Liberia, March-April 1972. Am J Trop Med Hyg. 1973;22: 773–779. doi:10.4269/ajtmh.1973.22.773

61. Nakounné E, Selekon B, Morvan J. Veille microbiologique : les fièvres hémorragiques virales en République centrafricaine ; 2000; 8.

62. Nimo-Paintsil SC, Fichet-Calvet E, Borremans B, Letizia AG, Mohareb E, Bonney JHK, et al. Rodent-borne infections in rural Ghanaian farming communities. Schieffelin J, editor. PLoS ONE. 2019;14: e0215224. doi:10.1371/journal.pone.0215224

63. O'Hearn AE, Voorhees MA, Fetterer DP, Wauquier N, Coomber MR, Bangura J, et al. Serosurveillance of viral pathogens circulating in West Africa. Virol J. 2016;13: 163. doi:10.1186/s12985-016-0621-4

64. Okogbenin S, Okoeguale J, Akpede G, Colubri A, Barnes KG, Mehta S, et al. Retrospective Cohort Study of Lassa Fever in Pregnancy, Southern Nigeria. Emerg Infect Dis. 2019;25: 1494–1500. doi:10.3201/eid2508.181299

65. Oloniniyi OK, Unigwe US, Okada S, Kimura M, Koyano S, Miyazaki Y, et al. Genetic characterization of Lassa virus strains isolated from 2012 to 2016 in southeastern Nigeria. PLoS Negl Trop Dis. 2018;12. doi:10.1371/journal.pntd.0006971

66. Omilabu SA, Badaru SO, Okokhere P, Asogun D, Drosten C, Emmerich P, et al. Lassa Fever, Nigeria, 2003 and 2004. Emerg Infect Dis. 2005;11: 1642–1644. doi:10.3201/eid1110.041343

67. Paix MA, Poveda JD, Malvy D, Bailly C, Merlin M, Fleury HJ. [Serological study of the virus responsible for hemorrhagic fever in an urban population of Cameroon]. Bull Soc Pathol Exot Filiales. 1988;81: 679–682.

68. Panning M, Emmerich P, Ölschläger S, Bojenko S, Koivogui L, Marx A, et al. Laboratory Diagnosis of Lassa Fever, Liberia. Emerg Infect Dis. 2010;16: 1041–1043. doi:10.3201/eid1606.100040

69. Rodhain F, Gonzalez JP, Mercier E, Helynck B, Larouze B, Hannoun C. Arbovirus infections and viral haemorrhagic fevers in Uganda: a serological survey in Karamoja district, 1984. Transactions of the Royal Society of Tropical Medicine and Hygiene. 1989;83: 851–854. doi:10.1016/0035-9203(89)90352-0

70. Salu OB, James AB, Bankolé HS, Agbla JM, Da Silva M, Gbaguidi F, et al. Molecular confirmation and phylogeny of Lassa fever virus in Benin Republic 2014–2016. African Journal of Laboratory Medicine. 2019;8. doi:10.4102/ajlm.v8i1.803

71. Schoepp RJ, Rossi CA, Khan SH, Goba A, Fair JN. Undiagnosed Acute Viral Febrile Illnesses, Sierra Leone. Emerg Infect Dis. 2014;20: 1176–1182. doi:10.3201/eid2007.131265

72. Shaffer JG, Schieffelin JS, Gbakie M, Alhasan F, Roberts NB, Goba A, et al. A medical records and data capture and management system for Lassa fever in Sierra Leone: Approach, implementation, and challenges. Verdonck K, editor. PLoS ONE. 2019;14: e0214284. doi:10.1371/journal.pone.0214284

73. Shehu NY, Gomerep SS, Isa SE, Iraoyah KO, Mafuka J, Bitrus N, et al. Lassa Fever 2016 Outbreak in Plateau State, Nigeria—The Changing Epidemiology and Clinical Presentation. Front Public Health. 2018;6: 232. doi:10.3389/fpubh.2018.00232

74. Smith EA, Fabiyi A, Kuteyi OE, Tomori O. Epidemiological aspect of the 1976 Pankshin Lassa fever outbreak. Niger Med J. 1979;9: 20–22.

75. Sogoba N, Rosenke K, Adjemian J, Diawara SI, Maiga O, Keita M, et al. Lassa Virus Seroprevalence in Sibirilia Commune, Bougouni District, Southern Mali. Emerg Infect Dis. 2016;22: 657–663. doi:10.3201/eid2204.151814

76. ter Meulen J, Koulemou K, Wittekindt T, Windisch K, Strigl S, Conde S, et al. Detection of Lassa Virus Antinucleoprotein Immunoglobulin G (IgG) and IgM Antibodies by a Simple Recombinant Immunoblot Assay for Field Use. Journal of Clinical Microbiology. 1998;36: 3143–3148. doi:10.1128/JCM.36.11.3143-3148.1998

77. ter Meulen J, Lenz O, Koivogui L, Magassouba N, Kaushik SK, Lewis R, et al. Short communication: Lassa fever in Sierra Leone: UN peacekeepers are at risk. Trop Med Int Health. 2001;6: 83–84. doi:10.1046/j.1365-3156.2001.00676.x

78. Tomori O, Fabiyi A, Sorungbe A, Smith A, McCormick JB. Viral hemorrhagic fever antibodies in Nigerian populations. Am J Trop Med Hyg. 1988;38: 407–410. doi:10.4269/ajtmh.1988.38.407

79. Troup JM, White HA, Fom AL, Carey DE. An outbreak of Lassa fever on the Jos plateau, Nigeria, in January-February 1970. A preliminary report. Am J Trop Med Hyg. 1970;19: 695–696. doi:10.4269/ajtmh.1970.19.695

80. Van der Waals FW, Pomeroy KL, Goudsmit J, Asher DM, Gajdusek DC. Hemorrhagic fever virus infections in an isolated rainforest area of central Liberia. Limitations of the indirect immunofluorescence slide test for antibody screening in Africa. Trop Geogr Med. 1986;38: 209–214.

81. Webb PA, McCormick JB, King IJ, Bosman I, Johnson KM, Elliott LH, et al. Lassa fever in children in Sierra Leone, West Africa. Trans R Soc Trop Med Hyg. 1986;80: 577–582. doi:10.1016/0035-9203(86)90147-1

82. Yalley-Ogunro JE, Frame JD, Hanson AP. Endemic Lassa fever in Liberia. VI. Village serological surveys for evidence of Lassa virus activity in Lofa County, Liberia. Transactions of the Royal Society of Tropical Medicine and Hygiene. 1984;78: 764–770. doi:10.1016/0035-9203(84)90013-0
