# Supplementary material for: Systematic review and meta-analysis of the epidemiology of Lassa virus in humans, rodents and other mammals in sub-Saharan Africa
Source: PLoS Negl Trop Dis. 2020 Aug 26;14(8):e0008589. doi: 10.1371/journal.pntd.0008589 (PMC7478710; doi:10.1371/journal.pntd.0008589)
Supplement: S2 Fig — (PDF) [file pntd.0008589.s011.pdf]

S2 Fig: Prevalence of Lassa virus infections in rodents in sub-Saharan Africa

Study	Positive	Total	Prevalence (%)	95% CI	Weight
**Current contact_ Hylomyscus pamfi (1 Study)**					
Olayemi, 2016 − Nigeria	5	12	41.67	[15.17; 72.33]	0.6%
**Random effect meta−analysis**		**12**	**41.67**	**[14.90; 70.99]**	**0.6%**
Heterogeneity: not applicable					
**Current contact_ Mus baoulei (1 Study)**					
Olayemi, 2018 − Nigeria	0	15	0.00	[0.00; 21.80]	0.6%
Yadouleton, 2019 − Benin	3	14	21.43	[4.66; 50.80]	0.6%
**Random effect meta−analysis**		**29**	**6.96**	**[0.00; 39.10]**	**1.3%**
Heterogeneity: I^2 = 76.5% [0.0%; 94.7%], τ^2 = 0.0543, p = 0.0391					
**Current contact_ Mastomys species (7 Studies)**					
Leski (GPC gene), 2015 − Sierra Leone	34	214	15.89	[11.26; 21.49]	1.0%
Leski (NP gene), 2015 − Sierra Leone	39	214	18.22	[13.29; 24.06]	1.0%
Leski (NP gene), 2015 − Sierra Leone	33	214	15.42	[10.86; 20.97]	1.0%
Leski, 2015 − Sierra Leone	9	51	17.65	[8.40; 30.87]	0.9%
Safronetz, 2010 − Mali	6	82	7.32	[2.73; 15.25]	0.9%
Safronetz, 2010 − Mali	6	82	7.32	[2.73; 15.25]	0.9%
Safronetz, 2010 − Mali	6	82	7.32	[2.73; 15.25]	0.9%
**Random effect meta−analysis**		**939**	**12.84**	**[9.46; 16.64]**	**6.6%**
Heterogeneity: I^2 = 59.3% [6.4%; 82.3%], τ^2 = 0.0028, p = 0.0223					
**Current contact_ Mastomys natalensis (10 Studies)**					
Coulibaly−N'Golo, 2011 − Ivory Coast	0	729	0.00	[0.00; 0.50]	1.0%
Fichet−Calvet, 2007 − Guinea	80	553	14.47	[11.64; 17.68]	1.0%
Kouadio , 2015 − Ivory Coast	4	18	22.22	[6.41; 47.64]	0.7%
Lalis , 2012 − Guinea	69	656	10.52	[8.28; 13.12]	1.0%
Lecompte, 2006 − Guinea	96	847	11.33	[9.28; 13.66]	1.0%
Olayemi, 2016 − Nigeria	0	41	0.00	[0.00; 8.60]	0.8%
Olayemi, 2016 − Nigeria	15	274	5.47	[3.10; 8.87]	1.0%
Olayemi, 2018 − Nigeria	16	273	5.86	[3.39; 9.34]	1.0%
Safronetz, 2013 − Mali	19	591	3.21	[1.95; 4.98]	1.0%
Safronetz, 2013 − Mali	19	591	3.21	[1.95; 4.98]	1.0%
**Random effect meta−analysis**		**4573**	**5.47**	**[2.11; 10.12]**	**9.6%**
Heterogeneity: I^2 = 96.8% [95.4%; 97.7%], τ^2 = 0.0170, p < 0.0001					
**Current contact_ Mastomys erythroleucus (5 Studies)**					
Fichet−Calvet, 2007 − Guinea	0	65	0.00	[0.00; 5.52]	0.9%
Lecompte, 2006 − Guinea	0	202	0.00	[0.00; 1.81]	1.0%
Olayemi, 2016 − Guinea	6	16	37.50	[15.20; 64.57]	0.7%
Olayemi, 2016 − Nigeria	3	63	4.76	[0.99; 13.29]	0.9%
Olayemi, 2018 − Nigeria	3	116	2.59	[0.54; 7.37]	1.0%
**Random effect meta−analysis**		**462**	**3.05**	**[0.00; 10.28]**	**4.4%**
Heterogeneity: I^2 = 87.1% [72.3%; 94.0%], τ^2 = 0.0206, p < 0.0001					
**Current contact_ Mus (Nannomys) species (1 Study)**					
Lecompte, 2006 − Guinea	2	165	1.21	[0.15; 4.31]	1.0%
**Random effect meta−analysis**		**165**	**1.21**	**[0.02; 3.62]**	**1.0%**
Heterogeneity: not applicable					
**Current contact_*Negative Rodents (27 Studies)**					
Coulibaly−N'Golo, 2011 − Ivory Coast	0	52	0.00	[0.00; 6.85]	0.9%
Coulibaly−N'Golo, 2011 − Ivory Coast	0	48	0.00	[0.00; 7.40]	0.9%
Coulibaly−N'Golo, 2011 − Ivory Coast	0	32	0.00	[0.00; 10.89]	0.8%
Coulibaly−N'Golo, 2011 − Ivory Coast	0	74	0.00	[0.00; 4.86]	0.9%
Coulibaly−N'Golo, 2011 − Ivory Coast	0	27	0.00	[0.00; 12.77]	0.8%
Coulibaly−N'Golo, 2011 − Ivory Coast	0	17	0.00	[0.00; 19.51]	0.7%
Fichet−Calvet, 2007 − Guinea	0	16	0.00	[0.00; 20.59]	0.7%
Fichet−Calvet, 2007 − Guinea	0	41	0.00	[0.00; 8.60]	0.8%
Fichet−Calvet, 2007 − Guinea	0	23	0.00	[0.00; 14.82]	0.7%
Fichet−Calvet, 2007 − Guinea	0	109	0.00	[0.00; 3.33]	0.9%
Lecompte, 2006 − Guinea	0	15	0.00	[0.00; 21.80]	0.6%
Lecompte, 2006 − Guinea	0	39	0.00	[0.00; 9.03]	0.8%
Lecompte, 2006 − Guinea	0	40	0.00	[0.00; 8.81]	0.8%
Lecompte, 2006 − Guinea	0	117	0.00	[0.00; 3.10]	1.0%
Lecompte, 2006 − Guinea	0	49	0.00	[0.00; 7.25]	0.9%
Lecompte, 2006 − Guinea	0	59	0.00	[0.00; 6.06]	0.9%
Lecompte, 2006 − Guinea	0	29	0.00	[0.00; 11.94]	0.8%
Olayemi, 2018 − Nigeria	0	19	0.00	[0.00; 17.65]	0.7%
Olayemi, 2018 − Nigeria	0	17	0.00	[0.00; 19.51]	0.7%
Olayemi, 2018 − Nigeria	0	16	0.00	[0.00; 20.59]	0.7%
Olayemi, 2018 − Nigeria	0	12	0.00	[0.00; 26.46]	0.6%
Olayemi, 2018 − Nigeria	0	20	0.00	[0.00; 16.84]	0.7%
Olayemi, 2018 − Nigeria	0	187	0.00	[0.00; 1.95]	1.0%
Yadouleton, 2019 − Benin	0	26	0.00	[0.00; 13.23]	0.8%
Yadouleton, 2019 − Benin	0	10	0.00	[0.00; 30.85]	0.5%
Yadouleton, 2019 − Benin	0	12	0.00	[0.00; 26.46]	0.6%
Yadouleton, 2019 − Benin	0	210	0.00	[0.00; 1.74]	1.0%
**Random effect meta−analysis**		**1316**	**0.00**	**[0.00; 0.00]**	**21.1%**
Heterogeneity: I^2 = 0% [<0.0%; <0.0%], τ^2 = 0, p = 1.0000					
**Current contact_ Unspecified rodents (21 Studies)**					
Agbonlahor, 2017 − Nigeria	17	47	36.17	[22.67; 51.48]	0.9%
Agbonlahor, 2017 − Nigeria	0	337	0.00	[0.00; 1.09]	1.0%
Agbonlahor, 2017 − Nigeria	4	546	0.73	[0.20; 1.87]	1.0%
Agbonlahor, 2017 − Nigeria	3	594	0.51	[0.10; 1.47]	1.0%
Boiro, 1987 − Guinea	4	158	2.53	[0.69; 6.35]	1.0%
Demartini, 1975 − Sierra Leone	14	28	50.00	[30.65; 69.35]	0.8%
Demby, 2001 − Guinea	0	49	0.00	[0.00; 7.25]	0.9%
Demby, 2001 − Guinea	2	309	0.65	[0.08; 2.32]	1.0%
Demby, 2001 − Guinea	46	884	5.20	[3.83; 6.88]	1.0%
Fair, 2007 − Guinea	7	105	6.67	[2.72; 13.25]	0.9%
Keenlyside, 1983 − Sierra Leone	2	66	3.03	[0.37; 10.52]	0.9%
Mariën, 2017 − Guinea	168	1139	14.75	[12.74; 16.94]	1.0%
Monath, 1974 − Sierra Leone	14	82	17.07	[9.66; 26.98]	0.9%
Monath, 1974 − Sierra Leone	0	268	0.00	[0.00; 1.37]	1.0%
Safronetz, 2010 − Mali	0	21	0.00	[0.00; 16.11]	0.7%
Safronetz, 2010 − Mali	0	21	0.00	[0.00; 16.11]	0.7%
Safronetz, 2010 − Mali	0	21	0.00	[0.00; 16.11]	0.7%
Wulff, 1975 − Nigeria	1	23	4.35	[0.11; 21.95]	0.7%
Wulff, 1975 − Nigeria	5	32	15.62	[5.28; 32.79]	0.8%
Wulff, 1975 − Nigeria	2	37	5.41	[0.66; 18.19]	0.8%
Wulff, 1975 − Nigeria	0	59	0.00	[0.00; 6.06]	0.9%
**Random effect meta−analysis**		**4826**	**4.12**	**[1.45; 7.79]**	**18.7%**
Heterogeneity: I^2 = 95.4% [94.1%; 96.5%], τ^2 = 0.0249, p < 0.0001					
**Past contact_ Mus baoulei (1 Study)**					
Olayemi, 2018 − Nigeria	5	15	33.33	[11.82; 61.62]	0.6%
**Random effect meta−analysis**		**15**	**33.33**	**[11.30; 59.49]**	**0.6%**
Heterogeneity: not applicable					
**Past contact_ Mastomys natalensis (3 Studies)**					
Fichet−Calvet, 2014 − Guinea, Mali	109	596	18.29	[15.26; 21.63]	1.0%
Olayemi, 2018 − Nigeria	40	273	14.65	[10.68; 19.41]	1.0%
Safronetz, 2013 − Mali	35	511	6.85	[4.82; 9.40]	1.0%
**Random effect meta−analysis**		**1380**	**12.83**	**[6.23; 21.33]**	**3.0%**
Heterogeneity: I^2 = 94.3% [86.8%; 97.6%], τ^2 = 0.0094, p < 0.0001					
**Past contact_ Lemniscomys striatus (1 Study)**					
Fichet−Calvet, 2014 − Guinea, Mali	4	32	12.50	[3.51; 28.99]	0.8%
**Random effect meta−analysis**		**32**	**12.50**	**[2.88; 26.59]**	**0.8%**
Heterogeneity: not applicable					
**Past contact_ Mastomys erythroleucus (2 Studies)**					
Fichet−Calvet, 2014 − Guinea, Mali	7	251	2.79	[1.13; 5.66]	1.0%
Olayemi, 2018 − Nigeria	16	116	13.79	[8.09; 21.43]	1.0%
**Random effect meta−analysis**		**367**	**7.18**	**[0.22; 21.45]**	**1.9%**
Heterogeneity: I^2 = 93% [76.7%; 97.9%], τ^2 = 0.0208, p = 0.0002					
**Past contact_ Mus minutoides (2 Studies)**					
Fichet−Calvet, 2014 − Guinea, Mali	3	40	7.50	[1.57; 20.39]	0.8%
Olayemi, 2018 − Nigeria	1	19	5.26	[0.13; 26.03]	0.7%
**Random effect meta−analysis**		**59**	**6.58**	**[1.10; 15.00]**	**1.5%**
Heterogeneity: I^2 = 0%, τ^2 = 0, p = 0.8723					
**Past contact_ Praomys daltoni (2 Studies)**					
Fichet−Calvet, 2014 − Guinea, Mali	4	112	3.57	[0.98; 8.89]	1.0%
Olayemi, 2018 − Nigeria	6	187	3.21	[1.19; 6.85]	1.0%
**Random effect meta−analysis**		**299**	**3.32**	**[1.50; 5.75]**	**1.9%**
Heterogeneity: I^2 = 0%, τ^2 = 0, p = 0.8182					
**Past contact_ Rattus rattus (2 Studies)**					
Fichet−Calvet, 2014 − Guinea, Mali	0	72	0.00	[0.00; 4.99]	0.9%
Olayemi, 2018 − Nigeria	2	20	10.00	[1.23; 31.70]	0.7%
**Random effect meta−analysis**		**92**	**2.24**	**[0.00; 19.95]**	**1.6%**
Heterogeneity: I^2 = 81.7% [22.3%; 95.7%], τ^2 = 0.0348, p = 0.0196					
**Past contact_ Praomys rostratus (1 Study)**					
Fichet−Calvet, 2014 − Guinea, Mali	2	163	1.23	[0.15; 4.36]	1.0%
**Random effect meta−analysis**		**163**	**1.23**	**[0.02; 3.66]**	**1.0%**
Heterogeneity: not applicable					
**Past contact_#Negative rondents (10 Studies)**					
Fichet−Calvet, 2014 − Guinea, Mali	0	93	0.00	[0.00; 3.89]	0.9%
Fichet−Calvet, 2014 − Guinea, Mali	0	49	0.00	[0.00; 7.25]	0.9%
Fichet−Calvet, 2014 − Guinea, Mali	0	21	0.00	[0.00; 16.11]	0.7%
Fichet−Calvet, 2014 − Guinea, Mali	0	20	0.00	[0.00; 16.84]	0.7%
Fichet−Calvet, 2014 − Guinea, Mali	0	13	0.00	[0.00; 24.71]	0.6%
Fichet−Calvet, 2014 − Guinea, Mali	0	10	0.00	[0.00; 30.85]	0.5%
Fichet−Calvet, 2014 − Guinea, Mali	0	28	0.00	[0.00; 12.34]	0.8%
Olayemi, 2018 − Nigeria	0	16	0.00	[0.00; 20.59]	0.7%
Olayemi, 2018 − Nigeria	0	17	0.00	[0.00; 19.51]	0.7%
Olayemi, 2018 − Nigeria	0	12	0.00	[0.00; 26.46]	0.6%
**Random effect meta−analysis**		**279**	**0.00**	**[0.00; 0.32]**	**7.1%**
Heterogeneity: I^2 = 0% [<0.0%; <0.0%], τ^2 = 0, p = 0.9988					
**Past contact_ Unspecified rodents (18 Studies)**					
Bowen, 1975 − Nigeria	0	17	0.00	[0.00; 19.51]	0.7%
Carey, 1972 − Nigeria	0	86	0.00	[0.00; 4.20]	0.9%
Demby, 2001 − Guinea	4	49	8.16	[2.27; 19.60]	0.9%
Demby, 2001 − Guinea	4	309	1.29	[0.35; 3.28]	1.0%
Demby, 2001 − Guinea	96	884	10.86	[8.89; 13.10]	1.0%
Gonzalez, 1983 − Central African Republic	0	65	0.00	[0.00; 5.52]	0.9%
Gonzalez, 1983 − Central African Republic	28	153	18.30	[12.52; 25.35]	1.0%
Gonzalez, 1989 − Cameroon	4	126	3.17	[0.87; 7.93]	1.0%
Mariën, 2017 − Guinea	318	1139	27.92	[25.33; 30.62]	1.0%
McCormick, 1987 − Sierra Leone	162	1435	11.29	[9.70; 13.04]	1.0%
Okoror, 2005 − Nigeria	102	218	46.79	[40.02; 53.65]	1.0%
Okoror, 2005 − Nigeria	0	758	0.00	[0.00; 0.49]	1.0%
Safronetz, 2013 − Mali	1	204	0.49	[0.01; 2.70]	1.0%
Saluzzo, 1988 − Senegal	1	71	1.41	[0.04; 7.60]	0.9%
Saluzzo, 1988 − Senegal	1	81	1.23	[0.03; 6.69]	0.9%
Saluzzo, 1988 − Senegal	0	214	0.00	[0.00; 1.71]	1.0%
Saluzzo, 1988 − Senegal	3	419	0.72	[0.15; 2.08]	1.0%
Saluzzo, 1988 − Senegal	14	655	2.14	[1.17; 3.56]	1.0%
**Random effect meta−analysis**		**6883**	**4.39**	**[1.06; 9.50]**	**17.2%**
Heterogeneity: I^2 = 98.4% [98.1%; 98.7%], τ^2 = 0.0434, p < 0.0001					
**Overall random effect meta−analysis**		**21891**	**3.19**	**[1.98; 4.62]**	**100.0%**
Residual heterogeneity: I^2 = 94.8% [94.2%; 95.4%], p = 0					

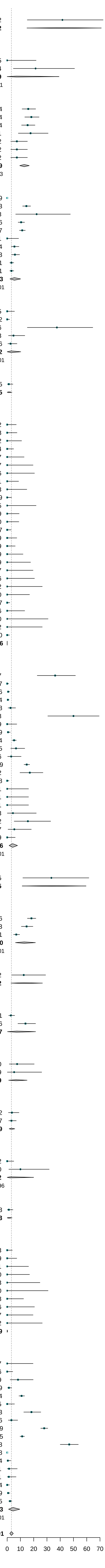

Reference

1. Agbonlahor DE, Erah A, Agba IM, Oviasogie FE, Ehiaghe AF, Wankasi M, et al. Prevalence of Lassa virus among rodents trapped in three South-South States of Nigeria. J Vector Borne Dis. 2017; 5.

2. Boiro I, Lomonossov NN, Sotsinski VA, Constantinov OK, Tkachenko EA, Inapogui AP, et al. [Clinico-epidemiologic and laboratory research on hemorrhagic fevers in Guinea]. Bull Soc Pathol Exot Filiales. 1987;80: 607–612.

3. Bowen GS, Wulff H, Casals J, Noonan A, Downs WG. Lassa fever in Onitsha, East Central State, Nigeria, in 1974. 1975; 6.

4. Carey DE, Kemp GE, White HA, Pinneo L, Addy RF, Fom ALMD, et al. Lassa fever Epidemiological aspects of the 1970 epidemic, Jos, Nigeria. Trans R Soc Trop Med Hyg. 1972;66: 402–408. doi:10.1016/0035-9203(72)90271-4

5. Coulibaly-N'Golo D, Allali B, Rieger T, Akoua-Koffi C. Novel Arenavirus Sequences in Hylomyscus sp. and Mus (Nannomys) setulosus from Coˆ te d'Ivoire: Implications for Evolution of Arenaviruses in Africa. PLoS ONE. 2011;6: 9.

6. Demartini JC, Green DE, Monath TP. Lassa virus infection in Mastomys natalensis in Sierra Leone. 1975; 12.

7. Demby AH, Inapogui A, Kargbo K, Koninga J, Kourouma K, Kanu J, et al. Lassa fever in Guinea: II. Distribution and prevalence of Lassa virus infection in small mammals. Vector Borne Zoonotic Dis. 2001;1: 283–297. doi:10.1089/15303660160025912

8. Fair J, Jentes E, Inapogui A, Kourouma K, Goba A, Bah A, et al. Lassa Virus-Infected Rodents in Refugee Camps in Guinea: A Looming Threat to Public Health in a Politically Unstable Region. Vector-Borne and Zoonotic Diseases. 2007;7: 167–171. doi:10.1089/vbz.2006.0581

9. Fichet-Calvet E, Lecompte E, Koivogui L, Soropogui B, Doré A, Kourouma F, et al. Fluctuation of Abundance and Lassa Virus Prevalence in *Mastomys natalensis* in Guinea, West Africa. Vector-Borne and Zoonotic Diseases. 2007;7: 119–128. doi:10.1089/vbz.2006.0520

10. Fichet-Calvet E, Becker-Ziaja B, Koivogui L, Günther S. Lassa Serology in Natural Populations of Rodents and Horizontal Transmission. Vector-Borne and Zoonotic Diseases. 2014;14: 665–674. doi:10.1089/vbz.2013.1484

11. Gonzalez JP, McCormick JB, Saluzzo JF, Herve JP, Georges AJ, Johnson KM. An arenavirus isolated from wild-caught rodents (Pramys species) in the Central African Republic. Intervirology. 1983;19: 105–112. doi:10.1159/000149344

12. Gonzalez JP, Josse R, Johnson ED, Merlin M, Georges AJ, Abandja J, et al. Antibody prevalence against haemorrhagic fever viruses in randomized representative central African populations. Research in Virology. 1989;140: 319–331. doi:10.1016/S0923-2516(89)80112-8

13. Keenlyside RA, McCormick JB, Webb PA, Smith E, Elliott L, Johnson KM. Case-control study of Mastomys natalensis and humans in Lassa virus-infected households in Sierra Leone. Am J Trop Med Hyg. 1983;32: 829–837. doi:10.4269/ajtmh.1983.32.829

14. Kouadio L, Nowak K, Akoua-Koffi C, Weiss S, Allali BK, Witkowski PT, et al. Lassa Virus in Multimammate Rats, Côte d'Ivoire, 2013. Emerg Infect Dis. 2015;21: 1481–1483. doi:10.3201/eid2108.150312

15. Lalis A, Leblois R, Lecompte E, Denys C, ter Meulen J, Wirth T. The Impact of Human Conflict on the Genetics of Mastomys natalensis and Lassa Virus in West Africa. Mores CN, editor. PLoS ONE. 2012;7: e37068. doi:10.1371/journal.pone.0037068

16. Lecompte E, Fichet-Calvet E, Daffis S, Koulémou K, Sylla O, Kourouma F, et al. Mastomys natalensis and Lassa Fever, West Africa. Emerg Infect Dis. 2006;12: 1971–1974. doi:10.3201/eid1212.060812

17. Leski TA, Stockelman MG, Moses LM, Park M, Stenger DA, Ansumana R, et al. Sequence Variability and Geographic Distribution of Lassa Virus, Sierra Leone - Volume 21, Number 4—April 2015 - Emerging Infectious Diseases journal - CDC. 2015 [cited 26 Oct 2019]. doi:10.3201/eid2104.141469

18. Mariën J, Borremans B, Gryseels S, Soropogui B, De Bruyn L, Bongo GN, et al. No measurable adverse effects of Lassa, Morogoro and Gairo arenaviruses on their rodent reservoir host in natural conditions. Parasites Vectors. 2017;10: 210. doi:10.1186/s13071-017-2146-0

19. McCormick JB, Webb PA, Krebs JW, Johnson KM, Smith ES. A Prospective Study of the Epidemiology and Ecology of Lassa Fever. J Infect Dis. 1987;155: 437–444. doi:10.1093/infdis/155.3.437

20. Monath TP, Newhouse VF, Kemp GE, Setzer HW, Cacciapuoti A. Lassa virus isolation from Mastomys natalensis rodents during an epidemic in Sierra Leone. Science. 1974;185: 263–265. doi:10.1126/science.185.4147.263

21. Okoror LE, Esumeh FI, Agbonlahor DE, Umolu PI. Lassa virus: Seroepidemiological survey of rodents caught in Ekpoma and environs. Trop Doct. 2005;35: 16–17. doi:10.1258/0049475053001912

22. Olayemi A, Cadar D, Magassouba N, Obadare A, Kourouma F, Oyeyiola A, et al. New Hosts of The Lassa Virus. Sci Rep. 2016;6: 25280. doi:10.1038/srep25280

23. Olayemi A, Obadare A, Oyeyiola A, Igbokwe J, Fasogbon A, Igbahenah F, et al. Arenavirus Diversity and Phylogeography of *Mastomys natalensis* Rodents, Nigeria. Emerg Infect Dis. 2016;22: 687–690. doi:10.3201/eid2204.150155

24. Olayemi A, Oyeyiola A, Obadare A, Igbokwe J, Adesina AS, Onwe F, et al. Widespread arenavirus occurrence and seroprevalence in small mammals, Nigeria. Parasites Vectors. 2018;11: 416. doi:10.1186/s13071-018-2991-5

25. Safronetz D, Lopez JE, Sogoba N, Traore' SF, Raffel SJ, Fischer ER, et al. Detection of Lassa virus, Mali. Emerging Infect Dis. 2010;16: 1123–1126. doi:10.3201/eid1607.100146

26. Safronetz D, Sogoba N, Lopez JE, Maiga O, Dahlstrom E, Zivcec M, et al. Geographic Distribution and Genetic Characterization of Lassa Virus in Sub-Saharan Mali. PLoS Negl Trop Dis. 2013;7. doi:10.1371/journal.pntd.0002582

27. Saluzzo JF, Adam F, McCormick JB, Digoutte JP. Lassa Fever Virus in Senegal. Journal of Infectious Diseases. 1988;157: 605–605. doi:10.1093/infdis/157.3.605

28. Wulff H, Fabiyi A, Monath TP. Recent isolations of Lassa virus from Nigerian rodents. Bull World Health Organ. 1975;52: 609–613.

29. Yadouleton A, Agolinou A, Kourouma F, Saizonou R, Pahlmann M, Bedié SK, et al. Lassa Virus in Pygmy Mice, Benin, 2016–2017. Emerg Infect Dis. 2019;25: 1977–1979. doi:10.3201/eid2510.180523
