# Supplementary figures and images for: Systematic review and meta-analysis of the epidemiology of Lassa virus in humans, rodents and other mammals in sub-Saharan Africa
Source: PLoS Negl Trop Dis. 2020 Aug 26;14(8):e0008589. doi: 10.1371/journal.pntd.0008589 (PMC7478710; doi:10.1371/journal.pntd.0008589)

S3 Fig: Funnel plot for publication for Lassa virus case fatality rate in humans

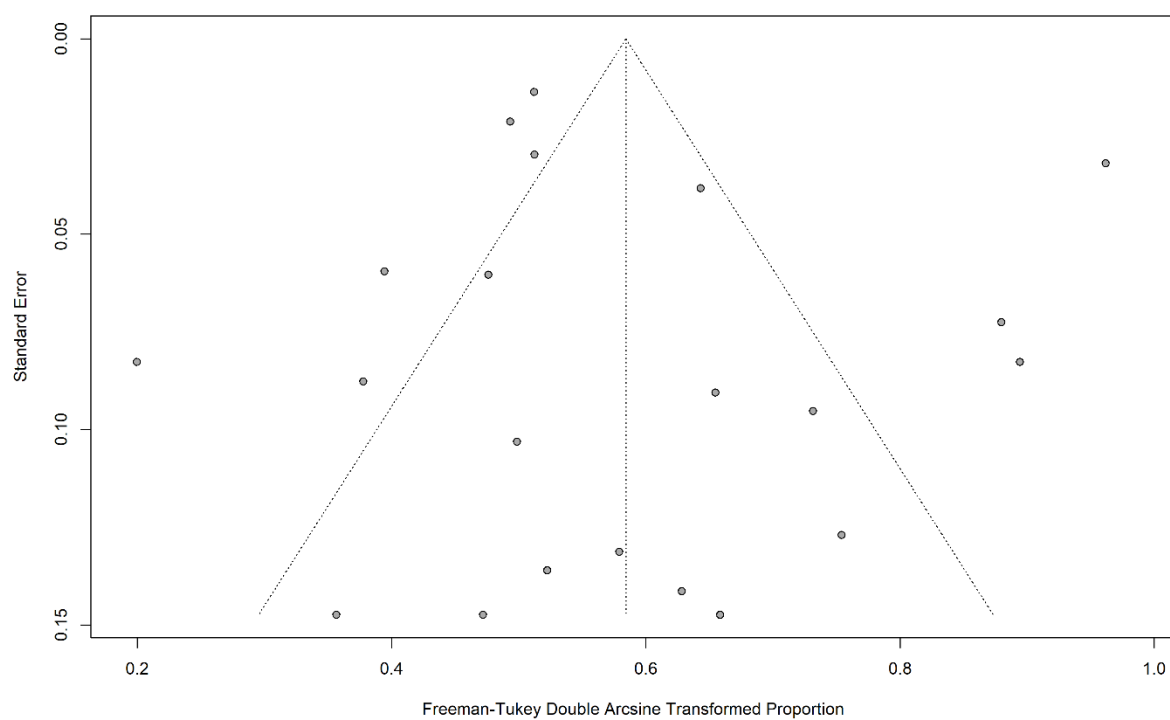

Supplement: S3 Fig — (PDF) [file pntd.0008589.s012.pdf]

S4 Fig: Funnel plot for publication for Lassa virus prevalence in humans

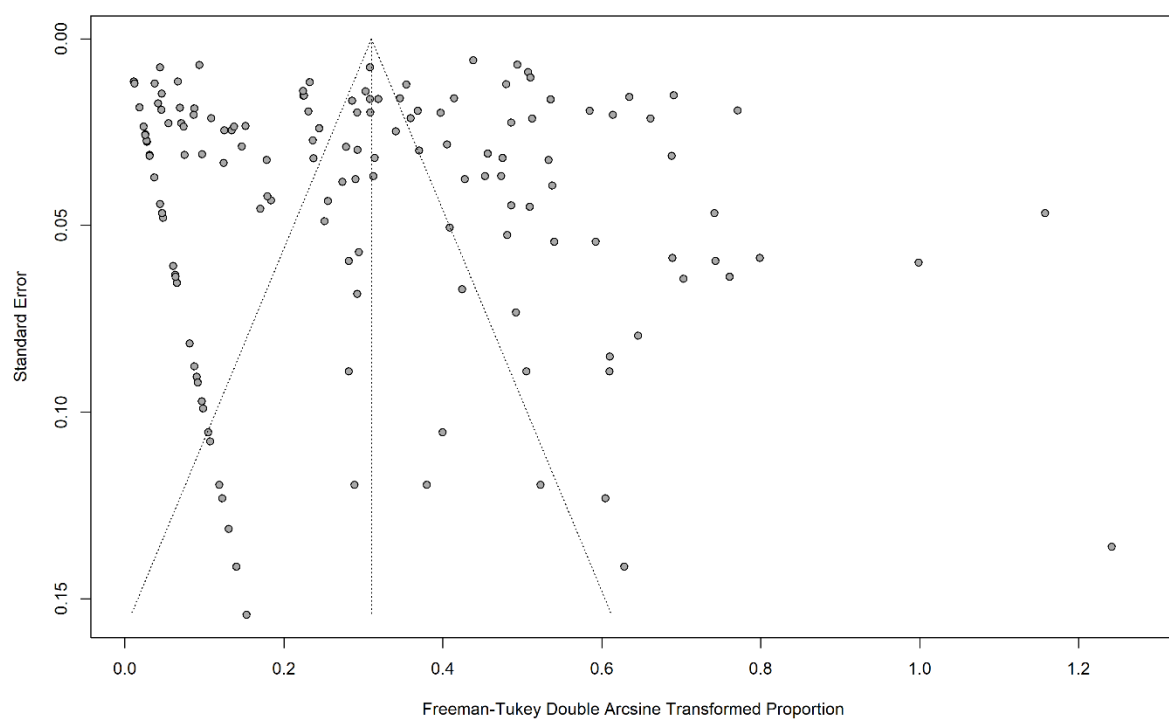

Supplement: S4 Fig — (PDF) [file pntd.0008589.s013.pdf]

S5 Fig: Funnel plot for publication for Lassa virus prevalence in rodents

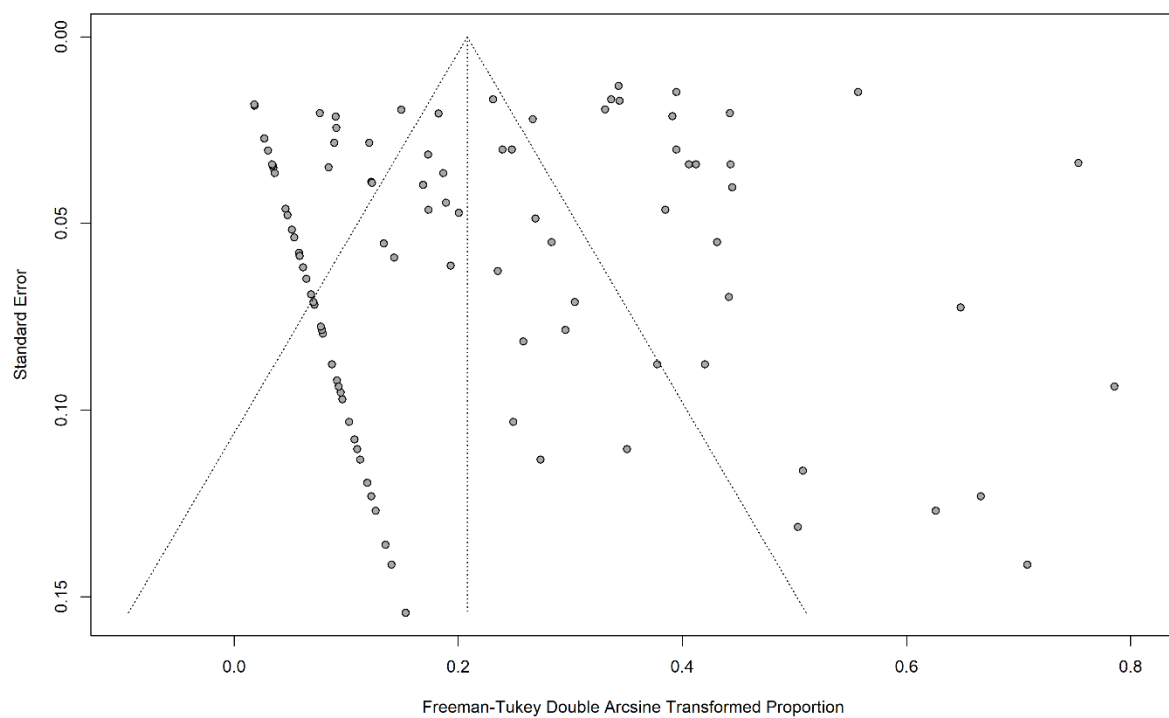

Supplement: S5 Fig — (PDF) [file pntd.0008589.s014.pdf]

S6 Fig: Funnel plot for publication for Lassa virus prevalence in others mammals

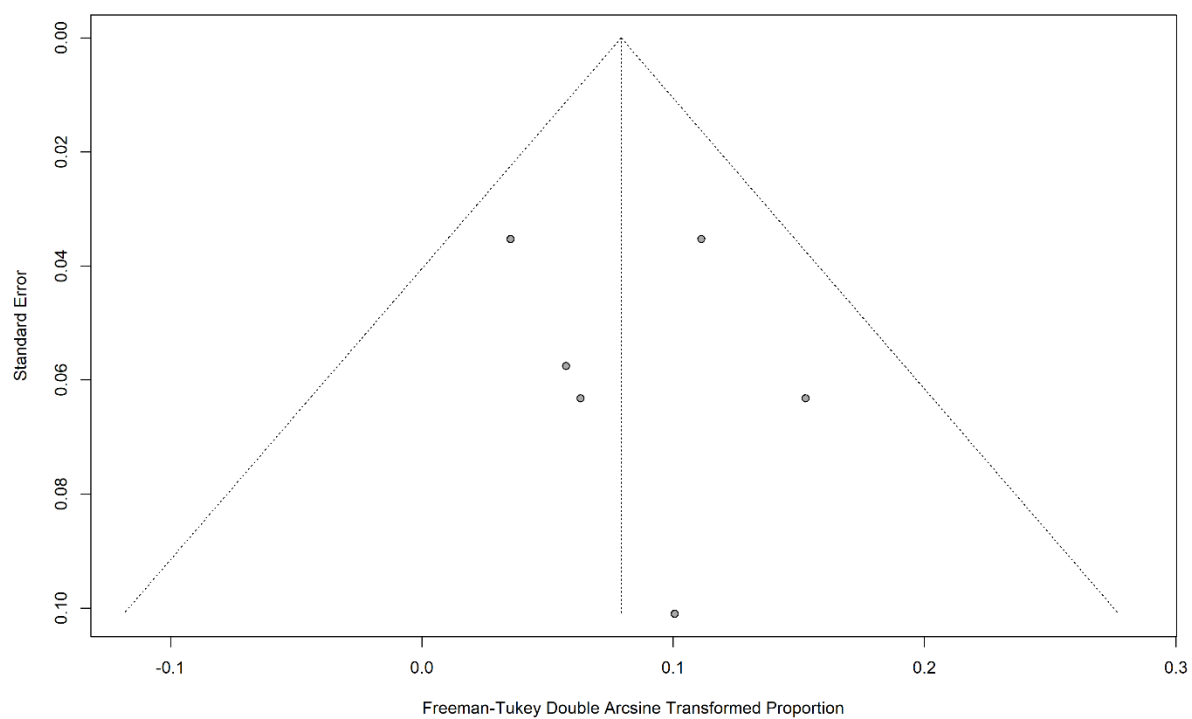

Supplement: S6 Fig — (PDF) [file pntd.0008589.s015.pdf]
